# Supplementary material for: Effects of facial skin pigmentation on social judgments in a Mexican population
Source: PLoS One. 2023 Nov 30;18(11):e0279858. doi: 10.1371/journal.pone.0279858 (PMC10688750; doi:10.1371/journal.pone.0279858)
Supplement: S1 Fig — PERLA scale distribution of the 700 participant’s own skin color ratings. The color levels go from 1 to 10, being 1 the lightest and 10 the darkest. 11 (1.6%) picked skin tone level 1, 36 (5.1%) skin tone level 2, 42 (6%) skin tone level 3, 240 (34.3%) skin tone level 4, 201 (28.8%) skin tone level 5, 102 (14.6%) skin tone level 6, 45 (6.4%) skin tone level 7, 14 (2%) skin tone level 8, 7 (1%) skin tone level 9, and 1 (0.1%) skin tone level 10. (PDF) [file pone.0279858.s001.pdf]

S1 Fig. Participants' self-perceived skin color ratings.

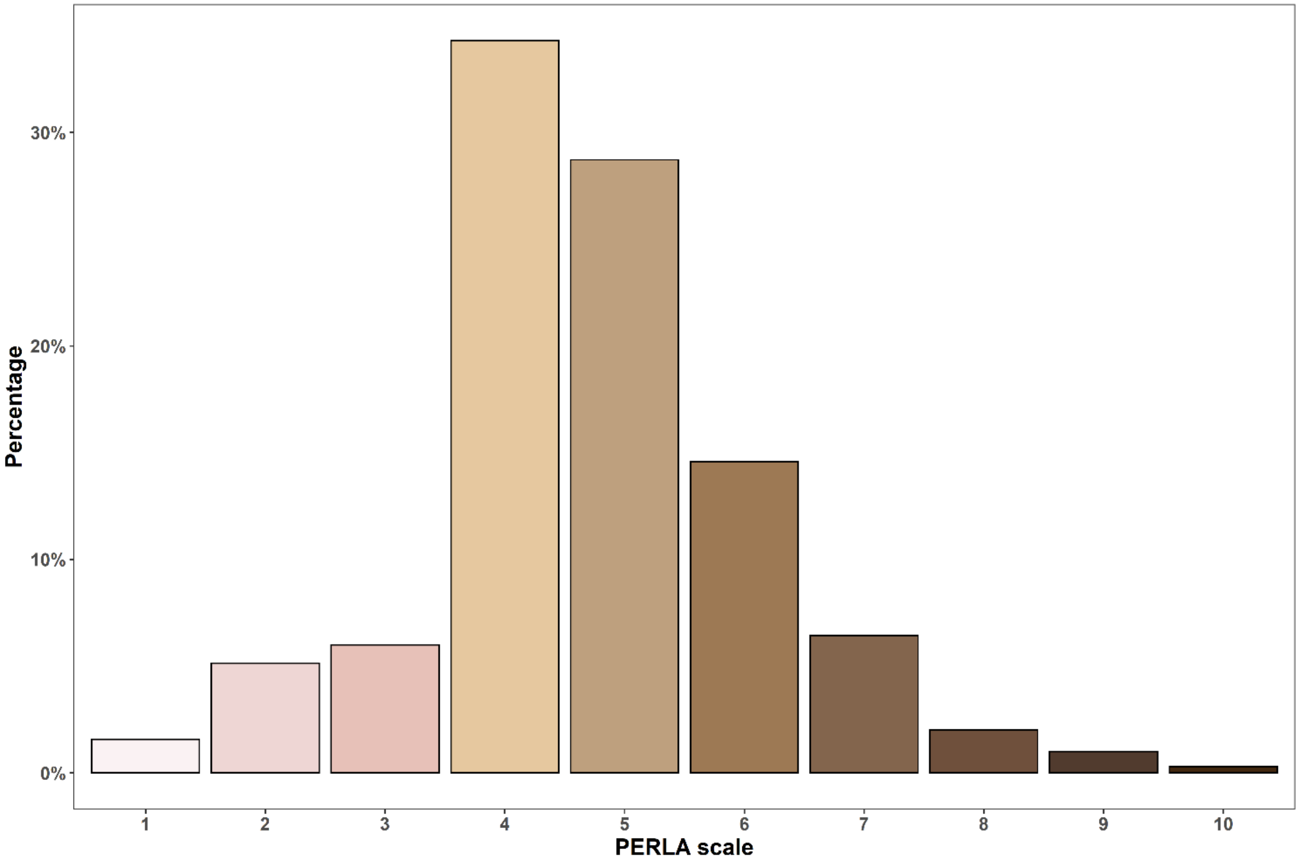

S1 Fig. PERLA scale distribution of the 700 participants' own skin color ratings. The color levels go from 1 to 10, being 1 the lightest and 10 the darkest. 11 (1.6 %) picked skin tone level 1, 36 (5.1%) skin tone level 2, 42 (6%) skin tone level 3, 240 (34.3%) skin tone level 4, 201 (28.8%) skin tone level 5, 102 (14.6%) skin tone level 6, 45 (6.4%) skin tone level 7, 14 (2%) skin tone level 8, 7 (1%) skin tone level 9, and 1 (0.1%) skin tone level 10.
