# Supplementary material for: Effects of facial skin pigmentation on social judgments in a Mexican population
Source: PLoS One. 2023 Nov 30;18(11):e0279858. doi: 10.1371/journal.pone.0279858 (PMC10688750; doi:10.1371/journal.pone.0279858)
Supplement: S1 Table — Estimates (Est.) with 95% confidence intervals (CI) of the fixed predictors of the models for: (a) Perceived attractiveness, (b) Perceived trustworthiness, (c) Perceived health, (d) Perceived dominance, (e) Perceived aggressiveness, and (f) Perceived masculinity. Note: (⸸) The dominance best fitted model did not include the skin color transformation predictor, therefore the estimates shown represent the main effect of the natural skin color group only. (PDF) [file pone.0279858.s002.pdf]

S1 Table. Models estimates with all participants included.

S1 Table. Estimates (Est.) with 95% Confidence Intervals (CI) of the fixed predictors of the models with all participates included for: (a) Attractiveness perception, (b) Trustworthiness perception, (c) Perceived health, (d) Dominance perception, (e) Aggressiveness perception, and (f) Masculinity perception.

	**Me'Phaa**			**European American**		
	**NAT**	**LSCt**	**DSCt**	**NAT**	**LSCt**	**DSCt**
	Est. [95%CI]	***Est. [95%CI]***	***Est. [95%CI]***	***Est. [95%CI]***	***Est. [95%CI]***	***Est. [95%CI]***
a) Attractiveness perception	2.50 [2.21 - 2.82]	3.75 [3.44- 4.09]	2.93 [2.68 - 3.19]	4.26 [3.78- 4.79]	4.74 [4.35- 5.17]	3.26 [2.99- 3.55]
b) Trustworthiness perception	4.18 [4.01- 4.35]	4.45 [4.27 - 4.63]	4.29 [4.12 - 4.46]	4.73 [4.54 - 4.92]	5.04 [4.84 - 5.24]	4.85 [4.67 - 5.05]
c) Perceived health	4.27 [3.82 - 4.78]	5.49 [5.18 - 5.82]	5.19 [4.84 - 5.57]	5.08 [4.54 - 5.67]	5.94 [5.60 - 6.29]	5.04 [4.70 - 5.41]
d) Dominance perception	5.27 [4.91 - 5.67]	⸸	⸸	4.28 [3.96 - 4.63]	⸸	⸸
e) Aggressiveness perception	5.46 [5.24 - 5.70]	5.09 [4.87 - 5.32]	5.38 [5.17 - 5.59]	5.17 [4.95 - 5.40]	4.76 [4.55 - 4.99]	4.55 [4.36 - 4.74]
f) Masculinity perception	6.16 [5.77 - 6.58]	5.17 [4.86 - 5.50]	5.93 [5.57 - 6.33]	5.13 [4.80 - 5.49]	4.83 [4.54 - 5.14]	4.88 [4.57 - 5.21]

Note: (⸸) The dominance best fitted model did not include the skin color transformation predictor, therefore the estimates shown represent the main effect of the natural skin color group only.
