# Supplementary material for: Effects of facial skin pigmentation on social judgments in a Mexican population
Source: PLoS One. 2023 Nov 30;18(11):e0279858. doi: 10.1371/journal.pone.0279858 (PMC10688750; doi:10.1371/journal.pone.0279858)
Supplement: S2 Table — Pearson’s correlation between the natural versions scores of attractiveness, trustworthiness, perceived health, dominance, masculinity, and aggressiveness. *p<0.05, **p<0.01, ***p<0.001. (PDF) [file pone.0279858.s003.pdf]

S2 Table. Pearson's correlation analysis between the scores of all perceptions rates of natural versions only.

Table S1.3
Pearson's correlation between the natural versions scores of all six perceptions rated.

	Dominance perception	Masculinity perception	Perceived health	Trustworthiness perception	Attractiveness perception
**Aggressiveness perception**	.21***	.29***	.10***	.06*	.06*
**Dominance perception**		.48***	.16***	.08**	.15***
**Masculinity perception**			.24***	.24***	.15***
**Perceived health**				.44***	.46***
**Trustworthiness perception**					.41***

*$p<0.05$, **$p<0.01$, ***$p<0.001$
