# Supplementary material for: Effects of facial skin pigmentation on social judgments in a Mexican population
Source: PLoS One. 2023 Nov 30;18(11):e0279858. doi: 10.1371/journal.pone.0279858 (PMC10688750; doi:10.1371/journal.pone.0279858)
Supplement: Attachment — Submitted filename: PONE-D-22-34514_R1_reviewer-revPF.pdf [file pone.0279858.s005.pdf]

PLOS ONE

Effects of facial skin pigmentation on social judgments in a Mexican population

--Manuscript Draft--

**Manuscript Number:**	PONE-D-22-34514R1
**Article Type:**	Research Article
**Full Title:**	Effects of facial skin pigmentation on social judgments in a Mexican population
**Short Title:**	Effects of facial skin pigmentation on social judgments in a Mexican population
**Corresponding Author:**	Isaac G-Santoyo<br>Universidad Nacional Autonoma de Mexico Facultad de Psicologia<br>CDMX, CDMX MEXICO
**Keywords:**	Skin Color Pigmentation, Colorism, Mexican social perceptions, social learning, sexually dimorphic traits, Skin color social and biological evolution
**Abstract:**	People quickly and involuntarily form impressions of others based on their facial physical attributes, which can modulate critical social interactions. Skin pigmentation is one of the most variable and conspicuous facial traits among human populations. Empirical evidence suggests that these variations reflect ancestral ecological selective pressures balancing cutaneous vitamin D synthesis with the protection of the dermis from ultraviolet radiation. Nevertheless, skin pigmentation may currently be subject to additional selective pressures. For instance, the colonial era in Central and South America developed a highly stratified society based on ethnic origins, and light skin pigmentation became associated with social status. Facial skin pigmentation is also sexually dimorphic, with males tending to exhibit darker skin than females, a difference that could be driven by sexual selection. To explore whether social learning and sexual selection represent additional selective pressures on skin pigmentation, we tested how this facial trait influences fundamental social perceptions in a Mexican population (N = 700, 489 female). We sampled facial images of eight European American males with natural light facial skin pigmentation and eight males from an indigenous pre-Columbian community from Mexico, the Me'Phaa, with naturally darker facial skin pigmentation. We produced stimuli from these images by interchanging skin pigmentation while preserving the facial shape. Stimuli were rated on attractiveness, trustworthiness, perceived health, dominance, aggressiveness, and femininity/masculinity. We found that the natural light-skinned faces of European American males were perceived as more attractive, trustworthy, and healthy but less dominant than the natural dark Me'phaa faces. Furthermore, the interchange of facial skin color in these original groups reversed rates of these perceptions, mainly the attractiveness perception. These results partially support the hypothesis that dark facial skin color may aid male-male contest competition. However, they more strongly support that light skin pigmentation became associated with favorable attributes related to pro-sociality due to possible social learning in this Mexican population.
**Order of Authors:**	Jaaziel Martínez-Ramírez
	David Puts
	Javier Nieto
	Isaac G-Santoyo
**Response to Reviewers:**	Response to edito and reviewers<br><br>Effects of facial skin pigmentation on social judgments in a Mexican population<br>Jaaziel Martínez-Ramírez, David Puts , Javier Nieto, Isaac G-Santoyo*<br><br>*Corr. Author.<br><br>Dear Editor,<br><br>We thank the reviewers and you for their time and the generous comments on the manuscript. We have edited the manuscript to address all of their concerns. You will find each revised comment below. A new version of the R-Markdown where you can

Powered by Editorial Manager® and ProduXion Manager® from Aries Systems Corporation

find some new plots and analyses is also available.

Reviewer 1: Edward Telles

-This is an important topic. The increasing interest and research on the topic is largely about the growing acknowledgment of the importance of race and color in Mexican society, by scientists and the general population. However, this analysis though seems to be largely of an evolutionary biological view with only a nod to ongoing racism and discrimination. Indeed, I could not find any use of “racism” but rather the use of “colorism.” When colorism is acknowledged, the authors usually refer to historical colorism under colonialization, which is learned across generations. As if racism and discrimination no longer exist. There is no consideration of the role of a Western ideology of race and racism and particularly, how Mexican elites, narratives, media, etc, shaped an ideology of mestizaje, which downplayed racism. See a considerable and growing literature by Patricio Solis, Regina Martinez Casas, Alan Knight and several others that shows the history of race in Mexico and how individuals use color to evaluate others in the labor market and other arenas. Indeed, the authors cite the PERLA studies but seem to overlook its findings about discrimination and inequality. Contrary to their claim that a social classification system based on phenotype no longer exists (p.5), these studies show that it is very profound in Mexican society, like it is throughout the Americas. I found it odd for, example, the claim that indigenous women chose to mate with Spanish men simply because they wanted to give their offspring better chances. What about the role of power or even rape?

Response:

We appreciate this comment. Indeed, our manuscript is in line with the perspective of the reviewer. The expression of various forms of racism in modern Mexican society is evident, leading to profound discrimination against individuals with physical attributes resembling pre-Columbian populations, with skin color being the most prominent attribute. Correspondingly, our study provides experimental evidence for the first time in a Mexican population that a relationship exists between "light" skin color and subjective preferences for favorable social traits such as attractiveness and reliability. Our manuscript aims to neither conceal nor distort this social effect; instead, it contributes to the extensive field of study in this area.

Based on the framework of behavioral ecology, social evolutionary psychology, and cultural evolution, our study aimed to test two non-mutually exclusive hypotheses explaining the effects of facial skin pigmentation on social judgments in the Mexican population. In the so-called social learning hypothesis, we proposed that this psychological mechanism underlies the preferences for lighter skin color and its association with social benefits, prestige, or deference. This association originated during the colonial period through the racial/color hierarchy system known as "castas," which placed the European colonizers at the top of the social hierarchy and the indigenous population at the bottom.

Therefore, we propose that skin color has led to the hierarchization of social benefits within the Mexican population through an extensive learning process that originated with the conquest. This process has influenced the ancestral evolutionary function of this physical attribute, from a mechanism of filtering UV radiation or synthesizing vitamin D to being an essential factor in social selection. In line with this, we do not deny that racism, colorism, and discrimination are also outcomes of this process and that nowadays, the cultural preferences for lighter skin color and other European phenotypical features may be reinforced by these sociological mechanisms. Our explanation from an ecological/evolutionary perspective does not contradict other authors from different fields of knowledge. Instead, it complements its study, not only due to the implementation of an experimental design that modifies skin color and controls for other confounding variables but also because of the theoretical argument that unifies the same observed phenomenon of racism through its observation in one of its facets related to subjective preferences determining different types of social interaction.

Additionally, although we did not explicitly use "racism," we considered "colorism" a more appropriate word to describe the effect observed regarding skin color, since we did not delve further into the implications of racism or other types of discrimination as it

falls outside the scope of this study. Nonetheless, colorism, a form of prejudice or discrimination based on skin color, is evident, whereby individuals are treated differently based on this physical attribute, with lighter skin tones often being favored or perceived as more desirable than those with darker skin tones. Colorism can manifest both within racial and ethnic groups (as observed in our study) and between them, resulting in various negative impacts, such as reinforcing harmful stereotypes and creating disparities in education, employment, and social status. It is often interconnected with broader issues of racism and discrimination, making it essential to address in pursuing social justice and equality.
Consequently, we have extensively rewritten a significant portion of the introduction and discussion sections to clarify our stance (for example see lines 113-131 and 502-513). . Moreover, we have expanded the information regarding previous studies demonstrating different racial features within the current Mexican population. Here, we have emphasized modifying the phrase "no longer exist" to more clearly convey that these preferences continue to permeate the psyche of the contemporary Mexican population, at least based on our evaluated sample (>700).

-The authors focus on the evaluations according to particular personality dimensions. They should consider the role of stereotypes as in how indigenous people are considered ugly -score low on attractiveness (note the recent reactions to the actress Yalitza Aparicio) and stereotypes about not being trusted and being sickly (two other dimensions studied). These findings are consistent with the use of ethnic/racial stereotypes. Aggressiveness may also be according to the idea of indigenous as savages. The dimensions of masculinity and dominance are less clear. Racism and stereotypes about indigenous people should certainly be considered at least as a hypothesis, which may compete with hypotheses about social learning, etc. The color ratings of the European faces is also consistent with ratings as they may appear more indigenous.

Response: We appreciate this comment. Indeed, our results could be closely linked to racism and stereotypes; again, our intention has not been to deny these concepts but to complement them. In this line, we do not posit racism and stereotypes as a hypothesis competing with the hypothesis of social learning but rather as a possible consequence of this historical-social learning, which determines what is deemed "good" or "bad" based on skin color.
What is particularly interesting here is that the rater's participants had no information about the ethnicity of the presented stimuli. Therefore, we filtered out any information that could promote stereotypes by the evaluators beforehand, to observe the exclusive effect of skin color. In our experiment (please see the referred web page in line 268- labneuroecologiacog.com), we did not use labels or any other indication to suggest that the face belonged to a European American or a Me'Phaa man. Hence, if a rater had any stereotype bias towards Indigenous people, it would be harder for them to recognize the face as Indigenous. We also applied masks to all facial stimuli to minimize potential confounding factors.
Consequently, our results demonstrate a shift in participants' perceptions when skin color is oppositely modified to the original ethnic group. This change shows that a light-skinned face was evaluated positively on socially favorable dimensions (such as attractiveness and trustworthiness) and negatively on "unfavorable" dimensions. This effect was regardless of the stimuli' facial shape, the rater's preexisting stereotypical prejudices, the original ethnic group of the facial stimuli, or the rater's self-perception of skin color or ethnicity. We clarified this information in the discussion, also emphasizing that the approximations of racism and stereotypes evaluated here could be, in part, a consequence of this historical-social learning.

-As far as the analysis, I think it is mostly well done but I am surprised that the color or the ethnicity of the raters was not controlled (See Hill, Mark E. "Race of the interviewer and perception of skin color: Evidence from the multi-city study of urban inequality." American Sociological Review (2002): 99-108. The authors claim that the rater color was concentrated in a few colors and so they didn’t control that. I don’t understand why that should be important. Also, if raters are indigenous, they may evaluate in a different way. By the way, there seems to be a hesitancy to call the Me’Pha “indigenous.” For these Mexican raters, I imagine stereotypes are based on indigeneity and they may have little or no familiarity with that particular ethnic group.

Powered by Editorial Manager® and ProduXion Manager® from Aries Systems Corporation

Response:
The data collected from self-reported ethnicities and PERLA scale responses indicated that 84% of the raters identified as Mestizos, and 78% chose levels 4-6 on the PERLA scale. The remaining 16% and 22% (respectively) were divided among four other ethnic groups and seven skin color levels. It's worth mentioning that some combinations had very low percentages, such as the self-reported ethnicity "Afro-American" at only 0.3%. This low percentage of other ethnic groups and scores on the PERLA scale suggests that our sample is quite homogeneous. Here, variables with low representativeness, such as these two mentioned, lead to heterogeneity of variance due to the unbalanced data in the sample size of each predictor included (i.e., ethnicity with five levels and PERLA scale with 11 levels). This unbalanced data and over-parametrization are significant structural violations when using linear models (Zuur, Leno, & Elphick, 2010; Faraway, 2016). However, following the reviewer's observation, we conducted the method described by Zuur, Leno, and Elphick (2010) to investigate the possible effects of these two variables. After the model validation process, we plotted Pearson residuals of each selected model against participants' self-reported ethnicities and the PERLA scale responses for self-perceived skin color (the box plots can be found in the R Markdown, code lines 263-264 for the attractiveness plots, 455-456 for the dominance plots, 557-558 for the masculinity plots, 681-682 for the aggressiveness plots, 806-807 for the trustworthiness plots, and 936-937 for the perceived health plots). Due to the plots did not show any effect, and to avoid structural errors in the design of linear models, we did not include either of these two predictors. We briefly explained our decision in the statistical analysis section (lines 326-330). Finally, following the reviewer's observation, we already had included self-perception of ethnicity as "Indigenous" rather than particular ethnic groups, such as "Me'Phaa"(Please see line 191).
-I would refer to raters as “raters” or “participant raters rather than “participants.”
Response: We changed the heading “Participants” with “Participant raters” in all manuscript.

References:

Faraway, J. J. (2016). Extending the linear model with R: generalized linear, mixed effects and nonparametric regression models. CRC press.

Zuur, A. F., Ieno, E. N., & Elphick, C. S. (2010). A protocol for data exploration to avoid common statistical problems. Methods in ecology and evolution, 1(1), 3-14.

Reviewer #2: Peter Frost

- “Its main difficulty is that of distinguishing between gendered and ethnic significations of skin color. The two have long coexisted in Mexican society. The authors have tried to control for ethnic significations by varying skin color within each ethnic group. The results of that strategy are mixed. It seems to me that ethnic significations contaminated the raters’ perceptions of the European American faces to a greater degree than they contaminated the raters’ perceptions of the Me’Phaa faces.”

Response:
We appreciate this precise observation. We propose two main reasons why we observed different results in the effects of skin color transformation between the European American and Me'Phaa faces, both closely related to the suggestions made by the reviewer.
The first possibility is that the participants may have rated the stimuli differently due to varying degrees of familiarity with the facial features of the two ethnic groups. Here, raters participants were asked to rate facial stimuli from a closely related ethnic group (Me'Phaa faces) and stimuli from a more distant ethnic group (European American faces). In this case, the effects on subjective perceptions were more evident when changing skin color within the more familiarized groups. However, although we found a similar effect on European American faces, it was not statistically significant in several social perceptions.
The second possibility is attributed to a potential artifact in our method to modify the skin color. In this case, the manipulation process outcomes could have differed slightly

between the European American and Me'Phaa faces. To deal with this, we minimized the standard error by fitting random slopes for skin color transformation for each facial photograph ID. However, it is essential to highlight that there is always a risk of such artifacts in any manipulation process. We added a new paragraph in the discussion summarizing these possible explanations and limitations (lines 549-562).

-There are a few errors:

- The reference to Green and Martin (1990) does not support footnote #27

- The adjective “perceived” should be used in all of the headings, and not just “perceived health.” All of the qualities are perceived.

- Since perceived aggressiveness did not differ significantly between the two groups of faces, it should not be presented in the Discussion section as a perceived quality that differs between the two groups.

-In general, the wording is awkward. I would suggest the following corrections.
-Lines 83- 93 – replace the four sentences with: “The main pigments—melanin, hemoglobin, and carotenoids—absorb and reflect different wavelengths of light within the visible spectrum. Collagen also contributes by scattering light [10]. The skin’s pigments vary not only between individuals and populations but also between men and women, perhaps because of sexual selection [11]. Natural selection initially favored darker skin as a means to protect against the harmful effects of UV radiation in the tropics. The pressure of selection then shifted toward lighter skin among those humans who spread into higher latitudes with lower levels of UV [12-14]”

-Footnote #10 – add:
Edwards, E.A., and S.Q. Duntley. (1939). The pigments and color of living human skin. American Journal of Anatomy 65(1): 1-33. https://doi.org/10.1002/aja.1000650102

-Line 102 – replace “others” with “other”

-Line 110 – replace “preference honestly contributed to reproductive outcomes” with: “preference significantly contributes to reproductive success”

-Lines 128-140 – replace with: “The sex difference is due to exposure of skin tissues to differing ratios of androgens to estrogens, particularly at puberty. Testosterone has a stronger effect than estrogen on melanin synthesis and vascularization of the upper dermis [26].”

-Footnote #26 - add:

Edwards EA, Hamilton JB, Duntley SQ, Hubert G. Cutaneous vascular and pigmentary changes in castrate and eunuchoid men. Endocrinology 1941; 28(1): 119-128. doi:10.1210/endo-28-1-119

Manning JT, Bundred PE, Mather FM. Second to fourth digit ratio, sexual selection, and skin colour. Evolution and Human Behavior 2004; 25(1): 38-50. doi:10.1016/s1090-5138(03)00082-5

-Footnote #27 - the reference to Green and Martin (1990) does not support the statement that “males undergo a more intense facultative pigmentation after sun exposure and retain it for longer periods than females whereas female skin lightens faster after a reduction in sun exposure.”

Replace the reference to Green and Martin (1990) with:

Harvey RG. Ecological factors in skin color variation among Papua New Guineans, American Journal of Physical Anthropology 1985; 66(4): 407-416. doi:10.1002/ajpa.1330660409

-Footnotes #28-30 - add:

Frost P. Preference for darker faces in photographs at different phases of the menstrual cycle: Preliminary assessment of evidence for a hormonal relationship. Perceptual and Motor Skills 1994; 79(1): 507-14. doi:10.2466/pms.1994.79.1.507

Line 140 – replace “have” with “has”

-Line 148 – replace “no-WEIRD” with “non-WEIRD”

-Lines 151-152 – replace with: “In this study, we used an experimental design with a Mexican population to determine whether facial skin color can influence certain social perceptions: attractiveness, …”

-Line 155 – replace “To accomplish this” with “To that end”

-Line 164 – delete the extra comma after “perceptions”

-Line 193 – replace “create” with “created”

-Line 240 – replace “into” with “within”

-Line 253 – replace “prevent” with “made sure”

-Line 254 – insert “not” after “were”

-Line 256 – replace “on” with “of”

-Lines 259-261 – replace with: “… version. In other words, we compared natural European American faces with artificial light-skinned and dark-skinned versions and natural Me’Phaa faces with artificial light-skinned and dark-skinned versions. We could thus measure how the participants perceived differences in facial color independently of the face’s ethnicity.”

-Line 264 – replace “in” with “on”

- 272 – replace “men participants” with “the male participants”

-Line 281 – replace “be” with “have been”

-Line 285 – replace “in” with “on the”

-Line 329 – replace “Attractiveness” with “Perceived attractiveness”

-Line 330 – replace with: “Perceived attractiveness differed significantly between the two groups when they had their natural skin color”

-Line 331 – replace “as” with “more”

-Line 332 – replace “as” with “than”

-Lines 332-333 – replace with: “Furthermore, there were effects when natural skin color was changed within each of the two groups. Lightening the Me’Phaa faces made them more attractive (…), and darkening the European American faces made them less attractive (…). Both groups became slightly more attractive when skin color and skin texture were homogenized (…).

-Lines 343-344 – replace “men participants” with “the male participants”

-Lines 344-345 – replace “women participants” with “did the female participants”

-Table 2 - replace “Models estimates” with “Perception estimates”
- Replace “Attractiveness perception” with “Perceived attractiveness”
- Replace “Trustworthiness perception” with “Perceived trustworthiness”
- Replace “Dominance perception” with “Perceived dominance”
- Replace “Aggressiveness perception” with “Perceived aggressiveness”

Powered by Editorial Manager® and ProduXion Manager® from Aries Systems Corporation

	- Replace “Masculinity perception” with “Perceived masculinity”<br><br>-Line 357 – replace “Trustworthiness” with “Perceived trustworthiness”<br><br>-Line 359 – replace “as” with “more”<br><br>-Line 360 – replace “as” with “than”<br><br>-Line 361-363 – replace with: “In addition, lightening the Me’Phaa faces increased their perceived trustworthiness (…), whereas darkening the European American faces had no effect”<br><br>-Line 383 – replace “Perceived health” with “Perceived healthiness”<br><br>-Line 384 – replace “As in” with “As with”<br><br>-Lines 386-394 – replace with: “On average, natural European American faces were perceived as being 1.19 times healthier (…) than natural Me’Phaa faces (…). When facial skin color was changed within each ethnic group, the changes had different effects on perceptions of the two groups. Lightening the Me’Phaa faces increased their perceived health (…), whereas darkening the European American faces had no effect (…). Perceived health increased slightly for both groups when skin color and skin texture were homogenized.<br><br>-Line 409 – replace “Dominance” with “Perceived dominance”<br><br>-Lines 410-411 – replace with: “Unlike the previous qualities, perceived dominance was lower for European American faces that had their natural skin color”<br><br>-Lines 413-416 – replace with: “in contrast to perceived attractiveness, all faces received a lower dominance rating from male participants (…) than from female participants (…). Finally, we were unable to determine any effect from lightening, darkening, or homogenizing the faces, since these predictors …”<br><br>-Line 420 – replace “Aggressiveness” with “Perceived aggressiveness”<br><br>-Lines 421- 427 – replace with: “Although perceived aggressiveness was lower for the European American faces, the difference was not significant (…). Lightening the Me’Phaa faces made them seem less aggressive (…), but darkening the European American faces also made them seem less aggressive (…). Finally, perceived aggressiveness did not change for either group when skin color and skin texture were homogenized (…)”<br><br>-Line 442 – replace “Masculinity” with “Perceived masculinity”<br><br>-Lines 443-450 – “As with perceived dominance, perceived masculinity was lower for natural European American faces (…) than for natural Me’Phaa faces (…). Lightening the Me’Phaa faces made them seem less masculine (…), but darkening the European American faces had no effect (…). In addition, perceived masculinity did not change for either group when skin color and skin texture were homogenized (…).”<br><br>-Lines 469-472 (sentence) – replace with: “In comparison with natural Me’Phaa faces, natural European American faces were perceived not only as more attractive, healthier, and more trustworthy but also as less dominant and less masculine. They were furthermore perceived as less aggressive, but not significantly so.”<br><br>-Lines 484-486 – replace with: “… Mexican participants rated light-skinned faces more highly on all three aspects of social valence (attractiveness, healthiness, trustworthiness). In contrast, they rated dark-skinned faces more highly on two aspects of social dominance (dominance, masculinity).<br><br>-Line 493 – replace “well-remunerated” with “well-paid”<br><br>-Lines 495-498 (sentence) – “Consequently, a learned association between a physical

trait—in this case, light skin—and social deference might establish such a perception in a population over several generations"

-Line 498 – replace "learning association" with "learned association"

-Lines 505-507 (sentence) – replace with: "Also, in Mexico and other Latin American countries, lightness of skin correlates with years of schooling, hourly earnings, and other indicators of well-being."

-Line 511 – replace "dark skin color faces" with "dark-skinned faces"

-Lines 512-513 - replace "dark skin color faces" with "dark-skinned faces"

-Line 516 – replace the comma before "therefore" with a semi-colon

-Line 518 – insert "sexually" before "dimorphic"

-Lines 528-535 – Replace with: "For example, lightening the Me'Phaa faces made them seem more attractive, healthier, and more trustworthy. Conversely, darkening the European American faces made them seem less attractive. Other perceived qualities, however, did not change systematically when facial skin color was changed. Perceived dominance did not change when changes were made to facial skin color within either group. Lightening the Me'Phaa faces decreased their perceived masculinity and aggressiveness, but darkening the European American faces did not increase their perceived masculinity and aggressiveness."

-Lines 541-557 - Replace with: "Moreover, we not only lightened and darkened facial color but also homogenized it by removing such texture-related aspects as spots, freckles, or wrinkles […]. Our results suggest that skin texture does have an effect, particularly on perceived health. That effect, however, largely exists independently of the effects of skin color. Skin texture might nonetheless interact with skin color in perceptions of light and dark skin, and such interactions could be an exciting avenue for further studies. In addition, unlike the authors of previous studies, we manipulated overall skin color instead of gradually changing each of its aspects (e.g., luminosity, hue) […]. Future studies could examine how each aspect of skin color contributes to the way a population is perceived."

-Lines 558-574 - replace with: "In summary, we show that the Mexican population uses facial color to make certain social judgments. We found that a dark-skinned face is perceived as more dominant and more masculine. Those perceptions may be due to a process of coevolution between the human mind and the sex difference in skin color. One may therefore infer that male skin became darker through male-male competition rather than through female choice. We also found that a light-skinned face is perceived as more attractive, more trustworthy, and healthier. Those perceptions may be due to the association between skin color and social status that prevailed in Mexico during more than three centuries of colonial rule. Today, they are maintained by colorism. We are nonetheless speculating, and more data will be needed. Similar results could be obtained from other Latin American societies, which share a common colonial history and social structure."

Response:

We greatly appreciate the reviewer's effort and time in providing us with precise suggestions, which were timely and significantly improved our manuscript. Therefore, we have rewritten all sentences and added all recommended references as per the reviewer's assessment. Once again, thank you very much.

Reviewer #3: Vojtech Fiala

-The overall positive impression is also promoted by detailed report on the fitted models, availability of the script and data. It seems like a good craft (although I must admit that I have not checked if the 'necessary' rituals of frequentist statistics have been gone through by the authors). While I recommend to study for acceptance (this

being their final destiny), I still find some aspects of it problematic – and I think these should be considered at least by extending/re-writing some paragraphs within this otherwise good manuscript.

-Major objection 1: Why Poisson?

-First, I wonder, why the authors decided to use generalised linear model with the family set to "Poisson". The expectation that the dataset (data of the dependent variable) comes from Poisson distribution is not commented on in the manuscript. Picking 1-9 points is, in my opinion, not a count of rare events within interval (of whatever kind; how the parameters of such a distribution may be set?). While this objection may seem serious, it is not the case (given my expectations about the "intestines" of glm fit that are above the scope of the current text). Nonetheless, the authors should either justify their decision (maybe I am just wrong, and I'd appreciate being proven so, since this may positively affect my own subsequent work) or consider using different distribution for the dependent variable's population, which has been sampled.

Response:
We appreciate this observation. We decided to use GLMMs with Poisson errors due to the nature of our data, which are discrete quantitative data derived from a Likert scale. We did not average the perception data (i.e. among participant scores for each face rated) because we consider that this data treatment could affect the measures of the central tendency of some facial perceptions; this was particularly true for the attractiveness rating distribution, where we observed a positively skewed counts-type distribution (the histograms can be found in the R Markdown, code lines 234-238). Because of this and the discrete nature of our dependent variables, we decided to use Poisson GLMMs instead of Gaussians, which we explained briefly in the statistical analysis section (lines 302-307).

Major objection 2: Culture vs… and biology vs… (?)

Another serious notion that shall be considered during revisions, refer to the lines 163-173 of the manuscript. I put aside that the sentences are long and hard to follow. I have the following concerns: The authors begin by stating that lighter skin that is naturally associated with a given ethnicity may be a cue to favorable characteristic (as they are stereotypically ascribed to a face, not to its colouration itself – colour is cue to ethnicity, which affect the ascribed characteristics). However, the opposite hypothesis would read: Maybe, it does not matter if the face is European/Native American (I mean, the origin of the face) and what matters is lightness (as an exact measure). This contrasting view (while I acknowledge cannot be directly addressed in this study) is not applied here. The authors rather switch from cultural to biological explanation: once the face is darker, it is perceived as more attractive, also dominant, aggressive, etc., due to androgens. Together, this seems for me to not make much sense. Consider rewriting this paragraph (even if it is just to help slow-minded readers, which I eventually may be, to understand).

Response:

Thank you for this observation. In this study, we tested three hypotheses, each of them having its null and alternative prediction. These three hypotheses could be mutually non-exclusive and, therefore, complement each other. In the first one we propose that social learning may be the psychological mechanism underlying the effects of facial skin pigmentation on the social perceptions. Here we sought to test whether light facial color (and the opposite for dark facial color) promotes greater rate values in favorable social attributes, such as reliability, attractiveness or perceived health. This effect would be independent of the of the origin of the face, but only by the skin coloration. In our second hypothesis, we propose that these effects may rather be the results of ancestral sexual selection of an androgen-dependent trait such as skin color. Here, darker faces and not lighter would influence greater rates values in social perceptions such as attractiveness, aggressiveness, masculinity and dominance. If this is supported, we would expect that the experimental modification of the skin pigmentation would change the previous perceptions in both the European American and Me´Phaa faces. This means that, as noticed in the review, the study seeks to determine the role

	of skin color controlling for other facial features like shape. Finally, our third hypothesis suggests that neither lighter nor darker skin color would affect any social perception, neither in the original faces nor in stimuli with exchanged colors<br>We have rewritten this paragraph to make our hypothesis clearer (lines 170-186).<br><br>Minor notes:<br><br>-"There was no effect of the skin color manipulation or color exchange [for the perceived dominance] in any group."<br>This is a surprising result, given the previously identified positive association between darker skin and dominance-related characteristics like masculinity. I suggest the authors to add a paragraph in the discussion in which they comment on the result. Consider the opposite result for perceived aggressiveness, which was not significantly affected by the ethnicity in non-manipulated faces, while it was affected by skin colour manipulation in both the groups in the anticipated direction (the same applies for masculinity).<br><br>Response: As suggested by Torrance et al. (2014) it seems that the facial shape and surface features, such as skin color, contribute differently to judgements of attractiveness and dominance (See Torrance JS, Wincenciak J, Hahn AC, DeBruine LM, Jones BC (2014) The Relative Contributions of Facial Shape and Surface Information to Perceptions of Attractiveness and Dominance. PLoS ONE 9(10): e104415. doi: 10.1371/journal.pone.0104415). They found that the perception of dominance was more strongly affected by facial shape features whereas the opposite was observed for the perception of attractiveness. Yet another possibility is that the skin color and facial shape effects on some of these perceptions interact in such a way that skin color becomes relevant only when the facial shape strongly elicits one of these perceptions. This may be the case for what we observed in the perceptions of masculinity and aggressiveness, since the skin color manipulation affected mainly the Me´Phaa faces which, in turn, were the ones rated as more dominant and masculine. That would mean that skin color cues become relevant for the perception of masculine and aggressiveness only on faces with shapes that strongly elicit these perceptions. We have rewritten a paragraph in the discussion where we summarized this idea (lines 531-548).
**Additional Information:**	
**Question**	**Response**
**Financial Disclosure**<br><br>Enter a financial disclosure statement that describes the sources of funding for the work included in this submission. Review the submission guidelines for detailed requirements. View published research articles from *PLOS ONE* for specific examples.<br><br>This statement is required for submission and **will appear in the published article** if the submission is accepted. Please make sure it is accurate.	The present work was part of the degree requirements for JMR Doctorate thesis at the Programa de Maestría y Doctorado en Psicología, UNAM. JMR received a Doctorate Nacional grant from the Consejo Nacional de Ciencia y Tecnología (CONACyT, CVU: 921005), and the work was supported by founding from UNAM-PAPIIT [grant numbers IA209416, IA207019] and CONACYT Ciencia Básica [grant number 241744]. The funders had no role in study design, data collection and analysis, decision to publish, or preparation of the manuscript.

**Unfunded studies**<br>Enter: *The author(s) received no specific funding for this work.*<br><br>**Funded studies**<br>Enter a statement with the following details:<br>• Initials of the authors who received each award<br>• Grant numbers awarded to each author<br>• The full name of each funder<br>• URL of each funder website<br>• Did the sponsors or funders play any role in the study design, data collection and analysis, decision to publish, or preparation of the manuscript?<br>• **NO** - Include this sentence at the end of your statement: *The funders had no role in study design, data collection and analysis, decision to publish, or preparation of the manuscript.*<br>• **YES** - Specify the role(s) played.<br><br>* typeset	
**Competing Interests**<br><br>Use the instructions below to enter a competing interest statement for this submission. On behalf of all authors, disclose any competing interests that could be perceived to bias this work—acknowledging all financial support and any other relevant financial or non-financial competing interests.<br><br>This statement is required for submission and **will appear in the published article** if the submission is accepted. Please make sure it is accurate and that any funding sources listed in your Funding Information later in the submission form are also declared in your Financial Disclosure statement.<br><br>View published research articles from *PLOS ONE* for specific examples.	The authors have declared that no competing interests exist.

Powered by Editorial Manager® and ProduXion Manager® from Aries Systems Corporation

**NO authors have competing interests**<br><br>Enter: *The authors have declared that no competing interests exist.*<br><br>**Authors with competing interests**<br><br>Enter competing interest details beginning with this statement:<br><br>*I have read the journal's policy and the authors of this manuscript have the following competing interests: [insert competing interests here]*<br><br>* typeset	
**Ethics Statement**<br><br>Enter an ethics statement for this submission. This statement is required if the study involved:<br><br>• Human participants<br>• Human specimens or tissue<br>• Vertebrate animals or cephalopods<br>• Vertebrate embryos or tissues<br>• Field research<br><br>Write "N/A" if the submission does not require an ethics statement.<br><br>General guidance is provided below. Consult the submission guidelines for detailed instructions. **Make sure that all information entered here is included in the Methods section of the manuscript.**	All procedures were approved by the appropriate National Autonomous University of Mexico ethics committee (FPSI/CE/01/2016).

Powered by Editorial Manager® and ProduXion Manager® from Aries Systems Corporation

**Format for specific study types**<br><br>**Human Subject Research (involving human participants and/or tissue)**<br>• Give the name of the institutional review board or ethics committee that approved the study<br>• Include the approval number and/or a statement indicating approval of this research<br>• Indicate the form of consent obtained (written/oral) or the reason that consent was not obtained (e.g. the data were analyzed anonymously)<br><br>**Animal Research (involving vertebrate animals, embryos or tissues)**<br>• Provide the name of the Institutional Animal Care and Use Committee (IACUC) or other relevant ethics board that reviewed the study protocol, and indicate whether they approved this research or granted a formal waiver of ethical approval<br>• Include an approval number if one was obtained<br>• If the study involved *non-human primates*, add *additional details* about animal welfare and steps taken to ameliorate suffering<br>• If anesthesia, euthanasia, or any kind of animal sacrifice is part of the study, include briefly which substances and/or methods were applied<br><br>**Field Research**<br><br>Include the following details if this study involves the collection of plant, animal, or other materials from a natural setting:<br>• Field permit number<br>• Name of the institution or relevant body that granted permission	
**Data Availability**<br><br>Authors are required to make all data underlying the findings described fully available, without restriction, and from the time of publication. PLOS allows rare exceptions to address legal and ethical concerns. See the PLOS Data Policy and FAQ for detailed information.	Yes - all data are fully available without restriction

A Data Availability Statement describing where the data can be found is required at submission. Your answers to this question constitute the Data Availability Statement and **will be published in the article**, if accepted.<br><br>**Important:** Stating 'data available on request from the author' is not sufficient. If your data are only available upon request, select 'No' for the first question and explain your exceptional situation in the text box.<br><br>Do the authors confirm that all data underlying the findings described in their manuscript are fully available without restriction?	
**Describe where the data may be found in full sentences. If you are copying our sample text, replace any instances of XXX with the appropriate details.**<br><br>• If the data are **held or will be held in a public repository**, include URLs, accession numbers or DOIs. If this information will only be available after acceptance, indicate this by ticking the box below. For example: *All XXX files are available from the XXX database (accession number(s) XXX, XXX.)*.<br>• If the data are all contained **within the manuscript and/or Supporting Information files**, enter the following: *All relevant data are within the manuscript and its Supporting Information files.*<br>• If neither of these applies but you are able to provide **details of access elsewhere**, with or without limitations, please do so. For example:<br><br>*Data cannot be shared publicly because of [XXX]. Data are available from the XXX Institutional Data Access / Ethics Committee (contact via XXX) for researchers who meet the criteria for access to confidential data.*<br><br>*The data underlying the results presented in the study are available from (include the name of the third party*	All data, stimuli and codes for statistical analyses are openly available through the Open Science Framework:<br>https://osf.io/6gn5t/?view_only=89b534d1095b4abf87411b64fd6d8e08

and contact information or URL).<br>• This text is appropriate if the data are owned by a third party and authors do not have permission to share the data.<br><br>* typeset	
Additional data availability information:	Tick here if your circumstances are not covered by the questions above and you need the journal’s help to make your data available.

Powered by Editorial Manager® and ProduXion Manager® from Aries Systems Corporation

Manuscript R1_FinalVersion Click here to access/download;Manuscript;Manuscript_VF.docx

Effects of facial skin pigmentation on social judgments in a Mexican population

Jaaziel Martínez-Ramírez[1] , David Puts[2] , Javier Nieto[3] , Isaac G-Santoyo[1,4*]

1. Neuroecology lab, Faculty of Psychology, National Autonomous University of Mexico, Mexico City, Mexico.
2. Department of Anthropology, Pennsylvania State University, Pennsylvania, United States of America.
3. Laboratory of Learning and Adaptation, Faculty of Psychology, National Autonomous University of Mexico, Mexico City, Mexico.
4. Unidad de Investigación en Psicobiología y Neurociencias, National Autonomous University of Mexico, Mexico City, Mexico.

* Corresponding author

E-mail: isantoyo@psicologia.unam.mx (IG-S)

Abstract

People quickly and involuntarily form impressions of others based on their facial physical attributes, which can modulate critical social interactions. Skin pigmentation is one of the most variable and conspicuous facial traits among human populations. Empirical evidence suggests that these variations reflect ancestral ecological selective pressures balancing cutaneous vitamin D synthesis with the protection of the dermis from ultraviolet radiation. Nevertheless, skin pigmentation may currently be subject to additional selective pressures. For instance, the colonial era in Central and South America developed a highly stratified society based on ethnic origins, and light skin pigmentation became associated with social status. Facial skin pigmentation is also sexually dimorphic, with males tending to exhibit darker skin than females, a difference that could be driven by sexual selection. To explore whether social learning and sexual selection represent additional selective pressures on skin pigmentation, we tested how this facial trait influences fundamental social perceptions in a Mexican population (N = 700, 489 female). We sampled facial images of eight European American males with natural light facial skin ~~pigmentation~~ and eight males from an indigenous pre-Columbian community from Mexico, the Me'Phaa, with naturally darker facial skin ~~pigmentation~~. We produced stimuli from these images by ~~interchanging~~ skin pigmentation while preserving the facial shape. Stimuli were rated on attractiveness, trustworthiness, perceived health, dominance, aggressiveness, and femininity/masculinity. We found that the natural light-skinned faces of European American males were perceived as more attractive, trustworthy, and healthy but less dominant than the natural dark Me'phaa faces. Furthermore, ~~the interchange of~~ facial skin color in these original groups ~~reversed rates of these perceptions~~, mainly ~~the attractiveness perception~~. These results

partially support the hypothesis that dark facial skin ~~color~~ may ~~aid male-male contest competition~~. ~~However, they more strongly support~~ that light ~~skin pigmentation~~ became associated with favorable ~~attributes related to pro-sociality due to possible~~ social learning in this Mexican population.

Introduction

Human faces provide rich information about salient attributes, such as mate quality [1,2], social dominance [3], and fecundity [4]. Hence, people quickly and involuntarily form impressions of others based on their facial appearance [5,6], which in turn may influence important social outcomes [7,8]. Skin pigmentation is one of the most conspicuous and variable facial traits between human populations [9]. The main pigments—melanin, hemoglobin, and carotenoids—absorb and reflect different wavelengths of light within the visible spectrum and collagen also contributes by scattering light [10,11]. The skin's pigments vary not only between individuals and populations but also between men and women, perhaps because of sexual selection [12]. Natural selection initially favored darker skin as a mean to protect against the harmful effects of UV radiation in the tropics. The pressure of selection then shifted toward lighter skin among those humans who spread into higher latitudes with lower levels of UV [13–15]. Melanin, the principal skin pigment that provides this gradient of light to dark color, acts as a photoprotective filter that reduces the penetration of all wavelengths of light into subepidermal tissue. Under the high UVR levels near the equator, dark skin pigmentation is particularly advantageous for protecting folate and reducing the risk of fatal birth defects. Human migration outside the tropics reduced the selective advantage of this photoprotective property and selected instead for lower melanization to facilitate cutaneous vitamin D biosynthesis, critical to the regulation of many biological processes [16]. Although these events are widely accepted as the main evolutionary events generating

human skin pigment variation, this product of natural selection could be currently subjected to other ecological pressures in addition to what caused its origin.

Learning is the psychological mechanism responsible for cultural transmission between conspecifics by promoting different social behaviors that could influence survival and reproduction [17]. Cultural transmission might be adaptive if a transmitted behavior saves learners the costs of individual learning. When this occurs, the selection process will favor improved learning efficiencies and behaviors in the learner that lead to better learning environments. For instance, social learning can influence mate preferences through two different mechanisms [18]; reinforcing a preference for a trait that may signal biological quality if that preference significantly contributes to reproductive success, such as offspring quality, or promoting new preferences for traits that do not appear to have direct links to the bearer's biological quality [19]. In this case, new social preferences may be selected by cultural transmission if the novel trait positively influences social benefits in a particular population [20].

The colonial period in Mexico and all countries of Central and South America led to a highly stratified society based on ethnic origins. During the 300 years that lasted this period, light skin pigmentation, usually present in Spanish colonizers, was one of the most conspicuous phenotypic traits associated with higher social status and hence access to social benefits, such as education and wealth [21]. This racial/color hierarchy system, known as *castes*, placed the European colonizers at the top of the social hierarchy and the indigenous populations at the bottom, it developed deep roots in Latin American society, and even though the *castes* system is officially abolished, there is robust evidence of social stratification based on skin color in the contemporary Mexican society [22]. For example,

recent studies find that people with darker skin color have, on average, lower educational levels, occupational status, and hourly earnings, are more likely to live in poverty, and have fewer chances of social mobility in comparison with people of lighter skin colors; these differences are significant even after controlling for sociodemographic factors such as age, gender, and parental education [22–25]. In addition, a cultural preference for light skin pigmentation and other European phenotypical features are also being influenced by social media in Western societies, which might reinforce the association between skin color and social status. Hence, if light skin pigmentation is culturally preferred because it is associated with social benefits, prestige, or deference, a new skin pigmentation-based signal may have emerged due to an extension of historical, sociocultural learning.

Another non-mutually exclusive hypothesis is that facial skin pigmentation could be driven by perceptions shaped by sexual selection. Human skin pigmentation is a sexually dimorphic trait in which men usually have darker skin pigmentation than women [26–28]. The sex difference is due to exposure of skin tissues to differing ratios of androgens to estrogens, particularly at puberty. Testosterone has a stronger effect than estrogen on melanin synthesis and vascularization of the upper dermis [29–31]. In addition, males undergo a more intense facultative pigmentation after sun exposure and retain it for longer periods than females whereas female skin lightens faster after a reduction in sun exposure [32]. This sexual dimorphism might influence sexual preferences since several studies have shown that darker skin color in men is perceived more attractive [33–36]. Therefore, this female preference for darker skin color could reflect ancestral selection pressures shaping perceptions of an androgen-dependent trait. In this case, the basis of this preference may be the honest signaling of mate quality in terms of male immune function (i.e.,

immunocompetence handicap hypothesis), vigor, or other physiological costs of developing this trait [37–39].

Although the effects of skin pigmentation on social perceptions is an active area of research in social psychology, prior research has focused mainly on racial attitudes and generally does not employ an evolutionary framework. For instance, Hagiwara et al. [40] evaluated affective reactions and other social attributions towards African American men by manipulating facial shape features (nose and lips) while controlling for skin tone. Similar studies have examined the role of skin tone and facial shape features ~~on~~ racial attitudes ~~following~~ similar methods [41,42]. Moreover, most experimental studies of facial perception have been carried out ~~in~~ participants from WEIRD (Western, educated, industrialized, rich, and democratic) societies, limiting the generalizability of findings [43]. ~~Furthermore~~, prior studies ~~carried out~~ in non-WEIRD Latin American samples have implemented a social theoretical framework with a non-experimental design to explore correlations between skin tone and social attributes, such as status or labor hierarchy [44].

In this study, we used an experimental design with a Mexican population to determine whether facial skin color can influence certain social perceptions: attractiveness; attractiveness, trustworthiness, perceived health, dominance, aggressiveness, and femininity/masculinity [45–47]. To that end, we performed a crossover experiment on men's faces, selecting two groups of faces with contrasting innate skin pigmentation: the first composed of faces with natural light facial skin tone, selected from a European American population, ~~while~~ the second ~~group~~ of faces with a darker facial skin tone, selected from a Mexican pre-Columbian population, self-identified as Me'Phaa. Using these two groups, we created six sets of facial stimuli ~~interchanging~~ the natural skin pigmentation

but preserving the facial shape. Finally, we presented all stimuli to be evaluated in the six social dimensions previously mentioned.

We hypothesize that if social learning is acting on the perceptions of facial skin pigmentation, we expect that the lighter skin faces group would be perceived as more attractive, trustworthy, and healthier since positive evaluations of these attributes strongly promote pro-sociality and social benefits but less dominant and aggressive because these perceptions, in contrast, are considered as potentially harmful and may lead to avoidance of social interactions [48]. However, if skin pigmentation more strongly reflects ancestral selection shaping perceptions of an androgen-dependent trait, then we would expect that faces with darker skin color would be perceived not only as more attractive and healthier but also as more dominant, aggressive, and masculine due to the association between these last three attributes to the behavioral and physiological effects of androgens [49,50]. In both cases, if differences in skin pigmentation are the principal facial trait driving these effects, we expect that the experimental manipulation of the facial skin color would change the previous patterns in the perceptions in both the European American and Me´Phaa faces. Finally, if neither light nor dark skin color affects any social perception, both in the original faces and experimentally exchanged stimuli, then skin color would not function in the social selection nor the sexual selection of dimorphic traits.

Material and methods

Participant raters

A total of 489 Mexican adult women (mean age = 25.4 years, SD = 8.6 years) and 211 Mexican adult men (mean age = 26.5 years, SD = 9.1 years) participated as raters in

this study. Self-reported ethnicities were 84.0 % Mestizo, 6.3 % European, 3.9 % Indigenous (particular groups not specified), 0.3 % African, and 6.3 % Other. A total of 485 participants reported living in Mexico City, 95 in the surrounded metropolitan area, and the rest in different states of Mexico. All ~~Participant~~ raters were recruited online ~~by~~ a board advertisement from the Department of Psychology at the National Autonomous University of Mexico.

Skin color facial stimuli

~~To develop the experimental manipulation in facial skin pigmentation, we~~ first created two contrasting light and dark facial skin ~~color~~ prototypes~~. This process was made~~ selecting 30 facial photographs of young European American men and 30 facial photographs of young Me'Phaa Mexican Indigenous men from larger photograph sets. (See below the criteria of selection for both groups of photographs). The European American faces were selected from a set of facial photographs ~~collected in~~ the Department of Anthropology at ~~the~~ Pennsylvania State University [51]. Facial photographs were taken from a distance of 2 m using a 12-megapixel Olympus E-300 camera with a mounted flash at a resolution of 1200 × 1000 pixels. The facial photographs of the Me'Phaa were selected from a collection of facial photographs from the NeuroEcology Lab at the National Autonomous University of Mexico. These facial photographs were taken from a distance of 2m using a 21-megapixel Nikon 5500 camera illuminated by two studio strobes (Lusana Studio, 400w) with reflective umbrellas. The strobes were placed 1.85 m from the target on each side and lights were ~~arrange in~~ 40° angles. In both sets of facial photographs participants were asked to remove any earrings, glasses, or facial jewelry, and to maintain a neutral expression.

The two groups of facial photographs (European American and Me´Phaa) were selected in order to obtain two samples of faces whose facial skin pigmentation was contrasting, one group with lighter skin and color ranges typical for the European American average, and another group with darker skin and color ranges typical for an Indigenous Mexican average. Therefore, in order to evaluate if the facial photographs in both groups met these criteria, we measured its skin color values in CIELab color space. Following procedures reported in previous studies [52,53], we first standardized the facial images by color calibration, then we took color samples of three separate patches located on the forehead and on each cheek and calculated the average CIELab values using Photoshop CS6. Given that lightness (L^*) values are negative correlated with some biochemical measurements of melanin content such as the total melanin content of the epidermis and vary with ethnicity [54], we focused on this parameter to evaluate skin color differences between the two selected groups. European American (L^* mean = 61.08, SD = 6.42) and Me'Phaa (L^* mean = 49.84, SD = 4.05) lightness mean values were statistically different (t (58) = -8.07, $p < 0.01$), and lightness values for both groups were in the ranges for White/European and Hispanic ethnicities reported in previous studies [55,56].

From the 60 facial photographs selected, we created two composite faces that averaged the skin color of the 30 original faces from each group using the software Psychomorph [57]. The averaged composite face of the European American men was used as the light skin color prototype, while the composite face of the Me'Phaa men was the dark skin color prototype. Next, we randomly selected eight faces from the European American men's set and eight from the Me'Phaa men's set and created two different versions of each face, one displaying light skin color and the one displaying dark skin

~~color~~. These two versions were created by adding or subtracting 70% of the skin color differences between the light and dark facial skin ~~color~~ prototypes previously created ~~but~~ leaving facial shape unchanged. Finally, we repeated the skin color measurement process (see above) in both groups of the new composite faces (light skin ~~color~~ transformed and dark skin ~~color~~ transformed faces). The average lightness value was L^* = 66.74, SD = 1.37 in the light skin ~~color~~ transformed versions and L^* = 52.75, SD = 2.58 in the dark skin ~~color~~ transformed versions. ~~We observed the method we used for~~ skin color manipulation slightly increased the lightness values ~~compared~~ to the values ~~we observed in~~ the natural faces, ~~however these differences~~ were not statistically significant for any group (t (44) = -0.52, $p = 0.6$; t (44) = -0.43, $p = 0.6$), and both groups stayed within the ranges reported for White/European and Hispanic ethnicities in previous studies [55,56]. At the end of this process, we obtained three versions for each of the eight European American and ~~the~~ eight Me'Phaa faces: a natural unmodified version (NAT), a light skin ~~color~~ transformed version (LSCt), and dark skin ~~color~~ transformed version (DSCt; Fig. 1), ~~resulting in~~ a total of 48 facial stimuli.

Fig. 1. Example stimuli. Three versions of the same facial stimuli of a European American male (up) and a Me'Phaa male (bottom). Natural unmodified versions (left), Light Skin ~~Color~~ transformed versions (middle), and Dark Skin ~~Color~~ transformed versions (right).

Facial perception rates

To obtain the ratings, we divided the 48 stimuli into eight sets of six stimuli each. The stimuli were assigned ~~into every set counterbalancing by~~ group (European American and Me'Phaa) and version (NAT, LSCt, and DSCt) and we made sure that two or more versions of the same face were not included together in the same set. Given that the

experimental transformation ~~resulted in the homogenization of~~ facial skin color and texture, two features that affect perceptions of human faces [58,59], we decided to include the natural version of each face (NAT) to be able to compare the ratings of the natural versions with the ratings of the concordant skin color transformed version~~, that is compare~~ the Natural versions with the Light skin ~~color~~ transformed versions in the European American faces and the Natural versions with the dark skin ~~color~~ transformed versions in the Me'Phaa faces. By doing this we could measure the effect of the color and texture homogenization.

The facial evaluations were performed online at the webpage labneuroecologiacog.com. Each participant was randomly assigned to one of the eight facial stimuli sets and was asked to rate each face separately on a 9-point Likert scale according to the following perceptions~~;~~ attractiveness (1 = not attractive at all, 9 = very attractive)~~,~~ trustworthiness (1= not trustworthy at all, 9= very trustworthy)~~,~~ perceived health (1= not healthy at all, 9= very healthy)~~,~~ dominance (1 = not dominant at all, 9 = very dominant)~~,~~ aggressiveness (1= not aggressive at all, 9= very aggressive) and femininity/masculinity (1= very ~~femininity~~/not masculine at all, 9= very masculine/ not ~~femininity~~ at all) . For all trials, we randomized both the order of facial stimuli and the order of the ~~perception asked~~. In addition, to standardize the response ~~evoked~~ for attractiveness in men and women, we asked the men participants, *"How attractive does this face look for a woman?"* (For similar methods see [60]). Finally, raters completed a socio-demographic survey and were asked about their sexual orientation, ~~implementing~~ a 5-point Likert scale, from entirely same-gender to entirely opposite-gender orientation. As a result, 610 ~~Participant~~ raters (437 women and 173 men) identified themselves as opposite-gender oriented/heterosexual, 42 (15 women and 27 men) identified as same-gender sexual orientation, 35 (28 women and 7 men) identified as bisexual, and 13 (9 women and 4 men)

reported not having any sexual orientation. We controlled the sexual orientation information in the statistical analysis (see below). In addition, self-perceived skin color may have been influencing the rater's responses [23,61]. Hence, ~~participates~~ were asked to rate their own skin color using the PERLA color palette. The PERLA color palette is a scale of 11 skin tones (1 = lightest, 11 = darkest) that was made ~~as an effort to have~~ an easy-to-use ~~tool that summarize~~ the typical skin tones found in regions of Latin America [62]. Similar to previous studies ~~conducted~~ on the Mexican population [24], we found that 77. 6% of ~~participants~~ responses were concentrated in the middle levels of the scale (4-6), while the rest ~~of participants~~ were ~~distributed through~~ the extremes~~;~~ 12.7 % in the lightest skin tones ~~levels~~ (1-3) and 9.7% the darkest ~~levels~~ (7-10; see S1 Fig.). Finally, all raters signed an informed consent form before evaluating facial stimuli, and all procedures were approved by the appropriate National Autonomous University of Mexico ethics committee (FPSI/CE/01/2016) and are in compliance with Mexican legislation and the Code of Ethical Principles for Medical Research Involving Human Subjects of the World Medical Association (Declaration of Helsinki).

Statistical analysis

To determine whether the experimental manipulation ~~in~~ facial skin color modified the ~~perception of social judgments~~, we fitted generalized linear mixed models (GLMM) ~~of the Poisson Family~~ with the log link function. We decided to fit Poisson GLMMs because, unlike other studies where the ratings for each facial stimulus are averaged, here all dependent variables come from discrete raw ratings ~~data~~. Moreover, during the data

exploration we observed a positively skewed counts-type distribution with ~~the larger number of~~ observations at the lower values ~~in~~ some of the facial perceptions (i.e., attractiveness perception). We ~~performed~~ one ~~global~~ model per each perception evaluated. ~~Global~~ models included as the dependent variable any of the following facial perceptions: attractiveness, trustworthiness, perceived health, dominance, aggressiveness or femininity/masculinity. Fixed predictors for all ~~global~~ models included the ethnic group of each evaluated face (i.e., European American or Me'phaa faces), the ~~skin color faces-~~ natural and transformed faces (i.e., NAT, LSCt or DSCt)~~-~~ sex of ~~participant~~ raters, and the interaction between ethnic group and skin color transformed faces. To account for the potential non-independence of data, we included a random intercept for ~~participant~~ ID, a random intercept for facial photograph ID (the individual code of each original face used), and a random slope for skin color transformation on facial photograph ID.

To evaluate the effect of ~~participant~~ sexual preferences, we ~~performed~~ models ~~both including~~ responses from all participants and responses from heterosexual participants only. Next, each ~~global~~ model was then reduced to obtain a minimum adequate model using the Akaike Information Criterion (AIC). We selected the models with the lowest AIC values and a ΔAIC higher than two units from the second most supported model [63]. When the difference between the best-supported model and the subsequent model was $\Delta<2$, we followed the statistical principle of parsimony [64], and hence we selected the minimum adequate model as the one with the lowest AIC and the least number of parameters (Table 1). Finally, once we obtained minimum adequate models, model diagnostics were performed, ~~checking~~ residual distribution and linearity of residuals in each model. In order to check for any effect of other factors not included in the models [65,66], we plotted

Pearson residuals of each selected model against participant´s self-reported ethnicities and the PERLA scale responses for self-perceived skin color. Since the plots did not show any clear effect, we did not include any of these two factors as fixed predictors. All analyses were performed using the *lme4* [67] and *bbmle* [68] packages in R, version 4.0.2 [69].

Table 1. Information criteria for the best ~~fitted~~ models.

	df	$AICc$	$\Delta AICc$	$w_i(AICc)$
(a) Perceived attractiveness				
**Group + Color + Participant sex + Group:Color**	**14**	**14331**	**0.000**	**0.999**
Group + Color + Participant sex	12	14346	15.053	0.001
Group + Color + Group:Color	13	14348	16.957	0.000
(b) Perceived trustworthiness				
**Group + Color**	**11**	**15167**	**0.000**	**0.506**
Group + Color + Participant sex	12	15169	1.778	0.208
Group + Color + Group: Color	13	15170	2.596	0.138
(c) Perceived health				
**Group + Color + Group:Color**	**13**	**15561**	**0.000**	**0.707**
Group + Color + Participant sex + Group:Color	14	15563	1.892	0.274
Group + Color	11	15570	9.186	0.007
(d) Perceived dominance				
**Group + Participant sex**	**10**	**15485**	**0.000**	**0.493**
Group + Color	11	15488	2.685	0.129
Group + Color + Group: Color	13	15489	3.137	0.103
(e) Perceived aggressiveness				
**Group + Color + Group:Color**	**13**	**16625**	**0.000**	**0.663**
Group + Color + Participant sex + Group:Color	14	16626	1.768	0.274
Group + Color	11	16630	5.479	0.043
(f) Perceived masculinity				
**Group + Color + Group:Color**	14	15486	0.000	0.482
Group + Color + Participant sex + Group:Color	**13**	**15486**	**0.035**	**0.474**
Group + Color	11	15492	6.367	0.020

Information criteria and fixed factors included for the three best ~~fitted~~ models for: (a) Perceived attractiveness, (b) Perceived trustworthiness, (c) Perceived health, (d) Perceived dominance, (e) Perceived aggressiveness, and (f) Perceived masculinity.

Models are in descending order. ΔAIC is the change in AIC between each model and the best model. Akaike weights are conditional probabilities for each model being the best model. Models in bold are the selected ones.

Results

Perceived attractiveness

Perceived attractiveness differed significantly between the two groups when they had their natural skin color ($z = 6.51$, $p < 0.01$). The European American faces were rated 1.70 times more attractive ($M = 4.30$, 95% CI [3.83 - 4.84]) than the Me'Phaa faces ($M = 2.50$, 95% CI [2.21 - 2.82], Table 2). Furthermore, there were effects when natural skin color was changed within each of the two groups. Lightening the Me'Phaa faces made them more attractive ($M = 3.75$, 95% CI [3.42 - 4.11], $z = 7.42$, $p < 0.01$), and darkening the European American faces made them less attractive ($M = 3.30$, 95% CI [3.02 - 3.60], $z = -6.25$, $p < 0.01$). In addition, we observed an effect of skin color and texture homogenization due to experimental manipulation. Both groups became slightly more attractive when skin color and skin texture were homogenized (~~For~~ Me'Phaa faces: $M = 2.94$, 95% CI [2.68 - 3.21]; ~~For~~ European American faces: $M = 4.77$, 95% CI [4.36 - 5.22]). However, this effect was significant for the Me'Phaa faces ($z = -3.23$, $p < 0.01$) but not for the European American faces ($z = -2.00$, $p = 0.34$; Fig. 2). Finally, we observed a significant main effect of ~~participant~~ sex ($z = 4.36$, $p<0.01$); ~~the men participants rated all faces as more attractive~~ ($M = 3.76$, 95% CI [3.48 - 4.06]) than ~~did the female participants~~ ($M = 3.27$, CI 95% [3.06 - 3.49]). The direction of the effects and statistical significance were not affected when including the sexual preference ~~of participants~~ (S1 Table.).

Table 2. Perception estimates with 95% confidence intervals.

	Me'Phaa			European American		
	NAT	LSCt	DSCt	NAT	LSCt	DSCt
	Est. [95%CI]	*Est. [95%CI]*	*Est. [95%CI]*	*Est. [95%CI]*	*Est. [95%CI]*	*Est. [95%CI]*
a) Perceived attractiveness	2.50 [2.21-2.82]	3.75 [3.42-4.11]	2.94 [2.68-3.21]	4.30 [3.83-4.84]	4.77 [4.36 5.22]	3.30 [3.02-3.60]
b) Perceived trustworthiness	4.21 [4.03-4.40]	4.48 [4.30-4.67]	4.33 [4.16-4.51]	4.75 [4.55-4.96]	5.06 [4.86-5.27]	4.89 [4.70-5.08]
c) Perceived health	4.28 [3.83-4.79]	5.51 [5.19-5.84]	5.21 [4.85-5.60]	5.10 [4.56-5.69]	5.96 [5.62-6.31]	5.05 [4.70-5.43]
d) Perceived dominance	5.23 [4.85-5.64]	□	□	4.31 [3.99-4.66]	□	□
e) Perceived aggressiveness	5.43 [5.17-5.69]	5.05 [4.85-5.26]	5.39 [5.17-5.62]	5.10 [4.85-5.35]	4.79 [4.59-4.99]	4.56 [4.37-4.77]
f) Perceived masculinity	6.18 [5.79-6.60]	5.15 [4.83-5.49]	5.97 [5.59-6.37]	5.14 [4.80-5.50]	4.83 [4.53-5.15]	4.93 [4.61-5.27]

Estimates (Est.) with 95% confidence intervals (CI) of the fixed predictors of the models for: (a) Perceived attractiveness, (b) Perceived trustworthiness, (c) Perceived health, (d) Perceived dominance, (e) Perceived aggressiveness, and (f) Perceived masculinity.
Note: (□*)* The Perceived dominance best ~~fitted~~ model did not include the skin color transformation predictor, therefore the estimates shown represent the main effect of the natural skin color group only.

Fig. 2. Perceived attractiveness estimates for the Me'Phaa and European American faces within each color version. Estimates for the Natural versions (NAT), Light skin ~~color~~ transformed versions (LSCt), and Dark skin ~~color~~ transformed versions (DSCt) of the Me'Phaa (left) and European American (Right) faces. Error bars show 95% Confidence Intervals. Grey points and dashed lines indicate the random effect estimates for each Face ID within each color version.

Perceived trustworthiness

Natural European American faces were rated 1.13 times more trustworthy (*M* = 4.75, 95% CI [4.55 - 4.96], *z* = 5.19, *p* < 0.01) than natural Me'Phaa faces (*M* = 4.21, 95% CI [4.03 - 4.40], Table 2). In addition, lightening the Me'Phaa faces increased their perceived trustworthiness (*M* = 4.48, 95% CI [4.30 - 4.67], *z* = 3.26, *p* < 0.01) whereas darkening the European American faces had no effect (*M* = 4.89, 95% CI [4.70 - 5.08], *z* = 1.44, *p* = 0.15). ~~Moreover, there was no effect of skin~~ color and texture homogenization ~~due to experimental manipulation~~ for the Me'Phaa faces (DSCt Me'Phaa faces M = 4.89, 95% CI [4.70 - 5.08], *z* = -1.43, *p* = 0.70), but ~~this increased~~ scores for European American faces (LSCt European American faces *M* = 5.06, 95% CI [4.86 - 5.27], *z* = -3.26, *p* = 0.01; Fig. 3). Finally, there was no effect of ~~participant~~ sex, as this predictor was not present in the minimum adequate model selected by AIC (Table 1). The direction of the effects and statistical significance were not affected when the sexual preference ~~of participants~~ was included (S1 Table.).

Fig. 3. Perceived trustworthiness estimates for Me'Phaa and European American faces within each color version. Estimates for the Natural versions (NAT), Light skin ~~color~~ transformed versions (LSCt), and Dark skin ~~color~~ transformed versions (DSCt) of the Me'Phaa (left) and European American (Right) faces. Error bars show 95% Confidence Intervals. Grey points and dashed lines indicate the random effect estimates for each Face ID within each color version.

Perceived health

As with perceived attractiveness and trustworthiness, we found a significant difference in the perceived health scores between the natural Me'Phaa and European American faces (z = 2.20, p = 0.02). On average, natural European American faces were perceived as being 1.19 times healthier (M = 5.10, 95% CI [4.56 - 5.69]) than natural Me'Phaa faces (M =4.28, 95% CI [3.83 - 4.79], Table 2). When facial skin color was changed within each ethnic group, the changes had different effects on perceptions of the two groups. Lightening the Me'Phaa faces increased their perceived health (M = 5.51, 95% CI [5.19 - 5.84], z = 4.96, p < 0.01), whereas darkening the European American faces had no effect (M = 5.05, 95% CI [4.70 - 5.43], z = 0.20, p = 0.99). Perceived health increased slightly for both groups when skin color and skin texture were homogenized (~~For~~ European Americans: M = 5.96 (95% CI [5.62 – 6.31], z = -3.12, p = 0.02; ~~For~~ Me'phaa people: M = 5.21, 95% CI [4.85 - 5.60], z = 4.38, p < 0.01; Fig. 4). Finally, there was no effect of ~~participant~~ sex, as this predictor was not present in the minimum adequate model selected by AIC (Table 1). The direction of the effects and statistical significance were not affected when the sexual preference ~~of participants~~ was included (S1 Table.).

Fig. 4. Perceived health estimates for Me'Phaa and European American faces within each color version. Estimates for the Natural versions (NAT), Light skin ~~color~~ transformed versions (LSCt), and Dark skin ~~color~~ transformed versions (DSCt) of the Me'Phaa (left) and European American (Right) faces. Error bars show 95% Confidence Intervals. Grey points and dashed lines indicate the random effect estimates for each Face ID within each color version.

Perceived dominance

Unlike the previous qualities, perceived dominance was lower for natural European American faces (*M* = 4.31, 95% CI [3.99 - 4.66], $z = -3.63$, $p < 0.01$) than Me'Phaa faces (*M* = 5.23, 95% CI [4.85 - 5.64], Table 2). Moreover, in contrast to perceived attractiveness, all faces received a lower dominance rating from male ~~participants~~ (*M* = 4.63, 95% CI [4.34 - 4.93], $z = -2.24$, $p = 0.02$) than from female ~~participants~~ (*M* = 4.88, 95% CI [4.61 - 5.16]). Finally, we were unable to determine any effect from lightening, darkening, or homogenizing the faces, since these predictors were not present in the minimum adequate model selected by AIC (Table 1). Statistical significance of these results did not change in the model that included all ~~participants~~ (S1 Table.).

Perceived aggressiveness

Although perceived aggressiveness was lower for the European American faces, the difference was not significant ($z = -1.83$, $p = 0.06$). Nevertheless, lightening the Me'Phaa faces made them seem less aggressive (*M* = 5.05, 95% CI [4.85 - 5.26], $z = -2.76$, $p < 0.01$) but darkening the European American faces also made them seem less aggressive (*M* = 4.56, 95% CI [4.37 - 4.77], $z = 4.17$, $p < 0.01$, Table 2). Finally, perceived aggressiveness did not change for either group when skin color and skin texture were homogenized (Me'Phaa faces *M* = 5.39, 95% CI [5.17 - 5.62]; $z = -0.23$, $p = 0.82$; European American faces *M* = 4.79, 95% CI [4.59 - 4.99]; $z = 2.35$, $p = 0.17$, Fig. 5.), and there was no effect of ~~participant~~ sex, as this predictor was not present in the minimum adequate model selected by AIC (Table 1). Finally, the direction of the effects and statistical significance were not affected when the sexual preference ~~of participants~~ was included (S1 Table.).

Fig. 5. Perceived aggressiveness estimates for Me'Phaa and European American faces within each color version. Estimates for the Natural versions (NAT), Light skin ~~color~~ transformed versions (LSCt), and Dark skin ~~color~~ transformed versions (DSCt) of the Me'Phaa (left) and European American (Right) faces. Error bars show 95% Confidence Intervals. Grey points and dashed lines indicate the random effect estimates for each Face ID within each color version.

Perceived masculinity

As with perceived dominance, perceived masculinity was lower for natural European American faces (*M* = 5.14, 95% CI [4.80 - 5.50], *z* = -3.95, *p* < 0.01 than for natural Me'Phaa faces (*M* = 6.18, 95% CI [5.79 - 6.60], Table 2). Moreover, lightening the Me'Phaa faces made them seem less masculine (*M* = 5.97, 95% CI [5.59 - 6.37], *z* = -5.59, *p* < 0.01), but darkening the European American faces had no effect (*M* = 4.93, 95% CI [4.61 - 5.27], *z* = 1.20, *p* = 0.83). In addition, perceived masculinity did not change for either group when skin color and skin texture were homogenized (DSCt Me'Phaa faces *M* = 5.97, 95% CI [5.59 - 6.37]; *z* = 1.05, *p* = 0.90; LSCt European American faces *M* = 4.83, 95% CI [4.53 - 5.15]; *z* = 1.82, *p* = 0.45, Fig. 6). Finally, there was no effect of ~~participant~~ sex, as this predictor was not present in the minimum adequate model selected by AIC (Table 1). The direction of the effects and statistical significance was not affected when the sexual preference ~~of participants~~ was included (S1 Table.).

Fig. 6. Perceived masculinity estimates for the Me'Phaa and European American faces within each color version. Estimates for the Natural versions (NAT), Light skin ~~color~~ transformed versions (LSCt), and Dark skin ~~color~~ transformed versions (DSCt) of the Me'Phaa (left) and European American (Right) faces. Error bars show 95% Confidence

Intervals. Grey points and dashed lines indicate the random effect estimates for each Face ID within each color version.

Discussion

Our findings suggest that facial skin pigmentation influences social judgments in a Mexican population. Based on our results, we could distinguish two groupings of social perceptions that facial skin pigmentation affects in opposite directions. In comparison with natural Me'Phaa faces, natural European American faces were perceived not only as more attractive, healthier, and more trustworthy but also as less dominant and less masculine. They were furthermore perceived as less aggressive, but not significantly so. Moreover, the perceptions grouped in these two dimensions correlate more within than between dimensions (S2 Table.). The apparent grouping of social perceptions found here resembles the valence-dominance model of facial perception proposed by Oosterhof and Todorov [70] and recently discussed by Jones et al. [6]. The valence-dominance model suggests that humans make impressions of others based on their facial appearance in the dimensions of valence and dominance. The valence dimension is related to facial judgments such as trustworthiness and attractiveness; hence, higher rates of this dimension could promote positive social interactions, for example mate opportunities. The dominance dimension, in turn, is related to perceptions such as aggressiveness, dominance, and masculinity, which could inform about the physical or psychological capability to cause harm; hence, higher values of this dimension could negatively affect social interactions in a modern context [8]. Here, we found that Mexican participants rated light-skinned faces more highly on all three

aspects of social valence (attractiveness, healthiness, trustworthiness). In contrast, ~~they rated~~ dark-skinned faces more highly on two aspects of social dominance (dominance, masculinity).

In the context of our first hypothesis, these results support the idea that sociocultural learning favors the function of facial skin pigmentation as a mediator of social perceptions. Our Mexican participants showed a robust positive association of pro-social judgments (i.e., attractiveness, trustworthiness, and perceived health) with light skin pigmentation. During the Mexican colonial period, light skin pigmentation was one of the most conspicuous phenotypic traits associated with a higher social status and access to different benefits, such as education and wealth [21]. Learning can reinforce a preference for a trait that may signal social benefits, even when the selected trait is not linked initially to any reproductive or survival benefit [19]. Consequently, a learned association between a physical trait—in this case, light skin—and social deference might establish a perception in a population over several generations. Furthermore, several studies have shown cultural preferences for light skin pigmentation and other European phenotypical traits and within-group discrimination based on skin color, a social phenomenon known as colorism [71]. Also, in Mexico and other Latin American countries, lightness of skin correlates with years of schooling, hourly earnings, occupational status, poverty, and social mobility [23–25]. Therefore, the association of pro-social attributes with light skin pigmentation we observed may be the result of a preference that emerged as an adaptative strategy to deal with biological and social pressures during the colonial period, which nowadays is reinforced by social learning and cultural mechanisms such as colorism, or the influence of Western societies on social media.

We also found that ~~our participants rated~~ the dark-skinned faces as more dominant and masculine. However, in contrast to our second prediction, ~~dark skin color faces~~ were not rated as more attractive or healthy. These results could indicate that skin pigmentation, as a trait mediated by androgens, could ~~act as a mechanism to~~ inform the viewer about the physical or psychological capability to cause harm by the bearer of the signal [72,73]; therefore, this signaling function might be relevant in sexual selection through male-male competition instead of female choice[74]. Similar results have been found in previous studies where androgen-dependent sexually dimorphic traits such as facial shape [75], voice pitch [76,77], or height [78], more strongly affect the perception of dominance, aggressiveness, or masculinity rather than attractiveness.

~~On the other hand, our experimental~~ facial color ~~interchange over~~ 16 different faces ~~allowed us to~~ determine whether additional facial traits, such as shape, influence social perceptions in combination with skin color or even more than skin color. The human face embodies a ~~set of a~~ variety of signals and cues [79], and even though humans process faces in an integrative manner, this process follows a hierarchy of relevance in which different facial features are more important than others, depending on the perceptions evaluated [80,81]. In this way we found that some social perceptions were more affected by skin color than others. For example, lightening the Me'Phaa faces made them seem more attractive, healthier, and more trustworthy. Conversely, darkening the European American faces made them seem less attractive. Other perceived qualities, however, did not change systematically when facial skin color was changed. Perceived dominance ~~did not change when~~ changes ~~were made~~ to facial skin color within either group. Lightening the Me'Phaa faces decreased their perceived masculinity and aggressiveness, but darkening the

European American faces did not increase their perceived masculinity and aggressiveness. These results are consistent with studies on the role of skin color on certain perceptions, such as attractiveness [59,82], which show that skin color might contribute more than facial shape. Moreover, recent studies have shown that trustworthiness judgments rely on emotional expressions [8] or facial resemblance [83], and that dominance, masculine, and aggressiveness perception seem to rely more strongly on facial shape features [84–88]. Yet another possibility is that the skin color and facial shape effects on some of these perceptions interact in such a way that skin color becomes relevant only when the facial shape strongly elicits one of these perceptions. This may explain why the modification of skin color reduced the masculine and aggressiveness perception for the Me´Phaa faces, which were initially rated as more masculine and dominant, but it had no effect on the European American faces.

Another possibility is that there were systematic differences in the way that the experimental manipulation of skin color affected the European American and the Me´Phaa faces. In the statistical analysis we fitted random slopes for skin color transformation on each facial photograph ID to reduce the standard error caused by these differences, however, it is important to note that any manipulation process presents a risk of such artifacts. On the other hand, previous studies have shown that individuals are better at judging faces of their own ethnicity [35,89]. In this study our Mexican participants were asked to rate facial stimuli of a more closely related ethnic group (Me´Phaa faces), and stimuli from a more distant ethnic group (European American faces). Although 83% of our raters reported living in Mexico City and the surrounded area, hence the high probabilities of being exposed to European facial features due to immigration of people of European

ethnicity and access to internet and social media [90,91], these differences on the effects of the skin color manipulation between the European American and the Me´Phaa faces might be due to different degrees of familiarity with the facial features of the two ethnic groups.

Moreover, we not only lightened and darkened facial color but also homogenized it by removing such texture-related aspects as spots, freckles, or wrinkles [59,92,93]. Our results suggest that skin texture does have an effect, particularly on perceived health. That effect, however, largely exists independently of the effects of skin color. Skin texture might nonetheless interact with skin color in perceptions of light and dark skin, and such interactions could be an exciting avenue for further studies. In addition, unlike the authors of previous studies, we manipulated overall skin color instead of gradually changing each of its aspects (e.g., luminosity, hue) [33,35,46,53,60,82,94–97]. In our study, we aimed to test the differences in skin colors typically related to two different ethnic groups using a broader manipulation of skin color. Nonetheless, future studies could examine how each aspect of skin color contributes to the way a population is perceived.

In summary, we show that the Mexican population uses facial color to make certain social judgments. We found that a dark-skinned face is perceived as more dominant and more masculine. Those perceptions may be due to a process of coevolution between the human mind and the sex difference in skin color. One may therefore infer that male skin became darker through male-male competition rather than through female choice. We also found that a light-skinned face is perceived as more attractive, more trustworthy, and healthier. Those perceptions may be due to the association between skin color and social status that prevailed in Mexico during more than three centuries of colonial rule. Today, they are maintained by colorism. We are nonetheless speculating, and more data will be

needed. Similar results could be obtained from other Latin American societies, which share a common colonial history and social structure.

Acknowledgements

The authors are grateful to Juan Sebastían Lucero Carrasquilla for his help and assistance during the facial photograph acquisition of the Me'Phaa men sample.

References

1. Little AC, Jones BC, Debruine LM. Facial attractiveness: Evolutionary based research. Philosophical Transactions of the Royal Society B: Biological Sciences. 2011;366: 1638–1659. doi:10.1098/rstb.2010.0404
2. Puts DA, Jones BC, Debruine LM. Sexual selection on human faces and voices. J Sex Res. 2012;49: 227–243. doi:10.1080/00224499.2012.658924
3. Gonzalez-Santoyo I, Wheatley JR, Welling LLM, Cárdenas RA, Jimenez-Trejo F, Dawood K, et al. The face of female dominance: Women with dominant faces have lower cortisol. Horm Behav. 2015;71: 16–21. doi:10.1016/j.yhbeh.2015.03.006
4. Puts DA, Bailey DH, Cárdenas RA, Burriss RP, Welling LLM, Wheatley JR, et al. Women's attractiveness changes with estradiol and progesterone across the ovulatory cycle. Horm Behav. 2013;63: 13–19. doi:10.1016/j.yhbeh.2012.11.007
5. Ritchie KL, Palermo R, Rhodes G. Forming impressions of facial attractiveness is mandatory. Sci Rep. 2017;7: 1–8. doi:10.1038/s41598-017-00526-9
6. Jones BC, DeBruine LM, Flake JK, Liuzza MT, Antfolk J, Arinze NC, et al. To which world regions does the valence–dominance model of social perception apply? Nat Hum Behav. 2021;5: 159–169. doi:10.1038/s41562-020-01007-2
7. Olivola CY, Funk F, Todorov A. Social attributions from faces bias human choices. Trends Cogn Sci. 2014;18: 566–570. doi:10.1016/j.tics.2014.09.007
8. Todorov A, Olivola CY, Dotsch R, Mende-Siedlecki P. Social attributions from faces: Determinants, consequences, accuracy, and functional significance. Annu Rev Psychol. 2015;66: 519–545. doi:10.1146/annurev-psych-113011-143831

9. Barsh GS. What controls variation in human skin color? PLoS Biol. 2003;1: 19–23. doi:10.1371/journal.pbio.0000027

10. Samson N, Fink B, Matts PJ. Visible skin condition and perception of human facial appearance. Int J Cosmet Sci. 2010;32: 167–184. doi:10.1111/j.1468-2494.2009.00535.x

11. Edwards EA, Duntley SQ. THE PIGMENTS AND COLOR OF LIVING HUMAN SKIN. American Journal of Anathmy. 1939;65.

12. Darwin C. The Descent of Man, and Selection in Relation to Sex. J. Murray. London; 1871.

13. Jablonski NG, Chaplin G. The evolution of human skin coloration. J Hum Evol. 2000;39: 57–106. doi:10.1006/jhev.2000.0403

14. Adhikari K, Mendoza-Revilla J, Sohail A, Fuentes-Guajardo M, Lampert J, Chacón-Duque JC, et al. A GWAS in Latin Americans highlights the convergent evolution of lighter skin pigmentation in Eurasia. Nat Commun. 2019;10. doi:10.1038/s41467-018-08147-0

15. Deng L, Xu S. Adaptation of human skin color in various populations. Hereditas. 2018;155: 1. doi:10.1186/s41065-017-0036-2

16. Jablonski NG, Chaplin G. Human skin pigmentation as an adaptation to UV radiation. Proc Natl Acad Sci U S A. 2010;107: 8962–8968. doi:10.1073/pnas.0914628107

17. Feldman MW, Cavalli-Sforza LL. Cultural and biological evolutionary processes: Gene-culture disequilibrium. Isotopenpraxis. 1984;20: 1604–1607. doi:10.1073/pnas.81.5.1604

18. Verzijden MN, ten Cate C, Servedio MR, Kozak GM, Boughman JW, Svensson EI. The impact of learning on sexual selection and speciation. Trends Ecol Evol. 2012;27: 511–519. doi:10.1016/j.tree.2012.05.007

19. Little AC, Jones BC, Debruine LM, Caldwell CA. Social learning and human mate preferences: A potential mechanism for generating and maintaining between-population diversity in attraction. Philosophical Transactions of the Royal Society B: Biological Sciences. 2011;366: 366–375. doi:10.1098/rstb.2010.0192

20. Rendell L, Fogarty L, Hoppitt WJE, Morgan TJH, Webster MM, Laland KN. Cognitive culture: Theoretical and empirical insights into social learning strategies. Trends Cogn Sci. 2011;15: 68–76. doi:10.1016/j.tics.2010.12.002

21. Dixon AR, Telles EE. Skin color and colorism: Global research, concepts, and measurement. Annu Rev Sociol. 2017;43: 405–424. doi:10.1146/annurev-soc-060116-053315

22. Villarreal A. Stratification by skin color in contemporary Mexico. Am Sociol Rev. 2010;75: 652–678. doi:10.1177/0003122410378232

23. Telles E, Flores RD, Urrea-Giraldo F. Pigmentocracies: Educational inequality, skin color and census ethnoracial identification in eight Latin American countries. Res Soc Stratif Mobil. 2015;40: 39–58. doi:10.1016/j.rssm.2015.02.002

24. Campos-Vazquez RM, Medina-Cortina EM. Skin Color and Social Mobility: Evidence From Mexico. Demography. 2019;56: 321–343. doi:10.1007/s13524-018-0734-z

25. Flores R, Telles E. Social Stratification in Mexico: Disentangling Color, Ethnicity, and Class. Am Sociol Rev. 2012;77: 486–494. doi:10.1177/0003122412444720

26. van den Berghe PL, Frost P. Skin color preference, sexual dimorphism and sexual selection: A case of gene culture co-evolution? Ethn Racial Stud. 1986;9: 87–113. doi:10.1080/01419870.1986.9993516

27. Madrigal L, Kelly W. Human skin-color sexual dimorphism: A test of the sexual selection hypothesis. reply to frost (2007). Am J Phys Anthropol. 2007;133: 780–781. doi:10.1002/ajpa.20582

28. Aoki K. Sexual selection as a cause of human skin colour variation: Darwin's hypothesis revisited. Ann Hum Biol. 2002;29: 589–608. doi:10.1080/03014460210000019144

29. Giacomoni PU, Mammone T, Teri M. Gender-linked differences in human skin. J Dermatol Sci. 2009;55: 144–149. doi:10.1016/j.jdermsci.2009.06.001

30. Edwards EA, Hamilton JB, Duntley SQ, Hubert G. CUTANEOUS VASCULAR AND PIGMENTARY CHANGES IN CASTRATE AND EUNUCHOID MEN. Endocrinology. 1941;28: 119–128.

31. Manning JT, Bundred PE, Mather FM. Second to fourth digit ratio, sexual selection, and skin colour. Evolution and Human Behavior. 2004;25: 38–50. doi:10.1016/S1090-5138(03)00082-5

32. Harvey RG. Ecological factors in skin color variation among Papua New Guineans. Am J Phys Anthropol. 1985;66: 407–416. doi:10.1002/ajpa.1330660409

33. Carrito M de L, Santos IMB dos, Lefevre CE, Whitehead RD, Silva CF da, Perrett DI. The role of sexually dimorphic skin colour and shape in attractiveness of male faces. Evolution and Human Behavior. 2016;37: 125–133. doi:10.1016/j.evolhumbehav.2015.09.006

34. Stephen ID, Coetzee V, Perrett DI. Carotenoid and melanin pigment coloration affect perceived human health. Evolution and Human Behavior. 2011;32: 216–227. doi:10.1016/j.evolhumbehav.2010.09.003

35. Coetzee V, Greeff JM, Stephen ID, Perrett DI. Cross-cultural agreement in facial attractiveness preferences: The role of ethnicity and gender. PLoS One. 2014;9. doi:10.1371/journal.pone.0099629

36. Frost P. Different Phases of the Menstrual Cycle : Preliminary assesment of evidence for a hormonal relationship. Percept Mot Skills. 1994;79: 507–514.

37. Folstad I, Karter AJ. Parasites, bright males, and the immunocompetence handicap. American Naturalist. 1992;139: 603–622. doi:10.1086/285346

38. Rantala MJ, Moore FR, Skrinda I, Krama T, Kivleniece I, Kecko S, et al. Evidence for the stress-linked immunocompetence handicap hypothesis in humans. Nat Commun. 2012;3. doi:10.1038/ncomms1696

39. Marcinkowska UM, Rantala MJ, Lee AJ, Kozlov M V., Aavik T, Cai H, et al. Women's preferences for men's facial masculinity are strongest under favorable ecological conditions. Sci Rep. 2019;9: 1–10. doi:10.1038/s41598-019-39350-8

40. Hagiwara N, Kashy DA, Cesario J. The independent effects of skin tone and facial features on Whites' affective reactions to Blacks. J Exp Soc Psychol. 2012;48: 892–898. doi:10.1016/j.jesp.2012.02.001

41. Stepanova E V., Strube MJ. What's in a face? the role of skin tone, facial physiognomy, and color presentation mode of facial primes in affective priming effects. Journal of Social Psychology. 2012;152: 212–227. doi:10.1080/00224545.2011.597797

42. Dunham Y, Stepanova E V., Dotsch R, Todorov A. The development of race-based perceptual categorization skin color dominates early category judgments. Dev Sci. 2015;18: 469–483. doi:10.1111/desc.12228

43. Jack RE, Crivelli C, Wheatley T. Data-Driven Methods to Diversify Knowledge of Human Psychology. Trends Cogn Sci. 2018;22: 1–5. doi:10.1016/j.tics.2017.10.002

44. Torres F, Salgado M, Mackenna B, Núñez J. Who differentiates by skin color? Status attributions and skin pigmentation in Chile. Front Psychol. 2019;10: 1–12. doi:10.3389/fpsyg.2019.01516

45. Oosterhof NN, Todorov A. The functional basis of face evaluation. Proc Natl Acad Sci U S A. 2008;105: 11087–11092. doi:10.1073/pnas.0805664105

46. Stephen ID, Law Smith MJ, Stirrat MR, Perrett DI. Facial skin coloration affects perceived health of human faces. Int J Primatol. 2009;30: 845–857. doi:10.1007/s10764-009-9380-z

47. Stephen ID, Oldham FH, Perrett DI, Barton RA. Redness enhances perceived aggression, dominance and attractiveness in men's faces. Evolutionary Psychology. 2012;10: 562–572. doi:10.1177/147470491201000312

48. Borras-Guevara ML, Batres C, Perrett DI. Fear of Violence among Colombian Women Is Associated with Reduced Preferences for High-BMI Men. Human Nature. 2019;30: 341–369. doi:10.1007/s12110-019-09350-8

49. Carré JM, Archer J. Testosterone and human behavior: the role of individual and contextual variables. Curr Opin Psychol. 2018;19: 149–153. doi:10.1016/j.copsyc.2017.03.021

50. Geniole SN, Bird BM, McVittie JS, Purcell RB, Archer J, Carré JM. Is testosterone linked to human aggression? A meta-analytic examination of the relationship between baseline, dynamic, and manipulated testosterone on human aggression. Horm Behav. 2020;123: 104644. doi:10.1016/j.yhbeh.2019.104644

51. Puts DA, Welling LLM, Burriss RP, Dawood K. Men's masculinity and attractiveness predict their female partners' reported orgasm frequency and timing. Evolution and Human Behavior. 2012;33: 1–9. doi:10.1016/j.evolhumbehav.2011.03.003

52. Foo YZ, Rhodes G, Simmons LW. The carotenoid beta-carotene enhances facial color, attractiveness and perceived health, but not actual health, in humans. Behavioral Ecology. 2017;28: 570–578. doi:10.1093/beheco/arw188

53. Kleisner K, Kočnar T, Tureček P, Stella D, Akoko RM, Třebický V, et al. African and European perception of African female attractiveness. Evolution and Human Behavior. 2017;38: 744–755. doi:10.1016/j.evolhumbehav.2017.07.002

54. Alaluf S, Atkins D, Barrett K, Blount M, Carter N, Heath A. The impact of epidermal melanin on objective measurements of human skin colour. Pigment Cell Res. 2002;15: 119–126. doi:10.1034/j.1600-0749.2002.1o072.x

55. Visscher MO. Skin Color and Pigmentation in Ethnic Skin. Facial Plast Surg Clin North Am. 2017;25: 119–125. doi:10.1016/j.fsc.2016.08.011

56. Del Bino S, Bernerd F. Variations in skin colour and the biological consequences of ultraviolet radiation exposure. British Journal of Dermatology. 2013;169: 33–40. doi:10.1111/bjd.12529

57. Tiddeman B, Perrett D. Transformation of dynamic facial image sequences using static 2D prototypes. Visual Computer. 2002;18: 218–225. doi:10.1007/s003710100142

58. Coetzee V, Faerber SJ, Greeff JM, Lefevre CE, Re DE, Perrett DI. African Perceptions of Female Attractiveness. PLoS One. 2012;7: 3–8. doi:10.1371/journal.pone.0048116

59. Fink B, Grammer K, Matts PJ. Visible skin color distribution plays a role in the perception of age, attractiveness, and health in female faces. Evolution and Human Behavior. 2006;27: 433–442. doi:10.1016/j.evolhumbehav.2006.08.007

60. Lefevre CE, Perrett DI. Fruit over sunbed: Carotenoid skin colouration is found more attractive than melanin colouration. Quarterly Journal of Experimental Psychology. 2015;68: 284–293. doi:10.1080/17470218.2014.944194

61. Paschetta C, de Azevedo S, Ramallo V, Cintas C, Pérez O, Navarro P, et al. The impact of socioeconomic and phenotypic traits on self-perception of ethnicity in Latin America. Sci Rep. 2021;11: 1–11. doi:10.1038/s41598-021-92061-x

62. Telles EE. Pigmentocracies: Ethnicity, Race and Color in Latin America. North Carolina: UNC Press Book; 2014.

63. Bolker BM, Brooks ME, Clark CJ, Geange SW, Poulsen JR, Stevens MHH, et al. Generalized linear mixed models: a practical guide for ecology and evolution. Trends Ecol Evol. 2009;24: 127–135. doi:10.1016/j.tree.2008.10.008

64. Burnham KP, Anderson DR. Multimodel inference: Understanding AIC and BIC in model selection. Sociol Methods Res. 2004;33: 261–304. doi:10.1177/0049124104268644

65. Zuur AF, Ieno EN, Walker NJ, Saveliev AA, Smith GM. Mixed Effects Models and Extensions in Ecology with R. NewYork: Springer; 2009.

66. Zuur AF, Ieno EN. A protocol for conducting and presenting results of regression-type analyses. Methods Ecol Evol. 2016;7: 636–645. doi:10.1111/2041-210X.12577

67. Bates D, Mächler M, Bolker BM, Walker SC. Fitting linear mixed-effects models using lme4. J Stat Softw. 2015;67. doi:10.18637/jss.v067.i01

68. Bolker M Ben. Package ‘ bbmle .’ 2021.

69. R Core Team. R: A language and environment for statistical computing. Vienna, Austria: R Foundation for Statistical Computing.; 2020.

70. Oosterhof NN, Todorov A. The functional basis of face evaluation. Proc Natl Acad Sci U S A. 2008;105: 11087–11092. doi:10.1073/pnas.0805664105

71. Dixon AR. Colorism and classism confounded: Perceptions of discrimination in Latin America. Soc Sci Res. 2019;79: 32–55. doi:10.1016/j.ssresearch.2018.12.019

72. Kleisner K, Tureček P, Roberts SC, Havlíček J, Valentova JV, Akoko RM, et al. How and why patterns of sexual dimorphism in human faces vary across the world. Sci Rep. 2021;11: 1–14. doi:10.1038/s41598-021-85402-3

73. Puts DA. Human sexual selection. Curr Opin Psychol. 2016;7: 28–32. doi:10.1016/j.copsyc.2015.07.011

74. Puts DA. Beauty and the beast: Mechanisms of sexual selection in humans. Evolution and Human Behavior. 2010;31: 157–175. doi:10.1016/j.evolhumbehav.2010.02.005

75. Windhager S, Schaefer K, Fink B. Geometric morphometrics of male facial shape in relation to physical strength and perceived attractiveness, dominance, and masculinity. American Journal of Human Biology. 2011;23: 805–814. doi:10.1002/ajhb.21219

76. Puts DA, Apicella CL, Cárdenas RA. Masculine voices signal men's threat potential in forager and industrial societies. Proceedings of the Royal Society B: Biological Sciences. 2012;279: 601–609. doi:10.1098/rspb.2011.0829

77. Rosenfield KA, Sorokowska A, Sorokowski P, Puts DA. Sexual selection for low male voice pitch among Amazonian forager-horticulturists. Evolution and Human Behavior. 2020;41: 3–11. doi:10.1016/j.evolhumbehav.2019.07.002

78. Batres C, Re DE, Perrett DI. Influence of Perceived Height, Masculinity, and Age on Each Other and on Perceptions of Dominance in Male Faces. Perception. 2015;44: 1293–1309. doi:10.1177/0301006615596898

79. Waller BM, Kavanagh E, Micheletta J, Clark PR, Whitehouse J. The face is central to primate multicomponent signals. Int J Primatol. 2022. doi:10.1007/s10764-021-00260-0

80. Karczmarek P, Pedrycz W, Kiersztyn A, Rutka P. A study in facial features saliency in face recognition: an analytic hierarchy process approach. Soft comput. 2017;21: 7503–7517. doi:10.1007/s00500-016-2305-9

81. Rotshtein P, Geng JJ, Driver J, Dolan RJ. Role of features and second-order spatial relations in face discrimination, face recognition, and individual face skills: Behavioral and functional magnetic resonance imaging data. J Cogn Neurosci. 2007;19: 1435–1452. doi:10.1162/jocn.2007.19.9.1435

82. Scott IML, Pound N, Stephen ID, Clark AP, Penton-Voak IS. Does masculinity matter? The contribution of masculine face shape to male attractiveness in humans. PLoS One. 2010;5. doi:10.1371/journal.pone.0013585

83. DeBruine LM. Trustworthy but not lust-worthy: Context-specific effects of facial resemblance. Proceedings of the Royal Society B: Biological Sciences. 2005;272: 919–922. doi:10.1098/rspb.2004.3003

84. Mogilski JK, Welling LLM. The relative contribution of jawbone and cheekbone prominence, eyebrow thickness, eye size, and face length to evaluations of facial masculinity and attractiveness: A conjoint data-driven approach. Front Psychol. 2018;9: 1–11. doi:10.3389/fpsyg.2018.02428

85. Torrance JS, Wincenciak J, Hahn AC, DeBruine LM, Jones BC. The relative contributions of facial shape and surface information to perceptions of attractiveness and dominance. PLoS One. 2014;9. doi:10.1371/journal.pone.0104415

86. Třebický V, Saribay SA, Kleisner K, Akoko RM, Kočnar T, Valentova JV, et al. Cross-Cultural Evidence for Apparent Racial Outgroup Advantage: Congruence between Perceived Facial Aggressiveness and Fighting Success. Sci Rep. 2018;8: 1–7. doi:10.1038/s41598-018-27751-0

87. Mitteroecker P, Windhager S, Müller GB, Schaefer K. The morphometrics of "masculinity" in human faces. PLoS One. 2015;10: 1–13. doi:10.1371/journal.pone.0118374

88. Windhager S, Schaefer K, Fink B. Geometric morphometrics of male facial shape in relation to physical strength and perceived attractiveness, dominance, and masculinity. American Journal of Human Biology. 2011;23: 805–814. doi:10.1002/ajhb.21219

89. Rhodes G, Lee K, Palermo R, Weiss M, Yoshikawa S, Clissa P, et al. Attractiveness of own-race, other-race, and mixed-race faces. Perception. 2005;34: 319–340. doi:10.1068/p5191

90. INEGI. ENCUESTA NACIONAL SOBRE DISPONIBILIDAD Y USO DE TECNOLOGÍAS DE LA INFORMACIÓN EN LOS HOGARES (ENDUTIH) 2021. Encuesta Nacional Sobre Disponibilidad Y Uso De Tecnologías De La Información En Los Hogares (Endutih) 20211. 2022; 1–19.

91. INEGI. Los extranjeros en México. XII Censo General de Población y Vivienda, 2000. 2014; 67.

92. Russell R. Sex, beauty, and the relative luminance of facial features. Perception. 2003;32: 1093–1107. doi:10.1068/p5101

93. Oh DW, Dotsch R, Todorov A. Contributions of shape and reflectance information to social judgments from faces. Vision Res. 2019;165: 131–142. doi:10.1016/j.visres.2019.10.010

94. Han C, Wang H, Hahn AC, Fisher CI, Kandrik M, Fasolt V, et al. Cultural differences in preferences for facial coloration. Evolution and Human Behavior. 2018;39: 154–159. doi:10.1016/j.evolhumbehav.2017.11.005

95. Stephen ID, Scott IML, Coetzee V, Pound N, Perrett DI, Penton-Voak IS. Cross-cultural effects of color, but not morphological masculinity, on perceived attractiveness of men's faces. Evolution and Human Behavior. 2012;33: 260–267. doi:10.1016/j.evolhumbehav.2011.10.003

96. Whitehead RD, Re D, Xiao D, Ozakinci G, Perrett DI. You are what you eat: Within-subject increases in fruit and vegetable consumption confer beneficial skin-color changes. PLoS One. 2012;7: 1–9. doi:10.1371/journal.pone.0032988

97. Whitehead RD, Ozakinci gözde, Perrett DI. Attractive skin coloration: Harnessing sexual selection to improve diet and health. Evolutionary Psychology. 2012;10: 842–854. doi:10.1177/147470491201000507

Supporting information

S1 Fig. Participant's self-perceived skin color ratings. PERLA scale distribution of the 700 participant's own skin color ratings. The color levels go from 1 to 10, ~~being~~ 1 the lightest and 10 the darkest. 11 (1.6 %) picked skin tone level 1, 36 (5.1%) skin tone level 2, 42 (6%) skin tone level 3, 240 (34.3%) skin tone level 4, 201 (28.8%) skin tone level 5, 102 (14.6%) skin tone level 6, 45 (6.4%) skin tone level 7, 14 (2%) skin tone level 8, 7 (1%) skin tone level 9, and 1 (0.1%) skin tone level 10.

S1 Table. Perception estimates with 95% confidence intervals with all participants included. Estimates (Est.) with 95% confidence intervals (CI) of the fixed predictors of the models for: (a) Perceived attractiveness, (b) Perceived trustworthiness, (c) Perceived health, (d) Perceived dominance, (e) Perceived aggressiveness, and (f) Perceived masculinity. *Note:* (□*)* The dominance best ~~fitted~~ model did not include the skin color transformation predictor, therefore the estimates shown represent the main effect of the natural skin color group only.

S2 Table. Pearson's correlation analysis between the scores of all perceptions rates of the natural versions only. Pearson's correlation between the natural versions scores of attractiveness, trustworthiness, perceived health, dominance, masculinity, and aggressiveness. *$p<0.05$, **$p<0.01$, ***$p<0.001$.

Figure

Click here to access/download;Figure;Fig1.tif

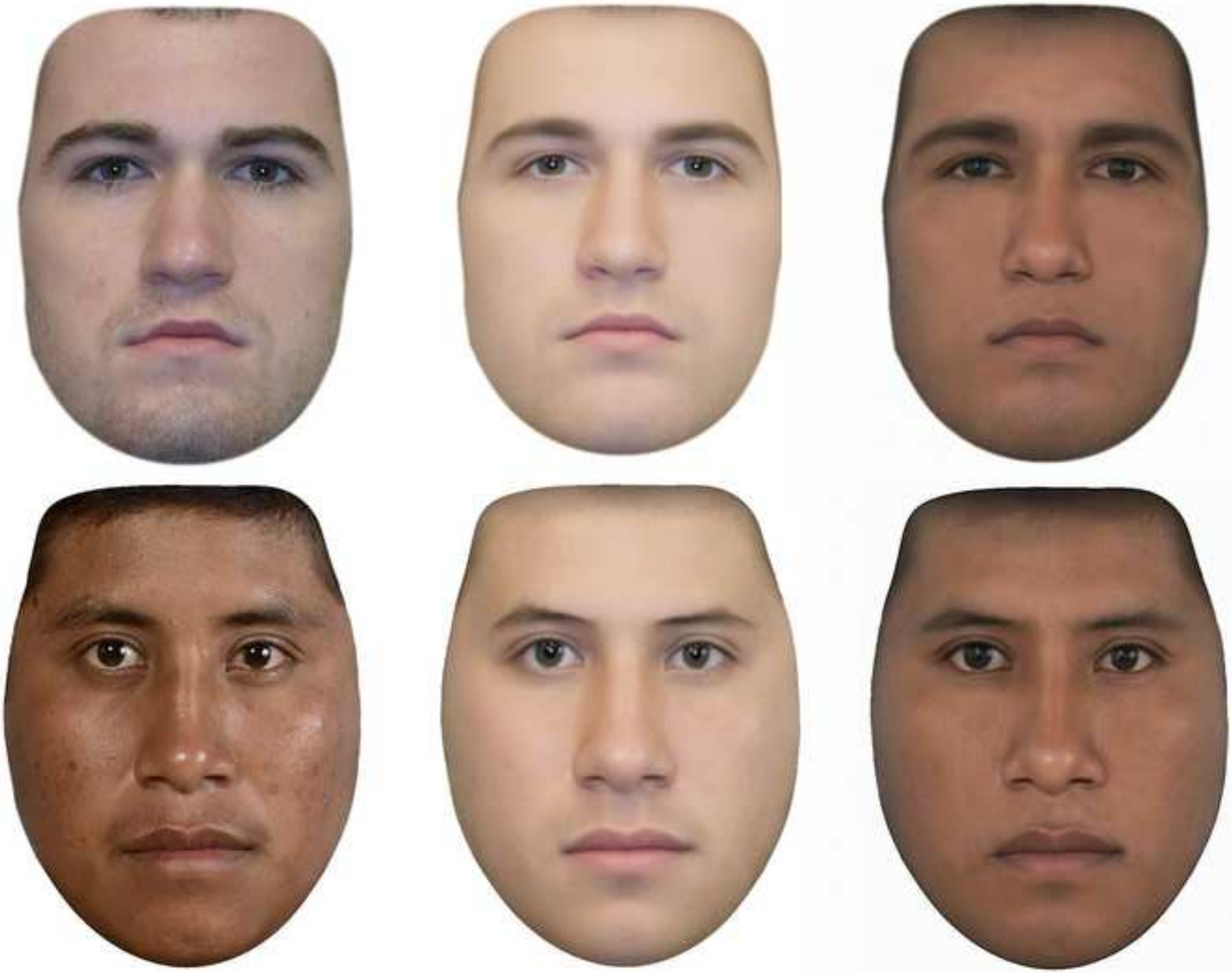

Figure

Click here to access/download;Figure;Fig2.tif

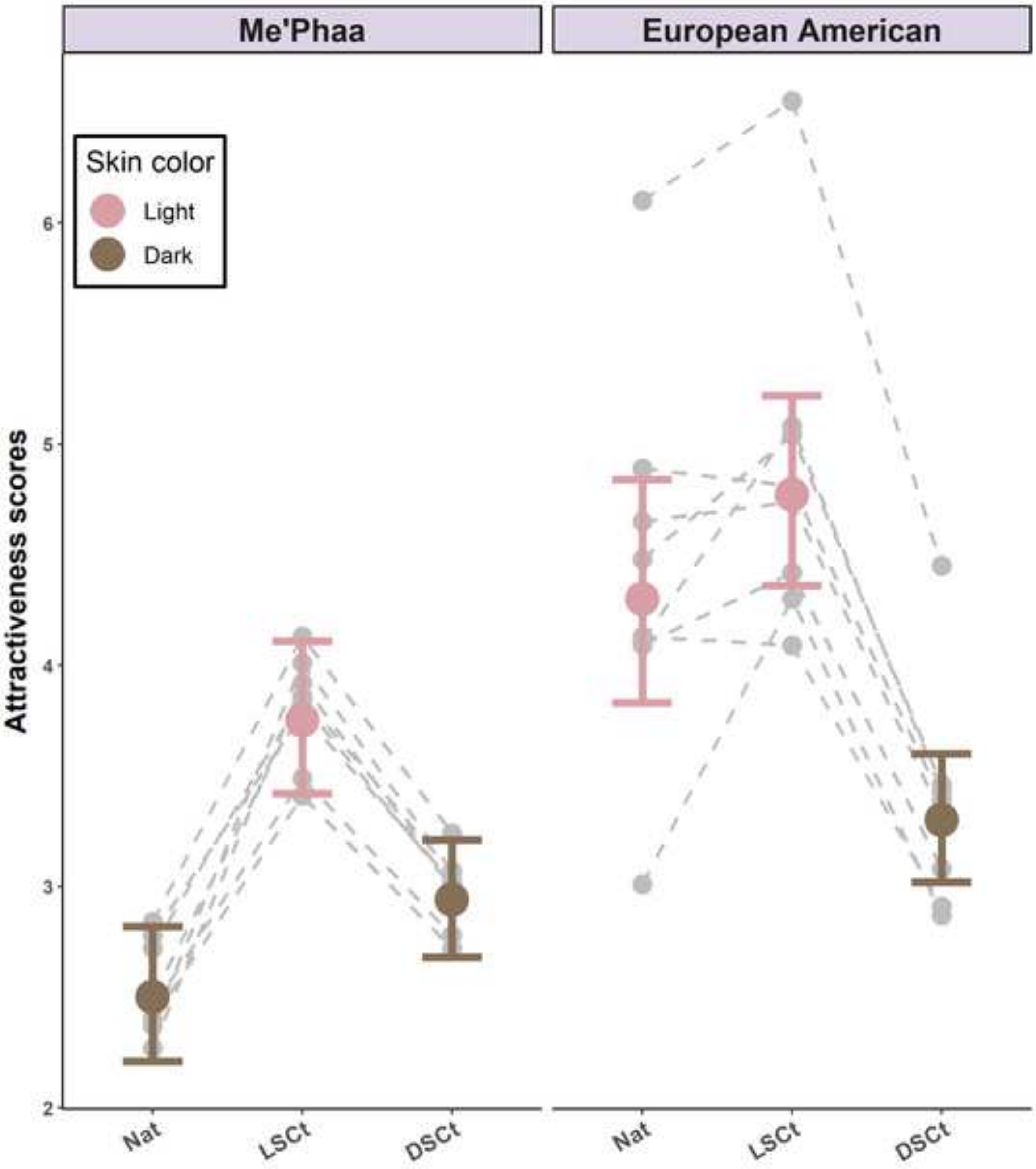

Figure Click here to access/download;Figure;Fig3.tif

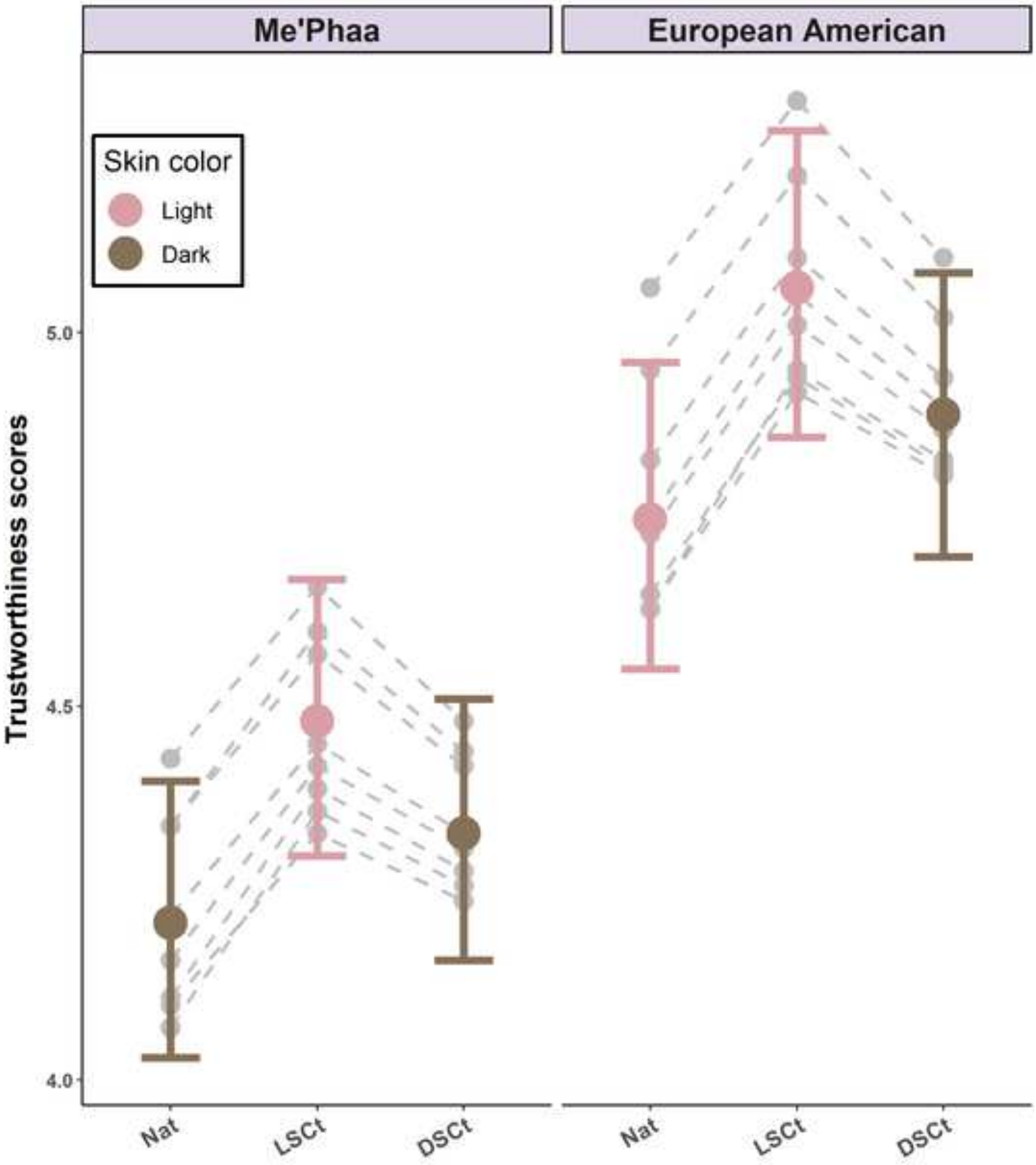

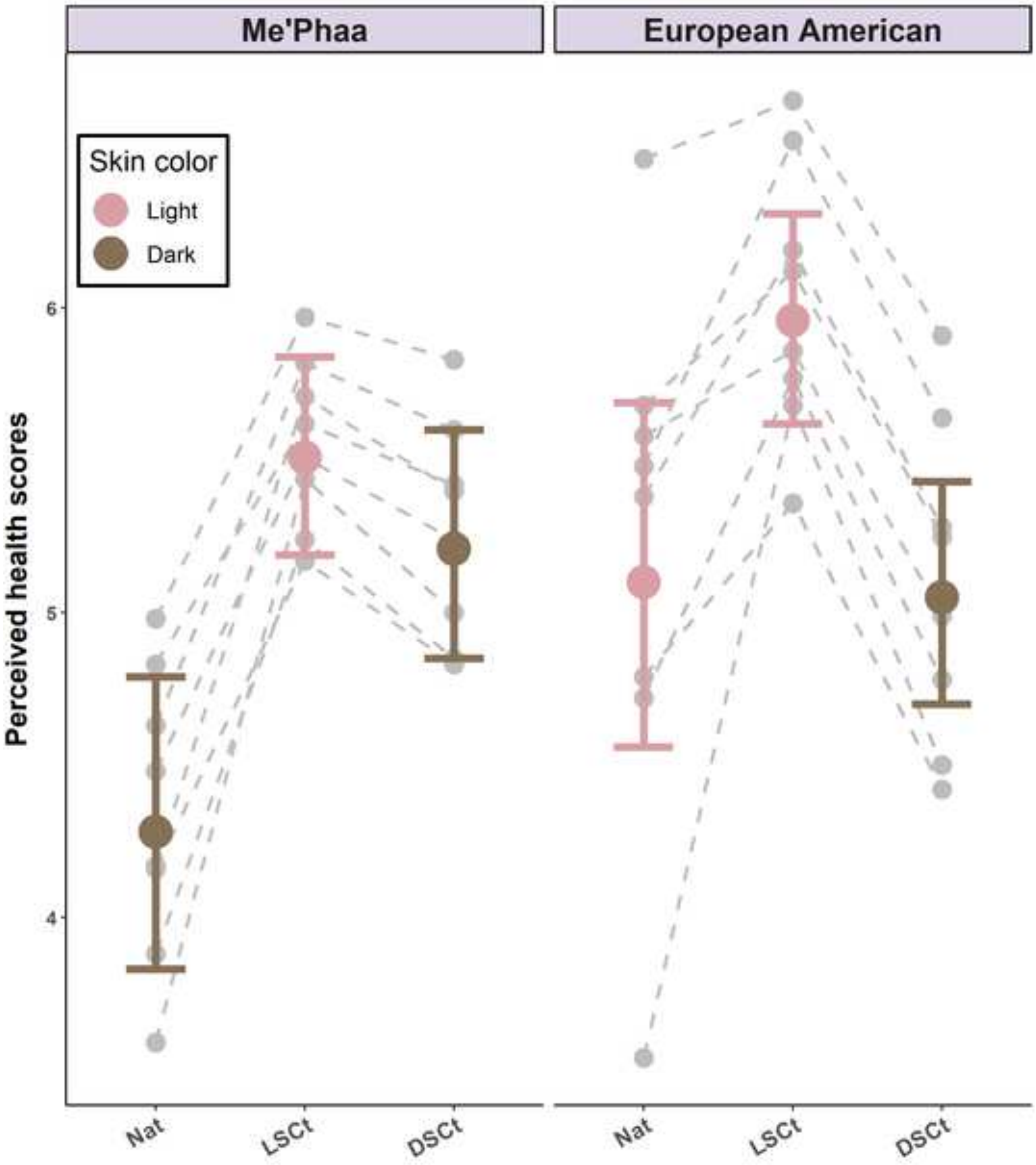
Me'Phaa
European American
Skin color
Light
Dark
Perceived health scores
6
5
4
Nat
LSCt
DSCt
Nat
LSCt
DSCt

Figure

Click here to access/download;Figure;Fig5.tif

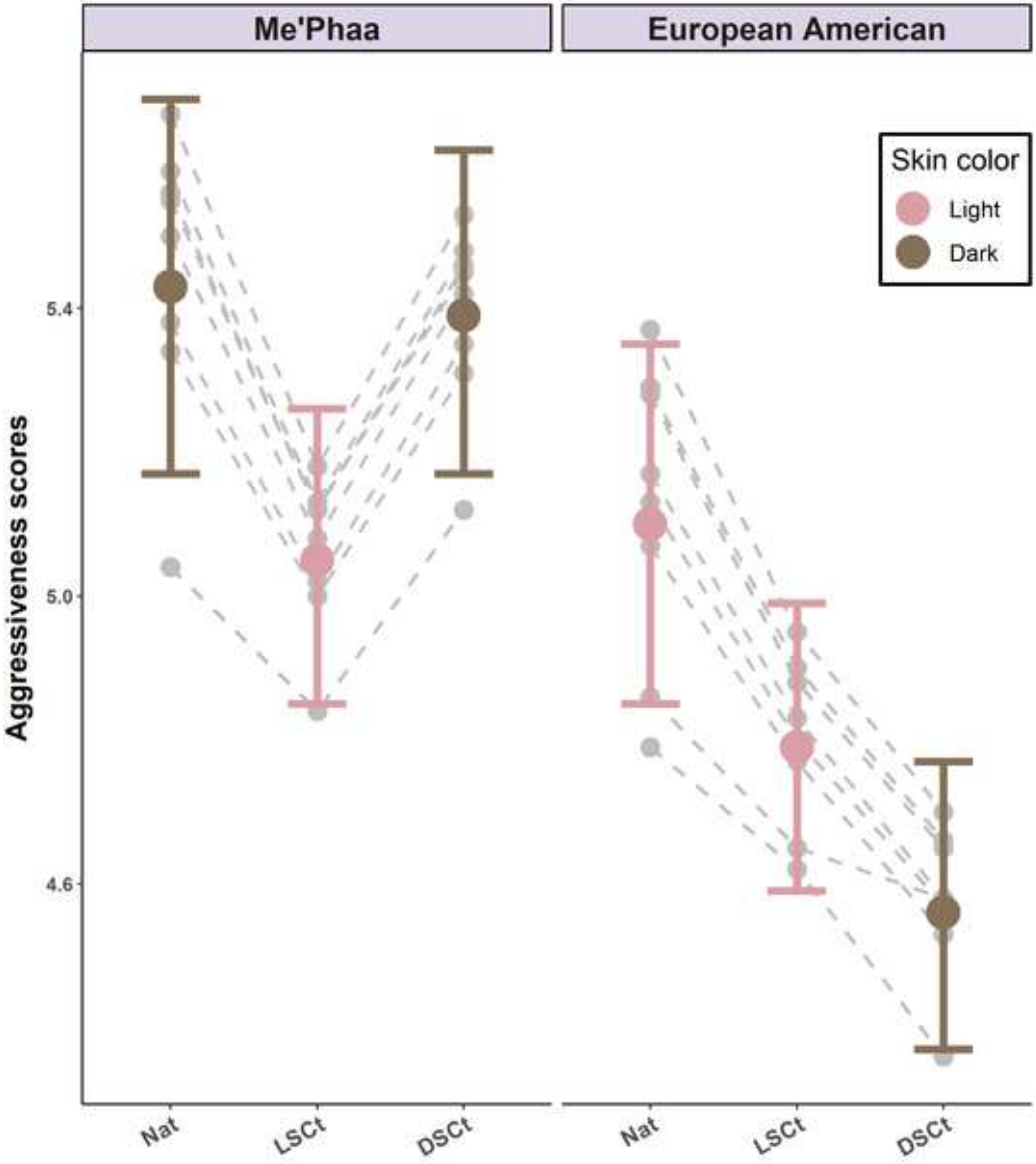

Figure

Click here to access/download;Figure;Fig6.tif

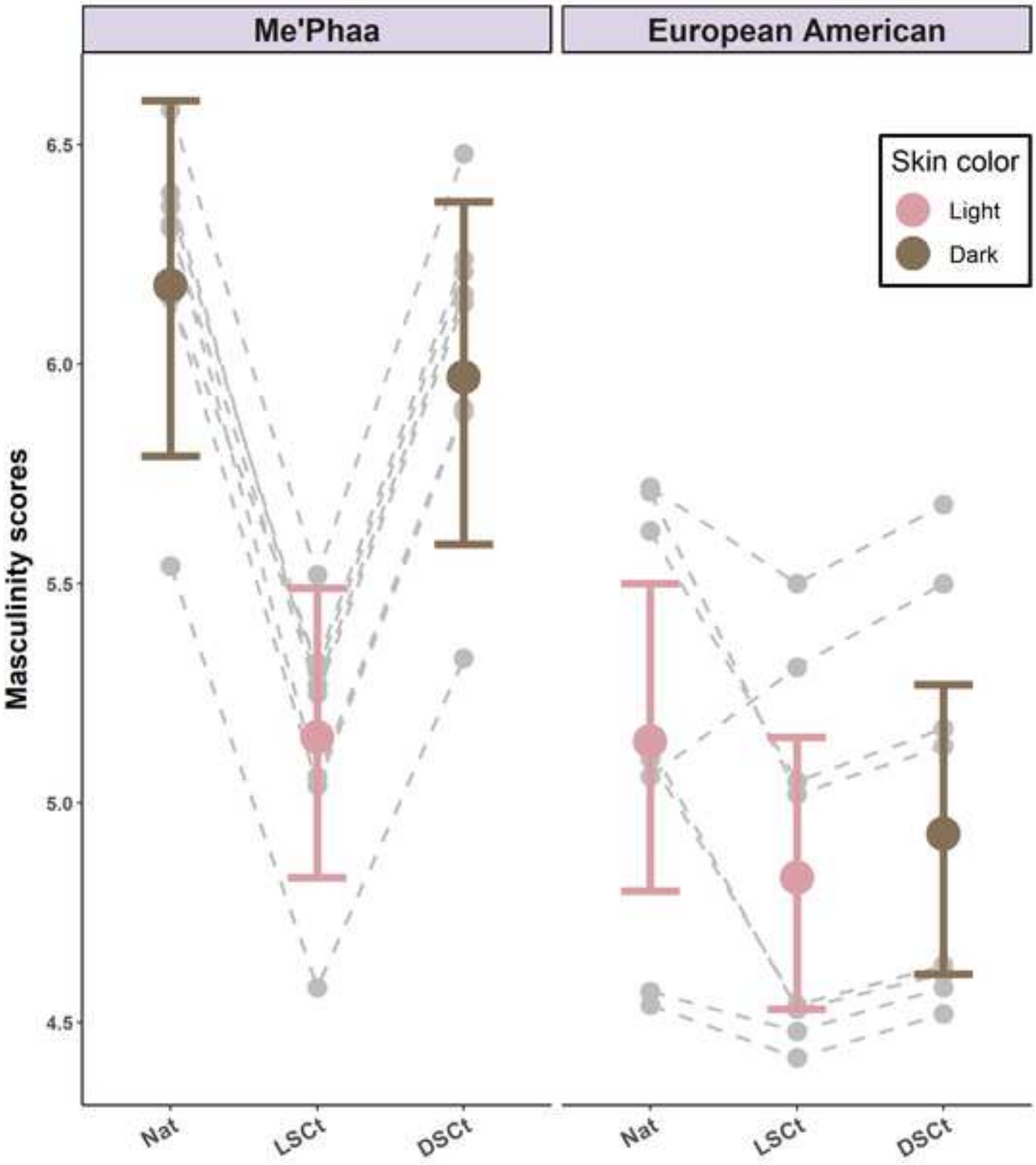

Click here to access/download
Supporting Information
S1_Fig.pdf

Click here to access/download
Supporting Information
S1_Table.pdf

Click here to access/download
Supporting Information
S2_Table.pdf

Effects of facial skin pigmentation on social judgments in a Mexican population

Jaaziel Martínez-Ramírez[1] , David Puts[2] , Javier Nieto[3] , Isaac G-Santoyo[1,4*]

1. Neuroecology lab, Faculty of Psychology, National Autonomous University of Mexico, Mexico City, Mexico.
2. Department of Anthropology, Pennsylvania State University, Pennsylvania, United States of America.
3. Laboratory of Learning and Adaptation, Faculty of Psychology, National Autonomous University of Mexico, Mexico City, Mexico.
4. Unidad de Investigación en Psicobiología y Neurociencias, National Autonomous University of Mexico, Mexico City, Mexico.

* Corresponding author

E-mail: isantoyo@psicologia.unam.mx (IG-S)

Abstract

People quickly and involuntarily form impressions of others based on their facial physical attributes, which can modulate critical social interactions. Skin pigmentation is one of the most variable and conspicuous facial traits among human populations. Empirical evidence suggests that these variations reflect ancestral ecological selective pressures balancing cutaneous vitamin D synthesis with the protection of the dermis from ultraviolet radiation. Nevertheless, skin pigmentation may currently be subject to additional selective pressures. For instance, the colonial era in Central and South America developed a highly stratified society based on ethnic origins, and light skin pigmentation became associated with social status. Facial skin pigmentation is also sexually dimorphic, with males tending to exhibit darker skin than females, a difference that could be driven by sexual selection. To explore whether social learning and sexual selection represent additional selective pressures on skin pigmentation, we tested how this facial trait influences fundamental social perceptions in a Mexican population (N = 700, 489 female). We sampled facial images of eight European American males with natural light facial skin pigmentation and eight males from an indigenous pre-Columbian community from Mexico, the Me'Phaa, with naturally darker facial skin pigmentation. We produced stimuli from these images by interchanging skin pigmentation while preserving the facial shape. Stimuli were rated on attractiveness, trustworthiness, perceived health, dominance, aggressiveness, and femininity/masculinity. We found that the natural light-skinned faces of European American males were perceived as more attractive, trustworthy, and healthy but less dominant than the natural dark Me'phaa faces. Furthermore, the interchange of facial skin color in these original groups reversed rates of these perceptions, mainly the attractiveness perception. These results

partially support the hypothesis that dark facial skin color may aid male-male contest competition. However, they more strongly support that light skin pigmentation became associated with favorable attributes related to pro-sociality due to possible social learning in this Mexican population.

Introduction

Human faces provide rich information about salient attributes, such as mate quality [1,2], social dominance [3], and fecundity [4]. Hence, people quickly and involuntarily form impressions of others based on their facial appearance [5,6], which in turn may influence important social outcomes [7,8]. Skin pigmentation is one of the most conspicuous and variable facial traits between human populations [9]. The main pigments—melanin, hemoglobin, and carotenoids—absorb and reflect different wavelengths of light within the visible spectrum and collagen also contributes by scattering light [10,11]. The skin's pigments vary not only between individuals and populations but also between men and women, perhaps because of sexual selection [12]. Natural selection initially favored darker skin as a mean to protect against the harmful effects of UV radiation in the tropics. The pressure of selection then shifted toward lighter skin among those humans who spread into higher latitudes with lower levels of UV [13–15]. Melanin, the principal skin pigment that provides this gradient of light to dark color, acts as a photoprotective filter that reduces the penetration of all wavelengths of light into subepidermal tissue. Under the high UVR levels near the equator, dark skin pigmentation is particularly advantageous for protecting folate and reducing the risk of fatal birth defects. Human migration outside the tropics reduced the selective advantage of this photoprotective property and selected instead for lower melanization to facilitate cutaneous vitamin D biosynthesis, critical to the regulation of many biological processes [16]. Although these events are widely accepted as the main evolutionary events generating

human skin pigment variation, this product of natural selection could be currently subjected to other ecological pressures in addition to what caused its origin.

Learning is the psychological mechanism responsible for cultural transmission between conspecifics by promoting different social behaviors that could influence survival and reproduction [17]. Cultural transmission might be adaptive if a transmitted behavior saves learners the costs of individual learning. When this occurs, the selection process will favor improved learning efficiencies and behaviors in the learner that lead to better learning environments. For instance, social learning can influence mate preferences through two different mechanisms [18]; reinforcing a preference for a trait that may signal biological quality if that preference significantly contributes to reproductive success, such as offspring quality, or promoting new preferences for traits that do not appear to have direct links to the bearer's biological quality [19]. In this case, new social preferences may be selected by cultural transmission if the novel trait positively influences social benefits in a particular population [20].

The colonial period in Mexico and all countries of Central and South America led to a highly stratified society based on ethnic origins. During the 300 years that lasted this period, light skin pigmentation, usually present in Spanish colonizers, was one of the most conspicuous phenotypic traits associated with higher social status and hence access to social benefits, such as education and wealth [21]. This racial/color hierarchy system, known as *castes*, placed the European colonizers at the top of the social hierarchy and the indigenous populations at the bottom, it developed deep roots in Latin American society, and even though the *castes* system is officially abolished, there is robust evidence of social stratification based on skin color in the contemporary Mexican society [22]. For example,

recent studies find that people with darker skin color have, on average, lower educational levels, occupational status, and hourly earnings, are more likely to live in poverty, and have fewer chances of social mobility in comparison with people of lighter skin colors; these differences are significant even after controlling for sociodemographic factors such as age, gender, and parental education [22–25]. In addition, a cultural preference for light skin pigmentation and other European phenotypical features are also being influenced by social media in Western societies, which might reinforce the association between skin color and social status. Hence, if light skin pigmentation is culturally preferred because it is associated with social benefits, prestige, or deference, a new skin pigmentation-based signal may have emerged due to an extension of historical, sociocultural learning.

Another non-mutually exclusive hypothesis is that facial skin pigmentation could be driven by perceptions shaped by sexual selection. Human skin pigmentation is a sexually dimorphic trait in which men usually have darker skin pigmentation than women [26–28]. The sex difference is due to exposure of skin tissues to differing ratios of androgens to estrogens, particularly at puberty. Testosterone has a stronger effect than estrogen on melanin synthesis and vascularization of the upper dermis [29–31]. In addition, males undergo a more intense facultative pigmentation after sun exposure and retain it for longer periods than females whereas female skin lightens faster after a reduction in sun exposure [32]. This sexual dimorphism might influence sexual preferences since several studies have shown that darker skin color in men is perceived more attractive [33–36]. Therefore, this female preference for darker skin color could reflect ancestral selection pressures shaping perceptions of an androgen-dependent trait. In this case, the basis of this preference may be the honest signaling of mate quality in terms of male immune function (i.e.,

immunocompetence handicap hypothesis), vigor, or other physiological costs of developing this trait [37–39].

Although the effects of skin pigmentation on social perceptions is an active area of research in social psychology, prior research has focused mainly on racial attitudes and generally does not employ an evolutionary framework. For instance, Hagiwara et al. [40] evaluated affective reactions and other social attributions towards African American men by manipulating facial shape features (nose and lips) while controlling for skin tone. Similar studies have examined the role of skin tone and facial shape features on racial attitudes following similar methods [41,42]. Moreover, most experimental studies of facial perception have been carried out in participants from WEIRD (Western, educated, industrialized, rich, and democratic) societies, limiting the generalizability of findings [43]. Furthermore, prior studies carried out in non-WEIRD Latin American samples have implemented a social theoretical framework with a non-experimental design to explore correlations between skin tone and social attributes, such as status or labor hierarchy [44].

In this study, we used an experimental design with a Mexican population to determine whether facial skin color can influence certain social perceptions: attractiveness; attractiveness, trustworthiness, perceived health, dominance, aggressiveness, and femininity/masculinity [45–47]. To that end, we performed a crossover experiment on men's faces, selecting two groups of faces with contrasting innate skin pigmentation: the first composed of faces with natural light facial skin tone, selected from a European American population, while the second group of faces with a darker facial skin tone, selected from a Mexican pre-Columbian population, self-identified as Me'Phaa. Using these two groups, we created six sets of facial stimuli interchanging the natural skin pigmentation

but preserving the facial shape. Finally, we presented all stimuli to be evaluated in the six social dimensions previously mentioned.

We hypothesize that if social learning is acting on the perceptions of facial skin pigmentation, we expect that the lighter skin faces group would be perceived as more attractive, trustworthy, and healthier since positive evaluations of these attributes strongly promote pro-sociality and social benefits but less dominant and aggressive because these perceptions, in contrast, are considered as potentially harmful and may lead to avoidance of social interactions [48]. However, if skin pigmentation more strongly reflects ancestral selection shaping perceptions of an androgen-dependent trait, then we would expect that faces with darker skin color would be perceived not only as more attractive and healthier but also as more dominant, aggressive, and masculine due to the association between these last three attributes to the behavioral and physiological effects of androgens [49,50]. In both cases, if differences in skin pigmentation are the principal facial trait driving these effects, we expect that the experimental manipulation of the facial skin color would change the previous patterns in the perceptions in both the European American and Me´Phaa faces. Finally, if neither light nor dark skin color affects any social perception, both in the original faces and experimentally exchanged stimuli, then skin color would not function in the social selection nor the sexual selection of dimorphic traits.

Material and methods

Participant raters

A total of 489 Mexican adult women (mean age = 25.4 years, SD = 8.6 years) and 211 Mexican adult men (mean age = 26.5 years, SD = 9.1 years) participated as raters in

this study. Self-reported ethnicities were 84.0 % Mestizo, 6.3 % European, 3.9 % Indigenous (particular groups not specified), 0.3 % African, and 6.3 % Other. A total of 485 participants reported living in Mexico City, 95 in the surrounded metropolitan area, and the rest in different states of Mexico. All Participant raters were recruited online by a board advertisement from the Department of Psychology at the National Autonomous University of Mexico.

Skin color facial stimuli

To develop the experimental manipulation in facial skin pigmentation, we first created two contrasting light and dark facial skin color prototypes. This process was made selecting 30 facial photographs of young European American men and 30 facial photographs of young Me’Phaa Mexican Indigenous men from larger photograph sets. (See below the criteria of selection for both groups of photographs). The European American faces were selected from a set of facial photographs collected in the Department of Anthropology at the Pennsylvania State University [51]. Facial photographs were taken from a distance of 2 m using a 12-megapixel Olympus E-300 camera with a mounted flash at a resolution of 1200 × 1000 pixels. The facial photographs of the Me’Phaa were selected from a collection of facial photographs from the NeuroEcology Lab at the National Autonomous University of Mexico. These facial photographs were taken from a distance of 2m using a 21-megapixel Nikon 5500 camera illuminated by two studio strobes (Lusana Studio, 400w) with reflective umbrellas. The strobes were placed 1.85 m from the target on each side and lights were arrange in 40° angles. In both sets of facial photographs participants were asked to remove any earrings, glasses, or facial jewelry, and to maintain a neutral expression.

The two groups of facial photographs (European American and Me´Phaa) were selected in order to obtain two samples of faces whose facial skin pigmentation was contrasting, one group with lighter skin and color ranges typical for the European American average, and another group with darker skin and color ranges typical for an Indigenous Mexican average. Therefore, in order to evaluate if the facial photographs in both groups met these criteria, we measured its skin color values in CIELab color space. Following procedures reported in previous studies [52,53], we first standardized the facial images by color calibration, then we took color samples of three separate patches located on the forehead and on each cheek and calculated the average CIELab values using Photoshop CS6. Given that lightness (L^*) values are negative correlated with some biochemical measurements of melanin content such as the total melanin content of the epidermis and vary with ethnicity [54], we focused on this parameter to evaluate skin color differences between the two selected groups. European American (L^* mean = 61.08, SD = 6.42) and Me'Phaa (L^* mean = 49.84, SD = 4.05) lightness mean values were statistically different ($t(58) = -8.07$, $p < 0.01$), and lightness values for both groups were in the ranges for White/European and Hispanic ethnicities reported in previous studies [55,56].

From the 60 facial photographs selected, we created two composite faces that averaged the skin color of the 30 original faces from each group using the software Psychomorph [57]. The averaged composite face of the European American men was used as the light skin color prototype, while the composite face of the Me'Phaa men was the dark skin color prototype. Next, we randomly selected eight faces from the European American men's set and eight from the Me'Phaa men's set and created two different versions of each face, one displaying light skin color and the one displaying dark skin

color. These two versions were created by adding or subtracting 70% of the skin color differences between the light and dark facial skin color prototypes previously created but leaving facial shape unchanged. Finally, we repeated the skin color measurement process (see above) in both groups of the new composite faces (light skin color transformed and dark skin color transformed faces). The average lightness value was L^* = 66.74, SD = 1.37 in the light skin color transformed versions and L^* = 52.75, SD = 2.58 in the dark skin color transformed versions. We observed the method we used for skin color manipulation slightly increased the lightness values compared to the values we observed in the natural faces, however these differences were not statistically significant for any group (t (44) = -0.52, $p = 0.6$; t (44) = -0.43, $p = 0.6$), and both groups stayed within the ranges reported for White/European and Hispanic ethnicities in previous studies [55,56]. At the end of this process, we obtained three versions for each of the eight European American and the eight Me’Phaa faces: a natural unmodified version (NAT), a light skin color transformed version (LSCt), and dark skin color transformed version (DSCt; Fig. 1), resulting in a total of 48 facial stimuli.

Fig. 1. Example stimuli. Three versions of the same facial stimuli of a European American male (up) and a Me’Phaa male (bottom). Natural unmodified versions (left), Light Skin Color transformed versions (middle), and Dark Skin Color transformed versions (right).

Facial perception rates

To obtain the ratings, we divided the 48 stimuli into eight sets of six stimuli each. The stimuli were assigned into every set counterbalancing by group (European American and Me’Phaa) and version (NAT, LSCt, and DSCt) and we made sure that two or more versions of the same face were not included together in the same set. Given that the

experimental transformation resulted in the homogenization of facial skin color and texture, two features that affect perceptions of human faces [58,59], we decided to include the natural version of each face (NAT) to be able to compare the ratings of the natural versions with the ratings of the concordant skin color transformed version, that is compare the Natural versions with the Light skin color transformed versions in the European American faces and the Natural versions with the dark skin color transformed versions in the Me'Phaa faces. By doing this we could measure the effect of the color and texture homogenization.

The facial evaluations were performed online at the webpage labneuroecologiacog.com. Each participant was randomly assigned to one of the eight facial stimuli sets and was asked to rate each face separately on a 9-point Likert scale according to the following perceptions; attractiveness (1 = not attractive at all, 9 = very attractive), trustworthiness (1= not trustworthy at all, 9= very trustworthy), perceived health (1= not healthy at all, 9= very healthy), dominance (1 = not dominant at all, 9 = very dominant), aggressiveness (1= not aggressive at all, 9= very aggressive) and femininity/masculinity (1= very femininity/not masculine at all, 9= very masculine/ not femininity at all) . For all trials, we randomized both the order of facial stimuli and the order of the perception asked. In addition, to standardize the response evoked for attractiveness in men and women, we asked the men participants, *"How attractive does this face look for a woman?"* (For similar methods see [60]). Finally, raters completed a socio-demographic survey and were asked about their sexual orientation, implementing a 5-point Likert scale, from entirely same-gender to entirely opposite-gender orientation. As a result, 610 Participant raters (437 women and 173 men) identified themselves as opposite-gender oriented/heterosexual, 42 (15 women and 27 men) identified as same-gender sexual orientation, 35 (28 women and 7 men) identified as bisexual, and 13 (9 women and 4 men)

reported not having any sexual orientation. We controlled the sexual orientation information in the statistical analysis (see below). In addition, self-perceived skin color may have been influencing the rater's responses [23,61]. Hence, participates were asked to rate their own skin color using the PERLA color palette. The PERLA color palette is a scale of 11 skin tones (1 = lightest, 11 = darkest) that was made as an effort to have an easy-to-use tool that summarize the typical skin tones found in regions of Latin America [62]. Similar to previous studies conducted on the Mexican population [24], we found that 77. 6% of participants responses were concentrated in the middle levels of the scale (4-6), while the rest of participants were distributed through the extremes; 12.7 % in the lightest skin tones levels (1-3) and 9.7% the darkest levels (7-10; see S1 Fig.). Finally, all raters signed an informed consent form before evaluating facial stimuli, and all procedures were approved by the appropriate National Autonomous University of Mexico ethics committee (FPSI/CE/01/2016) and are in compliance with Mexican legislation and the Code of Ethical Principles for Medical Research Involving Human Subjects of the World Medical Association (Declaration of Helsinki).

Statistical analysis

To determine whether the experimental manipulation in facial skin color modified the perception of social judgments, we fitted generalized linear mixed models (GLMM) of the Poisson Family with the log link function. We decided to fit Poisson GLMMs because, unlike other studies where the ratings for each facial stimulus are averaged, here all dependent variables come from discrete raw ratings data. Moreover, during the data

exploration we observed a positively skewed counts-type distribution with the larger number of observations at the lower values in some of the facial perceptions (i.e., attractiveness perception). We performed one global model per each perception evaluated. Global models included as the dependent variable any of the following facial perceptions: attractiveness, trustworthiness, perceived health, dominance, aggressiveness or femininity/masculinity. Fixed predictors for all global models included the ethnic group of each evaluated face (i.e., European American or Me'phaa faces), the skin color faces-natural and transformed faces (i.e., NAT, LSCt or DSCt)-sex of participant raters, and the interaction between ethnic group and skin color transformed faces. To account for the potential non-independence of data, we included a random intercept for participant ID, a random intercept for facial photograph ID (the individual code of each original face used), and a random slope for skin color transformation on facial photograph ID.

To evaluate the effect of participant sexual preferences, we performed models both including responses from all participants and responses from heterosexual participants only. Next, each global model was then reduced to obtain a minimum adequate model using the Akaike Information Criterion (AIC). We selected the models with the lowest AIC values and a ΔAIC higher than two units from the second most supported model [63]. When the difference between the best-supported model and the subsequent model was $\Delta<2$, we followed the statistical principle of parsimony [64], and hence we selected the minimum adequate model as the one with the lowest AIC and the least number of parameters (Table 1). Finally, once we obtained minimum adequate models, model diagnostics were performed, checking residual distribution and linearity of residuals in each model. In order to check for any effect of other factors not included in the models [65,66], we plotted

Pearson residuals of each selected model against participant´s self-reported ethnicities and the PERLA scale responses for self-perceived skin color. Since the plots did not show any clear effect, we did not include any of these two factors as fixed predictors. All analyses were performed using the *lme4* [67] and *bbmle* [68] packages in R, version 4.0.2 [69].

Table 1. Information criteria for the best fitted models.

	df	*AICc*	Δ*AICc*	$w_i(AICc)$
(a) Perceived attractiveness				
**Group + Color + Participant sex + Group:Color**	**14**	**14331**	**0.000**	**0.999**
Group + Color + Participant sex	12	14346	15.053	0.001
Group + Color + Group:Color	13	14348	16.957	0.000
(b) Perceived trustworthiness				
**Group + Color**	**11**	**15167**	**0.000**	**0.506**
Group + Color + Participant sex	12	15169	1.778	0.208
Group + Color + Group: Color	13	15170	2.596	0.138
(c) Perceived health				
**Group + Color + Group:Color**	**13**	**15561**	**0.000**	**0.707**
Group + Color + Participant sex + Group:Color	14	15563	1.892	0.274
Group + Color	11	15570	9.186	0.007
(d) Perceived dominance				
**Group + Participant sex**	**10**	**15485**	**0.000**	**0.493**
Group + Color	11	15488	2.685	0.129
Group + Color + Group: Color	13	15489	3.137	0.103
(e) Perceived aggressiveness				
**Group + Color + Group:Color**	**13**	**16625**	**0.000**	**0.663**
Group + Color + Participant sex + Group:Color	14	16626	1.768	0.274
Group + Color	11	16630	5.479	0.043
(f) Perceived masculinity				
**Group + Color + Group:Color**	14	15486	0.000	0.482
Group + Color + Participant sex + Group:Color	**13**	**15486**	**0.035**	**0.474**
Group + Color	11	15492	6.367	0.020

Information criteria and fixed factors included for the three best fitted models for: (a) Perceived attractiveness, (b) Perceived trustworthiness, (c) Perceived health, (d) Perceived dominance, (e) Perceived aggressiveness, and (f) Perceived masculinity.
Models are in descending order. ΔAIC is the change in AIC between each model and the best model. Akaike weights are conditional probabilities for each model being the best model. Models in bold are the selected ones.

Results

Perceived attractiveness

Perceived attractiveness differed significantly between the two groups when they had their natural skin color ($z = 6.51$, $p < 0.01$). The European American faces were rated 1.70 times more attractive ($M = 4.30$, 95% CI [3.83 - 4.84]) than the Me'Phaa faces ($M = 2.50$, 95% CI [2.21 - 2.82], Table 2). Furthermore, there were effects when natural skin color was changed within each of the two groups. Lightening the Me'Phaa faces made them more attractive ($M = 3.75$, 95% CI [3.42 - 4.11], $z = 7.42$, $p < 0.01$), and darkening the European American faces made them less attractive ($M = 3.30$, 95% CI [3.02 - 3.60], $z = -6.25$, $p < 0.01$). In addition, we observed an effect of skin color and texture homogenization due to experimental manipulation. Both groups became slightly more attractive when skin color and skin texture were homogenized (For Me'Phaa faces: $M = 2.94$, 95% CI [2.68 - 3.21]; For European American faces: $M = 4.77$, 95% CI [4.36 - 5.22]). However, this effect was significant for the Me'Phaa faces ($z = -3.23$, $p < 0.01$) but not for the European American faces ($z = -2.00$, $p = 0.34$; Fig. 2). Finally, we observed a significant main effect of participant sex ($z = 4.36$, $p<0.01$); the men participants rated all faces as more attractive ($M = 3.76$, 95% CI [3.48 - 4.06]) than did the female participants ($M = 3.27$, CI 95% [3.06 - 3.49]). The direction of the effects and statistical significance were not affected when including the sexual preference of participants (S1 Table.).

Table 2. Perception estimates with 95% confidence intervals.

	Me'Phaa			European American		
	**NAT**	**LSCt**	**DSCt**	**NAT**	**LSCt**	**DSCt**
	Est. [95%CI]	***Est. [95%CI]***	***Est. [95%CI]***	***Est. [95%CI]***	***Est. [95%CI]***	***Est. [95%CI]***
a) Perceived attractiveness	2.50 [2.21-2.82]	3.75 [3.42-4.11]	2.94 [2.68-3.21]	4.30 [3.83-4.84]	4.77 [4.36 5.22]	3.30 [3.02-3.60]
b) Perceived trustworthiness	4.21 [4.03-4.40]	4.48 [4.30-4.67]	4.33 [4.16-4.51]	4.75 [4.55-4.96]	5.06 [4.86-5.27]	4.89 [4.70-5.08]
c) Perceived health	4.28 [3.83-4.79]	5.51 [5.19-5.84]	5.21 [4.85-5.60]	5.10 [4.56-5.69]	5.96 [5.62-6.31]	5.05 [4.70-5.43]
d) Perceived dominance	5.23 [4.85-5.64]	□	□	4.31 [3.99-4.66]	□	□
e) Perceived aggressiveness	5.43 [5.17-5.69]	5.05 [4.85-5.26]	5.39 [5.17-5.62]	5.10 [4.85-5.35]	4.79 [4.59-4.99]	4.56 [4.37-4.77]
f) Perceived masculinity	6.18 [5.79-6.60]	5.15 [4.83-5.49]	5.97 [5.59-6.37]	5.14 [4.80-5.50]	4.83 [4.53-5.15]	4.93 [4.61-5.27]

Estimates (Est.) with 95% confidence intervals (CI) of the fixed predictors of the models for: (a) Perceived attractiveness, (b) Perceived trustworthiness, (c) Perceived health, (d) Perceived dominance, (e) Perceived aggressiveness, and (f) Perceived masculinity.
Note: (□*)* The Perceived dominance best fitted model did not include the skin color transformation predictor, therefore the estimates shown represent the main effect of the natural skin color group only.

Fig. 2. Perceived attractiveness estimates for the Me'Phaa and European American faces within each color version. Estimates for the Natural versions (NAT), Light skin color transformed versions (LSCt), and Dark skin color transformed versions (DSCt) of the Me'Phaa (left) and European American (Right) faces. Error bars show 95% Confidence Intervals. Grey points and dashed lines indicate the random effect estimates for each Face ID within each color version.

Perceived trustworthiness

Natural European American faces were rated 1.13 times more trustworthy (*M* = 4.75, 95% CI [4.55 - 4.96], $z = 5.19$, $p < 0.01$) than natural Me'Phaa faces (*M* = 4.21, 95% CI [4.03 - 4.40], Table 2). In addition, lightening the Me'Phaa faces increased their perceived trustworthiness (*M* = 4.48, 95% CI [4.30 - 4.67], $z = 3.26$, $p < 0.01$) whereas darkening the European American faces had no effect (*M* = 4.89, 95% CI [4.70 - 5.08], $z = 1.44$, $p = 0.15$). Moreover, there was no effect of skin color and texture homogenization due to experimental manipulation for the Me'Phaa faces (DSCt Me'Phaa faces M = 4.89, 95% CI [4.70 - 5.08], $z = -1.43$, $p = 0.70$), but this increased scores for European American faces (LSCt European American faces *M* = 5.06, 95% CI [4.86 - 5.27], $z = -3.26$, $p = 0.01$; Fig. 3). Finally, there was no effect of participant sex, as this predictor was not present in the minimum adequate model selected by AIC (Table 1). The direction of the effects and statistical significance were not affected when the sexual preference of participants was included (S1 Table.).

Fig. 3. Perceived trustworthiness estimates for Me'Phaa and European American faces within each color version. Estimates for the Natural versions (NAT), Light skin color transformed versions (LSCt), and Dark skin color transformed versions (DSCt) of the Me'Phaa (left) and European American (Right) faces. Error bars show 95% Confidence Intervals. Grey points and dashed lines indicate the random effect estimates for each Face ID within each color version.

Perceived health

As with perceived attractiveness and trustworthiness, we found a significant difference in the perceived health scores between the natural Me'Phaa and European American faces ($z = 2.20$, $p = 0.02$). On average, natural European American faces were perceived as being 1.19 times healthier ($M = 5.10$, 95% CI [4.56 - 5.69]) than natural Me'Phaa faces ($M = 4.28$, 95% CI [3.83 - 4.79], Table 2). When facial skin color was changed within each ethnic group, the changes had different effects on perceptions of the two groups. Lightening the Me'Phaa faces increased their perceived health ($M = 5.51$, 95% CI [5.19 - 5.84], $z = 4.96$, $p < 0.01$), whereas darkening the European American faces had no effect ($M = 5.05$, 95% CI [4.70 - 5.43], $z = 0.20$, $p = 0.99$). Perceived health increased slightly for both groups when skin color and skin texture were homogenized (For European Americans: $M = 5.96$ (95% CI [5.62 – 6.31], $z = -3.12$, $p = 0.02$; For Me'phaa people: $M = 5.21$, 95% CI [4.85 - 5.60], $z = 4.38$, $p < 0.01$; Fig. 4). Finally, there was no effect of participant sex, as this predictor was not present in the minimum adequate model selected by AIC (Table 1). The direction of the effects and statistical significance were not affected when the sexual preference of participants was included (S1 Table.).

Fig. 4. Perceived health estimates for Me'Phaa and European American faces within each color version. Estimates for the Natural versions (NAT), Light skin color transformed versions (LSCt), and Dark skin color transformed versions (DSCt) of the Me'Phaa (left) and European American (Right) faces. Error bars show 95% Confidence Intervals. Grey points and dashed lines indicate the random effect estimates for each Face ID within each color version.

Perceived dominance

Unlike the previous qualities, perceived dominance was lower for natural European American faces (*M* = 4.31, 95% CI [3.99 - 4.66], *z* = -3.63, *p* < 0.01) than Me'Phaa faces (*M* = 5.23, 95% CI [4.85 - 5.64], Table 2). Moreover, in contrast to perceived attractiveness, all faces received a lower dominance rating from male participants (*M* = 4.63, 95% CI [4.34 - 4.93], *z* = -2.24, *p* = 0.02) than from female participants (*M* = 4.88, 95% CI [4.61 - 5.16]). Finally, we were unable to determine any effect from lightening, darkening, or homogenizing the faces, since these predictors were not present in the minimum adequate model selected by AIC (Table 1). Statistical significance of these results did not change in the model that included all participants (S1 Table.).

Perceived aggressiveness

Although perceived aggressiveness was lower for the European American faces, the difference was not significant (*z* = -1.83, *p* = 0.06). Nevertheless, lightening the Me'Phaa faces made them seem less aggressive (*M* = 5.05, 95% CI [4.85 - 5.26], *z* = -2.76, *p* < 0.01) but darkening the European American faces also made them seem less aggressive (*M* = 4.56, 95% CI [4.37 - 4.77], *z* = 4.17, *p* < 0.01, Table 2). Finally, perceived aggressiveness did not change for either group when skin color and skin texture were homogenized (Me'Phaa faces *M* = 5.39, 95% CI [5.17 - 5.62]; *z* = -0.23, *p* = 0.82; European American faces *M* = 4.79, 95% CI [4.59 - 4.99]; *z* = 2.35, *p* = 0.17, Fig. 5.), and there was no effect of participant sex, as this predictor was not present in the minimum adequate model selected by AIC (Table 1). Finally, the direction of the effects and statistical significance were not affected when the sexual preference of participants was included (S1 Table.).

Fig. 5. Perceived aggressiveness estimates for Me'Phaa and European American faces within each color version. Estimates for the Natural versions (NAT), Light skin color transformed versions (LSCt), and Dark skin color transformed versions (DSCt) of the Me'Phaa (left) and European American (Right) faces. Error bars show 95% Confidence Intervals. Grey points and dashed lines indicate the random effect estimates for each Face ID within each color version.

Perceived masculinity

As with perceived dominance, perceived masculinity was lower for natural European American faces (*M* = 5.14, 95% CI [4.80 - 5.50], $z = -3.95$, $p < 0.01$ than for natural Me'Phaa faces (*M* = 6.18, 95% CI [5.79 - 6.60], Table 2). Moreover, lightening the Me'Phaa faces made them seem less masculine (*M* = 5.97, 95% CI [5.59 - 6.37], $z = -5.59$, $p < 0.01$), but darkening the European American faces had no effect (*M* = 4.93, 95% CI [4.61 - 5.27], $z = 1.20$, $p = 0.83$). In addition, perceived masculinity did not change for either group when skin color and skin texture were homogenized (DSCt Me'Phaa faces *M* = 5.97, 95% CI [5.59 - 6.37]; $z = 1.05$, $p = 0.90$; LSCt European American faces *M* = 4.83, 95% CI [4.53 - 5.15]; $z = 1.82$, $p = 0.45$, Fig. 6). Finally, there was no effect of participant sex, as this predictor was not present in the minimum adequate model selected by AIC (Table 1). The direction of the effects and statistical significance was not affected when the sexual preference of participants was included (S1 Table.).

Fig. 6. Perceived masculinity estimates for the Me'Phaa and European American faces within each color version. Estimates for the Natural versions (NAT), Light skin color transformed versions (LSCt), and Dark skin color transformed versions (DSCt) of the Me'Phaa (left) and European American (Right) faces. Error bars show 95% Confidence

Intervals. Grey points and dashed lines indicate the random effect estimates for each Face ID within each color version.

Discussion

Our findings suggest that facial skin pigmentation influences social judgments in a Mexican population. Based on our results, we could distinguish two groupings of social perceptions that facial skin pigmentation affects in opposite directions. In comparison with natural Me’Phaa faces, natural European American faces were perceived not only as more attractive, healthier, and more trustworthy but also as less dominant and less masculine. They were furthermore perceived as less aggressive, but not significantly so. Moreover, the perceptions grouped in these two dimensions correlate more within than between dimensions (S2 Table.). The apparent grouping of social perceptions found here resembles the valence-dominance model of facial perception proposed by Oosterhof and Todorov [70] and recently discussed by Jones et al. [6]. The valence-dominance model suggests that humans make impressions of others based on their facial appearance in the dimensions of valence and dominance. The valence dimension is related to facial judgments such as trustworthiness and attractiveness; hence, higher rates of this dimension could promote positive social interactions, for example mate opportunities. The dominance dimension, in turn, is related to perceptions such as aggressiveness, dominance, and masculinity, which could inform about the physical or psychological capability to cause harm; hence, higher values of this dimension could negatively affect social interactions in a modern context [8]. Here, we found that Mexican participants rated light-skinned faces more highly on all three

aspects of social valence (attractiveness, healthiness, trustworthiness). In contrast, they rated dark-skinned faces more highly on two aspects of social dominance (dominance, masculinity).

In the context of our first hypothesis, these results support the idea that sociocultural learning favors the function of facial skin pigmentation as a mediator of social perceptions. Our Mexican participants showed a robust positive association of pro-social judgments (i.e., attractiveness, trustworthiness, and perceived health) with light skin pigmentation. During the Mexican colonial period, light skin pigmentation was one of the most conspicuous phenotypic traits associated with a higher social status and access to different benefits, such as education and wealth [21]. Learning can reinforce a preference for a trait that may signal social benefits, even when the selected trait is not linked initially to any reproductive or survival benefit [19]. Consequently, a learned association between a physical trait—in this case, light skin—and social deference might establish a perception in a population over several generations. Furthermore, several studies have shown cultural preferences for light skin pigmentation and other European phenotypical traits and within-group discrimination based on skin color, a social phenomenon known as colorism [71]. Also, in Mexico and other Latin American countries, lightness of skin correlates with years of schooling, hourly earnings, occupational status, poverty, and social mobility [23–25]. Therefore, the association of pro-social attributes with light skin pigmentation we observed may be the result of a preference that emerged as an adaptative strategy to deal with biological and social pressures during the colonial period, which nowadays is reinforced by social learning and cultural mechanisms such as colorism, or the influence of Western societies on social media.

We also found that our participants rated the dark-skinned faces as more dominant and masculine. However, in contrast to our second prediction, dark skin color faces were not rated as more attractive or healthy. These results could indicate that skin pigmentation, as a trait mediated by androgens, could act as a mechanism to inform the viewer about the physical or psychological capability to cause harm by the bearer of the signal [72,73]; therefore, this signaling function might be relevant in sexual selection through male-male competition instead of female choice[74]. Similar results have been found in previous studies where androgen-dependent sexually dimorphic traits such as facial shape [75], voice pitch [76,77], or height [78], more strongly affect the perception of dominance, aggressiveness, or masculinity rather than attractiveness.

On the other hand, our experimental facial color interchange over 16 different faces allowed us to determine whether additional facial traits, such as shape, influence social perceptions in combination with skin color or even more than skin color. The human face embodies a set of a variety of signals and cues [79], and even though humans process faces in an integrative manner, this process follows a hierarchy of relevance in which different facial features are more important than others, depending on the perceptions evaluated [80,81]. In this way we found that some social perceptions were more affected by skin color than others. For example, lightening the Me'Phaa faces made them seem more attractive, healthier, and more trustworthy. Conversely, darkening the European American faces made them seem less attractive. Other perceived qualities, however, did not change systematically when facial skin color was changed. Perceived dominance did not change when changes were made to facial skin color within either group. Lightening the Me'Phaa faces decreased their perceived masculinity and aggressiveness, but darkening the

European American faces did not increase their perceived masculinity and aggressiveness. These results are consistent with studies on the role of skin color on certain perceptions, such as attractiveness [59,82], which show that skin color might contribute more than facial shape. Moreover, recent studies have shown that trustworthiness judgments rely on emotional expressions [8] or facial resemblance [83], and that dominance, masculine, and aggressiveness perception seem to rely more strongly on facial shape features [84–88]. Yet another possibility is that the skin color and facial shape effects on some of these perceptions interact in such a way that skin color becomes relevant only when the facial shape strongly elicits one of these perceptions. This may explain why the modification of skin color reduced the masculine and aggressiveness perception for the Me´Phaa faces, which were initially rated as more masculine and dominant, but it had no effect on the European American faces.

Another possibility is that there were systematic differences in the way that the experimental manipulation of skin color affected the European American and the Me´Phaa faces. In the statistical analysis we fitted random slopes for skin color transformation on each facial photograph ID to reduce the standard error caused by these differences, however, it is important to note that any manipulation process presents a risk of such artifacts. On the other hand, previous studies have shown that individuals are better at judging faces of their own ethnicity [35,89]. In this study our Mexican participants were asked to rate facial stimuli of a more closely related ethnic group (Me´Phaa faces), and stimuli from a more distant ethnic group (European American faces). Although 83% of our raters reported living in Mexico City and the surrounded area, hence the high probabilities of being exposed to European facial features due to immigration of people of European

ethnicity and access to internet and social media [90,91], these differences on the effects of the skin color manipulation between the European American and the Me´Phaa faces might be due to different degrees of familiarity with the facial features of the two ethnic groups.

Moreover, we not only lightened and darkened facial color but also homogenized it by removing such texture-related aspects as spots, freckles, or wrinkles [59,92,93]. Our results suggest that skin texture does have an effect, particularly on perceived health. That effect, however, largely exists independently of the effects of skin color. Skin texture might nonetheless interact with skin color in perceptions of light and dark skin, and such interactions could be an exciting avenue for further studies. In addition, unlike the authors of previous studies, we manipulated overall skin color instead of gradually changing each of its aspects (e.g., luminosity, hue) [33,35,46,53,60,82,94–97]. In our study, we aimed to test the differences in skin colors typically related to two different ethnic groups using a broader manipulation of skin color. Nonetheless, future studies could examine how each aspect of skin color contributes to the way a population is perceived.

In summary, we show that the Mexican population uses facial color to make certain social judgments. We found that a dark-skinned face is perceived as more dominant and more masculine. Those perceptions may be due to a process of coevolution between the human mind and the sex difference in skin color. One may therefore infer that male skin became darker through male-male competition rather than through female choice. We also found that a light-skinned face is perceived as more attractive, more trustworthy, and healthier. Those perceptions may be due to the association between skin color and social status that prevailed in Mexico during more than three centuries of colonial rule. Today, they are maintained by colorism. We are nonetheless speculating, and more data will be

needed. Similar results could be obtained from other Latin American societies, which share a common colonial history and social structure.

Acknowledgements

The authors are grateful to Juan Sebastían Lucero Carrasquilla for his help and assistance during the facial photograph acquisition of the Me'Phaa men sample.

References

1. Little AC, Jones BC, Debruine LM. Facial attractiveness: Evolutionary based research. Philosophical Transactions of the Royal Society B: Biological Sciences. 2011;366: 1638–1659. doi:10.1098/rstb.2010.0404
2. Puts DA, Jones BC, Debruine LM. Sexual selection on human faces and voices. J Sex Res. 2012;49: 227–243. doi:10.1080/00224499.2012.658924
3. Gonzalez-Santoyo I, Wheatley JR, Welling LLM, Cárdenas RA, Jimenez-Trejo F, Dawood K, et al. The face of female dominance: Women with dominant faces have lower cortisol. Horm Behav. 2015;71: 16–21. doi:10.1016/j.yhbeh.2015.03.006
4. Puts DA, Bailey DH, Cárdenas RA, Burriss RP, Welling LLM, Wheatley JR, et al. Women's attractiveness changes with estradiol and progesterone across the ovulatory cycle. Horm Behav. 2013;63: 13–19. doi:10.1016/j.yhbeh.2012.11.007
5. Ritchie KL, Palermo R, Rhodes G. Forming impressions of facial attractiveness is mandatory. Sci Rep. 2017;7: 1–8. doi:10.1038/s41598-017-00526-9
6. Jones BC, DeBruine LM, Flake JK, Liuzza MT, Antfolk J, Arinze NC, et al. To which world regions does the valence–dominance model of social perception apply? Nat Hum Behav. 2021;5: 159–169. doi:10.1038/s41562-020-01007-2
7. Olivola CY, Funk F, Todorov A. Social attributions from faces bias human choices. Trends Cogn Sci. 2014;18: 566–570. doi:10.1016/j.tics.2014.09.007
8. Todorov A, Olivola CY, Dotsch R, Mende-Siedlecki P. Social attributions from faces: Determinants, consequences, accuracy, and functional significance. Annu Rev Psychol. 2015;66: 519–545. doi:10.1146/annurev-psych-113011-143831

9. Barsh GS. What controls variation in human skin color? PLoS Biol. 2003;1: 19–23. doi:10.1371/journal.pbio.0000027

10. Samson N, Fink B, Matts PJ. Visible skin condition and perception of human facial appearance. Int J Cosmet Sci. 2010;32: 167–184. doi:10.1111/j.1468-2494.2009.00535.x

11. Edwards EA, Duntley SQ. THE PIGMENTS AND COLOR OF LIVING HUMAN SKIN. American Journal of Anathmy. 1939;65.

12. Darwin C. The Descent of Man, and Selection in Relation to Sex. J. Murray. London; 1871.

13. Jablonski NG, Chaplin G. The evolution of human skin coloration. J Hum Evol. 2000;39: 57–106. doi:10.1006/jhev.2000.0403

14. Adhikari K, Mendoza-Revilla J, Sohail A, Fuentes-Guajardo M, Lampert J, Chacón-Duque JC, et al. A GWAS in Latin Americans highlights the convergent evolution of lighter skin pigmentation in Eurasia. Nat Commun. 2019;10. doi:10.1038/s41467-018-08147-0

15. Deng L, Xu S. Adaptation of human skin color in various populations. Hereditas. 2018;155: 1. doi:10.1186/s41065-017-0036-2

16. Jablonski NG, Chaplin G. Human skin pigmentation as an adaptation to UV radiation. Proc Natl Acad Sci U S A. 2010;107: 8962–8968. doi:10.1073/pnas.0914628107

17. Feldman MW, Cavalli-Sforza LL. Cultural and biological evolutionary processes: Gene-culture disequilibrium. Isotopenpraxis. 1984;20: 1604–1607. doi:10.1073/pnas.81.5.1604

18. Verzijden MN, ten Cate C, Servedio MR, Kozak GM, Boughman JW, Svensson EI. The impact of learning on sexual selection and speciation. Trends Ecol Evol. 2012;27: 511–519. doi:10.1016/j.tree.2012.05.007

19. Little AC, Jones BC, Debruine LM, Caldwell CA. Social learning and human mate preferences: A potential mechanism for generating and maintaining between-population diversity in attraction. Philosophical Transactions of the Royal Society B: Biological Sciences. 2011;366: 366–375. doi:10.1098/rstb.2010.0192

20. Rendell L, Fogarty L, Hoppitt WJE, Morgan TJH, Webster MM, Laland KN. Cognitive culture: Theoretical and empirical insights into social learning strategies. Trends Cogn Sci. 2011;15: 68–76. doi:10.1016/j.tics.2010.12.002

21. Dixon AR, Telles EE. Skin color and colorism: Global research, concepts, and measurement. Annu Rev Sociol. 2017;43: 405–424. doi:10.1146/annurev-soc-060116-053315

22. Villarreal A. Stratification by skin color in contemporary Mexico. Am Sociol Rev. 2010;75: 652–678. doi:10.1177/0003122410378232

23. Telles E, Flores RD, Urrea-Giraldo F. Pigmentocracies: Educational inequality, skin color and census ethnoracial identification in eight Latin American countries. Res Soc Stratif Mobil. 2015;40: 39–58. doi:10.1016/j.rssm.2015.02.002

24. Campos-Vazquez RM, Medina-Cortina EM. Skin Color and Social Mobility: Evidence From Mexico. Demography. 2019;56: 321–343. doi:10.1007/s13524-018-0734-z

25. Flores R, Telles E. Social Stratification in Mexico: Disentangling Color, Ethnicity, and Class. Am Sociol Rev. 2012;77: 486–494. doi:10.1177/0003122412444720

26. van den Berghe PL, Frost P. Skin color preference, sexual dimorphism and sexual selection: A case of gene culture co-evolution? Ethn Racial Stud. 1986;9: 87–113. doi:10.1080/01419870.1986.9993516

27. Madrigal L, Kelly W. Human skin-color sexual dimorphism: A test of the sexual selection hypothesis. reply to frost (2007). Am J Phys Anthropol. 2007;133: 780–781. doi:10.1002/ajpa.20582

28. Aoki K. Sexual selection as a cause of human skin colour variation: Darwin’s hypothesis revisited. Ann Hum Biol. 2002;29: 589–608. doi:10.1080/03014460210000019144

29. Giacomoni PU, Mammone T, Teri M. Gender-linked differences in human skin. J Dermatol Sci. 2009;55: 144–149. doi:10.1016/j.jdermsci.2009.06.001

30. Edwards EA, Hamilton JB, Duntley SQ, Hubert G. CUTANEOUS VASCULAR AND PIGMENTARY CHANGES IN CASTRATE AND EUNUCHOID MEN. Endocrinology. 1941;28: 119–128.

31. Manning JT, Bundred PE, Mather FM. Second to fourth digit ratio, sexual selection, and skin colour. Evolution and Human Behavior. 2004;25: 38–50. doi:10.1016/S1090-5138(03)00082-5

32. Harvey RG. Ecological factors in skin color variation among Papua New Guineans. Am J Phys Anthropol. 1985;66: 407–416. doi:10.1002/ajpa.1330660409

33. Carrito M de L, Santos IMB dos, Lefevre CE, Whitehead RD, Silva CF da, Perrett DI. The role of sexually dimorphic skin colour and shape in attractiveness of male faces. Evolution and Human Behavior. 2016;37: 125–133. doi:10.1016/j.evolhumbehav.2015.09.006

34. Stephen ID, Coetzee V, Perrett DI. Carotenoid and melanin pigment coloration affect perceived human health. Evolution and Human Behavior. 2011;32: 216–227. doi:10.1016/j.evolhumbehav.2010.09.003

35. Coetzee V, Greeff JM, Stephen ID, Perrett DI. Cross-cultural agreement in facial attractiveness preferences: The role of ethnicity and gender. PLoS One. 2014;9. doi:10.1371/journal.pone.0099629

36. Frost P. Different Phases of the Menstrual Cycle : Preliminary assesment of evidence for a hormonal relationship. Percept Mot Skills. 1994;79: 507–514.

37. Folstad I, Karter AJ. Parasites, bright males, and the immunocompetence handicap. American Naturalist. 1992;139: 603–622. doi:10.1086/285346

38. Rantala MJ, Moore FR, Skrinda I, Krama T, Kivleniece I, Kecko S, et al. Evidence for the stress-linked immunocompetence handicap hypothesis in humans. Nat Commun. 2012;3. doi:10.1038/ncomms1696

39. Marcinkowska UM, Rantala MJ, Lee AJ, Kozlov M V., Aavik T, Cai H, et al. Women’s preferences for men’s facial masculinity are strongest under favorable ecological conditions. Sci Rep. 2019;9: 1–10. doi:10.1038/s41598-019-39350-8

40. Hagiwara N, Kashy DA, Cesario J. The independent effects of skin tone and facial features on Whites’ affective reactions to Blacks. J Exp Soc Psychol. 2012;48: 892–898. doi:10.1016/j.jesp.2012.02.001

41. Stepanova E V., Strube MJ. What’s in a face? the role of skin tone, facial physiognomy, and color presentation mode of facial primes in affective priming effects. Journal of Social Psychology. 2012;152: 212–227. doi:10.1080/00224545.2011.597797

42. Dunham Y, Stepanova E V., Dotsch R, Todorov A. The development of race-based perceptual categorization skin color dominates early category judgments. Dev Sci. 2015;18: 469–483. doi:10.1111/desc.12228

43. Jack RE, Crivelli C, Wheatley T. Data-Driven Methods to Diversify Knowledge of Human Psychology. Trends Cogn Sci. 2018;22: 1–5. doi:10.1016/j.tics.2017.10.002

44. Torres F, Salgado M, Mackenna B, Núñez J. Who differentiates by skin color? Status attributions and skin pigmentation in Chile. Front Psychol. 2019;10: 1–12. doi:10.3389/fpsyg.2019.01516

45. Oosterhof NN, Todorov A. The functional basis of face evaluation. Proc Natl Acad Sci U S A. 2008;105: 11087–11092. doi:10.1073/pnas.0805664105

46. Stephen ID, Law Smith MJ, Stirrat MR, Perrett DI. Facial skin coloration affects perceived health of human faces. Int J Primatol. 2009;30: 845–857. doi:10.1007/s10764-009-9380-z

47. Stephen ID, Oldham FH, Perrett DI, Barton RA. Redness enhances perceived aggression, dominance and attractiveness in men’s faces. Evolutionary Psychology. 2012;10: 562–572. doi:10.1177/147470491201000312

48. Borras-Guevara ML, Batres C, Perrett DI. Fear of Violence among Colombian Women Is Associated with Reduced Preferences for High-BMI Men. Human Nature. 2019;30: 341–369. doi:10.1007/s12110-019-09350-8

49. Carré JM, Archer J. Testosterone and human behavior: the role of individual and contextual variables. Curr Opin Psychol. 2018;19: 149–153. doi:10.1016/j.copsyc.2017.03.021

50. Geniole SN, Bird BM, McVittie JS, Purcell RB, Archer J, Carré JM. Is testosterone linked to human aggression? A meta-analytic examination of the relationship between baseline, dynamic, and manipulated testosterone on human aggression. Horm Behav. 2020;123: 104644. doi:10.1016/j.yhbeh.2019.104644

51. Puts DA, Welling LLM, Burriss RP, Dawood K. Men's masculinity and attractiveness predict their female partners' reported orgasm frequency and timing. Evolution and Human Behavior. 2012;33: 1–9. doi:10.1016/j.evolhumbehav.2011.03.003

52. Foo YZ, Rhodes G, Simmons LW. The carotenoid beta-carotene enhances facial color, attractiveness and perceived health, but not actual health, in humans. Behavioral Ecology. 2017;28: 570–578. doi:10.1093/beheco/arw188

53. Kleisner K, Kočnar T, Tureček P, Stella D, Akoko RM, Třebický V, et al. African and European perception of African female attractiveness. Evolution and Human Behavior. 2017;38: 744–755. doi:10.1016/j.evolhumbehav.2017.07.002

54. Alaluf S, Atkins D, Barrett K, Blount M, Carter N, Heath A. The impact of epidermal melanin on objective measurements of human skin colour. Pigment Cell Res. 2002;15: 119–126. doi:10.1034/j.1600-0749.2002.1o072.x

55. Visscher MO. Skin Color and Pigmentation in Ethnic Skin. Facial Plast Surg Clin North Am. 2017;25: 119–125. doi:10.1016/j.fsc.2016.08.011

56. Del Bino S, Bernerd F. Variations in skin colour and the biological consequences of ultraviolet radiation exposure. British Journal of Dermatology. 2013;169: 33–40. doi:10.1111/bjd.12529

57. Tiddeman B, Perrett D. Transformation of dynamic facial image sequences using static 2D prototypes. Visual Computer. 2002;18: 218–225. doi:10.1007/s003710100142

58. Coetzee V, Faerber SJ, Greeff JM, Lefevre CE, Re DE, Perrett DI. African Perceptions of Female Attractiveness. PLoS One. 2012;7: 3–8. doi:10.1371/journal.pone.0048116

59. Fink B, Grammer K, Matts PJ. Visible skin color distribution plays a role in the perception of age, attractiveness, and health in female faces. Evolution and Human Behavior. 2006;27: 433–442. doi:10.1016/j.evolhumbehav.2006.08.007

60. Lefevre CE, Perrett DI. Fruit over sunbed: Carotenoid skin colouration is found more attractive than melanin colouration. Quarterly Journal of Experimental Psychology. 2015;68: 284–293. doi:10.1080/17470218.2014.944194

61. Paschetta C, de Azevedo S, Ramallo V, Cintas C, Pérez O, Navarro P, et al. The impact of socioeconomic and phenotypic traits on self-perception of ethnicity in Latin America. Sci Rep. 2021;11: 1–11. doi:10.1038/s41598-021-92061-x

62. Telles EE. Pigmentocracies: Ethnicity, Race and Color in Latin America. North Carolina: UNC Press Book; 2014.

63. Bolker BM, Brooks ME, Clark CJ, Geange SW, Poulsen JR, Stevens MHH, et al. Generalized linear mixed models: a practical guide for ecology and evolution. Trends Ecol Evol. 2009;24: 127–135. doi:10.1016/j.tree.2008.10.008

64. Burnham KP, Anderson DR. Multimodel inference: Understanding AIC and BIC in model selection. Sociol Methods Res. 2004;33: 261–304. doi:10.1177/0049124104268644

65. Zuur AF, Ieno EN, Walker NJ, Saveliev AA, Smith GM. Mixed Effects Models and Extensions in Ecology with R. NewYork: Springer; 2009.

66. Zuur AF, Ieno EN. A protocol for conducting and presenting results of regression-type analyses. Methods Ecol Evol. 2016;7: 636–645. doi:10.1111/2041-210X.12577

67. Bates D, Mächler M, Bolker BM, Walker SC. Fitting linear mixed-effects models using lme4. J Stat Softw. 2015;67. doi:10.18637/jss.v067.i01

68. Bolker M Ben. Package ‘ bbmle .’ 2021.

69. R Core Team. R: A language and environment for statistical computing. Vienna, Austria: R Foundation for Statistical Computing.; 2020.

70. Oosterhof NN, Todorov A. The functional basis of face evaluation. Proc Natl Acad Sci U S A. 2008;105: 11087–11092. doi:10.1073/pnas.0805664105

71. Dixon AR. Colorism and classism confounded: Perceptions of discrimination in Latin America. Soc Sci Res. 2019;79: 32–55. doi:10.1016/j.ssresearch.2018.12.019

72. Kleisner K, Tureček P, Roberts SC, Havlíček J, Valentova JV, Akoko RM, et al. How and why patterns of sexual dimorphism in human faces vary across the world. Sci Rep. 2021;11: 1–14. doi:10.1038/s41598-021-85402-3

73. Puts DA. Human sexual selection. Curr Opin Psychol. 2016;7: 28–32. doi:10.1016/j.copsyc.2015.07.011

74. Puts DA. Beauty and the beast: Mechanisms of sexual selection in humans. Evolution and Human Behavior. 2010;31: 157–175. doi:10.1016/j.evolhumbehav.2010.02.005

75. Windhager S, Schaefer K, Fink B. Geometric morphometrics of male facial shape in relation to physical strength and perceived attractiveness, dominance, and masculinity. American Journal of Human Biology. 2011;23: 805–814. doi:10.1002/ajhb.21219

76. Puts DA, Apicella CL, Cárdenas RA. Masculine voices signal men’s threat potential in forager and industrial societies. Proceedings of the Royal Society B: Biological Sciences. 2012;279: 601–609. doi:10.1098/rspb.2011.0829

77. Rosenfield KA, Sorokowska A, Sorokowski P, Puts DA. Sexual selection for low male voice pitch among Amazonian forager-horticulturists. Evolution and Human Behavior. 2020;41: 3–11. doi:10.1016/j.evolhumbehav.2019.07.002

78. Batres C, Re DE, Perrett DI. Influence of Perceived Height, Masculinity, and Age on Each Other and on Perceptions of Dominance in Male Faces. Perception. 2015;44: 1293–1309. doi:10.1177/0301006615596898

79. Waller BM, Kavanagh E, Micheletta J, Clark PR, Whitehouse J. The face is central to primate multicomponent signals. Int J Primatol. 2022. doi:10.1007/s10764-021-00260-0

80. Karczmarek P, Pedrycz W, Kiersztyn A, Rutka P. A study in facial features saliency in face recognition: an analytic hierarchy process approach. Soft comput. 2017;21: 7503–7517. doi:10.1007/s00500-016-2305-9

81. Rotshtein P, Geng JJ, Driver J, Dolan RJ. Role of features and second-order spatial relations in face discrimination, face recognition, and individual face skills: Behavioral and functional magnetic resonance imaging data. J Cogn Neurosci. 2007;19: 1435–1452. doi:10.1162/jocn.2007.19.9.1435

82. Scott IML, Pound N, Stephen ID, Clark AP, Penton-Voak IS. Does masculinity matter? The contribution of masculine face shape to male attractiveness in humans. PLoS One. 2010;5. doi:10.1371/journal.pone.0013585

83. DeBruine LM. Trustworthy but not lust-worthy: Context-specific effects of facial resemblance. Proceedings of the Royal Society B: Biological Sciences. 2005;272: 919–922. doi:10.1098/rspb.2004.3003

84. Mogilski JK, Welling LLM. The relative contribution of jawbone and cheekbone prominence, eyebrow thickness, eye size, and face length to evaluations of facial masculinity and attractiveness: A conjoint data-driven approach. Front Psychol. 2018;9: 1–11. doi:10.3389/fpsyg.2018.02428

85. Torrance JS, Wincenciak J, Hahn AC, DeBruine LM, Jones BC. The relative contributions of facial shape and surface information to perceptions of attractiveness and dominance. PLoS One. 2014;9. doi:10.1371/journal.pone.0104415

86. Třebický V, Saribay SA, Kleisner K, Akoko RM, Kočnar T, Valentova JV, et al. Cross-Cultural Evidence for Apparent Racial Outgroup Advantage: Congruence between Perceived Facial Aggressiveness and Fighting Success. Sci Rep. 2018;8: 1–7. doi:10.1038/s41598-018-27751-0

87. Mitteroecker P, Windhager S, Müller GB, Schaefer K. The morphometrics of "masculinity" in human faces. PLoS One. 2015;10: 1–13. doi:10.1371/journal.pone.0118374

88. Windhager S, Schaefer K, Fink B. Geometric morphometrics of male facial shape in relation to physical strength and perceived attractiveness, dominance, and masculinity. American Journal of Human Biology. 2011;23: 805–814. doi:10.1002/ajhb.21219

89. Rhodes G, Lee K, Palermo R, Weiss M, Yoshikawa S, Clissa P, et al. Attractiveness of own-race, other-race, and mixed-race faces. Perception. 2005;34: 319–340. doi:10.1068/p5191

90. INEGI. ENCUESTA NACIONAL SOBRE DISPONIBILIDAD Y USO DE TECNOLOGÍAS DE LA INFORMACIÓN EN LOS HOGARES (ENDUTIH) 2021. Encuesta Nacional Sobre Disponibilidad Y Uso De Tecnologías De La Información En Los Hogares (Endutih) 20211. 2022; 1–19.

91. INEGI. Los extranjeros en México. XII Censo General de Población y Vivienda, 2000. 2014; 67.

92. Russell R. Sex, beauty, and the relative luminance of facial features. Perception. 2003;32: 1093–1107. doi:10.1068/p5101

93. Oh DW, Dotsch R, Todorov A. Contributions of shape and reflectance information to social judgments from faces. Vision Res. 2019;165: 131–142. doi:10.1016/j.visres.2019.10.010

94. Han C, Wang H, Hahn AC, Fisher CI, Kandrik M, Fasolt V, et al. Cultural differences in preferences for facial coloration. Evolution and Human Behavior. 2018;39: 154–159. doi:10.1016/j.evolhumbehav.2017.11.005

95. Stephen ID, Scott IML, Coetzee V, Pound N, Perrett DI, Penton-Voak IS. Cross-cultural effects of color, but not morphological masculinity, on perceived attractiveness of men's faces. Evolution and Human Behavior. 2012;33: 260–267. doi:10.1016/j.evolhumbehav.2011.10.003

96. Whitehead RD, Re D, Xiao D, Ozakinci G, Perrett DI. You are what you eat: Within-subject increases in fruit and vegetable consumption confer beneficial skin-color changes. PLoS One. 2012;7: 1–9. doi:10.1371/journal.pone.0032988

97. Whitehead RD, Ozakinci gözde, Perrett DI. Attractive skin coloration: Harnessing sexual selection to improve diet and health. Evolutionary Psychology. 2012;10: 842–854. doi:10.1177/147470491201000507

Supporting information

S1 Fig. Participant's self-perceived skin color ratings. PERLA scale distribution of the 700 participant's own skin color ratings. The color levels go from 1 to 10, being 1 the lightest and 10 the darkest. 11 (1.6 %) picked skin tone level 1, 36 (5.1%) skin tone level 2, 42 (6%) skin tone level 3, 240 (34.3%) skin tone level 4, 201 (28.8%) skin tone level 5, 102 (14.6%) skin tone level 6, 45 (6.4%) skin tone level 7, 14 (2%) skin tone level 8, 7 (1%) skin tone level 9, and 1 (0.1%) skin tone level 10.

S1 Table. Perception estimates with 95% confidence intervals with all participants included. Estimates (Est.) with 95% confidence intervals (CI) of the fixed predictors of the models for: (a) Perceived attractiveness, (b) Perceived trustworthiness, (c) Perceived health, (d) Perceived dominance, (e) Perceived aggressiveness, and (f) Perceived masculinity. *Note:* (□*)* The dominance best fitted model did not include the skin color transformation predictor, therefore the estimates shown represent the main effect of the natural skin color group only.

S2 Table. Pearson's correlation analysis between the scores of all perceptions rates of the natural versions only. Pearson's correlation between the natural versions scores of attractiveness, trustworthiness, perceived health, dominance, masculinity, and aggressiveness. *p<0.05, **p<0.01, ***p<0.001.

Response to reviewers

Effects of facial skin pigmentation on social judgments in a Mexican population

Jaaziel Martínez-Ramírez, David Puts , Javier Nieto, Isaac G-Santoyo*

*Corr. Author.

Reviewer 1: Edward Telles

-This is an important topic. The increasing interest and research on the topic is largely about the growing acknowledgment of the importance of race and color in Mexican society, by scientists and the general population. However, this analysis though seems to be largely of an evolutionary biological view with only a nod to ongoing racism and discrimination. Indeed, I could not find any use of “racism” but rather the use of “colorism.” When colorism is acknowledged, the authors usually refer to historical colorism under colonialization, which is learned across generations. As if racism and discrimination no longer exist. There is no consideration of the role of a Western ideology of race and racism and particularly, how Mexican elites, narratives, media, etc, shaped an ideology of mestizaje, which downplayed racism. See a considerable and growing literature by Patricio Solis, Regina Martinez Casas, Alan Knight and several others that shows the history of race in Mexico and how individuals use color to evaluate others in the labor market and other arenas. Indeed, the authors cite the PERLA studies but seem to overlook its findings about discrimination and inequality. Contrary to their claim that a social classification system based on phenotype no longer exists (p.5), these studies show that it is very profound in Mexican society, like it is throughout the Americas. I found it odd for, example, the claim that indigenous women chose to mate with Spanish men simply because they wanted to give their offspring better chances. What about the role of power or even rape?

Response:

We appreciate this comment. Indeed, our manuscript is in line with the perspective of the reviewer. The expression of various forms of racism in modern Mexican society is evident, leading to profound discrimination against individuals with physical attributes resembling pre-Columbian populations, with skin color being the most prominent attribute. Correspondingly, our study provides experimental evidence for the first time in a Mexican population that a relationship exists between "light" skin color and subjective preferences for favorable social traits such as attractiveness and reliability. Our manuscript aims to neither conceal nor distort this social effect; instead, it contributes to the extensive field of study in this area.
Based on the framework of behavioral ecology, social evolutionary psychology, and cultural evolution, our study aimed to test two non-mutually exclusive hypotheses explaining the effects of facial skin pigmentation on social judgments in the Mexican population. In the so-called social learning hypothesis, we proposed that this psychological

mechanism underlies the preferences for lighter skin color and its association with social benefits, prestige, or deference. This association originated during the colonial period through the racial/color hierarchy system known as "castas," which placed the European colonizers at the top of the social hierarchy and the indigenous population at the bottom. Therefore, we propose that skin color has led to the hierarchization of social benefits within the Mexican population through an extensive learning process that originated with the conquest. This process has influenced the ancestral evolutionary function of this physical attribute, from a mechanism of filtering UV radiation or synthesizing vitamin D to being an essential factor in social selection. In line with this, we do not deny that racism, colorism, and discrimination are also outcomes of this process and that nowadays, the cultural preferences for lighter skin color and other European phenotypical features may be reinforced by these sociological mechanisms. Our explanation from an ecological/evolutionary perspective does not contradict other authors from different fields of knowledge. Instead, it complements its study, not only due to the implementation of an experimental design that modifies skin color and controls for other confounding variables but also because of the theoretical argument that unifies the same observed phenomenon of racism through its observation in one of its facets related to subjective preferences determining different types of social interaction.

Additionally, although we did not explicitly use "racism," we considered "colorism" a more appropriate word to describe the effect observed regarding skin color, since we did not delve further into the implications of racism or other types of discrimination as it falls outside the scope of this study. Nonetheless, colorism, a form of prejudice or discrimination based on skin color, is evident, whereby individuals are treated differently based on this physical attribute, with lighter skin tones often being favored or perceived as more desirable than those with darker skin tones. Colorism can manifest both within racial and ethnic groups (as observed in our study) and between them, resulting in various negative impacts, such as reinforcing harmful stereotypes and creating disparities in education, employment, and social status. It is often interconnected with broader issues of racism and discrimination, making it essential to address in pursuing social justice and equality. Consequently, we have extensively rewritten a significant portion of the introduction and discussion sections to clarify our stance (for example see lines 113-131 and 502-513). . Moreover, we have expanded the information regarding previous studies demonstrating different racial features within the current Mexican population. Here, we have emphasized modifying the phrase "no longer exist" to more clearly convey that these preferences continue to permeate the psyche of the contemporary Mexican population, at least based on our evaluated sample (>700).

-The authors focus on the evaluations according to particular personality dimensions. They should consider the role of stereotypes as in how indigenous people are considered ugly - score low on attractiveness (note the recent reactions to the actress Yalitza Aparicio) and stereotypes about not being trusted and being sickly (two other dimensions studied). These findings are consistent with the use of ethnic/racial stereotypes. Aggressiveness may also be according to the idea of indigenous as savages. The dimensions of masculinity and dominance are less clear. Racism and stereotypes about indigenous people should certainly be considered at least as a hypothesis, which may compete with hypotheses about social

learning, etc. The color ratings of the European faces is also consistent with ratings as they may appear more indigenous.

Response: We appreciate this comment. Indeed, our results could be closely linked to racism and stereotypes; again, our intention has not been to deny these concepts but to complement them. In this line, we do not posit racism and stereotypes as a hypothesis competing with the hypothesis of social learning but rather as a possible consequence of this historical-social learning, which determines what is deemed "good" or "bad" based on skin color.

What is particularly interesting here is that the rater's participants had no information about the ethnicity of the presented stimuli. Therefore, we filtered out any information that could promote stereotypes by the evaluators beforehand, to observe the exclusive effect of skin color. In our experiment (please see the referred web page in line 268- labneuroecologiacog.com), we did not use labels or any other indication to suggest that the face belonged to a European American or a Me'Phaa man. Hence, if a rater had any stereotype bias towards Indigenous people, it would be harder for them to recognize the face as Indigenous. We also applied masks to all facial stimuli to minimize potential confounding factors.

Consequently, our results demonstrate a shift in participants' perceptions when skin color is oppositely modified to the original ethnic group. This change shows that a light-skinned face was evaluated positively on socially favorable dimensions (such as attractiveness and trustworthiness) and negatively on "unfavorable" dimensions. This effect was regardless of the stimuli' facial shape, the rater's preexisting stereotypical prejudices, the original ethnic group of the facial stimuli, or the rater's self-perception of skin color or ethnicity. We clarified this information in the discussion, also emphasizing that the approximations of racism and stereotypes evaluated here could be, in part, a consequence of this historical-social learning.

-As far as the analysis, I think it is mostly well done but I am surprised that the color or the ethnicity of the raters was not controlled (See Hill, Mark E. "Race of the interviewer and perception of skin color: Evidence from the multi-city study of urban inequality." American Sociological Review (2002): 99-108. The authors claim that the rater color was concentrated in a few colors and so they didn't control that. I don't understand why that should be important. Also, if raters are indigenous, they may evaluate in a different way. By the way, there seems to be a hesitancy to call the Me'Pha "indigenous." For these Mexican raters, I imagine stereotypes are based on indigeneity and they may have little or no familiarity with that particular ethnic group.

Response:

The data collected from self-reported ethnicities and PERLA scale responses indicated that 84% of the raters identified as Mestizos, and 78% chose levels 4-6 on the PERLA scale. The remaining 16% and 22% (respectively) were divided among four other ethnic groups and seven skin color levels. It's worth mentioning that some combinations had very low percentages, such as the self-reported ethnicity "Afro-American" at only 0.3%. This low percentage of other ethnic groups and scores on the PERLA scale suggests that our sample

is quite homogeneous. Here, variables with low representativeness, such as these two mentioned, lead to heterogeneity of variance due to the unbalanced data in the sample size of each predictor included (i.e., ethnicity with five levels and PERLA scale with 11 levels). This unbalanced data and over-parametrization are significant structural violations when using linear models (Zuur, Leno, & Elphick, 2010; Faraway, 2016). However, following the reviewer's observation, we conducted the method described by Zuur, Leno, and Elphick (2010) to investigate the possible effects of these two variables. After the model validation process, we plotted Pearson residuals of each selected model against participants' self-reported ethnicities and the PERLA scale responses for self-perceived skin color (the box plots can be found in the R Markdown, code lines 263-264 for the attractiveness plots, 455-456 for the dominance plots, 557-558 for the masculinity plots, 681-682 for the aggressiveness plots, 806-807 for the trustworthiness plots, and 936-937 for the perceived health plots). Due to the plots did not show any effect, and to avoid structural errors in the design of linear models, we did not include either of these two predictors. We briefly explained our decision in the statistical analysis section (lines 326-330).

Finally, following the reviewer's observation, we already had included self-perception of ethnicity as "Indigenous" rather than particular ethnic groups, such as "Me'Phaa"(Please see line 191).

-I would refer to raters as “raters” or “participant raters rather than “participants.”
Response: We changed the heading “Participants” with “Participant raters” in all manuscript.

References:
Faraway, J. J. (2016). *Extending the linear model with R: generalized linear, mixed effects and nonparametric regression models*. CRC press.

Zuur, A. F., Ieno, E. N., & Elphick, C. S. (2010). A protocol for data exploration to avoid common statistical problems. *Methods in ecology and evolution, 1*(1), 3-14.

Reviewer #2: Peter Frost

- “Its main difficulty is that of distinguishing between gendered and ethnic significations of skin color. The two have long coexisted in Mexican society. The authors have tried to control for ethnic significations by varying skin color within each ethnic group. The results of that strategy are mixed. It seems to me that ethnic significations contaminated the raters’ perceptions of the European American faces to a greater degree than they contaminated the raters’ perceptions of the Me’Phaa faces.”

Response:
We appreciate this precise observation. We propose two main reasons why we observed different results in the effects of skin color transformation between the European American and Me'Phaa faces, both closely related to the suggestions made by the reviewer.

The first possibility is that the participants may have rated the stimuli differently due to varying degrees of familiarity with the facial features of the two ethnic groups. Here, raters participants were asked to rate facial stimuli from a closely related ethnic group (Me'Phaa faces) and stimuli from a more distant ethnic group (European American faces). In this case, the effects on subjective perceptions were more evident when changing skin color within the more familiarized groups. However, although we found a similar effect on European American faces, it was not statistically significant in several social perceptions. The second possibility is attributed to a potential artifact in our method to modify the skin color. In this case, the manipulation process outcomes could have differed slightly between the European American and Me'Phaa faces. To deal with this, we minimized the standard error by fitting random slopes for skin color transformation for each facial photograph ID. However, it is essential to highlight that there is always a risk of such artifacts in any manipulation process. We added a new paragraph in the discussion summarizing these possible explanations and limitations (lines 549-562).

-There are a few errors:

- The reference to Green and Martin (1990) does not support footnote #27

- The adjective "perceived" should be used in all of the headings, and not just "perceived health." All of the qualities are perceived.

- Since perceived aggressiveness did not differ significantly between the two groups of faces, it should not be presented in the Discussion section as a perceived quality that differs between the two groups.

-In general, the wording is awkward. I would suggest the following corrections.
-Lines 83- 93 – replace the four sentences with: "The main pigments—melanin, hemoglobin, and carotenoids—absorb and reflect different wavelengths of light within the visible spectrum. Collagen also contributes by scattering light [10]. The skin's pigments vary not only between individuals and populations but also between men and women, perhaps because of sexual selection [11]. Natural selection initially favored darker skin as a means to protect against the harmful effects of UV radiation in the tropics. The pressure of selection then shifted toward lighter skin among those humans who spread into higher latitudes with lower levels of UV [12-14]"

-Footnote #10 – add:
Edwards, E.A., and S.Q. Duntley. (1939). The pigments and color of living human skin. American Journal of Anatomy 65(1): 1-33. https://doi.org/10.1002/aja.1000650102

-Line 102 – replace "others" with "other"

-Line 110 – replace "preference honestly contributed to reproductive outcomes" with: "preference significantly contributes to reproductive success"

-Lines 128-140 – replace with: "The sex difference is due to exposure of skin tissues to differing ratios of androgens to estrogens, particularly at puberty. Testosterone has a

stronger effect than estrogen on melanin synthesis and vascularization of the upper dermis [26].”

-Footnote #26 - add:

Edwards EA, Hamilton JB, Duntley SQ, Hubert G. Cutaneous vascular and pigmentary changes in castrate and eunuchoid men. Endocrinology 1941; 28(1): 119-128. doi:10.1210/endo-28-1-119

Manning JT, Bundred PE, Mather FM. Second to fourth digit ratio, sexual selection, and skin colour. Evolution and Human Behavior 2004; 25(1): 38-50. doi:10.1016/s1090-5138(03)00082-5

-Footnote #27 - the reference to Green and Martin (1990) does not support the statement that “males undergo a more intense facultative pigmentation after sun exposure and retain it for longer periods than females whereas female skin lightens faster after a reduction in sun exposure.”

Replace the reference to Green and Martin (1990) with:

Harvey RG. Ecological factors in skin color variation among Papua New Guineans, American Journal of Physical Anthropology 1985; 66(4): 407-416. doi:10.1002/ajpa.1330660409

-Footnotes #28-30 - add:

Frost P. Preference for darker faces in photographs at different phases of the menstrual cycle: Preliminary assessment of evidence for a hormonal relationship. Perceptual and Motor Skills 1994; 79(1): 507-14. doi:10.2466/pms.1994.79.1.507

Line 140 – replace “have” with “has”

-Line 148 – replace “no-WEIRD” with “non-WEIRD”

-Lines 151-152 – replace with: “In this study, we used an experimental design with a Mexican population to determine whether facial skin color can influence certain social perceptions: attractiveness, …”

-Line 155 – replace “To accomplish this” with “To that end”

-Line 164 – delete the extra comma after “perceptions”

-Line 193 – replace “create” with “created”

-Line 240 – replace “into” with “within”

-Line 253 – replace “prevent” with “made sure”

-Line 254 – insert “not” after “were”

-Line 256 – replace “on” with “of”

-Lines 259-261 – replace with: “… version. In other words, we compared natural European American faces with artificial light-skinned and dark-skinned versions and natural Me’Phaa faces with artificial light-skinned and dark-skinned versions. We could thus measure how the participants perceived differences in facial color independently of the face’s ethnicity.”

-Line 264 – replace “in” with “on”

- 272 – replace “men participants” with “the male participants”

-Line 281 – replace “be” with “have been”

-Line 285 – replace “in” with “on the”

-Line 329 – replace “Attractiveness” with “Perceived attractiveness”

-Line 330 – replace with: “Perceived attractiveness differed significantly between the two groups when they had their natural skin color”

-Line 331 – replace “as” with “more”

-Line 332 – replace “as” with “than”

-Lines 332-333 – replace with: “Furthermore, there were effects when natural skin color was changed within each of the two groups. Lightening the Me’Phaa faces made them more attractive (…), and darkening the European American faces made them less attractive (…). Both groups became slightly more attractive when skin color and skin texture were homogenized (…).

-Lines 343-344 – replace “men participants” with “the male participants”

-Lines 344-345 – replace “women participants” with “did the female participants”

-Table 2 - replace “Models estimates” with “Perception estimates”
- Replace “Attractiveness perception” with “Perceived attractiveness”
- Replace “Trustworthiness perception” with “Perceived trustworthiness”
- Replace “Dominance perception” with “Perceived dominance”
- Replace “Aggressiveness perception” with “Perceived aggressiveness”
- Replace “Masculinity perception” with “Perceived masculinity”

-Line 357 – replace “Trustworthiness” with “Perceived trustworthiness”

-Line 359 – replace “as” with “more”

-Line 360 – replace “as” with “than”

-Line 361-363 – replace with: “In addition, lightening the Me’Phaa faces increased their perceived trustworthiness (…), whereas darkening the European American faces had no effect”

-Line 383 – replace “Perceived health” with “Perceived healthiness”

-Line 384 – replace “As in” with “As with”

-Lines 386-394 – replace with: “On average, natural European American faces were perceived as being 1.19 times healthier (…) than natural Me’Phaa faces (…). When facial skin color was changed within each ethnic group, the changes had different effects on perceptions of the two groups. Lightening the Me’Phaa faces increased their perceived health (…), whereas darkening the European American faces had no effect (…). Perceived health increased slightly for both groups when skin color and skin texture were homogenized.

-Line 409 – replace “Dominance” with “Perceived dominance”

-Lines 410-411 – replace with: “Unlike the previous qualities, perceived dominance was lower for European American faces that had their natural skin color”

-Lines 413-416 – replace with: “in contrast to perceived attractiveness, all faces received a lower dominance rating from male participants (…) than from female participants (…). Finally, we were unable to determine any effect from lightening, darkening, or homogenizing the faces, since these predictors …”

-Line 420 – replace “Aggressiveness” with “Perceived aggressiveness”

-Lines 421- 427 – replace with: “Although perceived aggressiveness was lower for the European American faces, the difference was not significant (…). Lightening the Me’Phaa faces made them seem less aggressive (…), but darkening the European American faces also made them seem less aggressive (…). Finally, perceived aggressiveness did not change for either group when skin color and skin texture were homogenized (…)”

-Line 442 – replace “Masculinity” with “Perceived masculinity”

-Lines 443-450 – “As with perceived dominance, perceived masculinity was lower for natural European American faces (…) than for natural Me’Phaa faces (…). Lightening the Me’Phaa faces made them seem less masculine (…), but darkening the European American faces had no effect (…). In addition, perceived masculinity did not change for either group when skin color and skin texture were homogenized (…).”

-Lines 469-472 (sentence) – replace with: “In comparison with natural Me’Phaa faces, natural European American faces were perceived not only as more attractive, healthier, and

more trustworthy but also as less dominant and less masculine. They were furthermore perceived as less aggressive, but not significantly so.”

-Lines 484-486 – replace with: “… Mexican participants rated light-skinned faces more highly on all three aspects of social valence (attractiveness, healthiness, trustworthiness). In contrast, they rated dark-skinned faces more highly on two aspects of social dominance (dominance, masculinity).

-Line 493 – replace “well-remunerated” with “well-paid”

-Lines 495-498 (sentence) – “Consequently, a learned association between a physical trait—in this case, light skin—and social deference might establish such a perception in a population over several generations”

-Line 498 – replace “learning association” with “learned association”

-Lines 505-507 (sentence) – replace with: “Also, in Mexico and other Latin American countries, lightness of skin correlates with years of schooling, hourly earnings, and other indicators of well-being.”

-Line 511 – replace “dark skin color faces” with “dark-skinned faces”

-Lines 512-513 - replace “dark skin color faces” with “dark-skinned faces”

-Line 516 – replace the comma before “therefore” with a semi-colon

-Line 518 – insert “sexually” before “dimorphic”

-Lines 528-535 – Replace with: “For example, lightening the Me’Phaa faces made them seem more attractive, healthier, and more trustworthy. Conversely, darkening the European American faces made them seem less attractive. Other perceived qualities, however, did not change systematically when facial skin color was changed. Perceived dominance did not change when changes were made to facial skin color within either group. Lightening the Me’Phaa faces decreased their perceived masculinity and aggressiveness, but darkening the European American faces did not increase their perceived masculinity and aggressiveness.”

-Lines 541-557 - Replace with: “Moreover, we not only lightened and darkened facial color but also homogenized it by removing such texture-related aspects as spots, freckles, or wrinkles […]. Our results suggest that skin texture does have an effect, particularly on perceived health. That effect, however, largely exists independently of the effects of skin color. Skin texture might nonetheless interact with skin color in perceptions of light and dark skin, and such interactions could be an exciting avenue for further studies. In addition, unlike the authors of previous studies, we manipulated overall skin color instead of gradually changing each of its aspects (e.g., luminosity, hue) […]. Future studies could examine how each aspect of skin color contributes to the way a population is perceived.”

-Lines 558-574 - replace with: “In summary, we show that the Mexican population uses

facial color to make certain social judgments. We found that a dark-skinned face is perceived as more dominant and more masculine. Those perceptions may be due to a process of coevolution between the human mind and the sex difference in skin color. One may therefore infer that male skin became darker through male-male competition rather than through female choice. We also found that a light-skinned face is perceived as more attractive, more trustworthy, and healthier. Those perceptions may be due to the association between skin color and social status that prevailed in Mexico during more than three centuries of colonial rule. Today, they are maintained by colorism. We are nonetheless speculating, and more data will be needed. Similar results could be obtained from other Latin American societies, which share a common colonial history and social structure."

Response:

We greatly appreciate the reviewer's effort and time in providing us with precise suggestions, which were timely and significantly improved our manuscript. Therefore, we have rewritten all sentences and added all recommended references as per the reviewer's assessment. Once again, thank you very much.

Reviewer #3: Vojtech Fiala

-The overall positive impression is also promoted by detailed report on the fitted models, availability of the script and data. It seems like a good craft (although I must admit that I have not checked if the 'necessary' rituals of frequentist statistics have been gone through by the authors). While I recommend to study for acceptance (this being their final destiny), I still find some aspects of it problematic – and I think these should be considered at least by extending/re-writing some paragraphs within this otherwise good manuscript.

-Major objection 1: Why Poisson?

-First, I wonder, why the authors decided to use generalised linear model with the family set to "Poisson". The expectation that the dataset (data of the dependent variable) comes from Poisson distribution is not commented on in the manuscript. Picking 1-9 points is, in my opinion, not a count of rare events within interval (of whatever kind; how the parameters of such a distribution may be set?). While this objection may seem serious, it is not the case (given my expectations about the "intestines" of glm fit that are above the scope of the current text). Nonetheless, the authors should either justify their decision (maybe I am just wrong, and I'd appreciate being proven so, since this may positively affect my own subsequent work) or consider using different distribution for the dependent variable's population, which has been sampled.

Response:

We appreciate this observation. We decided to use GLMMs with Poisson errors due to the nature of our data, which are discrete quantitative data derived from a Likert scale. We did not average the perception data (i.e. among participant scores for each face rated) because we consider that this data treatment could affect the measures of the central tendency of

some facial perceptions; this was particularly true for the attractiveness rating distribution, where we observed a positively skewed counts-type distribution (the histograms can be found in the R Markdown, code lines 234-238). Because of this and the discrete nature of our dependent variables, we decided to use Poisson GLMMs instead of Gaussians, which we explained briefly in the statistical analysis section (lines 302-307).

Major objection 2: Culture vs… and biology vs… (?)

Another serious notion that shall be considered during revisions, refer to the lines 163-173 of the manuscript. I put aside that the sentences are long and hard to follow. I have the following concerns: The authors begin by stating that lighter skin that is naturally associated with a given ethnicity may be a cue to favorable characteristic (as they are stereotypically ascribed to a face, not to its colouration itself – colour is cue to ethnicity, which affect the ascribed characteristics). However, the opposite hypothesis would read: Maybe, it does not matter if the face is European/Native American (I mean, the origin of the face) and what matters is lightness (as an exact measure). This contrasting view (while I acknowledge cannot be directly addressed in this study) is not applied here. The authors rather switch from cultural to biological explanation: once the face is darker, it is perceived as more attractive, also dominant, aggressive, etc., due to androgens. Together, this seems for me to not make much sense. Consider rewriting this paragraph (even if it is just to help slow-minded readers, which I eventually may be, to understand).

Response:

Thank you for this observation. In this study, we tested three hypotheses, each of them having its null and alternative prediction. These three hypotheses could be mutually non-exclusive and, therefore, complement each other. In the first one we propose that social learning may be the psychological mechanism underlying the effects of facial skin pigmentation on the social perceptions. Here we sought to test whether light facial color (and the opposite for dark facial color) promotes greater rate values in favorable social attributes, such as reliability, attractiveness or perceived health. This effect would be independent of the of the origin of the face, but only by the skin coloration. In our second hypothesis, we propose that these effects may rather be the results of ancestral sexual selection of an androgen-dependent trait such as skin color. Here, darker faces and not lighter would influence greater rates values in social perceptions such as attractiveness, aggressiveness, masculinity and dominance. If this is supported, we would expect that the experimental modification of the skin pigmentation would change the previous perceptions in both the European American and Me´Phaa faces. This means that, as noticed in the review, the study seeks to determine the role of skin color controlling for other facial features like shape. Finally, our third hypothesis suggests that neither lighter nor darker skin color would affect any social perception, neither in the original faces nor in stimuli with exchanged colors
We have rewritten this paragraph to make our hypothesis clearer (lines 170-186).

Minor notes:

-"There was no effect of the skin color manipulation or color exchange [for the perceived dominance] in any group."

This is a surprising result, given the previously identified positive association between darker skin and dominance-related characteristics like masculinity. I suggest the authors to add a paragraph in the discussion in which they comment on the result. Consider the opposite result for perceived aggressiveness, which was not significantly affected by the ethnicity in non-manipulated faces, while it was affected by skin colour manipulation in both the groups in the anticipated direction (the same applies for masculinity).

Response: As suggested by Torrance et al. (2014) it seems that the facial shape and surface features, such as skin color, contribute differently to judgements of attractiveness and dominance (See Torrance JS, Wincenciak J, Hahn AC, DeBruine LM, Jones BC (2014) The Relative Contributions of Facial Shape and Surface Information to Perceptions of Attractiveness and Dominance. PLoS ONE 9(10): e104415. doi: 10.1371/journal.pone.0104415). They found that the perception of dominance was more strongly affected by facial shape features whereas the opposite was observed for the perception of attractiveness. Yet another possibility is that the skin color and facial shape effects on some of these perceptions interact in such a way that skin color becomes relevant only when the facial shape strongly elicits one of these perceptions. This may be the case for what we observed in the perceptions of masculinity and aggressiveness, since the skin color manipulation affected mainly the Me´Phaa faces which, in turn, were the ones rated as more dominant and masculine. That would mean that skin color cues become relevant for the perception of masculine and aggressiveness only on faces with shapes that strongly elicit these perceptions. We have rewritten a paragraph in the discussion where we summarized this idea (lines 531-548).
