# Supplementary material for: Effects of facial skin pigmentation on social judgments in a Mexican population
Source: PLoS One. 2023 Nov 30;18(11):e0279858. doi: 10.1371/journal.pone.0279858 (PMC10688750; doi:10.1371/journal.pone.0279858)
Supplement: Attachment — Submitted filename: PONE-D-22-34514_R2_reviewerPF.pdf [file pone.0279858.s008.pdf]

PLOS ONE

Effects of facial skin pigmentation on social judgments in a Mexican population

--Manuscript Draft--

**Manuscript Number:**	PONE-D-22-34514R2
**Article Type:**	Research Article
**Full Title:**	Effects of facial skin pigmentation on social judgments in a Mexican population
**Short Title:**	Effects of facial skin pigmentation on social judgments in a Mexican population
**Corresponding Author:**	Isaac G-Santoyo<br>Universidad Nacional Autonoma de Mexico Facultad de Psicologia<br>CDMX, CDMX MEXICO
**Keywords:**	Skin Color Pigmentation, Colorism, Mexican social perceptions, social learning, sexually dimorphic traits, Skin color social and biological evolution
**Abstract:**	People quickly and involuntarily form impressions of others based on their facial physical attributes, which can modulate critical social interactions. Skin pigmentation is one of the most variable and conspicuous facial traits among human populations. Empirical evidence suggests that these variations reflect ancestral ecological selective pressures balancing cutaneous vitamin D synthesis with the protection of the dermis from ultraviolet radiation. Nevertheless, skin pigmentation may currently be subject to additional selective pressures. For instance, the colonial era in Central and South America developed a highly stratified society based on ethnic origins, and light skin pigmentation became associated with higher social status and deference. This association could have originated through historical social learning that promoted favorable social perceptions towards individuals with lighter skin color and unfavorable perceptions towards individuals with darker skin color, which could still be present in the perception of current populations.<br>Facial skin pigmentation is also sexually dimorphic, with males tending to exhibit darker skin than females, a difference that could be driven by sexual selection. To explore whether social learning and sexual selection represent additional selective pressures on skin pigmentation, we tested how this facial trait influences fundamental social perceptions in a Mexican population (N = 700, 489 female). We sampled facial images of eight European American males with natural lighter facial skin and eight males from an indigenous pre-Columbian community from Mexico, the Me'Phaa, with natural darker facial skin. We produced stimuli from these images by varying the skin pigmentation while preserving the facial shape. Stimuli were rated on attractiveness, trustworthiness, perceived health, dominance, aggressiveness, and femininity/masculinity. We found that the natural light-skinned faces were perceived as more attractive, trustworthy, and healthy but less dominant than the natural dark faces. Furthermore, by varying the facial skin color in these original groups, we altered the perceptions of them, mainly their attractiveness. These results partially support the hypothesis that dark facial skin color may help males compete for mates. Also, the results strongly support the view that lighter facial skin color became associated with social benefits through social learning in this Mexican population. Our findings, when viewed through the lens of cultural evolution, align with previous research in social psychology and anthropology. They hold the potential to offer a comprehensive understanding of the origin of this social phenomenon of cultural transmission, which currently plays a role in the formation of racial attitudes, stereotyping, and racial inequality in Mexican and other Latin American populations.
**Order of Authors:**	Jaaziel Martínez-Ramírez
	David Puts
	Javier Nieto
	Isaac G-Santoyo
**Response to Reviewers:**	Reviewer 1:<br><br>Comment:<br>This revision is an improvement but there are still two problems. The first is correctable

Powered by Editorial Manager® and ProduXion Manager® from Aries Systems Corporation

and I am not sure about the second:
1. The study focuses on the evolutionary role of skin color in Mexican society. However, they hardly engage with the metaphorical “elephant in the room:” current societal skin color inequality and discrimination. While the literature review acknowledges societal inequalities based on skin color, mostly in the social psychology and sociological literature, the authors do not discuss how it fits with their own findings. Except for mentioning this literature (not until pages 5-6), there is no serious engagement. How do their finding co-exist with those of racial attitudes, stereotyping and racial inequality? The abstract, for example, does not even mention that. I expect that readers would want to know how these evolutionary findings may complement (or not) the social science findings. For example, I see complementarity in how the findings are consistent with stereotypes of indigenous people.

Response:
We appreciate the previous comments from the reviewer; they have been instrumental in enhancing the manuscript's writing. Furthermore, we consider that these two recent observations provide valuable information to enhance the emphasis we have aimed about inequality and social discrimination based on skin stereotypes, and on how our evolutionary framework and results complement (or not) the social science findings. We strongly agree that our results are consistent with previous studies in the social sciences regarding stereotypes on indigenous people. Additionally, we do not deny the current existence of societal skin color inequality and discrimination in the studied Mexican population. In fact, we propose that these behaviors are the result of these historical social pressures and cultural transmission.
Finally, the deliberate choice for alluding these concepts up until page 4 was because the initial segment of our introduction aimed to elucidate the physiological underpinnings of skin color evolution within the human species. It's important to note that this emphasis was not indicative of a lack of recognition or engagement with the prevailing issues of contemporary societal disparities related to skin color. To be more precise with these assertions, we have expanded the information in introduction (lines 101-120) and discussion (lines 569-585). In addition, we have modified the abstract to more clearly incorporate these aspects mentioned above (Lines 60-67).

Comment:
2. My other comment is on the methods, which have implications for the substantive interpretations. Reviewers 2 and 3 also seem to have alluded to this. The authors claim that there is no information about the ethnicity of the stimuli subjects but that in one treatment, only skin color and not facial features, was changed. But skin color is the information! Darker skin color in Mexico suggests that persons are more indigenous. Note that skin color, like indigeneity, is fluid and on a continuum, especially between mestizos and indigenous people (see for example Villarreal 2013 in the American Sociological Review and Flores et al 2023 in the American Journal of Sociology).

Response:
Thank you for this insightful observation. Indeed, this aspect holds pivotal significance for our proposition. Precisely, the aim of this study was to ascertain whether skin color serves as a conduit of information influencing social perceptions. In our study we supported this premise experimentally by altering skin color according to the facial color patterns of these two racial groups. Consequently, we propose that skin color can be employed to associate ethnicity with its learned social stereotypes, and this interplay could potentially underpin social perceptions. These two elements, coloration and stereotype, would not be intertwined without a long and robust process of cultural learning and transmission that facilitates the stereotyping of a racial group based on a distinguishing phenotypic trait (i.e., skin color). In fact, this forms the bedrock of our proposition concerning cultural transmission and social learning in the evolution of human culture. We elucidate and expand upon this information in lines 484-492.
Aligned with this notion, what holds significance here is that the color-ethnicity-perception nexus has evolved as a consequence of learned cultural pressures that have delineated what is socially favorable (or unfavorable) based on an evaluation of skin color—a physical attribute rooted in a distinct evolutionary origin (i.e., environmental pressures due to UV radiation). In contemporary times, this attribute is also harnessed for categorization, stereotyping, and modulation of social perceptions. This historical social pressure revolving around skin color could furthermore give rise to

Powered by Editorial Manager® and ProduXion Manager® from Aries Systems Corporation

components of discrimination and racism within modern Mexican society. These intricate connections have been deeply interwoven throughout the history of Latin America and disentangling them proves challenging when attempting to elucidate their compounded effects. We clarify and expand upon this information in lines 569-585. Lastly, while acknowledging that disentangling racial stereotypes from skin color presents a formidable challenge, we believe that our study could serve as useful reference for future endeavors in social sciences that delve into this issue with the necessary depth. We do encourage this vision in the paragraph previously reported (lines 569-585). We also included a paragraph to explain that skin color and ethnicity form a continuous gradient, rather than a categorical distinction as implemented in the current study. Therefore, future research is needed to ascertain the extent to which these social perceptions are influenced by the fluid continuum of these two dimensions. Please see lines 536-548.

Reviewer #2:

Comment:
The manuscript could be better written. I have attached a corrected version of the PDF (see pages 16 to 51). Otherwise, I have no issues with the revised manuscript.

Response:
We greatly appreciate the reviewer's effort and time in providing us with precise suggestions, which will significantly improve our manuscript. Therefore, we have rewritten all sentences. Once again, thank you very much.

Reviewer #4:

Comment:
1.It’s a bit surprising that Lightness (L*) is the only parameter considered in this study whereas the aim is to investigate the influence of skin pigmentation on social judgments.
Obviously, the reason given in line 222 (lightness has a negative correlation with melanin) is not rational. In fact, melanin pigmentation is not only negatively affects the skin’s lightness, but possibly even more strongly and positively linked to skin yellowness. And it also influences skin redness. See for example the below reference. There are also other skin pigmentations. If it’s only lightness (L*) is investigated, the title and the scope should be restricted to the effect of ‘skin lightness’ not ‘skin pigmentation‘, on facial impressions. Otherwise, it’s not fair to study the influence of skin colour on facial impressions only based on L* values.
“Fruit over sunbed: Carotenoid skin colouration is found more attractive than melanin colouration,” Q. J. Exp. Psychol., vol. 68, no. 2, pp. 284–293, Feb. 2015, doi: 10.1080/17470218.2014.944194.
Kikuchi, Kumiko, et al. "Image analysis of skin colour heterogeneity focusing on skin chromophores and the age-related changes in facial skin." Skin Research and Technology 21.2 (2015): 175-183.

Response:
Thank you for this comment. Skin pigmentation arises from a complex interplay of three primary pigments: melanin, hemoglobin, and carotenoids, along with other components like collagen. In our study, we focused on the dimension of skin pigmentation linked to melanin, which spans from light to dark, given its salience and significant variation among human populations. Our method involved creating prototypes based on the average skin color values of each group and then using the differences between these prototypes as a "mask" to generate the two skin color versions. Consequently, because our manipulation was based on broad average values, the resulting stimuli exhibited variations not solely in the lightness parameter but across all dimensions; in essence, since the skin color dimension we focused on is inherently embedded within the broader context of "skin pigmentation", and given that we didn't exclusively modify lightness while controlling over other parameters, we chose to use the term "skin pigmentation" instead of "skin lightness."
On the other hand, we need to make it clear that we calculated the CIELab values to determine if the skin color in each of the two groups (natural lighter from European American faces, and natural darker from Me´phaa faces) had observable and

Powered by Editorial Manager® and ProduXion Manager® from Aries Systems Corporation

contrasting color properties. Here, we found that color properties effectively differed between groups. However, we chose lightness as it was the color parameter that contrasted the most in our groups and it could also influence other properties (like yellowness). We explained this in the manuscript, see lines 203-2010, and in 213-217. Now, the statement we made in line 222(i.e., previous version line) is well-founded. Alaluf et al. (2002) compared measurements of human skin color (CIELab parameters) with biochemical measurements of melanin content, composition, and melanosome size in skin biopsies. They discovered a robust negative correlation between lightness and total melanin content, as well as a weaker yet significant negative correlation between lightness and melanosome size. Although a positive correlation between yellowness and total melanin content was identified, it appeared to be influenced by lightness. Additionally, no other substantial correlations were found between yellowness and the other biological measurements. Given these considerations, we believe that this parameter best represents the dimension we focused on in our objectives. Nevertheless, for a better understanding of the use of this parameter in our study, we removed the association of this parameter with the biochemical relationships with melanin, since, in line with the reviewer, other color parameters could be affected by this pigment, and because determining the specific relations between the three parameters of color and the social perceptions is just out of the scope of this study (Please see lines 212-217). Having said this, we also believe that investigating how each facet of skin color contributes to the perception of this population could open up intriguing avenues for further research. This notion is elaborated upon in the discussion (Line 543-548).

Comment:
2.The creation of facial stimuli is unusual. Two averaged faces were created first and then the selected eight facial stimuli were either added or subtracted from the averaged face. Is there any reason for doing that? It is also not clear how the skin colour manipulation was done (lines 233-242). As the selected eight faces have different face shapes/ features/ colours/ colour distributions from the prototype, how the colour was added or subtracted without holding the facial shape unchanged? Whether the colour change was also applied to the facial features (eyes, mouth, brows). Any process for those facial features? Why was 70 per cent particularly selected? What software was used? It would be helpful to specify the detailed process.

Response:
We appreciate the comment. The software we used was Psychomorph and we did specify this information in the section named “Skin color facial stimuli” (now lines 218-220). Psychomorph has been used in numerous studies on face perception since it allows the independent manipulation of shape and color properties in facial photographs. To conduct the manipulations, the initial step involves outlining the facial contours of all photographs. Subsequently, two prototypes are fashioned, each representing the averaged values of the extreme points corresponding to the facial feature targeted for manipulation. Finally, using these two prototypes as reference, it is possible to create different versions of the same stimuli varying the facial feature based on the differences between the two prototypes (for a deeper understanding about how the software works and its details, we strongly recommend entering the following link: https://users.aber.ac.uk/bpt/jpsychomorph/).

Now, the sum or subtraction was made applying in each face the two color “mask” of the color prototypes. This was previously described and clarified in the section “Skin color facial stimuli” (Lines 218-239). Moreover, the setting of 70% of difference was used as a rule of thumb that the software developers recommend in order to avoid visual aberrations. This percentage not only prevents these visual aberrations to happened, but also ensures a significant change in color properties among the stimuli within the observable and feasible ranges for each ethnic group.
Finally, just like the unintended effect that occurred in texture homogenization because of the methodology we employed, a similar effect was observed in the color transformation, which extended to the eyes, mouth, and eyebrows. Our strategy to address this side effect involved comparing the ratings of the original versions with those of the corresponding versions with transformed skin color. This helped us measure the impact of the color homogenization effect. Nevertheless, we clarified this limitation in lines 531-548

Powered by Editorial Manager® and ProduXion Manager® from Aries Systems Corporation

Comment:
3.Apart from lightness (L*), the facial stimuli seem to show a large difference in yellowness (b*). Is that true? Since authors already 'measured its skin colour values in CIELab colour space (line 218)', it would be necessary to show the colour parameters in CLELAB colour space (including L*, a*, and b*) for all the face stimuli from both European American and Me'phaa groups, not just L*.

Response:
We appreciate this comment. As mentioned earlier, our calculation of the CIELab values primarily served as a validation method for selecting faces and for our manipulation process. In the response to comment 1 you can find all the information regarding why we used "L" as well as the lines manuscript we modified to clarify this important observation.
Additionally, emphasizing one of the three color parameters based on its significance to the study is a customary practice in facial perception research (as seen in Kleisner et al., 2017).

Comment:
4.It's not clear about the colour information of facial stimuli. Whether the facial stimuli used in this study could represent the naturally occurring skin colour variations in the two populations, European American and Me'phaa. The average colour or the prototype is not enough as the variations within the same ethnic group are usually larger than the variations across the two ethnic groups. See the below reference. Thus, it would be very helpful to show the evolutionary meaningful skin colour range and variation of the real human faces from those two groups.
"Characterising the variations in ethnic skin colours: a new calibrated database for human skin," Ski. Res. Technol., vol. 23, no. 1, pp. 21–29, Feb. 2017, doi: 10.1111/srt.12295.

Response:
Thanks for this comment. Although our study standardizes skin color towards an "average" color, we also included the "Natural" group; which mantain individual variations for each ethnic group (8 natural Me'phaa faces and 8 European American faces). When comparing the natural group (with individual color variations) and its corresponding "average" group, perceptions don't change significantly, except for health. This might partially tell us that intra-groups color variation has less impact than the categorical (and experimental) change from one "average" skin color to the other one (i.e., Dark to Light and vice versa). We also included random effects for each evaluated facial photograph in our models. Here, we noticed that this random effect plays an important role in perceptions, but the fixed effect (transformed groups) consistently affects these individual variations across the groups. This demonstrates a systematized effect of contrasting "skin" color groups, despite the random effect of natural variation. Additionally, the obtained average skin colors are within the typical range found in Mexican and European American populations. We've rewritten a section to clarify our rationale (lines 201-208 and 211-215).
Regarding the acknowledgement of the evolutionarily meaningful skin color range and variation of real human faces, we did provide a summary of human skin color evolution in the first section of the introduction (lines 75-90). Here, we emphasize that studying the evolution of human skin color has many aspects that have become more complex to describe the whole color diversity in human groups (e.g., microclimates, migration patterns, angles of UV radiation, etc.). Many of these factors are related to natural selection. In our case, we documented how the perception of human skin color, a trait shaped by natural selection, may have implications in other processes like sexual and social selection, as well as cultural transmission. This has already been clarified in the introduction and discussion.

Comment:
5.Line 240-242: 'The average lightness value was L* = 66.74, SD = 1.37 in the light skin colour transformed versions and L* = 52.75, SD = 2.58 in the dark skin colour transformed versions.' Are the light versions including both light European American and light Me'phaa faces and the dark versions include both dark European American

Powered by Editorial Manager® and ProduXion Manager® from Aries Systems Corporation

and dark Me'phaa faces? If so, the standard deviation looks very small. Does that mean the European American and Me'phaa faces have the same lightness levels after manipulations? How does that come if both their original lightness and the prototype are different? It's better to give the actual colour distributions of the 48 face stimuli, not only the average lightness values.

Response:
Thank you for bringing up this point. Effectively, our study employs a crossover design. Hence, we obtained a total of 6 groups, which include a version darker and a lighter for each natural color group. Moreover, as we've clarified before, the process of manipulating skin color entailed applying a "color mask" derived from the two skin color prototypes. Consequently, within these versions, the transformed groups with paired colors (2 lighter and 2 darker) did not show significant variation in lightness. This outcome aligns with expectations, given that the color manipulation was conducted in a similar manner for both natural color groups. Furthermore, we observe that the lightness (L) of the transformed color groups (darker and lighter) remains consistent with their corresponding natural groups, as well as with what is commonly found and naturally distributed in both populations. We have provided clarification on this topic in lines 211-215 and 228-235 to enhance comprehension.

Comment:
6.It looks like the same facial stimuli changed not only the skin colour but also some other facial appearance traits after manipulations. E.g. in the below, the stimuli FC16, FC16co, and FC16cb are manipulated versions from the same original face, FC10, FC10cb, FC10co are from the same face. If that's the case, it looks like the beard completely changed after the colour changed, especially for the light version, the beard disappeared in the meantime. Those appearance traits could significantly influence sexual dimorphism and are even more sexually dimorphic traits than skin colour, and thus affect social judgements, especially judgements from the other gender.

Response:
Although the effect of facial sexually dimorphic traits, such as beard, is widely acknowledged, in our study it did not seem to be a significant factor. In the next forest plot we show the perceived attractiveness random estimates for all three skin color versions for each of the 16 faces (form right to left: natural version, light skin color version, dark skin color version).

The forest plot shows that none of the two natural versions (FC10, FC16) got significant higher or lower ratings than the main estimate (right column). It also shows that removing the beard did not affect the rates significantly in comparison with the main estimate for each version (light skin color version, middle column; dark skin color version, left column). We have performed the same exploration for all response variables, and we found a similar result for all of them except for the perceived masculinity ratings, however, even those differences were not significant. The rest of the forest plot can be found in the R Markdown code lines 364, 594, 720, 851, and 984).

Comment:
7.The facial gloss could have a big influence on the lightness measurement results based on image methods. E.g. the stimulus FI15 below seems to have a considerable area of highlight. Within these areas, the colour could be completely different from the normal skin colour. Any considerations regarding the facial gloss issue? And how was the gloss morphed into different colour versions?

Response:
Thank you for the observation. The skin color prototypes were created by averaging the values of 30 faces of each group. One of the reasons why we had that sample size was to minimize the effect of any kind of individual variation such as the one mentioned; moreover, we did not use the actual lightness values to test the effect of skin color. Now regarding a possible difference in the way the raters perceived this

stimuli in all of its versions, a random effect exploration showed that there are no significative differences between the scores this stimuli got and the main estimate for each version. We have included foster plots that show this in the R Markdown (code lines 364, 594, 720, 851, and 984).

Comment:
8.Images were captured under different environments and camera settings. It would be good to give information on settings, such as the white balance, CCT, ISO, aperture size, shutter speed, image formats (raw or post-processed), any colour characterization process, etc.

Response:
We appreciate this observation. We understand the relevance of giving as much information on settings as possible, however the information written in the lines 190 – 200 is all the setting information we took. Regarding the color characterization process, we followed the color calibration methodology reported in lines 208-217 which is a common method in the facial perception studies.

Comment:
9.During the experiments, participants were asked to rate the face without particular restriction or focus, e.g. based on the facial skin colour. In such a case, it’s only meaningful to compare the rating of the lighter, natural, and darker versions of the same face. It’s not reasonable to compare different faces or faces of different ethnic groups as the influence of structural facial traits is tangled together. However, those structural facial traits are not quantified or included in the analysis. in fact, previous studies show colour is less important than the structural facial traits in face perceptions. Meanwhile, various facial colour cues may be involved in face perceptions. See the reference below. E.g. the skin colour variation may matter a lot (homogeneous skin is preferred), which links to the colour and texture homogenization mentioned in the current study. Are there any considerations in the data analysis related to those confounding variables in visual stimuli? “Different colour predictions of facial preference by Caucasian and Chinese observers,” Sci. Rep., vol. 12, no. 1, p. 12194, Jul. 2022, doi: 10.1038/s41598-022-15951-8.

Response:
We appreciate this comment. A complete explanation about why we compared the ratings between the natural versions of the European American and Me’Phaa faces can be found in the answer to the comment number 15. Now, regarding the considerations to the possible confounding effect of shape and color homogenization, we took different approaches. We designed a crossing over study where the skin color version is nested within the group in order to control the effect of facial shape; and we did so by creating different skin color version of the same facial stimuli. On the other hand, we are aware that one of the side effects of our protocol was the homogenization of color and texture, and we know this may have an effect on some of the perceptions we evaluated, so we compare the ratings of the natural versions with the ratings of the concordant skin color transformed version so we could have a measurement of this effect. Our results showed that, whereas skin color and texture homogenization seemed to affect some the perceptions, especially perceived health, in general the effect was either not significant or lower that the one due to skin color change. Nonetheless, we recognize this limitation and, hence, we highlight it in the discussion (lines 531-548).

Comment:
10.In the linear mixed effect model, the face colour was coded as categorical variable. But the same colour category has different lightness levels, e.g. the natural European American face and the natural Me’phaa face have different L*, and the lighter versions of different faces have different L*... Why not use the actual lightness (L*) value as a continuous variable in the lme analysis? Actual lightness value could be a more accurate measure for assessing the effect of skin lightness on impression judgements. The fixed effect of other colour parameters, such as a* and b*, could be included in the lme analysis as well.

Response:

Powered by Editorial Manager® and ProduXion Manager® from Aries Systems Corporation

We coded the skin color as a categorical variable because it is a more reasonable way to carry out the statistical analysis of a crossover research design. In addition, using lightness values as a continuous variable would present some major inconveniences for the data that we have. Firstly, continuous variables cannot be included as random factors, so we could not be able to deal with the lack of independency of our observations (the ratings come from three versions of the same faces). Secondly, since the skin color manipulation was based on average values, the lightness values´ distribution would behave in a “categorical” fashion, that is, the values would group in three points instead of being constantly distributed in the whole range of variation. The decision about what type of variable most be used is not based on the accuracy of the measurement but in the type of variable that fits with the specific needs of the research design.

Comment:
11.What’s the observer variation? Any evaluation of the consistency across different raters.
Of the observer variations we assessed, factors such as sex, age, sexual preference, self-perception of ethnic group, and skin color were included. These variations and their effects are reported in the section titled "Facial Perception Rates." Given that we recognize the relevance of the individual observer variation more than variables we measured them, we also decided to include “Rater” as a random factor in our mixed models. We know that, traditionally, a measure such as the Cronbach's alpha is calculated to know the participants response reliability. However, we think that our approach is better because mixed models do not only measure this sort of variance but also take it into account when they estimate the standard errors.
Comment:
12.Are there any differences in the results rated by mestizo participants and European participants? Is there any effect of the participants’ ethnicity on ratings?

Response:
We appreciate this question. We did not find that the raters’ ethnicities had an effect on their responses; and, as a matter of fact, this question was already asked by the first reviewer during the first submission and we wrote our answer with the whole process that led us to our conclusion in the statistical analysis section, lines 313-318.

Comment:
13.What’s the relationship between participants’ self-perceived skin colour ratings and their actual skin colour? Has their skin colour ever been measured by instruments like spectrophotometers? There might be a mismatch between self-reported and measured values.

Response:
This is an interesting question. Based on the literature available we do believe that there may be a mismatch between self-reported, reported by someone else and objective measures of skin color in Mexico and in other Latin American countries. In these regions, individuals could perceive themselves as lighter compared to others' perceptions and even lighter than what objective measures indicate. Despite the widespread theoretical consideration of this bias, to our knowledge, no studies have directly addressed this matter within Latin American populations. This highlights a significant knowledge gap that presents a substantial opportunity for investigation. With that being said, we deem this concept to extend beyond the scope of our current study, and we recognize that a completely new study is required to comprehensively examine and test this hypothesis.

Comment:
14.It would be interesting to know whether there are any significant differences in the structural facial traits (facial shapes and facial features) between the European American and Me’phaa faces, and what are the differences. It might be good to give some evidence based on the image database used in the present study.

Response:
This is an interesting question. A recent study found that some components of facial shape do vary across populations (Kleisner et al., 2021), and ethnicity is usually a significant predictor of facial shape variation in geometric morphometric studies.

Powered by Editorial Manager® and ProduXion Manager® from Aries Systems Corporation

Therefore, we do expect differences in the facial shape traits between the European American and Me'phaa faces, nonetheless, similar to the previous answer, we think that this idea is beyond the reach of our study. In this study we only focused on controlling the effects of shape, however, as suggested, we are currently working on a study on facial shape, using the same image database, where we sought to test the effect of shape variation on the same perceptions, and we model the facial shape configurations that best correlate with each of the perceptions in combination with skin color

Comment:
15.Both the abstract line 51-53 ('We found that….faces of …were.. more attractive….') and the discussion line 476-479 ('In comparison with….'.) gives an unreasonable conclusion. This conclusion is not justifying the effect of skin colour on facial impression but makes judgements which are out of the scope of the current study. It's hard to tell whether those comparisons were made based on the L* or other skin colour information or texture or the structural facial traits of the two ethnic groups. From what has been written, the scope of this study is to understand the role of skin lightness on face perceptions, not to compare the attractiveness of the two different ethnic faces. And it's not rational to make those comparisons on the bases of very different both structural facial traits and colour traits.

16.Similar issues for those statements in the results part: European American faces were rated 'x times' more attractive/trustworthy… than Me'phaa faces. Current data and analysis can only show the effect of skin colour, but cannot tell whether those 'x times' come from colour or other factors, also cannot prove the link between 'which ethnic face is more attractive' and 'colour plays a role in it'.

Response:
We agree with these two reviewer comments. Indeed, the focus of this study is to provide experimental evidence of how skin color affects social perceptions in a Mexican population, not to compare social perceptions of two ethnic groups. These groups not only differ in skin color but also in many other physical and cultural attributes, as previously mentioned. To make this objective clearer, we've made changes in the lines the reviewer mentioned and through the whole manuscript, directing the interpretation towards skin color and away from other facial or cultural attributes specific to ethnicity. Please see lines 54-57, and the results section for these modifications.
On the other hand, we used the term "x times" because it is the accurate way to describe differences when the estimated effect comes from a Poisson GLM. In this type of GLM, the effects are expected to be exponential. Therefore, when incorporating a categorical predictor, the estimates are expressed in terms of how many times it is greater or lesser compared to the baseline, instead of a fixed and continuous difference. Although we retained these terms ("x times"), we shifted the focus to skin color (evaluated in the L dimension) and not towards an "ethnic group." Thank you for this important observation.

Comment:
17.Whether the current results lead to the hypothesis? Skin colour preference/social preference also includes some mainstream aesthetic criteria, e.g. tanning skin is preferred in Western countries while whitening skin is preferred in Eastern countries, which may have nothing to do with racism or discrimination. Have these factors been considered in the current study? From the current data and analysis, it seems to be hard to elucidate the convincing mechanisms or drives behind the phenomenon. More survey and investigation is needed to know the clear answer.

Response:
This is an interesting question. We agree with the statement that some mainstream aesthetic criteria may play a role in the skin color preferences and that preference for tanning skin in Western countries is one example of it. However, skin color-based stratification and racism were in the very foundation of Latin American societies and still have strong effects on their social dynamic. Therefore, we think this is a more plausible factor that could explain the social preferences reported here. Nonetheless, we share the opinion that more investigation is needed to elucidate the relative

	contribution of mainstream aesthetic criteria on skin color preferences.<br><br>Comment:<br>18.Are there any considerations related to the adaptation to the living environment? Raters might be exposed to different environments composed of a variety of coloured faces. The long-term adaptation to the environment might influence their perceptions. It might be helpful to provide some evidence or data about the variety/proportions of faces in the participants' living environment.<br><br>Response:<br>Thank you for this observation. There are some studies that suggest that people are better at judging familiar faces, such as faces within the same ethnicity. We did propose this possibility as an explanation of the differences we observed in the effects of skin color between the two groups. We have rearranged the paragraph we had previously written to make it more visible (lines 549-557). It is important to point out that our discussion comes from an approximated measurement because there is not available data that allowed us to test this more precisely.<br><br>Comment:<br>19.Line 90: it would be more precise to say 'lower and more seasonal UVR'.<br><br>Response:<br>Thank you for this observation. We have changed the term.<br><br><br><br>Alaluf, S., Atkins, D., Barrett, K., Blount, M., Carter, N., & Heath, A. (2002). The impact of epidermal melanin on objective measurements of human skin colour. Pigment Cell Research, 15(2), 119–126. https://doi.org/10.1034/j.1600-0749.2002.1o072.x<br>Kleisner, K., Kočnar, T., Tureček, P., Stella, D., Akoko, R. M., Třebický, V., & Havlíček, J. (2017). African and European perception of African female attractiveness. Evolution and Human Behavior, 38(6), 744–755. https://doi.org/10.1016/j.evolhumbehav.2017.07.002<br>Kleisner, K., Tureček, P., Roberts, S. C., Havlíček, J., Valentova, J. V., Akoko, R. M., Leongómez, J. D., Apostol, S., Varella, M. A. C., & Saribay, S. A. (2021). How and why patterns of sexual dimorphism in human faces vary across the world. Scientific Reports, 11(1), 1–14. https://doi.org/10.1038/s41598-021-85402-3
**Additional Information:**	
**Question**	**Response**
**Financial Disclosure**<br><br>Enter a financial disclosure statement that describes the sources of funding for the work included in this submission. Review the submission guidelines for detailed requirements. View published research articles from *PLOS ONE* for specific examples.<br><br>This statement is required for submission and **will appear in the published article** if the submission is accepted. Please make sure it is accurate.	The present work was part of the degree requirements for JMR Doctorate thesis at the Programa de Maestría y Doctorado en Psicología, UNAM. JMR received a Doctorate Nacional grant from the Consejo Nacional de Ciencia y Tecnología (CONACyT, CVU: 921005), and the work was supported by founding from UNAM-PAPIIT [grant numbers IA209416, IA207019] and CONACYT Ciencia Básica [grant number 241744]. The funders had no role in study design, data collection and analysis, decision to publish, or preparation of the manuscript.

Powered by Editorial Manager® and ProduXion Manager® from Aries Systems Corporation

**Unfunded studies**<br>Enter: *The author(s) received no specific funding for this work.*<br><br>**Funded studies**<br>Enter a statement with the following details:<br>• Initials of the authors who received each award<br>• Grant numbers awarded to each author<br>• The full name of each funder<br>• URL of each funder website<br>• Did the sponsors or funders play any role in the study design, data collection and analysis, decision to publish, or preparation of the manuscript?<br>• **NO** - Include this sentence at the end of your statement: *The funders had no role in study design, data collection and analysis, decision to publish, or preparation of the manuscript.*<br>• **YES** - Specify the role(s) played.<br><br>* typeset	
**Competing Interests**<br><br>Use the instructions below to enter a competing interest statement for this submission. On behalf of all authors, disclose any competing interests that could be perceived to bias this work—acknowledging all financial support and any other relevant financial or non-financial competing interests.<br><br>This statement is required for submission and **will appear in the published article** if the submission is accepted. Please make sure it is accurate and that any funding sources listed in your Funding Information later in the submission form are also declared in your Financial Disclosure statement.<br><br>View published research articles from *PLOS ONE* for specific examples.	The authors have declared that no competing interests exist.

**NO authors have competing interests**<br><br>Enter: *The authors have declared that no competing interests exist.*<br><br>**Authors with competing interests**<br><br>Enter competing interest details beginning with this statement:<br><br>*I have read the journal's policy and the authors of this manuscript have the following competing interests: [insert competing interests here]*<br><br>* typeset	
**Ethics Statement**<br><br>Enter an ethics statement for this submission. This statement is required if the study involved:<br><br>• Human participants<br>• Human specimens or tissue<br>• Vertebrate animals or cephalopods<br>• Vertebrate embryos or tissues<br>• Field research<br><br>Write "N/A" if the submission does not require an ethics statement.<br><br>General guidance is provided below. Consult the submission guidelines for detailed instructions. **Make sure that all information entered here is included in the Methods section of the manuscript.**	All procedures were approved by the appropriate National Autonomous University of Mexico ethics committee (FPSI/CE/01/2016).

Powered by Editorial Manager® and ProduXion Manager® from Aries Systems Corporation

**Format for specific study types**<br><br>**Human Subject Research (involving human participants and/or tissue)**<br>• Give the name of the institutional review board or ethics committee that approved the study<br>• Include the approval number and/or a statement indicating approval of this research<br>• Indicate the form of consent obtained (written/oral) or the reason that consent was not obtained (e.g. the data were analyzed anonymously)<br><br>**Animal Research (involving vertebrate animals, embryos or tissues)**<br>• Provide the name of the Institutional Animal Care and Use Committee (IACUC) or other relevant ethics board that reviewed the study protocol, and indicate whether they approved this research or granted a formal waiver of ethical approval<br>• Include an approval number if one was obtained<br>• If the study involved *non-human primates*, add *additional details* about animal welfare and steps taken to ameliorate suffering<br>• If anesthesia, euthanasia, or any kind of animal sacrifice is part of the study, include briefly which substances and/or methods were applied<br><br>**Field Research**<br><br>Include the following details if this study involves the collection of plant, animal, or other materials from a natural setting:<br>• Field permit number<br>• Name of the institution or relevant body that granted permission	
**Data Availability**<br><br>Authors are required to make all data underlying the findings described fully available, without restriction, and from the time of publication. PLOS allows rare exceptions to address legal and ethical concerns. See the PLOS Data Policy and FAQ for detailed information.	Yes - all data are fully available without restriction

A Data Availability Statement describing where the data can be found is required at submission. Your answers to this question constitute the Data Availability Statement and **will be published in the article**, if accepted.<br><br>**Important:** Stating 'data available on request from the author' is not sufficient. If your data are only available upon request, select 'No' for the first question and explain your exceptional situation in the text box.<br><br>Do the authors confirm that all data underlying the findings described in their manuscript are fully available without restriction?	
**Describe where the data may be found in full sentences. If you are copying our sample text, replace any instances of XXX with the appropriate details.**<br><br>• If the data are **held or will be held in a public repository**, include URLs, accession numbers or DOIs. If this information will only be available after acceptance, indicate this by ticking the box below. For example: *All XXX files are available from the XXX database (accession number(s) XXX, XXX.*).<br>• If the data are all contained **within the manuscript and/or Supporting Information files**, enter the following: *All relevant data are within the manuscript and its Supporting Information files.*<br>• If neither of these applies but you are able to provide **details of access elsewhere**, with or without limitations, please do so. For example:<br><br>*Data cannot be shared publicly because of [XXX]. Data are available from the XXX Institutional Data Access / Ethics Committee (contact via XXX) for researchers who meet the criteria for access to confidential data.*<br><br>*The data underlying the results presented in the study are available from (include the name of the third party*	All data, stimuli and codes for statistical analyses are openly available through the Open Science Framework: https://osf.io/6gn5t/?view_only=89b534d1095b4abf87411b64fd6d8e08

and contact information or URL).<br>• This text is appropriate if the data are owned by a third party and authors do not have permission to share the data.<br><br>* typeset	
Additional data availability information:	Tick here if your circumstances are not covered by the questions above and you need the journal's help to make your data available.

Powered by Editorial Manager® and ProduXion Manager® from Aries Systems Corporation

Manuscript Click here to access/download;Manuscript;Manuscript.docx

Effects of facial skin pigmentation on social judgments in a Mexican population

Jaaziel Martínez-Ramírez[1] , David Puts[2] , Javier Nieto[3] , Isaac G-Santoyo[1,4*]

1. Neuroecology lab, Faculty of Psychology, National Autonomous University of Mexico, Mexico City, Mexico.
2. Department of Anthropology, Pennsylvania State University, Pennsylvania, United States of America.
3. Laboratory of Learning and Adaptation, Faculty of Psychology, National Autonomous University of Mexico, Mexico City, Mexico.
4. Unidad de Investigación en Psicobiología y Neurociencias, National Autonomous University of Mexico, Mexico City, Mexico.

* Corresponding author

E-mail: isantoyo@psicologia.unam.mx (IG-S)

Abstract

People quickly and involuntarily form impressions of others based on their facial physical attributes, which can modulate critical social interactions. Skin pigmentation is one of the most variable and conspicuous facial traits among human populations. Empirical evidence suggests that these variations reflect ancestral ecological selective pressures balancing cutaneous vitamin D synthesis with the protection of the dermis from ultraviolet radiation. Nevertheless, skin pigmentation may currently be subject to additional selective pressures. For instance, the colonial era in Central and South America developed a highly stratified society based on ethnic origins, and light skin pigmentation became associated with higher social status and deference. This association could have originated through historical social learning that promoted favorable social perceptions towards individuals with lighter skin color and unfavorable perceptions towards individuals with darker skin color, which could still be present in the perception of current populations.

Facial skin pigmentation is also sexually dimorphic, with males tending to exhibit darker skin than females, a difference that could be driven by sexual selection. To explore whether social learning and sexual selection represent additional selective pressures on skin pigmentation, we tested how this facial trait influences fundamental social perceptions in a Mexican population (N = 700, 489 female). We sampled facial images of eight European American males with natural lighter facial skin and eight males from an indigenous pre-Columbian community from Mexico, the Me'Phaa, with natural darker facial skin. We produced stimuli from these images by varying the skin pigmentation while preserving the facial shape. Stimuli were rated on attractiveness, trustworthiness, perceived health, dominance, aggressiveness, and femininity/masculinity. We found that the natural light-

skinned faces were perceived as more attractive, trustworthy, and healthy but less dominant than the natural dark faces. Furthermore, by varying the facial skin color in these original groups, we altered the perceptions of them, mainly their attractiveness. These results partially support the hypothesis that dark facial skin color may help males compete for mates. Also, the results strongly support the view that lighter facial skin color became associated with social benefits through social learning in this Mexican population. Our findings, when viewed through the lens of cultural evolution, align with previous research in social psychology and anthropology. They hold the potential to offer a comprehensive understanding of the origin of this social phenomenon of cultural transmission, which currently plays a role in the formation of racial attitudes, stereotyping, and racial inequality in Mexican and other Latin American populations.

Introduction

Human faces provide rich information about salient attributes, such as mate quality [1,2], social dominance [3], and fecundity [4]. Hence, people quickly and involuntarily form impressions of others based on their facial appearance [5,6], which in turn may influence important social outcomes [7,8]. Skin pigmentation is one of the most conspicuous and variable facial traits of human populations [9]. The main pigments—melanin, hemoglobin, and carotenoids—absorb and reflect different wavelengths of light within the visible spectrum, and collagen also contributes by scattering light [10,11]. The skin's pigments vary not only between individuals and populations but also between men and women, perhaps because of sexual selection [12]. Natural selection initially favored

darker skin as a mean to protect against the harmful effects of UV radiation in the tropics. The pressure of selection then shifted toward lighter skin among those humans who spread into higher latitudes with lower levels of UVR [13–15]. Melanin, the principal skin pigment that provides this gradient of light to dark color, acts as a photoprotective filter that reduces the penetration of all wavelengths of light into subepidermal tissue. Under the high UVA levels near the equator, dark skin pigmentation is particularly advantageous for protecting folate and reducing the risk of fatal birth defects. Human migration beyond the tropics reduced the selective advantage of this photoprotective property and selected instead for lower melanization to facilitate cutaneous vitamin D biosynthesis, critical to the regulation of many biological processes [16]. Although these events are widely accepted as the main evolutionary events generating human skin pigment variation, this product of natural selection could be currently subjected to other ecological pressures in addition to those that caused its origin.

Cultural transmission is the main means for passing on adaptative behaviors that improves one´s chances for survival and reproduction [17]. It is especially adaptive if a transmitted behavior saves learners the costs of individual learning through trial and error. For instance, cultural transmission can influence mate preferences through two different mechanisms [18]; by reinforcing a preference for a trait that may signal biological quality if that preference significantly contributes to reproductive success, such as offspring quality, or by promoting new preferences for traits that do not appear to have direct links to the bearer's biological quality [19]. In this case, new social preferences may be selected by cultural transmission if the novel trait positively influences social benefits in a particular population [20].

The colonial period in Mexico and all countries of Central and South America led to a highly stratified society based on ethnic origins. During the 300 years of that period, light skin pigmentation, usually present in Spanish colonizers, was one of the most conspicuous phenotypic traits associated with higher social status and hence access to social benefits, such as education and wealth [21]. This racial/color hierarchy system, known as *castes*, placed the European colonizers at the top of the social hierarchy and the indigenous populations at the bottom, it developed deep roots in Latin American society, and even though the *caste* system was officially abolished, there is robust evidence of social stratification based on skin color in contemporary Mexican society [22], which can be accompanied by racial attitudes, stereotyping and racial inequality [22].

Recent studies have shown that people with darker skin have, on average, lower educational levels, occupational status, and hourly earnings, are more likely to live in poverty, and have fewer chances of social mobility than do people with lighter skin; these differences are significant even after controlling for sociodemographic factors such as age, gender, and parental education [23–25]. In addition, a cultural preference for light skin and other European phenotypical features is also influenced by social media in Western societies, which might reinforce the association between skin color and social status. Hence, if light skin is culturally preferred because it is associated with social benefits, prestige, or deference, a new skin pigmentation signal may have emerged through an extension of historical, sociocultural learning.

Another non-mutually exclusive hypothesis is that facial skin pigmentation could be driven by perceptions shaped by sexual selection. Human skin pigmentation is a sexually dimorphic trait in the sense that men usually have darker skin pigmentation than women

[26–28]. The sex difference is due to exposure of skin tissues to differing ratios of androgens to estrogens, particularly at puberty. Testosterone has a stronger effect than estrogen on melanin synthesis and vascularization of the upper dermis [29–31]. In addition, males develop a more intense facultative pigmentation after sun exposure and retain it for longer periods than females whereas female skin lightens faster after a reduction in sun exposure [32]. This sexual dimorphism might influence sexual preferences, since several studies have shown that darker skin in men is perceived as more attractive [33–36]. Therefore, this female preference for darker skin color could reflect ancestral selection pressures shaping perceptions of an androgen-dependent trait. In this case, the basis of this preference may be the honest signaling of mate quality in terms of male immune function (i.e., immunocompetence handicap hypothesis), vigor, or other physiological costs of developing this trait [37–39].

Although the effects of skin pigmentation on social perceptions is an active area of research in social psychology, prior research has focused mainly on racial attitudes and generally does not employ an evolutionary framework. For instance, Hagiwara et al. [40] evaluated affective reactions and other social attributions towards African American men by manipulating facial shape features (nose and lips) while controlling for skin tone. Similar studies have examined the role of skin tone and facial shape features in racial attitudes through similar methods [41,42]. Moreover, most experimental studies of facial perception have been carried out on participants from WEIRD (Western, educated, industrialized, rich, and democratic) societies, thus limiting the generalizability of findings [43]. Finally, prior studies in non-WEIRD Latin American samples have implemented a

social theoretical framework with a non-experimental design to explore correlations between skin tone and social attributes, such as status or labor hierarchy [44].

In this study, we used an experimental design with a Mexican population to determine whether facial skin color can influence certain social perceptions: ~~attractiveness;~~ attractiveness, trustworthiness, perceived health, dominance, aggressiveness, and femininity/masculinity [45–47]. To that end, we performed a crossover experiment on men's faces, selecting two groups of faces with contrasting innate skin pigmentation: the first composed of faces with natural light facial skin tone, selected from a European American population, and the second composed of faces with a darker facial skin tone, selected from a Mexican pre-Columbian population, self-identified as Me'Phaa. Using these two groups, we created six sets of facial stimuli by varying the natural skin pigmentation while preserving the facial shape. Finally, we presented all stimuli to be evaluated in the six social dimensions previously mentioned.

We hypothesize that if social learning is acting on the perceptions of facial skin pigmentation, we expect that the lighter-skinned group would be perceived as more attractive, more trustworthy, and healthier because positive evaluations of these attributes strongly promote pro-sociality and social benefits, but less dominant and less aggressive because dominance and aggressiveness, in contrast, are considered to be potentially harmful and may lead to avoidance of social interactions [48]. However, if skin pigmentation more strongly reflects ancestral selection shaping perceptions of an androgen-dependent trait, then we would expect that faces with darker skin would be perceived not only as more attractive and healthier but also as more dominant, aggressive, and masculine due to the association between these last three attributes and the behavioral and

physiological effects of androgens [49,50]. In both cases, if differences in skin pigmentation are driving these effects, we expect that the experimental manipulation of facial skin color would change the previous patterns in the perceptions of both the European American and Me´Phaa faces. Finally, if neither light nor dark skin color affects any social perception, of the original faces and the experimentally exchanged stimuli, then skin color would play no role in either social selection or the sexual selection of dimorphic traits.

Material and methods

Participant raters

A total of 489 Mexican adult women (mean age = 25.4 years, SD = 8.6 years) and 211 Mexican adult men (mean age = 26.5 years, SD = 9.1 years) participated as raters in this study. Self-reported ethnicities were 84.0 % Mestizo, 6.3 % European, 3.9 % Indigenous (particular groups not specified), 0.3 % African, and 6.3 % Other. A total of 485 participants reported living in Mexico City, 95 in the surrounded metropolitan area, and the rest in different states of Mexico. All the raters were recruited online through a board advertisement from the Department of Psychology at the National Autonomous University of Mexico.

Skin color facial stimuli

We first created two contrasting light and dark facial skin prototypes by selecting 30 facial photographs of young European American men and 30 facial photographs of young Me'Phaa Mexican Indigenous men from larger photograph sets. (See below the criteria of

selection for both groups of photographs). The European American faces were selected from a set of facial photographs of the Department of Anthropology at Pennsylvania State University [51]. Facial photographs were taken from a distance of 2 m using a 12-megapixel Olympus E-300 camera with a mounted flash at a resolution of 1200 × 1000 pixels. The facial photographs of the Me’Phaa were selected from a collection of facial photographs from the NeuroEcology Lab at the National Autonomous University of Mexico. These facial photographs were taken from a distance of 2m using a 21-megapixel Nikon 5500 camera illuminated by two studio strobes (Lusana Studio, 400w) with reflective umbrellas. The strobes were placed 1.85 m from the target on each side and lights were arranged at 40° angles. In both sets of facial photographs participants were asked to remove any earrings, glasses, or facial jewelry, and to maintain a neutral expression.

The two groups of facial photographs (European American and Me´Phaa) were selected in order to obtain two samples of faces whose pigmentation was contrasting enough to avoid an overlapping effect: one group with lighter skin and color ranges typical for the European American average, and another group with darker skin and color ranges typical for the Indigenous Mexican average~~, and because both skin colors are within the typical range of colors found in~~ Mexican population. Therefore, in order to evaluate if the facial photographs in both groups met these criteria, we measured the skin color values in CIELab color space. Following procedures reported in previous studies [52,53], we first standardized the facial images by color calibration, then we took color samples of three separate patches located on the forehead and on each cheek and calculated the average CIELab values using Photoshop CS6. Lightness (*L**) value was the parameter that change the most between the ethnic groups we sampled [54].Therefore, we focused on this

parameter a representative value of skin color differences between the two selected groups. Here, European American (L^* mean = 61.08, SD = 6.42) and Me'Phaa (L^* mean = 49.84, SD = 4.05) mean lightness were statistically different (t (58) = -8.07, $p < 0.01$), and lightness values for both groups were in the ranges of White/European and Hispanic ethnicities reported in previous studies [55,56].

From the 60 facial photographs selected, we created two composite faces that averaged the skin color of the 30 original faces from each group using the software Psychomorph [57]. The averaged composite face of the European American men was the light skin prototype, while the composite face of the Me'Phaa men was the dark skin prototype. Next, we randomly selected eight faces from the European American ~~men's~~ set and eight from the Me'Phaa ~~men's~~ set and created two different versions of each face, one displaying light skin and ~~the~~ one displaying dark skin. These two versions were created by adding or subtracting 70% of the skin color differences between the light and dark facial skin prototypes previously created while leaving facial shape unchanged. Finally, we repeated the skin color measurement process (see above) in both groups of the new composite faces (light skin color transformed and dark skin color transformed faces). The average lightness value was L^* = 66.74, SD = 1.37 in both light skin color transformed versions (i.e. Natural Me´phaa and Natural European American faces) and L^* = 52.75, SD = 2.58 in both dark skin color transformed versions. The method we used for skin color manipulation slightly increased the lightness values with respect to the values of the natural faces, the increases were not statistically significant for any group (t (44) = -0.52, $p = 0.6$; t (44) = -0.43, $p = 0.6$), and both groups stayed within the ranges reported for White/European and Hispanic ethnicities in previous studies [55,56]. At the end of this

process, we obtained three versions for each of the eight European American and eight Me'Phaa faces: a natural unmodified version (NAT), a light skin color transformed version (LSCt), and a dark skin color transformed version (DSCt; Fig. 1), for a total of 48 facial stimuli.

Fig. 1. Example stimuli. Three versions of the same facial stimuli of a European American male (up) and a Me'Phaa male (bottom). Natural unmodified versions (left), Light Skin Color transformed versions (middle), and Dark Skin Color transformed versions (right).

Facial perception rates

To obtain the ratings, we divided the 48 stimuli into eight sets of six stimuli each. The stimuli were assigned to each set counterbalancing by group (European American and Me'Phaa) and version (NAT, LSCt, and DSCt) and we made sure that two or more versions of the same face were not included together in the same set. Given that the experimental transformation tended to homogenize facial skin color and texture, two features that affect perceptions of human faces [58,59], we decided to include the natural version of each face (NAT) to be able to compare the ratings of the natural versions with the ratings of the concordant skin color transformed version; that is, we compared the Natural versions with the Light skin color transformed versions in the European American faces and the Natural versions with the dark skin color transformed versions in the Me'Phaa faces. By doing this we could measure the effect of the color and texture homogenization.

The facial evaluations were performed online at the webpage labneuroecologiacog.com. Each participant was randomly assigned to one of the eight facial stimuli sets and was asked to rate each face separately on a 9-point Likert scale according to the following perceptions: attractiveness (1 = not attractive at all, 9 = very

attractive); trustworthiness (1= not trustworthy at all, 9= very trustworthy); perceived health (1= not healthy at all, 9= very healthy); dominance (1 = not dominant at all, 9 = very dominant); aggressiveness (1= not aggressive at all, 9= very aggressive); and femininity/masculinity (1= very feminine/not masculine at all, 9= very masculine/ not feminine at all). For all trials, we randomized both the order of facial stimuli and the order of the questions about the perceived qualities. In addition, to standardize the response for attractiveness in men and women, we asked the men participants, *"How attractive does this face look for a woman?"* (For similar methods see [60]). Finally, raters completed a socio-demographic survey and were asked about their sexual orientation, on a 5-point Likert scale, from entirely same-gender to entirely opposite-gender orientation. As a result, 610 raters (437 women and 173 men) identified themselves as opposite-gender oriented/heterosexual, 42 (15 women and 27 men) identified as same-gender sexual orientation, 35 (28 women and 7 men) identified as bisexual, and 13 (9 women and 4 men) reported not having any sexual orientation. We controlled the sexual orientation information in the statistical analysis (see below). In addition, self-perceived skin color may have been influencing the rater's responses [23,61]. Hence, raters were asked to rate their own skin color using the PERLA color palette. The PERLA color palette is a scale of 11 skin tones (1 = lightest, 11 = darkest) that was made to provide an easy-to-use portrayal of the typical skin tones found in regions of Latin America [62]. Similar to previous studies on the Mexican population [24], we found that 77. 6% of the raters´ responses were concentrated in the middle levels of the scale (4-6), while the rest were at the extremes: 12.7 % in the lightest skin tones ~~levels~~ (1-3) and 9.7% in the darkest ones (7-10; see S1 Fig.). Finally, all raters signed an informed consent form before evaluating facial stimuli, and all procedures were approved by the appropriate National Autonomous University of

Mexico ethics committee (FPSI/CE/01/2016) and are in compliance with Mexican legislation and the Code of Ethical Principles for Medical Research Involving Human Subjects of the World Medical Association (Declaration of Helsinki).

Statistical analysis

To determine whether the experimental manipulation of facial skin color modified the perceived qualities, we fitted Poisson generalized linear mixed models (GLMM) with the log link function. We decided to fit Poisson GLMMs because, unlike other studies where the ratings for each facial stimulus are averaged, here all dependent variables come from discrete raw ratings. Moreover, during the data exploration we observed a positively skewed counts-type distribution with most of the observations at the lower values in some of the facial perceptions (i.e., attractiveness perception). We ran one global model per each perception evaluated. Global models included as the dependent variable any of the following facial perceptions: attractiveness, trustworthiness, perceived health, dominance, aggressiveness, or femininity/masculinity. Fixed predictors for all global models included the ethnic group of each evaluated face (i.e., European American or Me'phaa faces), the natural and transformed skin color versions (i.e., NAT, LSCt or DSCt), sex of raters, and the interaction between ethnic group and skin color versions. To account for the potential non-independence of data, we included a random intercept for rater ID, a random intercept for facial photograph ID (the individual code of each original face used), and a random slope for skin color transformation on facial photograph ID.

To evaluate the effect of rater´s sexual preferences, we ran models for responses from all participants and models for responses from heterosexual participants only. Next, each global model was then reduced to obtain a minimum adequate model using the Akaike Information Criterion (AIC). We selected the models with the lowest AIC values and a ΔAIC higher than two units from the second most supported model [63]. When the difference between the best-supported model and the subsequent model was $\Delta<2$, we followed the statistical principle of parsimony [64], and hence we selected the minimum adequate model as the one with the lowest AIC and the least number of parameters (Table 1). Finally, once we obtained minimum adequate models, model diagnostics were performed, and we checked residual distribution and linearity of residuals in each model. In order to check for any effect of other factors not included in the models [65,66], we plotted Pearson residuals of each selected model against the rater´s self-reported ethnicities and the PERLA scale responses for self-perceived skin color. Since the plots did not show any clear effect, we did not include either of the two factors as fixed predictors. All analyses were performed using the *lme4* [67] and *bbmle* [68] packages in R, version 4.0.2 [69].

Table 1. Information criteria for the best fit models.

	df	$AICc$	$\Delta AICc$	$w_i(AICc)$
(a) Perceived attractiveness				
**Group + Color + Rater´s sex + Group:Color**	**14**	**14331**	**0.000**	**0.999**
Group + Color + Rater´s sex	12	14346	15.053	0.001
Group + Color + Group:Color	13	14348	16.957	0.000
(b) Perceived trustworthiness				
**Group + Color**	**11**	**15167**	**0.000**	**0.506**
Group + Color + Rater´s sex	12	15169	1.778	0.208
Group + Color + Group: Color	13	15170	2.596	0.138
(c) Perceived health				
**Group + Color + Group:Color**	**13**	**15561**	**0.000**	**0.707**
Group + Color + Rater´s sex + Group:Color	14	15563	1.892	0.274
Group + Color	11	15570	9.186	0.007
(d) Perceived dominance				
**Group + Rater´s sex**	**10**	**15485**	**0.000**	**0.493**
Group + Color	11	15488	2.685	0.129
Group + Color + Group: Color	13	15489	3.137	0.103
(e) Perceived aggressiveness				
**Group + Color + Group:Color**	**13**	**16625**	**0.000**	**0.663**
Group + Color + Rater´s sex + Group:Color	14	16626	1.768	0.274
Group + Color	11	16630	5.479	0.043
(f) Perceived masculinity				
**Group + Color + Group:Color**	14	15486	0.000	0.482
Group + Color + Rater´s sex + Group:Color	**13**	**15486**	**0.035**	**0.474**
Group + Color	11	15492	6.367	0.020

Information criteria and fixed factors included for the three best fit models for: (a) Perceived attractiveness, (b) Perceived trustworthiness, (c) Perceived health, (d) Perceived dominance, (e) Perceived aggressiveness, and (f) Perceived masculinity.

Models are in descending order. ΔAIC is the change in AIC between each model and the best model. Akaike weights are conditional probabilities for each model being the best model. Models in bold are the selected ones.

Results

Perceived attractiveness

Perceived attractiveness differed significantly between the two groups when they had their natural skin color (z = 6.51, p < 0.01). The natural ~~lighter facial~~ (i.e. composed ~~by~~ natural European American faces) ~~group were~~ rated 1.70 times more attractive (M = 4.30, 95% CI [3.83 - 4.84]) ~~that~~ the natural ~~darker facial~~ group (i.e. composed ~~by~~ natural Me´phaa faces; M = 2.50, 95% CI [2.21 - 2.82], Table 2). Furthermore, there were effects when natural skin color was changed within each of the two groups. Lightening the natural darker group (i.e. Me’Phaa faces) made them more attractive (M = 3.75, 95% CI [3.42 - 4.11], z = 7.42, p < 0.01), and darkening the opposite group (i.e. European American faces) made them less attractive (M = 3.30, 95% CI [3.02 - 3.60], z = -6.25, p < 0.01). In addition, we observed an effect of skin color and texture homogenization due to experimental manipulation. Both groups became slightly more attractive when skin color and skin texture were homogenized (Me’Phaa faces: M = 2.94, 95% CI [2.68 - 3.21]; European American faces: M = 4.77, 95% CI [4.36 - 5.22]). However, this effect was significant for the ~~darker faces~~ group (i.e. Me’Phaa faces; z = -3.23, p < 0.01) but not for ~~opposite~~ group (i.e. the European American faces; z = -2.00, p = 0.34; Fig. 2). Finally, we observed a significant main effect of rater´s sex (z = 4.36, p<0.01); all the faces seemed more attractive to the male (M = 3.76, 95% CI [3.48 - 4.06]) than to the female raters (M = 3.27, CI 95% [3.06 - 3.49]). The direction of the effects and statistical significance were not affected when including the rater´s sexual preference (S1 Table.).

Table 2. Perception estimates with 95% confidence intervals.

	Me'Phaa			European American		
	**NAT**	**LSCt**	**DSCt**	**NAT**	**LSCt**	**DSCt**
	Est. [95%CI]	***Est. [95%CI]***	***Est. [95%CI]***	***Est. [95%CI]***	***Est. [95%CI]***	***Est. [95%CI]***
a) Perceived attractiveness	2.50 [2.21-2.82]	3.75 [3.42-4.11]	2.94 [2.68-3.21]	4.30 [3.83-4.84]	4.77 [4.36 5.22]	3.30 [3.02-3.60]
b) Perceived trustworthiness	4.21 [4.03-4.40]	4.48 [4.30-4.67]	4.33 [4.16-4.51]	4.75 [4.55-4.96]	5.06 [4.86-5.27]	4.89 [4.70-5.08]
c) Perceived health	4.28 [3.83-4.79]	5.51 [5.19-5.84]	5.21 [4.85-5.60]	5.10 [4.56-5.69]	5.96 [5.62-6.31]	5.05 [4.70-5.43]
d) Perceived dominance	5.23 [4.85-5.64]	□	□	4.31 [3.99-4.66]	□	□
e) Perceived aggressiveness	5.43 [5.17-5.69]	5.05 [4.85-5.26]	5.39 [5.17-5.62]	5.10 [4.85-5.35]	4.79 [4.59-4.99]	4.56 [4.37-4.77]
f) Perceived masculinity	6.18 [5.79-6.60]	5.15 [4.83-5.49]	5.97 [5.59-6.37]	5.14 [4.80-5.50]	4.83 [4.53-5.15]	4.93 [4.61-5.27]

Estimates (Est.) with 95% confidence intervals (CI) of the fixed predictors of the models for: (a) Perceived attractiveness, (b) Perceived trustworthiness, (c) Perceived health, (d) Perceived dominance, (e) Perceived aggressiveness, and (f) Perceived masculinity.
Note: (□*)* The Perceived dominance best fit model did not include the skin color transformation predictor, therefore the estimates shown represent the main effect of the natural skin color group only.

Fig. 2. Perceived attractiveness estimates for the Me'Phaa and European American faces within each color version. Estimates for the Natural versions (NAT), Light skin color transformed versions (LSCt), and Dark skin color transformed versions (DSCt) of the Me'Phaa (left) and European American (Right) faces. Error bars show 95% Confidence Intervals. Grey points and dashed lines indicate the random effect estimates for each Face ID within each color version.

Perceived trustworthiness

Natural lighter group were rated 1.13 times more trustworthy (*M* = 4.75, 95% CI [4.55 - 4.96], *z* = 5.19, *p* < 0.01) than natural darker group (*M* = 4.21, 95% CI [4.03 - 4.40], Table 2). In addition, lightening the natural darker group (i.e. Me'Phaa faces) increased their perceived trustworthiness (*M* = 4.48, 95% CI [4.30 - 4.67], *z* = 3.26, *p* < 0.01) whereas darkening the natural lighter group (i.e. European American faces) had no effect (*M* = 4.89, 95% CI [4.70 - 5.08], *z* = 1.44, *p* = 0.15). Skin color and texture homogenization did not increase scores for the natural darker faces (DSCt Me'Phaa faces M = 4.89, 95% CI [4.70 - 5.08], *z* = -1.43, *p* = 0.70), but did increase scores for the natural lighter faces (LSCt European American faces *M* = 5.06, 95% CI [4.86 - 5.27], *z* = -3.26, *p* = 0.01; Fig. 3). Finally, there was no effect of rater´s sex, as this predictor was not present in the minimum adequate model selected by AIC (Table 1). The direction of the effects and statistical significance were not affected when the rater´s sexual preference was included (S1 Table.).

Fig. 3. Perceived trustworthiness estimates for Me'Phaa and European American faces within each color version. Estimates for the Natural versions (NAT), Light skin color transformed versions (LSCt), and Dark skin color transformed versions (DSCt) of the Me'Phaa (left) and European American (Right) faces. Error bars show 95% Confidence Intervals. Grey points and dashed lines indicate the random effect estimates for each Face ID within each color version.

Perceived health

As with perceived attractiveness and trustworthiness, we found a significant difference in the perceived health scores between the natural darker and lighter faces (*z* = 2.20, *p* = 0.02). On average, natural lighter faces were perceived as being 1.19 times

healthier (*M* = 5.10, 95% CI [4.56 - 5.69]) than natural darker faces (*M* =4.28, 95% CI [3.83 - 4.79], Table 2). When facial skin color was changed within each ethnic group, the changes had different effects on perceptions of the two groups. Lightening the natural darker faces increased their perceived health (*M* = 5.51, 95% CI [5.19 - 5.84], $z = 4.96$, $p < 0.01$), whereas darkening the lighter faces had no effect (*M* = 5.05, 95% CI [4.70 - 5.43], $z = 0.20$, $p = 0.99$). Perceived health increased slightly for both groups when skin color and skin texture were homogenized (European Americans: *M* = 5.96 (95% CI [5.62 – 6.31], $z = -3.12$, $p = 0.02$; Me'phaa people: *M* = 5.21, 95% CI [4.85 - 5.60], $z = 4.38$, $p < 0.01$; Fig. 4). Finally, there was no effect of the rater´s sex, as this predictor was not present in the minimum adequate model selected by AIC (Table 1). The direction of the effects and statistical significance were not affected when the rater´s sexual preference was included (S1 Table.).

Fig. 4. Perceived health estimates for Me'Phaa and European American faces within each color version. Estimates for the Natural versions (NAT), Light skin color transformed versions (LSCt), and Dark skin color transformed versions (DSCt) of the Me'Phaa (left) and European American (Right) faces. Error bars show 95% Confidence Intervals. Grey points and dashed lines indicate the random effect estimates for each Face ID within each color version.

Perceived dominance

Unlike the previous qualities, perceived dominance was lower for natural lighter faces (*M* = 4.31, 95% CI [3.99 - 4.66], $z = -3.63$, $p < 0.01$) than for darker faces (*M* = 5.23, 95% CI [4.85 - 5.64], Table 2). Moreover, in contrast to perceived attractiveness, all faces received a lower dominance rating from male raters (*M* = 4.63, 95% CI [4.34 - 4.93], $z = -2.24$, $p = 0.02$) than from female raters (*M* = 4.88, 95% CI [4.61 - 5.16]). Finally, we were

unable to determine any effect from lightening, darkening, or homogenizing the faces, since these predictors were not present in the minimum adequate model selected by AIC (Table 1). Statistical significance of these results did not change in the model that included all raters (S1 Table.).

Perceived aggressiveness

Although perceived aggressiveness was lower for the lighter faces, the difference was not significant ($z = -1.83$, $p = 0.06$). Nevertheless, lightening the darker faces made them seem less aggressive ($M = 5.05$, 95% CI [4.85 - 5.26], $z = -2.76$, $p < 0.01$) but darkening the lighter faces also made them seem less aggressive ($M = 4.56$, 95% CI [4.37 - 4.77], $z = 4.17$, $p < 0.01$, Table 2). Finally, perceived aggressiveness did not change for either group when skin color and skin texture were homogenized (Me'Phaa faces $M = 5.39$, 95% CI [5.17 - 5.62]; $z = -0.23$, $p = 0.82$; European American faces $M = 4.79$, 95% CI [4.59 - 4.99]; $z = 2.35$, $p = 0.17$, Fig. 5.), and there was no effect of the rater´s sex, as this predictor was not present in the minimum adequate model selected by AIC (Table 1). Finally, the direction of the effects and statistical significance were not affected when the rater´s sexual preference was included (S1 Table.).

Fig. 5. Perceived aggressiveness estimates for Me'Phaa and European American faces within each color version. Estimates for the Natural versions (NAT), Light skin color transformed versions (LSCt), and Dark skin color transformed versions (DSCt) of the Me'Phaa (left) and European American (Right) faces. Error bars show 95% Confidence Intervals. Grey points and dashed lines indicate the random effect estimates for each Face ID within each color version.

Perceived masculinity

As with perceived dominance, perceived masculinity was lower for natural lighter faces (*M* = 5.14, 95% CI [4.80 - 5.50], *z* = -3.95, *p* < 0.01 than for natural darker faces (*M* = 6.18, 95% CI [5.79 - 6.60], Table 2). Moreover, lightening the darker faces made them seem less masculine (*M* = 5.97, 95% CI [5.59 - 6.37], *z* = -5.59, *p* < 0.01), but darkening the lighter faces had no effect (*M* = 4.93, 95% CI [4.61 - 5.27], *z* = 1.20, *p* = 0.83). In addition, perceived masculinity did not change for either group when skin color and skin texture were homogenized (DSCt Me'Phaa faces *M* = 5.97, 95% CI [5.59 - 6.37]; *z* = 1.05, *p* = 0.90; LSCt European American faces *M* = 4.83, 95% CI [4.53 - 5.15]; *z* = 1.82, *p* = 0.45, Fig. 6). Finally, there was no effect of the rater´s sex, as this predictor was not present in the minimum adequate model selected by AIC (Table 1). The direction of the effects and statistical significance was not affected when the rater´s sexual preference was included (S1 Table.).

Fig. 6. Perceived masculinity estimates for the Me'Phaa and European American faces within each color version. Estimates for the Natural versions (NAT), Light skin color transformed versions (LSCt), and Dark skin color transformed versions (DSCt) of the Me'Phaa (left) and European American (Right) faces. Error bars show 95% Confidence Intervals. Grey points and dashed lines indicate the random effect estimates for each Face ID within each color version.

Discussion

Our findings suggest that facial skin pigmentation influences social judgments in a Mexican population. Based on our results, we could distinguish two groupings of social perceptions that facial skin pigmentation affects in opposite directions. In comparison with

natural Me'Phaa faces, natural European American faces were perceived not only as more attractive, healthier, and more trustworthy but also as less dominant and less masculine. They were furthermore perceived as less aggressive, but not significantly. Moreover, the perceptions grouped in these two dimensions correlate more within than between dimensions (S2 Table.). The apparent grouping of social perceptions found here resembles the valence-dominance model of facial perception proposed by Oosterhof and Todorov [70] and recently discussed by Jones et al. [6]. The valence-dominance model suggests that humans make impressions of others based on their facial appearance in the dimensions of valence and dominance. The valence dimension is related to facial judgments such as trustworthiness and attractiveness; hence, higher rates of this dimension could promote positive social interactions, for example mate opportunities. The dominance dimension, in turn, is related to perceptions such as aggressiveness, dominance, and masculinity, which could inform about the physical or psychological capability to cause harm; hence, higher values of this dimension could negatively affect social interactions in a modern context [8]. Here, we found that natural light-skinned faces were rated more highly on all three aspects of social valence (attractiveness, healthiness, trustworthiness). In contrast, natural dark-skinned faces were rated more highly on two aspects of social dominance (dominance, masculinity).

In the context of our first hypothesis, these results support the idea that sociocultural learning favors the function of facial skin pigmentation as a mediator of social perceptions. Our Mexican participants showed a robust positive association of pro-social judgments (i.e., attractiveness, trustworthiness, and perceived health) with light skin pigmentation. During the Mexican colonial period, light skin pigmentation was one of the

most conspicuous phenotypic traits associated with a higher social status and access to different benefits, such as education and wealth [21]. Learning can reinforce a preference for a trait that may signal social benefits, even when the selected trait is not linked initially to any reproductive or survival benefit [19]. Therefore, a learned association over several generations between a physical trait—such as light skin—and deference might not only establish a ~~social~~ positive perception ~~for~~ the population that ~~share~~ this trait, but also give rise to racial stereotypes that negatively affect ~~others~~ coexisting populations, ~~which reinforces~~ the learning.~~.~~ Although our methodological design did not explicitly provide information about the ethnicity of the stimuli subjects, it is possible that the stereotypes derived from this strong associative learning might have acted as the driving force influencing the social scores. In Mexico, darker skin color suggests that individuals are more indigenous, and consequently considered ~~as~~ more socially unfavorable. Furthermore, several studies have shown cultural preferences for light skin pigmentation and other European phenotypical traits and within-group discrimination based on skin color, a social phenomenon known as colorism [71]. In addition, in Mexico and other Latin American countries, lightness of skin correlates with years of schooling, hourly earnings, occupational status, poverty, and social mobility [23–25]. Therefore, the association of pro-social attributes with light skin pigmentation we observed may be the result of a preference that emerged as an adaptative strategy to deal with social pressures during the colonial period, which nowadays is reinforced by social learning and cultural mechanisms such as colorism, or the influence of Western societies on social media.

We also found that the dark-skinned faces were rated as more dominant and masculine. However, in contrast to our second prediction, they were not rated as more

attractive or healthy. These results could indicate that skin pigmentation, as a trait mediated by androgens, could inform the viewer about the physical or psychological capability to cause harm by the bearer of the signal [72,73]; therefore, this signaling function might be relevant in sexual selection through male-male competition instead of female choice[74]. Similar results have been found in previous studies where androgen-dependent sexually dimorphic traits, such as facial shape [75], voice pitch [76,77], or height [78], more strongly affect the perception of dominance, aggressiveness, or masculinity rather than attractiveness.

The human face embodies a variety of signals and cues [79], and even though humans process faces in an integrative manner, this process follows a hierarchy of relevance in which different facial features are more important than others, depending on the perceptions evaluated [80,81]. In this way we found that some social perceptions were more affected by skin color than others. For example, lightening the Me'Phaa faces made them seem more attractive, healthier, and more trustworthy. Conversely, darkening the European American faces made them seem less attractive. Other perceived qualities, however, did not change systematically when facial skin color was changed. Perceived dominance was not affected by manipulations of facial skin color within either group. Lightening the Me'Phaa faces decreased their perceived masculinity and aggressiveness, but darkening the European American faces did not increase their perceived masculinity and aggressiveness. These results are consistent with studies on the role of skin color in certain perceptions, such as attractiveness [59,82], which show that skin color might contribute more than facial shape. Moreover, recent studies have shown that trustworthiness judgments rely on emotional expressions [8] or facial resemblance [83], and

that perceptions of dominance, masculinity, and aggressiveness seem to rely more strongly on facial shape [84–88]. Yet another possibility is that skin color interacts with facial shape in some of these perceptions, and it becomes relevant, only when facial shape strongly elicits the perception. This may explain why lightening of skin color reduced the perceived masculinity and aggressiveness for the Me´Phaa faces, which were initially rated as more masculine and dominant, but darkening had no effect on the European American faces.

Another possibility is that there were systematic differences in the way that the experimental manipulation of skin color affected the European American and Me´Phaa faces. In the statistical analysis we fitted random slopes for skin color transformation on each facial photograph ID to reduce the standard error caused by these differences; however, it is important to note that any manipulation presents a risk of such artifacts. Moreover, we not only lightened and darkened facial color but also homogenized it by removing such texture-related aspects as spots, freckles, or wrinkles [59,89,90]. Comparing the two color transformed versions of each group, that is the ones with texture and color homogenized, we observed, in general, the main effects of skin color previously reported (see Fig. 2-6); however, our results suggest that skin texture does have an effect, particularly on perceived health. Therefore, skin texture might interact with skin color in perceptions of light and dark skin, and such interactions could be an exciting avenue for further studies. In addition, unlike the authors of previous studies, we manipulated overall skin color instead of gradually changing each of its aspects (e.g., luminosity, hue) [33,35,46,53,60,82,91–94]. In our study, we aimed to test the differences in skin colors typically related to two different ethnic groups by using a broader manipulation of skin

color. Nonetheless, future studies could examine how each aspect of skin color contributes to the way a population is perceived.

Finally, previous studies have shown that individuals are better at judging faces of their own ethnicity [35,95]. In this study our Mexican participants were asked to rate facial stimuli of a more closely related ethnic group (Me´Phaa faces), and stimuli from a more distant ethnic group (European American faces). Although 83% of our raters reported living in Mexico City and the surrounded area, and hence were likely exposed to European facial features due to immigration of people of European ethnicity and access to the internet and social media [96,97], these differences in the effects of the skin color manipulation between European American and Me´Phaa faces might be due to different degrees of familiarity with the facial features of the two ethnic groups.

In summary, we show that the Mexican population uses facial color to make certain social judgments. We found that a dark-skinned face is perceived as more dominant and more masculine. Those perceptions may be due to a process of coevolution between the human mind and the sex difference in skin color. One may therefore infer that male skin became darker through male-male competition rather than through female choice. We also found that a light-skinned face is perceived as more attractive, more trustworthy, and healthier. Those perceptions may be due to the association between skin color and social status that prevailed in Mexico during more than three centuries of colonial rule. Today, they are maintained by colorism. We are nonetheless speculating, and more data will be needed. Similar results could be obtained from other Latin American societies, which share a common colonial history and social structure.

This study may complement the social psychology and sociological literature on skin color-based discrimination. In Latin American countries, the distinction between indigenous and non-indigenous populations is a fluid continuum with complex relationships with skin color and socioeconomic status. In Mexico, cultural practices, sense of belonging, and regional language use are the characteristics that best describe this classification [98,99]; nonetheless, skin color still has effects on the way ~~the Mexican population~~ perceive each other and, thus, make this distinction, mainly by promoting racial attitudes, and stereotypes. For example, Flores and Telles [25] found that in Mexicans with the same skin color, those with high income are twice as likely to be perceived and described as white than those with low income. Here we found that our raters were more willing to rate light skinned faces higher in all three perceptions that are closely related to positive social interactions. Remarkably, even when our results come from a study designed to address different questions, they support the idea of a cultural preference for light skin color based on previous findings of studies on racial attitudes and social stratification. Therefore, ~~the efforts from~~ different disciplines ~~to~~ develop a better understanding of the complex effects that skin color has on several aspects of t~~he~~ Mexican psychology and society ~~need to continue~~.

Acknowledgements

The authors are grateful to Juan Sebastían Lucero Carrasquilla for his help and assistance during the facial photograph acquisition of the Me’Phaa men sample.

References

1. Little AC, Jones BC, Debruine LM. Facial attractiveness: Evolutionary based research. Philosophical Transactions of the Royal Society B: Biological Sciences. 2011;366: 1638–1659. doi:10.1098/rstb.2010.0404

2. Puts DA, Jones BC, Debruine LM. Sexual selection on human faces and voices. J Sex Res. 2012;49: 227–243. doi:10.1080/00224499.2012.658924

3. Gonzalez-Santoyo I, Wheatley JR, Welling LLM, Cárdenas RA, Jimenez-Trejo F, Dawood K, et al. The face of female dominance: Women with dominant faces have lower cortisol. Horm Behav. 2015;71: 16–21. doi:10.1016/j.yhbeh.2015.03.006

4. Puts DA, Bailey DH, Cárdenas RA, Burriss RP, Welling LLM, Wheatley JR, et al. Women's attractiveness changes with estradiol and progesterone across the ovulatory cycle. Horm Behav. 2013;63: 13–19. doi:10.1016/j.yhbeh.2012.11.007

5. Ritchie KL, Palermo R, Rhodes G. Forming impressions of facial attractiveness is mandatory. Sci Rep. 2017;7: 1–8. doi:10.1038/s41598-017-00526-9

6. Jones BC, DeBruine LM, Flake JK, Liuzza MT, Antfolk J, Arinze NC, et al. To which world regions does the valence–dominance model of social perception apply? Nat Hum Behav. 2021;5: 159–169. doi:10.1038/s41562-020-01007-2

7. Olivola CY, Funk F, Todorov A. Social attributions from faces bias human choices. Trends Cogn Sci. 2014;18: 566–570. doi:10.1016/j.tics.2014.09.007

8. Todorov A, Olivola CY, Dotsch R, Mende-Siedlecki P. Social attributions from faces: Determinants, consequences, accuracy, and functional significance. Annu Rev Psychol. 2015;66: 519–545. doi:10.1146/annurev-psych-113011-143831

9. Barsh GS. What controls variation in human skin color? PLoS Biol. 2003;1: 19–23. doi:10.1371/journal.pbio.0000027

10. Samson N, Fink B, Matts PJ. Visible skin condition and perception of human facial appearance. Int J Cosmet Sci. 2010;32: 167–184. doi:10.1111/j.1468-2494.2009.00535.x

11. Edwards EA, Duntley SQ. THE PIGMENTS AND COLOR OF LIVING HUMAN SKIN. American Journal of Anathmy. 1939;65.

12. Darwin C. The Descent of Man, and Selection in Relation to Sex. J. Murray. London; 1871.

13. Jablonski NG, Chaplin G. The evolution of human skin coloration. J Hum Evol. 2000;39: 57–106. doi:10.1006/jhev.2000.0403

14. Adhikari K, Mendoza-Revilla J, Sohail A, Fuentes-Guajardo M, Lampert J, Chacón-Duque JC, et al. A GWAS in Latin Americans highlights the convergent evolution of lighter skin pigmentation in Eurasia. Nat Commun. 2019;10. doi:10.1038/s41467-018-08147-0

15. Deng L, Xu S. Adaptation of human skin color in various populations. Hereditas. 2018;155: 1. doi:10.1186/s41065-017-0036-2

16. Jablonski NG, Chaplin G. Human skin pigmentation as an adaptation to UV radiation. Proc Natl Acad Sci U S A. 2010;107: 8962–8968. doi:10.1073/pnas.0914628107

17. Feldman MW, Cavalli-Sforza LL. Cultural and biological evolutionary processes: Gene-culture disequilibrium. Isotopenpraxis. 1984;20: 1604–1607. doi:10.1073/pnas.81.5.1604

18. Verzijden MN, ten Cate C, Servedio MR, Kozak GM, Boughman JW, Svensson EI. The impact of learning on sexual selection and speciation. Trends Ecol Evol. 2012;27: 511–519. doi:10.1016/j.tree.2012.05.007

19. Little AC, Jones BC, Debruine LM, Caldwell CA. Social learning and human mate preferences: A potential mechanism for generating and maintaining between-population diversity in attraction. Philosophical Transactions of the Royal Society B: Biological Sciences. 2011;366: 366–375. doi:10.1098/rstb.2010.0192

20. Rendell L, Fogarty L, Hoppitt WJE, Morgan TJH, Webster MM, Laland KN. Cognitive culture: Theoretical and empirical insights into social learning strategies. Trends Cogn Sci. 2011;15: 68–76. doi:10.1016/j.tics.2010.12.002

21. Dixon AR, Telles EE. Skin color and colorism: Global research, concepts, and measurement. Annu Rev Sociol. 2017;43: 405–424. doi:10.1146/annurev-soc-060116-053315

22. Villarreal A. Stratification by skin color in contemporary Mexico. Am Sociol Rev. 2010;75: 652–678. doi:10.1177/0003122410378232

23. Telles E, Flores RD, Urrea-Giraldo F. Pigmentocracies: Educational inequality, skin color and census ethnoracial identification in eight Latin American countries. Res Soc Stratif Mobil. 2015;40: 39–58. doi:10.1016/j.rssm.2015.02.002

24. Campos-Vazquez RM, Medina-Cortina EM. Skin Color and Social Mobility: Evidence From Mexico. Demography. 2019;56: 321–343. doi:10.1007/s13524-018-0734-z

25. Flores R, Telles E. Social Stratification in Mexico: Disentangling Color, Ethnicity, and Class. Am Sociol Rev. 2012;77: 486–494. doi:10.1177/0003122412444720

26. van den Berghe PL, Frost P. Skin color preference, sexual dimorphism and sexual selection: A case of gene culture co-evolution? Ethn Racial Stud. 1986;9: 87–113. doi:10.1080/01419870.1986.9993516

27. Madrigal L, Kelly W. Human skin-color sexual dimorphism: A test of the sexual selection hypothesis. reply to frost (2007). Am J Phys Anthropol. 2007;133: 780–781. doi:10.1002/ajpa.20582

28. Aoki K. Sexual selection as a cause of human skin colour variation: Darwin's hypothesis revisited. Ann Hum Biol. 2002;29: 589–608. doi:10.1080/03014460210000019144

29. Giacomoni PU, Mammone T, Teri M. Gender-linked differences in human skin. J Dermatol Sci. 2009;55: 144–149. doi:10.1016/j.jdermsci.2009.06.001

30. Edwards EA, Hamilton JB, Duntley SQ, Hubert G. CUTANEOUS VASCULAR AND PIGMENTARY CHANGES IN CASTRATE AND EUNUCHOID MEN. Endocrinology. 1941;28: 119–128.

31. Manning JT, Bundred PE, Mather FM. Second to fourth digit ratio, sexual selection, and skin colour. Evolution and Human Behavior. 2004;25: 38–50. doi:10.1016/S1090-5138(03)00082-5

32. Harvey RG. Ecological factors in skin color variation among Papua New Guineans. Am J Phys Anthropol. 1985;66: 407–416. doi:10.1002/ajpa.1330660409

33. Carrito M de L, Santos IMB dos, Lefevre CE, Whitehead RD, Silva CF da, Perrett DI. The role of sexually dimorphic skin colour and shape in attractiveness of male faces. Evolution and Human Behavior. 2016;37: 125–133. doi:10.1016/j.evolhumbehav.2015.09.006

34. Stephen ID, Coetzee V, Perrett DI. Carotenoid and melanin pigment coloration affect perceived human health. Evolution and Human Behavior. 2011;32: 216–227. doi:10.1016/j.evolhumbehav.2010.09.003

35. Coetzee V, Greeff JM, Stephen ID, Perrett DI. Cross-cultural agreement in facial attractiveness preferences: The role of ethnicity and gender. PLoS One. 2014;9. doi:10.1371/journal.pone.0099629

36. Frost P. Different Phases of the Menstrual Cycle : Preliminary assesment of evidence for a hormonal relationship. Percept Mot Skills. 1994;79: 507–514.

37. Folstad I, Karter AJ. Parasites, bright males, and the immunocompetence handicap. American Naturalist. 1992;139: 603–622. doi:10.1086/285346

38. Rantala MJ, Moore FR, Skrinda I, Krama T, Kivleniece I, Kecko S, et al. Evidence for the stress-linked immunocompetence handicap hypothesis in humans. Nat Commun. 2012;3. doi:10.1038/ncomms1696

39. Marcinkowska UM, Rantala MJ, Lee AJ, Kozlov M V., Aavik T, Cai H, et al. Women's preferences for men's facial masculinity are strongest under favorable ecological conditions. Sci Rep. 2019;9: 1–10. doi:10.1038/s41598-019-39350-8

40. Hagiwara N, Kashy DA, Cesario J. The independent effects of skin tone and facial features on Whites' affective reactions to Blacks. J Exp Soc Psychol. 2012;48: 892–898. doi:10.1016/j.jesp.2012.02.001

41. Stepanova E v., Strube MJ. What's in a face? the role of skin tone, facial physiognomy, and color presentation mode of facial primes in affective priming effects. Journal of Social Psychology. 2012;152: 212–227. doi:10.1080/00224545.2011.597797

42. Dunham Y, Stepanova E v., Dotsch R, Todorov A. The development of race-based perceptual categorization skin color dominates early category judgments. Dev Sci. 2015;18: 469–483. doi:10.1111/desc.12228

43. Jack RE, Crivelli C, Wheatley T. Data-Driven Methods to Diversify Knowledge of Human Psychology. Trends Cogn Sci. 2018;22: 1–5. doi:10.1016/j.tics.2017.10.002

44. Torres F, Salgado M, Mackenna B, Núñez J. Who differentiates by skin color? Status attributions and skin pigmentation in Chile. Front Psychol. 2019;10: 1–12. doi:10.3389/fpsyg.2019.01516

45. Oosterhof NN, Todorov A. The functional basis of face evaluation. Proc Natl Acad Sci U S A. 2008;105: 11087–11092. doi:10.1073/pnas.0805664105

46. Stephen ID, Law Smith MJ, Stirrat MR, Perrett DI. Facial skin coloration affects perceived health of human faces. Int J Primatol. 2009;30: 845–857. doi:10.1007/s10764-009-9380-z

47. Stephen ID, Oldham FH, Perrett DI, Barton RA. Redness enhances perceived aggression, dominance and attractiveness in men's faces. Evolutionary Psychology. 2012;10: 562–572. doi:10.1177/147470491201000312

48. Borras-Guevara ML, Batres C, Perrett DI. Fear of Violence among Colombian Women Is Associated with Reduced Preferences for High-BMI Men. Human Nature. 2019;30: 341–369. doi:10.1007/s12110-019-09350-8

49. Carré JM, Archer J. Testosterone and human behavior: the role of individual and contextual variables. Curr Opin Psychol. 2018;19: 149–153. doi:10.1016/j.copsyc.2017.03.021

50. Geniole SN, Bird BM, McVittie JS, Purcell RB, Archer J, Carré JM. Is testosterone linked to human aggression? A meta-analytic examination of the relationship between baseline, dynamic, and manipulated testosterone on human aggression. Horm Behav. 2020;123: 104644. doi:10.1016/j.yhbeh.2019.104644

51. Puts DA, Welling LLM, Burriss RP, Dawood K. Men's masculinity and attractiveness predict their female partners' reported orgasm frequency and timing. Evolution and Human Behavior. 2012;33: 1–9. doi:10.1016/j.evolhumbehav.2011.03.003

52. Foo YZ, Rhodes G, Simmons LW. The carotenoid beta-carotene enhances facial color, attractiveness and perceived health, but not actual health, in humans. Behavioral Ecology. 2017;28: 570–578. doi:10.1093/beheco/arw188

53. Kleisner K, Kočnar T, Tureček P, Stella D, Akoko RM, Třebický V, et al. African and European perception of African female attractiveness. Evolution and Human Behavior. 2017;38: 744–755. doi:10.1016/j.evolhumbehav.2017.07.002

54. Alaluf S, Atkins D, Barrett K, Blount M, Carter N, Heath A. The impact of epidermal melanin on objective measurements of human skin colour. Pigment Cell Res. 2002;15: 119–126. doi:10.1034/j.1600-0749.2002.1o072.x

55. Visscher MO. Skin Color and Pigmentation in Ethnic Skin. Facial Plast Surg Clin North Am. 2017;25: 119–125. doi:10.1016/j.fsc.2016.08.011

56. del Bino S, Bernerd F. Variations in skin colour and the biological consequences of ultraviolet radiation exposure. British Journal of Dermatology. 2013;169: 33–40. doi:10.1111/bjd.12529

57. Tiddeman B, Perrett D. Transformation of dynamic facial image sequences using static 2D prototypes. Visual Computer. 2002;18: 218–225. doi:10.1007/s003710100142

58. Coetzee V, Faerber SJ, Greeff JM, Lefevre CE, Re DE, Perrett DI. African Perceptions of Female Attractiveness. PLoS One. 2012;7: 3–8. doi:10.1371/journal.pone.0048116

59. Fink B, Grammer K, Matts PJ. Visible skin color distribution plays a role in the perception of age, attractiveness, and health in female faces. Evolution and Human Behavior. 2006;27: 433–442. doi:10.1016/j.evolhumbehav.2006.08.007

60. Lefevre CE, Perrett DI. Fruit over sunbed: Carotenoid skin colouration is found more attractive than melanin colouration. Quarterly Journal of Experimental Psychology. 2015;68: 284–293. doi:10.1080/17470218.2014.944194

61. Paschetta C, de Azevedo S, Ramallo V, Cintas C, Pérez O, Navarro P, et al. The impact of socioeconomic and phenotypic traits on self-perception of ethnicity in Latin America. Sci Rep. 2021;11: 1–11. doi:10.1038/s41598-021-92061-x

62. Telles EE. Pigmentocracies: Ethnicity, Race and Color in Latin America. North Carolina: UNC Press Book; 2014.

63. Bolker BM, Brooks ME, Clark CJ, Geange SW, Poulsen JR, Stevens MHH, et al. Generalized linear mixed models: a practical guide for ecology and evolution. Trends Ecol Evol. 2009;24: 127–135. doi:10.1016/j.tree.2008.10.008

64. Burnham KP, Anderson DR. Multimodel inference: Understanding AIC and BIC in model selection. Sociol Methods Res. 2004;33: 261–304. doi:10.1177/0049124104268644

65. Zuur AF, Ieno EN, Walker NJ, Saveliev AA, Smith GM. Mixed Effects Models and Extensions in Ecology with R. NewYork: Springer; 2009.

66. Zuur AF, Ieno EN. A protocol for conducting and presenting results of regression-type analyses. Methods Ecol Evol. 2016;7: 636–645. doi:10.1111/2041-210X.12577

67. Bates D, Mächler M, Bolker BM, Walker SC. Fitting linear mixed-effects models using lme4. J Stat Softw. 2015;67. doi:10.18637/jss.v067.i01

68. Bolker M ben. Package ‘ bbmle .’ 2021.

69. R Core Team. R: A language and environment for statistical computing. Vienna, Austria: R Foundation for Statistical Computing.; 2020.

70. Oosterhof NN, Todorov A. The functional basis of face evaluation. Proc Natl Acad Sci U S A. 2008;105: 11087–11092. doi:10.1073/pnas.0805664105

71. Dixon AR. Colorism and classism confounded: Perceptions of discrimination in Latin America. Soc Sci Res. 2019;79: 32–55. doi:10.1016/j.ssresearch.2018.12.019

72. Kleisner K, Tureček P, Roberts SC, Havlíček J, Valentova JV, Akoko RM, et al. How and why patterns of sexual dimorphism in human faces vary across the world. Sci Rep. 2021;11: 1–14. doi:10.1038/s41598-021-85402-3

73. Puts DA. Human sexual selection. Curr Opin Psychol. 2016;7: 28–32. doi:10.1016/j.copsyc.2015.07.011

74. Puts DA. Beauty and the beast: Mechanisms of sexual selection in humans. Evolution and Human Behavior. 2010;31: 157–175. doi:10.1016/j.evolhumbehav.2010.02.005

75. Windhager S, Schaefer K, Fink B. Geometric morphometrics of male facial shape in relation to physical strength and perceived attractiveness, dominance, and masculinity. American Journal of Human Biology. 2011;23: 805–814. doi:10.1002/ajhb.21219

76. Puts DA, Apicella CL, Cárdenas RA. Masculine voices signal men’s threat potential in forager and industrial societies. Proceedings of the Royal Society B: Biological Sciences. 2012;279: 601–609. doi:10.1098/rspb.2011.0829

77. Rosenfield KA, Sorokowska A, Sorokowski P, Puts DA. Sexual selection for low male voice pitch among Amazonian forager-horticulturists. Evolution and Human Behavior. 2020;41: 3–11. doi:10.1016/j.evolhumbehav.2019.07.002

78. Batres C, Re DE, Perrett DI. Influence of Perceived Height, Masculinity, and Age on Each Other and on Perceptions of Dominance in Male Faces. Perception. 2015;44: 1293–1309. doi:10.1177/0301006615596898

79. Waller BM, Kavanagh E, Micheletta J, Clark PR, Whitehouse J. The face is central to primate multicomponent signals. Int J Primatol. 2022. doi:10.1007/s10764-021-00260-0

80. Karczmarek P, Pedrycz W, Kiersztyn A, Rutka P. A study in facial features saliency in face recognition: an analytic hierarchy process approach. Soft comput. 2017;21: 7503–7517. doi:10.1007/s00500-016-2305-9

81. Rotshtein P, Geng JJ, Driver J, Dolan RJ. Role of features and second-order spatial relations in face discrimination, face recognition, and individual face skills: Behavioral and functional magnetic resonance imaging data. J Cogn Neurosci. 2007;19: 1435–1452. doi:10.1162/jocn.2007.19.9.1435

82. Scott IML, Pound N, Stephen ID, Clark AP, Penton-Voak IS. Does masculinity matter? The contribution of masculine face shape to male attractiveness in humans. PLoS One. 2010;5. doi:10.1371/journal.pone.0013585

83. DeBruine LM. Trustworthy but not lust-worthy: Context-specific effects of facial resemblance. Proceedings of the Royal Society B: Biological Sciences. 2005;272: 919–922. doi:10.1098/rspb.2004.3003

84. Mogilski JK, Welling LLM. The relative contribution of jawbone and cheekbone prominence, eyebrow thickness, eye size, and face length to evaluations of facial masculinity and attractiveness: A conjoint data-driven approach. Front Psychol. 2018;9: 1–11. doi:10.3389/fpsyg.2018.02428

85. Torrance JS, Wincenciak J, Hahn AC, DeBruine LM, Jones BC. The relative contributions of facial shape and surface information to perceptions of attractiveness and dominance. PLoS One. 2014;9. doi:10.1371/journal.pone.0104415

86. Třebický V, Saribay SA, Kleisner K, Akoko RM, Kočnar T, Valentova JV, et al. Cross-Cultural Evidence for Apparent Racial Outgroup Advantage: Congruence between Perceived Facial Aggressiveness and Fighting Success. Sci Rep. 2018;8: 1–7. doi:10.1038/s41598-018-27751-0

87. Mitteroecker P, Windhager S, Müller GB, Schaefer K. The morphometrics of “masculinity” in human faces. PLoS One. 2015;10: 1–13. doi:10.1371/journal.pone.0118374

88. Windhager S, Schaefer K, Fink B. Geometric morphometrics of male facial shape in relation to physical strength and perceived attractiveness, dominance, and masculinity. American Journal of Human Biology. 2011;23: 805–814. doi:10.1002/ajhb.21219

89. Russell R. Sex, beauty, and the relative luminance of facial features. Perception. 2003;32: 1093–1107. doi:10.1068/p5101

90. Oh DW, Dotsch R, Todorov A. Contributions of shape and reflectance information to social judgments from faces. Vision Res. 2019;165: 131–142. doi:10.1016/j.visres.2019.10.010

91. Han C, Wang H, Hahn AC, Fisher CI, Kandrik M, Fasolt V, et al. Cultural differences in preferences for facial coloration. Evolution and Human Behavior. 2018;39: 154–159. doi:10.1016/j.evolhumbehav.2017.11.005

92. Stephen ID, Scott IML, Coetzee V, Pound N, Perrett DI, Penton-Voak IS. Cross-cultural effects of color, but not morphological masculinity, on perceived attractiveness of men’s faces. Evolution and Human Behavior. 2012;33: 260–267. doi:10.1016/j.evolhumbehav.2011.10.003

93. Whitehead RD, Re D, Xiao D, Ozakinci G, Perrett DI. You are what you eat: Within-subject increases in fruit and vegetable consumption confer beneficial skin-color changes. PLoS One. 2012;7: 1–9. doi:10.1371/journal.pone.0032988

94. Whitehead RD, Ozakinci gözde, Perrett DI. Attractive skin coloration: Harnessing sexual selection to improve diet and health. Evolutionary Psychology. 2012;10: 842–854. doi:10.1177/147470491201000507

95. Rhodes G, Lee K, Palermo R, Weiss M, Yoshikawa S, Clissa P, et al. Attractiveness of own-race, other-race, and mixed-race faces. Perception. 2005;34: 319–340. doi:10.1068/p5191

96. INEGI. ENCUESTA NACIONAL SOBRE DISPONIBILIDAD Y USO DE TECNOLOGÍAS DE LA INFORMACIÓN EN LOS HOGARES (ENDUTIH) 2021. Encuesta Nacional Sobre Disponibilidad Y Uso De Tecnologías De La Información En Los Hogares (Endutih) 20211. 2022; 1–19.

97. INEGI. Los extranjeros en México. XII Censo General de Población y Vivienda, 2000. 2014; 67.

98. Flores RD, Vignau Loría M, Casas RM, Loria V, Martínez-Casas R. Transitory vs. durable boundary crossing: What explains the indigenous population boom in Mexico? 2021.

99. Villarreal A. Ethnic identification and its consequences for measuring inequality in Mexico. Am Sociol Rev. 2014;79: 775–806. doi:10.1177/0003122414541960

Supporting information

S1 Fig. Participant's self-perceived skin color ratings. PERLA scale distribution of the 700 participant's own skin color ratings. The color levels go from 1 to 10, being 1 the lightest and 10 the darkest. 11 (1.6 %) picked skin tone level 1, 36 (5.1%) skin tone level 2, 42 (6%) skin tone level 3, 240 (34.3%) skin tone level 4, 201 (28.8%) skin tone level 5, 102 (14.6%) skin tone level 6, 45 (6.4%) skin tone level 7, 14 (2%) skin tone level 8, 7 (1%) skin tone level 9, and 1 (0.1%) skin tone level 10.

S1 Table. Perception estimates with 95% confidence intervals with all participants included. Estimates (Est.) with 95% confidence intervals (CI) of the fixed predictors of the models for: (a) Perceived attractiveness, (b) Perceived trustworthiness, (c) Perceived health, (d) Perceived dominance, (e) Perceived aggressiveness, and (f) Perceived masculinity. *Note:* (□*)* The dominance best fitted model did not include the skin color transformation predictor, therefore the estimates shown represent the main effect of the natural skin color group only.

S2 Table. Pearson's correlation analysis between the scores of all perceptions rates of the natural versions only. Pearson's correlation between the natural versions scores of attractiveness, trustworthiness, perceived health, dominance, masculinity, and aggressiveness. *p<0.05, **p<0.01, ***p<0.001.

Figure

Click here to access/download;Figure;Fig1.tif

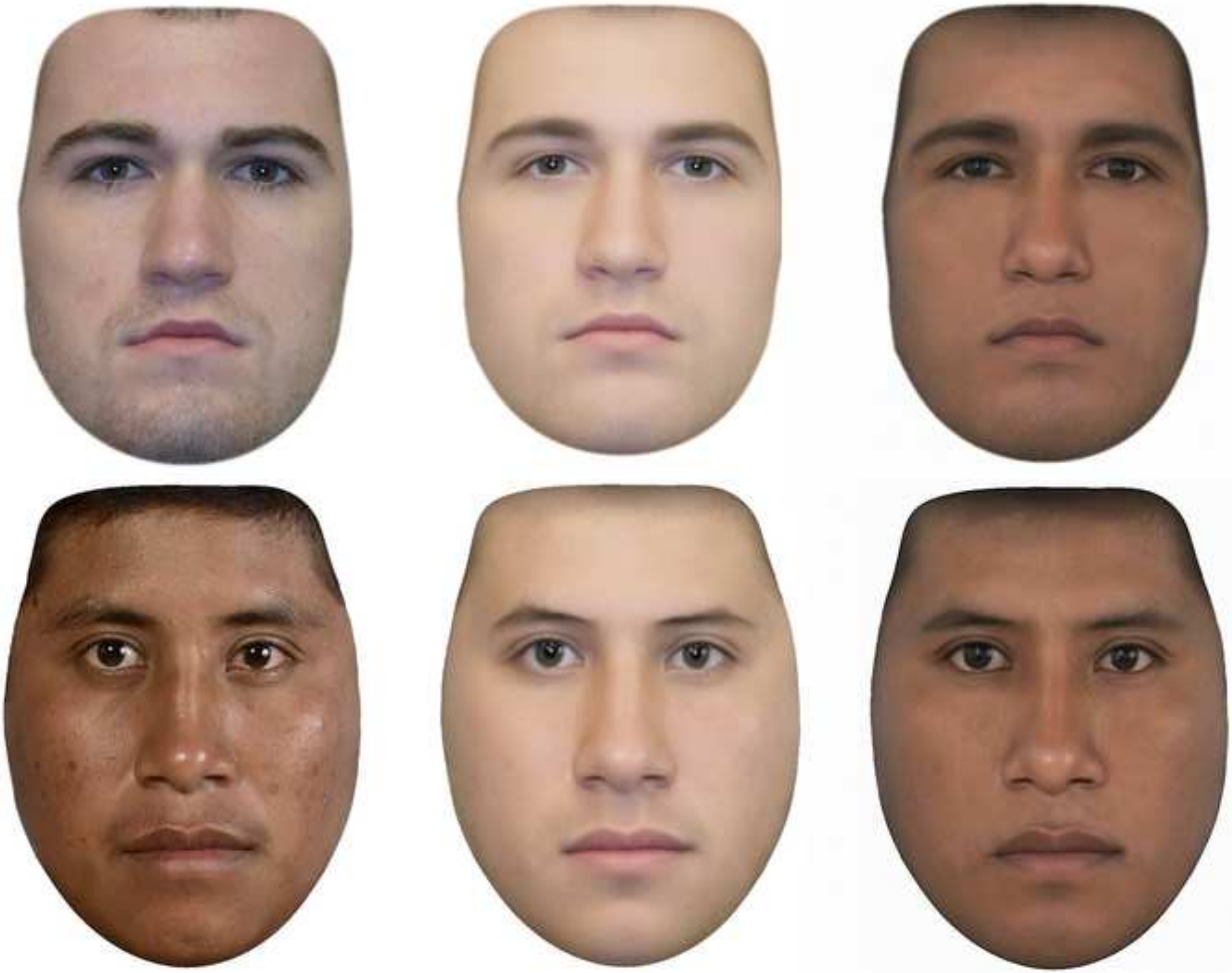

 Click here to access/download;Figure;Fig2.tif

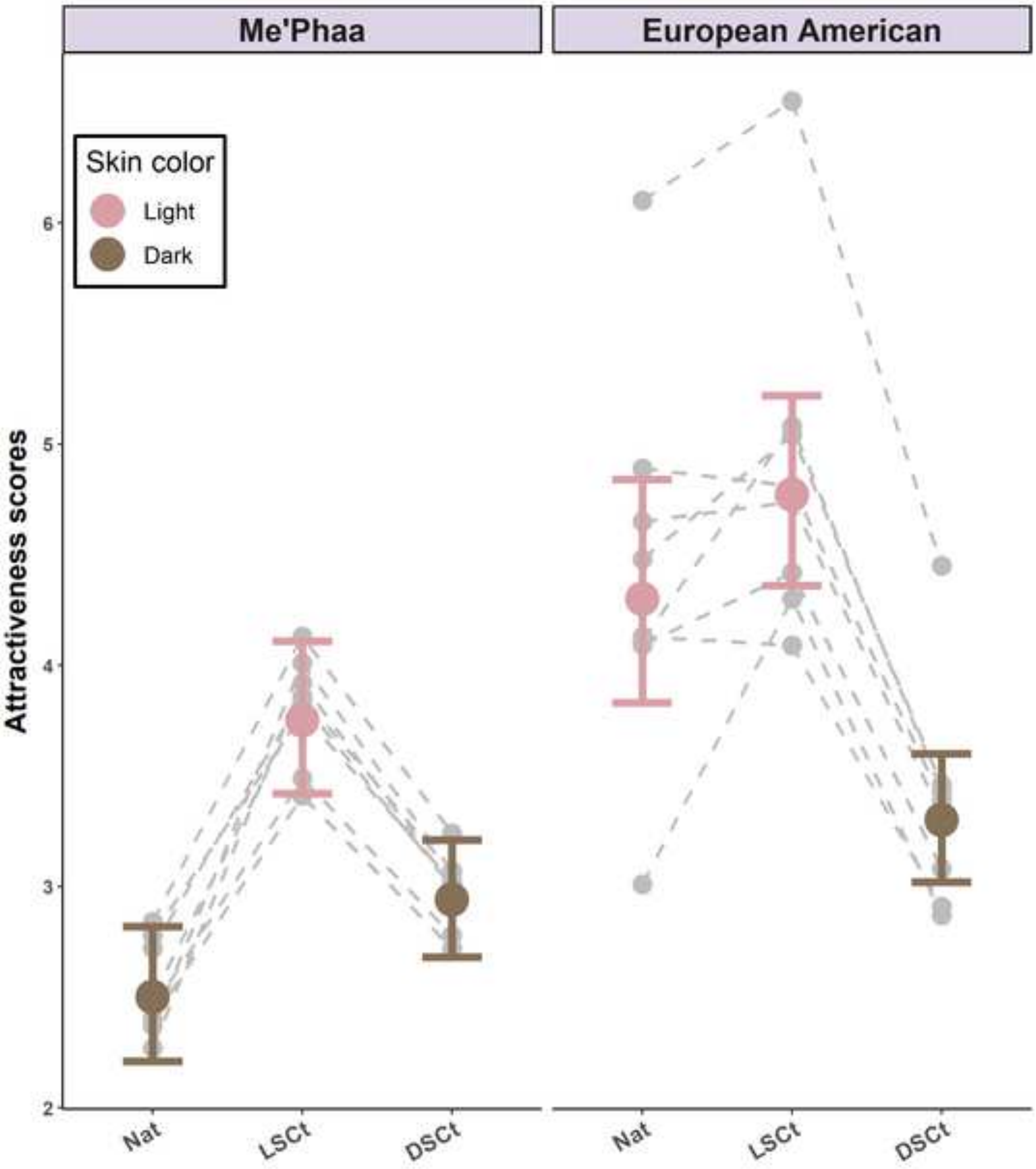

Figure
Click here to access/download;Figure;Fig3.tif

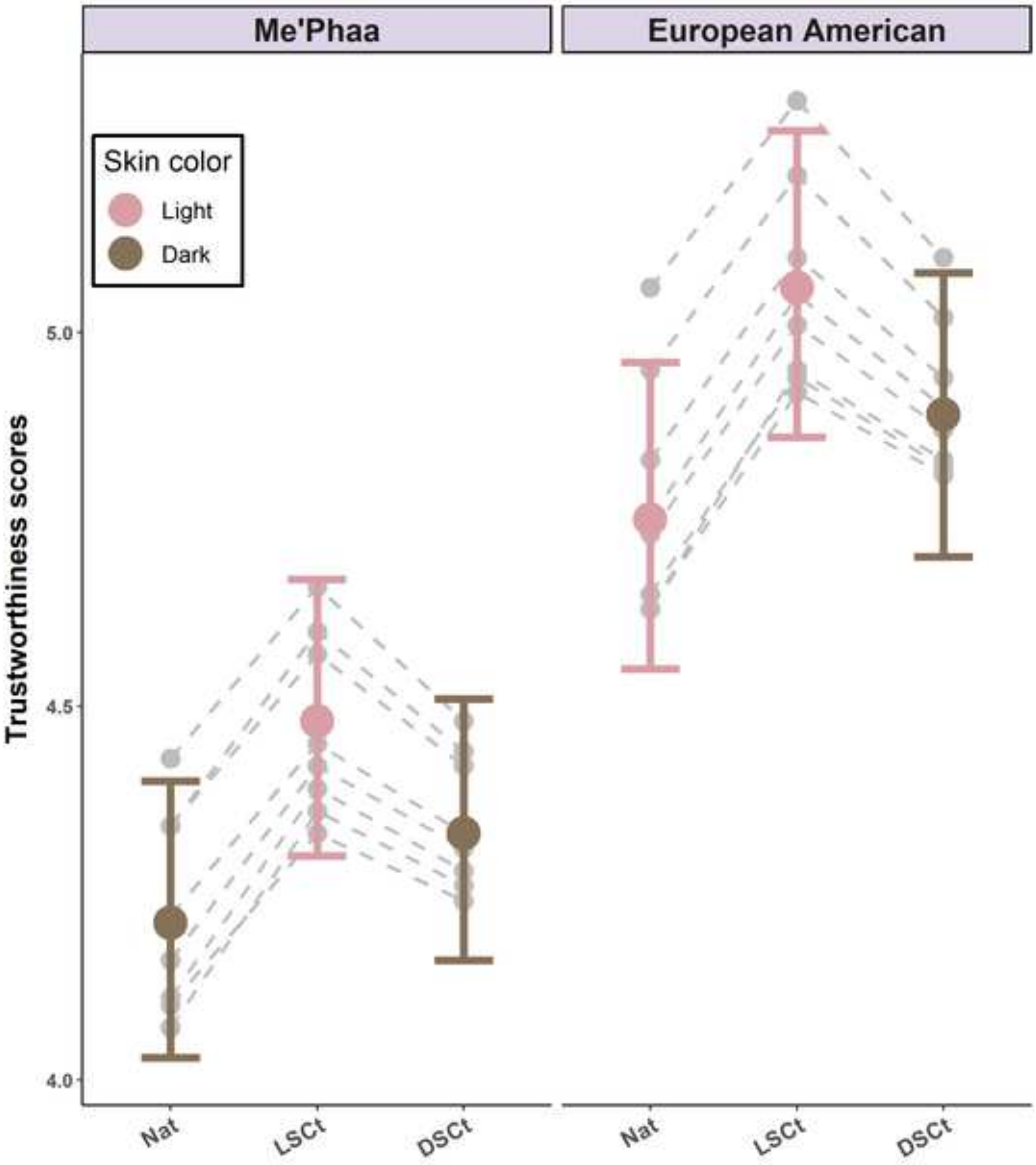

Figure Click here to access/download;Figure;Fig4.tif

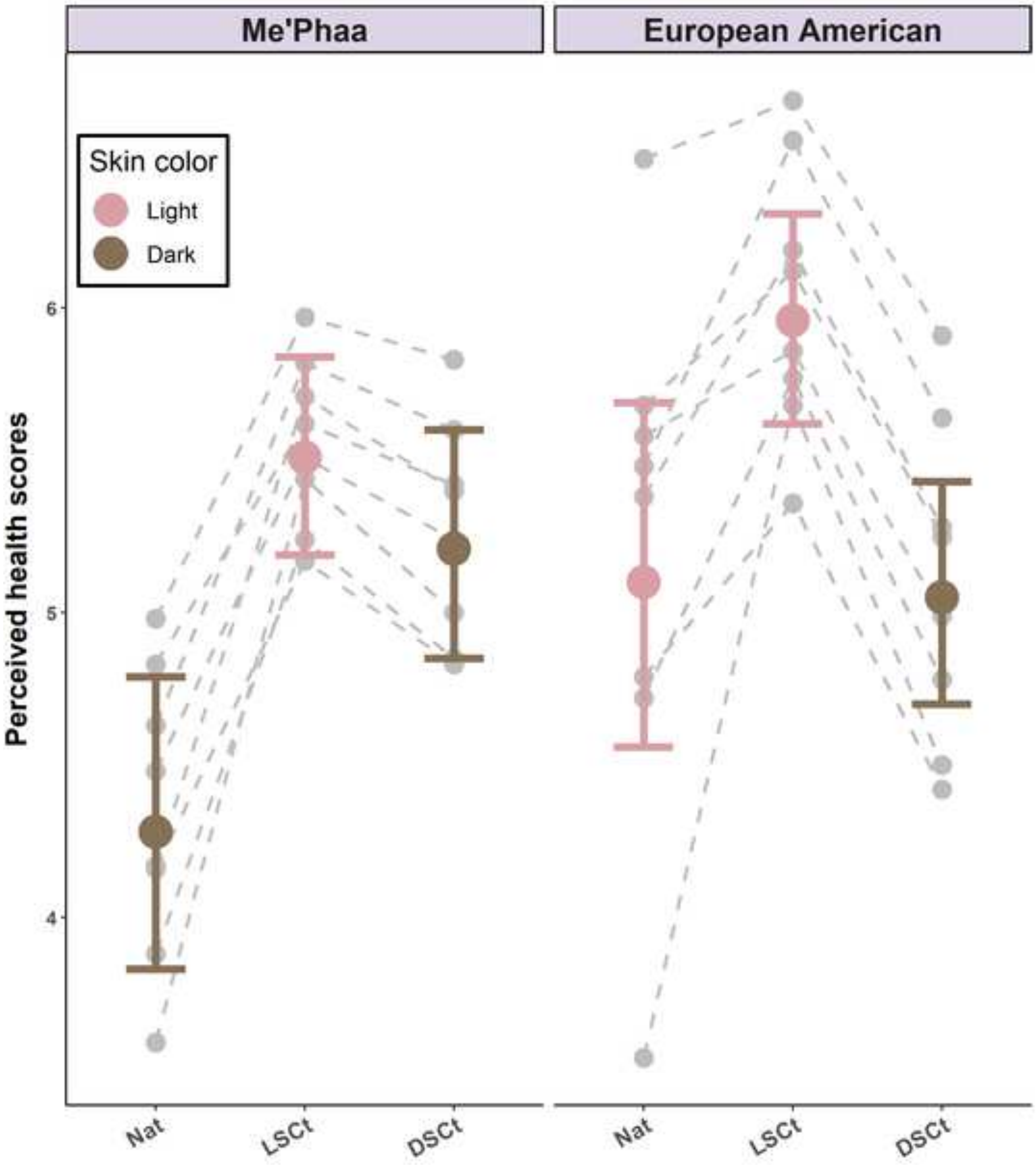

Figure

Click here to access/download;Figure;Fig5.tif

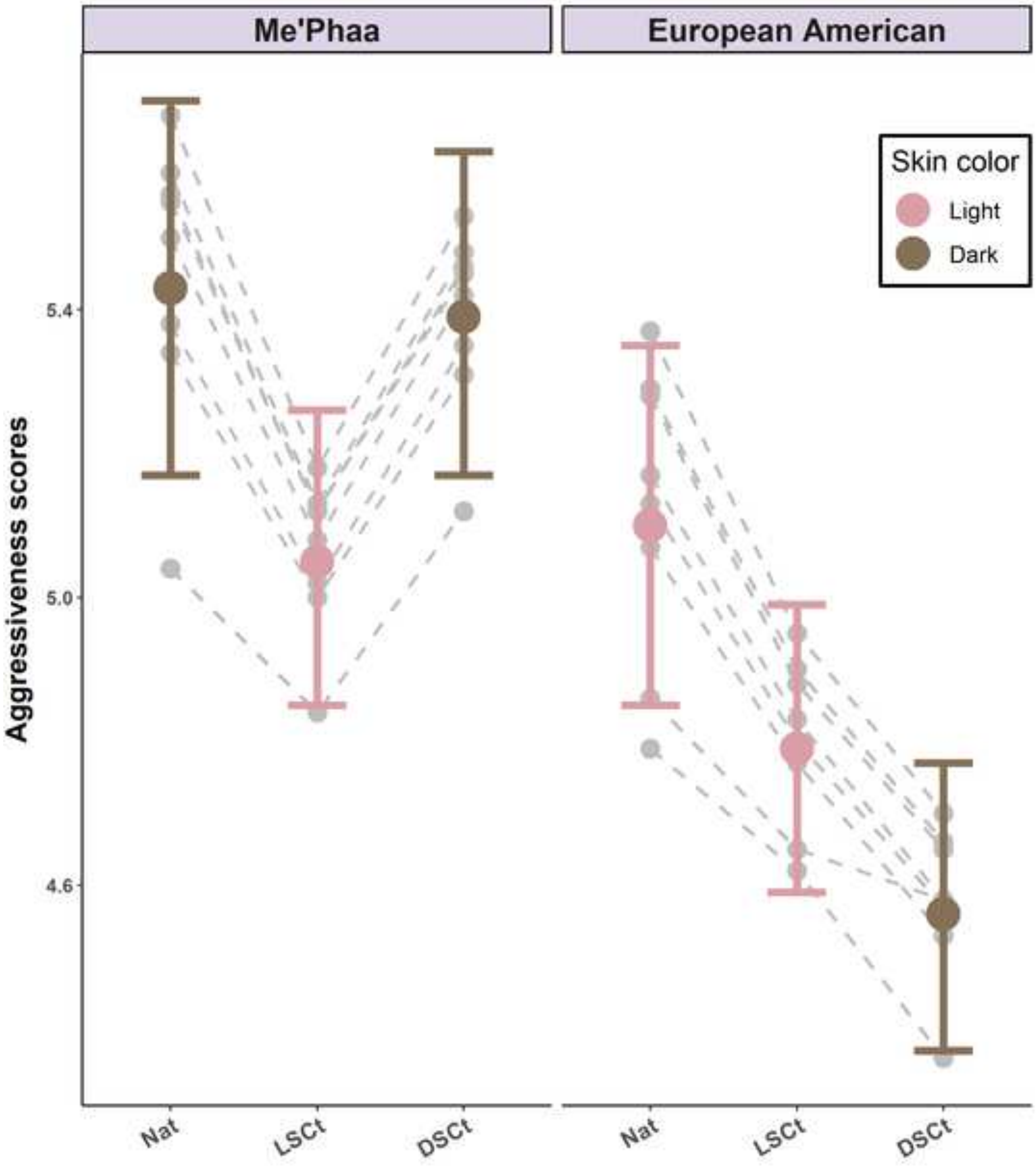

 Click here to access/download;Figure;Fig6.tif

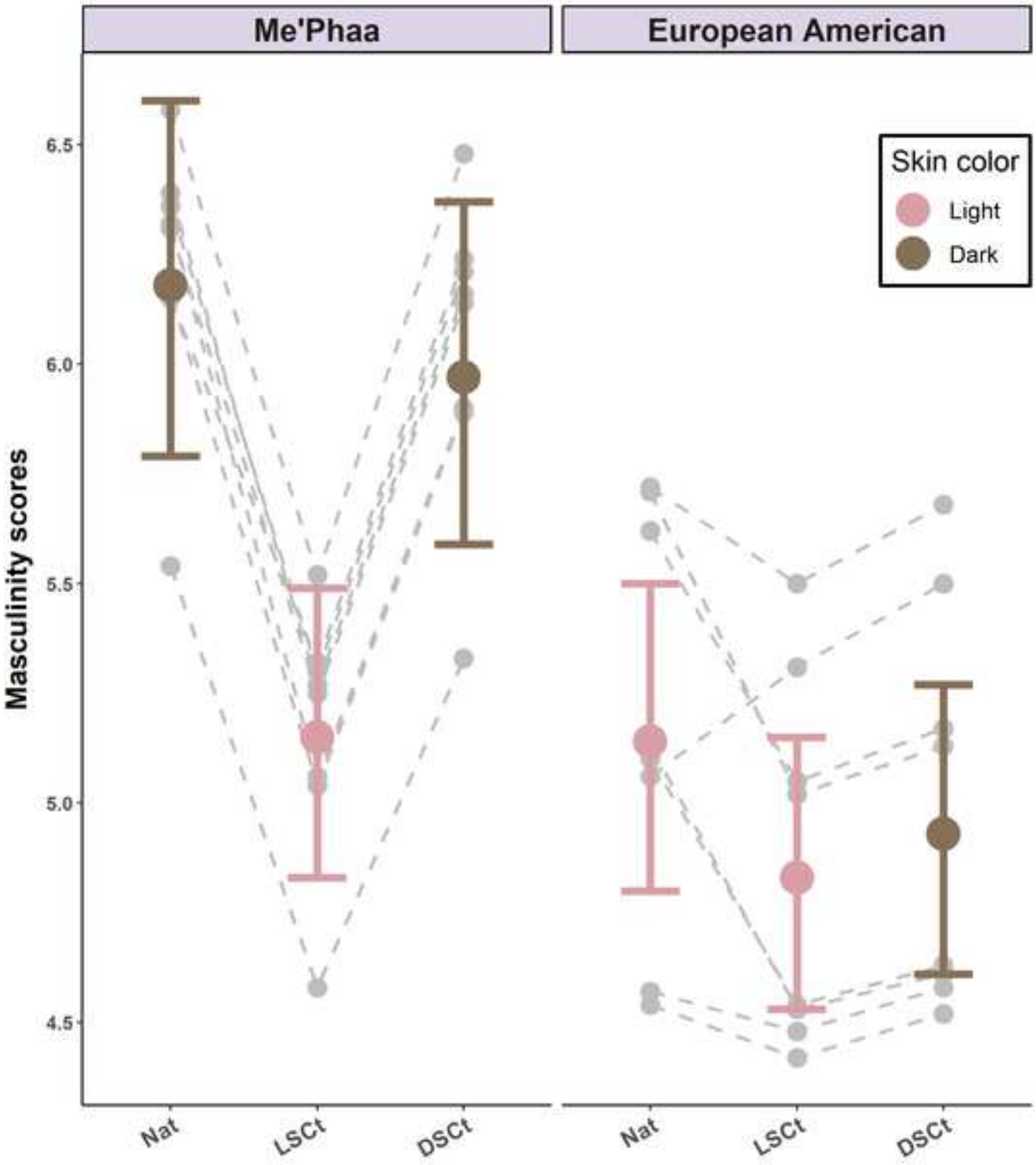

Click here to access/download
Supporting Information
S1_Fig.pdf

Click here to access/download
Supporting Information
S1_Table.pdf

Click here to access/download
Supporting Information
S2_Table.pdf

Effects of facial skin pigmentation on social judgments in a Mexican population

Jaaziel Martínez-Ramírez[1] , David Puts[2] , Javier Nieto[3] , Isaac G-Santoyo[1,4*]

1. Neuroecology lab, Faculty of Psychology, National Autonomous University of Mexico, Mexico City, Mexico.
2. Department of Anthropology, Pennsylvania State University, Pennsylvania, United States of America.
3. Laboratory of Learning and Adaptation, Faculty of Psychology, National Autonomous University of Mexico, Mexico City, Mexico.
4. Unidad de Investigación en Psicobiología y Neurociencias, National Autonomous University of Mexico, Mexico City, Mexico.

* Corresponding author

E-mail: isantoyo@psicologia.unam.mx (IG-S)

Abstract

People quickly and involuntarily form impressions of others based on their facial physical attributes, which can modulate critical social interactions. Skin pigmentation is one of the most variable and conspicuous facial traits among human populations. Empirical evidence suggests that these variations reflect ancestral ecological selective pressures balancing cutaneous vitamin D synthesis with the protection of the dermis from ultraviolet radiation. Nevertheless, skin pigmentation may currently be subject to additional selective pressures. For instance, the colonial era in Central and South America developed a highly stratified society based on ethnic origins, and light skin pigmentation became associated with higher social status and deference. This association could have originated through historical social learning that promoted favorable social perceptions towards individuals with lighter skin color and unfavorable perceptions towards individuals with darker skin color, which could still be present in the perception of current populations.

Facial skin pigmentation is also sexually dimorphic, with males tending to exhibit darker skin than females, a difference that could be driven by sexual selection. To explore whether social learning and sexual selection represent additional selective pressures on skin pigmentation, we tested how this facial trait influences fundamental social perceptions in a Mexican population (N = 700, 489 female). We sampled facial images of eight European American males with natural lighter facial skin and eight males from an indigenous pre-Columbian community from Mexico, the Me'Phaa, with natural darker facial skin. We produced stimuli from these images by varying the skin pigmentation while preserving the facial shape. Stimuli were rated on attractiveness, trustworthiness, perceived health, dominance, aggressiveness, and femininity/masculinity. We found that the natural light-

skinned faces were perceived as more attractive, trustworthy, and healthy but less dominant than the natural dark faces. Furthermore, by varying the facial skin color in these original groups, we altered the perceptions of them, mainly their attractiveness. These results partially support the hypothesis that dark facial skin color may help males compete for mates. Also, the results strongly support the view that lighter facial skin color became associated with social benefits through social learning in this Mexican population. Our findings, when viewed through the lens of cultural evolution, align with previous research in social psychology and anthropology. They hold the potential to offer a comprehensive understanding of the origin of this social phenomenon of cultural transmission, which currently plays a role in the formation of racial attitudes, stereotyping, and racial inequality in Mexican and other Latin American populations.

Introduction

Human faces provide rich information about salient attributes, such as mate quality [1,2], social dominance [3], and fecundity [4]. Hence, people quickly and involuntarily form impressions of others based on their facial appearance [5,6], which in turn may influence important social outcomes [7,8]. Skin pigmentation is one of the most conspicuous and variable facial traits of human populations [9]. The main pigments—melanin, hemoglobin, and carotenoids—absorb and reflect different wavelengths of light within the visible spectrum, and collagen also contributes by scattering light [10,11]. The skin's pigments vary not only between individuals and populations but also between men and women, perhaps because of sexual selection [12]. Natural selection initially favored

darker skin as a mean to protect against the harmful effects of UV radiation in the tropics. The pressure of selection then shifted toward lighter skin among those humans who spread into higher latitudes with lower levels of UVR [13–15]. Melanin, the principal skin pigment that provides this gradient of light to dark color, acts as a photoprotective filter that reduces the penetration of all wavelengths of light into subepidermal tissue. Under the high UVA levels near the equator, dark skin pigmentation is particularly advantageous for protecting folate and reducing the risk of fatal birth defects. Human migration beyond the tropics reduced the selective advantage of this photoprotective property and selected instead for lower melanization to facilitate cutaneous vitamin D biosynthesis, critical to the regulation of many biological processes [16]. Although these events are widely accepted as the main evolutionary events generating human skin pigment variation, this product of natural selection could be currently subjected to other ecological pressures in addition to those that caused its origin.

Cultural transmission is the main means for passing on adaptative behaviors that improves one´s chances for survival and reproduction [17]. It is especially adaptive if a transmitted behavior saves learners the costs of individual learning through trial and error. For instance, cultural transmission can influence mate preferences through two different mechanisms [18]; by reinforcing a preference for a trait that may signal biological quality if that preference significantly contributes to reproductive success, such as offspring quality, or by promoting new preferences for traits that do not appear to have direct links to the bearer's biological quality [19]. In this case, new social preferences may be selected by cultural transmission if the novel trait positively influences social benefits in a particular population [20].

The colonial period in Mexico and all countries of Central and South America led to a highly stratified society based on ethnic origins. During the 300 years of that period, light skin pigmentation, usually present in Spanish colonizers, was one of the most conspicuous phenotypic traits associated with higher social status and hence access to social benefits, such as education and wealth [21]. This racial/color hierarchy system, known as *castes*, placed the European colonizers at the top of the social hierarchy and the indigenous populations at the bottom, it developed deep roots in Latin American society, and even though the *caste* system was officially abolished, there is robust evidence of social stratification based on skin color in contemporary Mexican society [22], which can be accompanied by racial attitudes, stereotyping and racial inequality [22].

Recent studies have shown that people with darker skin have, on average, lower educational levels, occupational status, and hourly earnings, are more likely to live in poverty, and have fewer chances of social mobility than do people with lighter skin; these differences are significant even after controlling for sociodemographic factors such as age, gender, and parental education [23–25]. In addition, a cultural preference for light skin and other European phenotypical features is also influenced by social media in Western societies, which might reinforce the association between skin color and social status. Hence, if light skin is culturally preferred because it is associated with social benefits, prestige, or deference, a new skin pigmentation signal may have emerged through an extension of historical, sociocultural learning.

Another non-mutually exclusive hypothesis is that facial skin pigmentation could be driven by perceptions shaped by sexual selection. Human skin pigmentation is a sexually dimorphic trait in the sense that men usually have darker skin pigmentation than women

[26–28]. The sex difference is due to exposure of skin tissues to differing ratios of androgens to estrogens, particularly at puberty. Testosterone has a stronger effect than estrogen on melanin synthesis and vascularization of the upper dermis [29–31]. In addition, males develop a more intense facultative pigmentation after sun exposure and retain it for longer periods than females whereas female skin lightens faster after a reduction in sun exposure [32]. This sexual dimorphism might influence sexual preferences, since several studies have shown that darker skin in men is perceived as more attractive [33–36]. Therefore, this female preference for darker skin color could reflect ancestral selection pressures shaping perceptions of an androgen-dependent trait. In this case, the basis of this preference may be the honest signaling of mate quality in terms of male immune function (i.e., immunocompetence handicap hypothesis), vigor, or other physiological costs of developing this trait [37–39].

Although the effects of skin pigmentation on social perceptions is an active area of research in social psychology, prior research has focused mainly on racial attitudes and generally does not employ an evolutionary framework. For instance, Hagiwara et al. [40] evaluated affective reactions and other social attributions towards African American men by manipulating facial shape features (nose and lips) while controlling for skin tone. Similar studies have examined the role of skin tone and facial shape features in racial attitudes through similar methods [41,42]. Moreover, most experimental studies of facial perception have been carried out on participants from WEIRD (Western, educated, industrialized, rich, and democratic) societies, thus limiting the generalizability of findings [43]. Finally, prior studies in non-WEIRD Latin American samples have implemented a

social theoretical framework with a non-experimental design to explore correlations between skin tone and social attributes, such as status or labor hierarchy [44].

In this study, we used an experimental design with a Mexican population to determine whether facial skin color can influence certain social perceptions: attractiveness; attractiveness, trustworthiness, perceived health, dominance, aggressiveness, and femininity/masculinity [45–47]. To that end, we performed a crossover experiment on men's faces, selecting two groups of faces with contrasting innate skin pigmentation: the first composed of faces with natural light facial skin tone, selected from a European American population, and the second composed of faces with a darker facial skin tone, selected from a Mexican pre-Columbian population, self-identified as Me'Phaa. Using these two groups, we created six sets of facial stimuli by varying the natural skin pigmentation while preserving the facial shape. Finally, we presented all stimuli to be evaluated in the six social dimensions previously mentioned.

We hypothesize that if social learning is acting on the perceptions of facial skin pigmentation, we expect that the lighter-skinned group would be perceived as more attractive, more trustworthy, and healthier because positive evaluations of these attributes strongly promote pro-sociality and social benefits, but less dominant and less aggressive because dominance and aggressiveness, in contrast, are considered to be potentially harmful and may lead to avoidance of social interactions [48]. However, if skin pigmentation more strongly reflects ancestral selection shaping perceptions of an androgen-dependent trait, then we would expect that faces with darker skin would be perceived not only as more attractive and healthier but also as more dominant, aggressive, and masculine due to the association between these last three attributes and the behavioral and

physiological effects of androgens [49,50]. In both cases, if differences in skin pigmentation are driving these effects, we expect that the experimental manipulation of facial skin color would change the previous patterns in the perceptions of both the European American and Me´Phaa faces. Finally, if neither light nor dark skin color affects any social perception, of the original faces and the experimentally exchanged stimuli, then skin color would play no role in either social selection or the sexual selection of dimorphic traits.

Material and methods

Participant raters

A total of 489 Mexican adult women (mean age = 25.4 years, SD = 8.6 years) and 211 Mexican adult men (mean age = 26.5 years, SD = 9.1 years) participated as raters in this study. Self-reported ethnicities were 84.0 % Mestizo, 6.3 % European, 3.9 % Indigenous (particular groups not specified), 0.3 % African, and 6.3 % Other. A total of 485 participants reported living in Mexico City, 95 in the surrounded metropolitan area, and the rest in different states of Mexico. All the raters were recruited online through a board advertisement from the Department of Psychology at the National Autonomous University of Mexico.

Skin color facial stimuli

We first created two contrasting light and dark facial skin prototypes by selecting 30 facial photographs of young European American men and 30 facial photographs of young Me’Phaa Mexican Indigenous men from larger photograph sets. (See below the criteria of

selection for both groups of photographs). The European American faces were selected from a set of facial photographs of the Department of Anthropology at Pennsylvania State University [51]. Facial photographs were taken from a distance of 2 m using a 12-megapixel Olympus E-300 camera with a mounted flash at a resolution of 1200 × 1000 pixels. The facial photographs of the Me'Phaa were selected from a collection of facial photographs from the NeuroEcology Lab at the National Autonomous University of Mexico. These facial photographs were taken from a distance of 2m using a 21-megapixel Nikon 5500 camera illuminated by two studio strobes (Lusana Studio, 400w) with reflective umbrellas. The strobes were placed 1.85 m from the target on each side and lights were arranged at 40° angles. In both sets of facial photographs participants were asked to remove any earrings, glasses, or facial jewelry, and to maintain a neutral expression.

The two groups of facial photographs (European American and Me´Phaa) were selected in order to obtain two samples of faces whose pigmentation was contrasting enough to avoid an overlapping effect: one group with lighter skin and color ranges typical for the European American average, and another group with darker skin and color ranges typical for the Indigenous Mexican average, and because both skin colors are within the typical range of colors found in Mexican population. Therefore, in order to evaluate if the facial photographs in both groups met these criteria, we measured the skin color values in CIELab color space. Following procedures reported in previous studies [52,53], we first standardized the facial images by color calibration, then we took color samples of three separate patches located on the forehead and on each cheek and calculated the average CIELab values using Photoshop CS6. Lightness (L^*) value was the parameter that change the most between the ethnic groups we sampled [54].Therefore, we focused on this

parameter a representative value of skin color differences between the two selected groups. Here, European American (*L** mean = 61.08, SD = 6.42) and Me'Phaa (*L** mean = 49.84, SD = 4.05) mean lightness were statistically different (t (58) = -8.07, $p < 0.01$), and lightness values for both groups were in the ranges of White/European and Hispanic ethnicities reported in previous studies [55,56].

From the 60 facial photographs selected, we created two composite faces that averaged the skin color of the 30 original faces from each group using the software Psychomorph [57]. The averaged composite face of the European American men was the light skin prototype, while the composite face of the Me'Phaa men was the dark skin prototype. Next, we randomly selected eight faces from the European American men's set and eight from the Me'Phaa men's set and created two different versions of each face, one displaying light skin and the one displaying dark skin. These two versions were created by adding or subtracting 70% of the skin color differences between the light and dark facial skin prototypes previously created while leaving facial shape unchanged. Finally, we repeated the skin color measurement process (see above) in both groups of the new composite faces (light skin color transformed and dark skin color transformed faces). The average lightness value was *L** = 66.74, SD = 1.37 in both light skin color transformed versions (i.e. Natural Me´phaa and Natural European American faces) and *L** = 52.75, SD = 2.58 in both dark skin color transformed versions. The method we used for skin color manipulation slightly increased the lightness values with respect to the values of the natural faces, the increases were not statistically significant for any group (t (44) = -0.52, $p = 0.6$; t (44) = -0.43, $p = 0.6$), and both groups stayed within the ranges reported for White/European and Hispanic ethnicities in previous studies [55,56]. At the end of this

process, we obtained three versions for each of the eight European American and eight Me'Phaa faces: a natural unmodified version (NAT), a light skin color transformed version (LSCt), and a dark skin color transformed version (DSCt; Fig. 1), for a total of 48 facial stimuli.

Fig. 1. Example stimuli. Three versions of the same facial stimuli of a European American male (up) and a Me'Phaa male (bottom). Natural unmodified versions (left), Light Skin Color transformed versions (middle), and Dark Skin Color transformed versions (right).

Facial perception rates

To obtain the ratings, we divided the 48 stimuli into eight sets of six stimuli each. The stimuli were assigned to each set counterbalancing by group (European American and Me'Phaa) and version (NAT, LSCt, and DSCt) and we made sure that two or more versions of the same face were not included together in the same set. Given that the experimental transformation tended to homogenize facial skin color and texture, two features that affect perceptions of human faces [58,59], we decided to include the natural version of each face (NAT) to be able to compare the ratings of the natural versions with the ratings of the concordant skin color transformed version; that is, we compared the Natural versions with the Light skin color transformed versions in the European American faces and the Natural versions with the dark skin color transformed versions in the Me'Phaa faces. By doing this we could measure the effect of the color and texture homogenization.

The facial evaluations were performed online at the webpage labneuroecologiacog.com. Each participant was randomly assigned to one of the eight facial stimuli sets and was asked to rate each face separately on a 9-point Likert scale according to the following perceptions: attractiveness (1 = not attractive at all, 9 = very

attractive); trustworthiness (1= not trustworthy at all, 9= very trustworthy); perceived health (1= not healthy at all, 9= very healthy); dominance (1 = not dominant at all, 9 = very dominant); aggressiveness (1= not aggressive at all, 9= very aggressive); and femininity/masculinity (1= very feminine/not masculine at all, 9= very masculine/ not feminine at all). For all trials, we randomized both the order of facial stimuli and the order of the questions about the perceived qualities. In addition, to standardize the response for attractiveness in men and women, we asked the men participants, *"How attractive does this face look for a woman?"* (For similar methods see [60]). Finally, raters completed a socio-demographic survey and were asked about their sexual orientation, on a 5-point Likert scale, from entirely same-gender to entirely opposite-gender orientation. As a result, 610 raters (437 women and 173 men) identified themselves as opposite-gender oriented/heterosexual, 42 (15 women and 27 men) identified as same-gender sexual orientation, 35 (28 women and 7 men) identified as bisexual, and 13 (9 women and 4 men) reported not having any sexual orientation. We controlled the sexual orientation information in the statistical analysis (see below). In addition, self-perceived skin color may have been influencing the rater's responses [23,61]. Hence, raters were asked to rate their own skin color using the PERLA color palette. The PERLA color palette is a scale of 11 skin tones (1 = lightest, 11 = darkest) that was made to provide an easy-to-use portrayal of the typical skin tones found in regions of Latin America [62]. Similar to previous studies on the Mexican population [24], we found that 77. 6% of the raters´ responses were concentrated in the middle levels of the scale (4-6), while the rest were at the extremes: 12.7 % in the lightest skin tones levels (1-3) and 9.7% in the darkest ones (7-10; see S1 Fig.). Finally, all raters signed an informed consent form before evaluating facial stimuli, and all procedures were approved by the appropriate National Autonomous University of

Mexico ethics committee (FPSI/CE/01/2016) and are in compliance with Mexican legislation and the Code of Ethical Principles for Medical Research Involving Human Subjects of the World Medical Association (Declaration of Helsinki).

Statistical analysis

To determine whether the experimental manipulation of facial skin color modified the perceived qualities, we fitted Poisson generalized linear mixed models (GLMM) with the log link function. We decided to fit Poisson GLMMs because, unlike other studies where the ratings for each facial stimulus are averaged, here all dependent variables come from discrete raw ratings. Moreover, during the data exploration we observed a positively skewed counts-type distribution with most of the observations at the lower values in some of the facial perceptions (i.e., attractiveness perception). We ran one global model per each perception evaluated. Global models included as the dependent variable any of the following facial perceptions: attractiveness, trustworthiness, perceived health, dominance, aggressiveness, or femininity/masculinity. Fixed predictors for all global models included the ethnic group of each evaluated face (i.e., European American or Me'phaa faces), the natural and transformed skin color versions (i.e., NAT, LSCt or DSCt), sex of raters, and the interaction between ethnic group and skin color versions. To account for the potential non-independence of data, we included a random intercept for rater ID, a random intercept for facial photograph ID (the individual code of each original face used), and a random slope for skin color transformation on facial photograph ID.

To evaluate the effect of rater´s sexual preferences, we ran models for responses from all participants and models for responses from heterosexual participants only. Next, each global model was then reduced to obtain a minimum adequate model using the Akaike Information Criterion (AIC). We selected the models with the lowest AIC values and a ΔAIC higher than two units from the second most supported model [63]. When the difference between the best-supported model and the subsequent model was $\Delta<2$, we followed the statistical principle of parsimony [64], and hence we selected the minimum adequate model as the one with the lowest AIC and the least number of parameters (Table 1). Finally, once we obtained minimum adequate models, model diagnostics were performed, and we checked residual distribution and linearity of residuals in each model. In order to check for any effect of other factors not included in the models [65,66], we plotted Pearson residuals of each selected model against the rater´s self-reported ethnicities and the PERLA scale responses for self-perceived skin color. Since the plots did not show any clear effect, we did not include either of the two factors as fixed predictors. All analyses were performed using the *lme4* [67] and *bbmle* [68] packages in R, version 4.0.2 [69].

Table 1. Information criteria for the best fit models.

	df	$AICc$	$\Delta AICc$	$w_i(AICc)$
(a) Perceived attractiveness				
**Group + Color + Rater´s sex + Group:Color**	**14**	**14331**	**0.000**	**0.999**
Group + Color + Rater´s sex	12	14346	15.053	0.001
Group + Color + Group:Color	13	14348	16.957	0.000
(b) Perceived trustworthiness				
**Group + Color**	**11**	**15167**	**0.000**	**0.506**
Group + Color + Rater´s sex	12	15169	1.778	0.208
Group + Color + Group: Color	13	15170	2.596	0.138
(c) Perceived health				
**Group + Color + Group:Color**	**13**	**15561**	**0.000**	**0.707**
Group + Color + Rater´s sex + Group:Color	14	15563	1.892	0.274
Group + Color	11	15570	9.186	0.007
(d) Perceived dominance				
**Group + Rater´s sex**	**10**	**15485**	**0.000**	**0.493**
Group + Color	11	15488	2.685	0.129
Group + Color + Group: Color	13	15489	3.137	0.103
(e) Perceived aggressiveness				
**Group + Color + Group:Color**	**13**	**16625**	**0.000**	**0.663**
Group + Color + Rater´s sex + Group:Color	14	16626	1.768	0.274
Group + Color	11	16630	5.479	0.043
(f) Perceived masculinity				
**Group + Color + Group:Color**	14	15486	0.000	0.482
Group + Color + Rater´s sex + Group:Color	**13**	**15486**	**0.035**	**0.474**
Group + Color	11	15492	6.367	0.020

Information criteria and fixed factors included for the three best fit models for: (a) Perceived attractiveness, (b) Perceived trustworthiness, (c) Perceived health, (d) Perceived dominance, (e) Perceived aggressiveness, and (f) Perceived masculinity.

Models are in descending order. ΔAIC is the change in AIC between each model and the best model. Akaike weights are conditional probabilities for each model being the best model. Models in bold are the selected ones.

Results

Perceived attractiveness

Perceived attractiveness differed significantly between the two groups when they had their natural skin color (z = 6.51, p < 0.01). The natural lighter facial (i.e. composed by natural European American faces) group were rated 1.70 times more attractive (M = 4.30, 95% CI [3.83 - 4.84]) that the natural darker facial group (i.e. composed by natural Me´phaa faces; M = 2.50, 95% CI [2.21 - 2.82], Table 2). Furthermore, there were effects when natural skin color was changed within each of the two groups. Lightening the natural darker group (i.e. Me’Phaa faces) made them more attractive (M = 3.75, 95% CI [3.42 - 4.11], z = 7.42, p < 0.01), and darkening the opposite group (i.e. European American faces) made them less attractive (M = 3.30, 95% CI [3.02 - 3.60], z = -6.25, p < 0.01). In addition, we observed an effect of skin color and texture homogenization due to experimental manipulation. Both groups became slightly more attractive when skin color and skin texture were homogenized (Me’Phaa faces: M = 2.94, 95% CI [2.68 - 3.21]; European American faces: M = 4.77, 95% CI [4.36 - 5.22]). However, this effect was significant for the darker faces group (i.e. Me’Phaa faces; z = -3.23, p < 0.01) but not for opposite group (i.e. the European American faces; z = -2.00, p = 0.34; Fig. 2). Finally, we observed a significant main effect of rater´s sex (z = 4.36, p<0.01); all the faces seemed more attractive to the male (M = 3.76, 95% CI [3.48 - 4.06]) than to the female raters (M = 3.27, CI 95% [3.06 - 3.49]). The direction of the effects and statistical significance were not affected when including the rater´s sexual preference (S1 Table.).

Table 2. Perception estimates with 95% confidence intervals.

	Me'Phaa			European American		
	**NAT**	**LSCt**	**DSCt**	**NAT**	**LSCt**	**DSCt**
	Est. [95%CI]	*Est. [95%CI]*	*Est. [95%CI]*	*Est. [95%CI]*	*Est. [95%CI]*	*Est. [95%CI]*
a) Perceived attractiveness	2.50 [2.21-2.82]	3.75 [3.42-4.11]	2.94 [2.68-3.21]	4.30 [3.83-4.84]	4.77 [4.36 5.22]	3.30 [3.02-3.60]
b) Perceived trustworthiness	4.21 [4.03-4.40]	4.48 [4.30-4.67]	4.33 [4.16-4.51]	4.75 [4.55-4.96]	5.06 [4.86-5.27]	4.89 [4.70-5.08]
c) Perceived health	4.28 [3.83-4.79]	5.51 [5.19-5.84]	5.21 [4.85-5.60]	5.10 [4.56-5.69]	5.96 [5.62-6.31]	5.05 [4.70-5.43]
d) Perceived dominance	5.23 [4.85-5.64]	□	□	4.31 [3.99-4.66]	□	□
e) Perceived aggressiveness	5.43 [5.17-5.69]	5.05 [4.85-5.26]	5.39 [5.17-5.62]	5.10 [4.85-5.35]	4.79 [4.59-4.99]	4.56 [4.37-4.77]
f) Perceived masculinity	6.18 [5.79-6.60]	5.15 [4.83-5.49]	5.97 [5.59-6.37]	5.14 [4.80-5.50]	4.83 [4.53-5.15]	4.93 [4.61-5.27]

Estimates (Est.) with 95% confidence intervals (CI) of the fixed predictors of the models for: (a) Perceived attractiveness, (b) Perceived trustworthiness, (c) Perceived health, (d) Perceived dominance, (e) Perceived aggressiveness, and (f) Perceived masculinity.
Note: (□*)* The Perceived dominance best fit model did not include the skin color transformation predictor, therefore the estimates shown represent the main effect of the natural skin color group only.

Fig. 2. Perceived attractiveness estimates for the Me'Phaa and European American faces within each color version. Estimates for the Natural versions (NAT), Light skin color transformed versions (LSCt), and Dark skin color transformed versions (DSCt) of the Me'Phaa (left) and European American (Right) faces. Error bars show 95% Confidence Intervals. Grey points and dashed lines indicate the random effect estimates for each Face ID within each color version.

Perceived trustworthiness

Natural lighter group were rated 1.13 times more trustworthy (*M* = 4.75, 95% CI [4.55 - 4.96], *z* = 5.19, *p* < 0.01) than natural darker group (*M* = 4.21, 95% CI [4.03 - 4.40], Table 2). In addition, lightening the natural darker group (i.e. Me'Phaa faces) increased their perceived trustworthiness (*M* = 4.48, 95% CI [4.30 - 4.67], *z* = 3.26, *p* < 0.01) whereas darkening the natural lighter group (i.e. European American faces) had no effect (*M* = 4.89, 95% CI [4.70 - 5.08], *z* = 1.44, *p* = 0.15). Skin color and texture homogenization did not increase scores for the natural darker faces (DSCt Me'Phaa faces M = 4.89, 95% CI [4.70 - 5.08], *z* = -1.43, *p* = 0.70), but did increase scores for the natural lighter faces (LSCt European American faces *M* = 5.06, 95% CI [4.86 - 5.27], *z* = -3.26, *p* = 0.01; Fig. 3). Finally, there was no effect of rater´s sex, as this predictor was not present in the minimum adequate model selected by AIC (Table 1). The direction of the effects and statistical significance were not affected when the rater´s sexual preference was included (S1 Table.).

Fig. 3. Perceived trustworthiness estimates for Me'Phaa and European American faces within each color version. Estimates for the Natural versions (NAT), Light skin color transformed versions (LSCt), and Dark skin color transformed versions (DSCt) of the Me'Phaa (left) and European American (Right) faces. Error bars show 95% Confidence Intervals. Grey points and dashed lines indicate the random effect estimates for each Face ID within each color version.

Perceived health

As with perceived attractiveness and trustworthiness, we found a significant difference in the perceived health scores between the natural darker and lighter faces (*z* = 2.20, *p* = 0.02). On average, natural lighter faces were perceived as being 1.19 times

healthier (*M* = 5.10, 95% CI [4.56 - 5.69]) than natural darker faces (*M* =4.28, 95% CI [3.83 - 4.79], Table 2). When facial skin color was changed within each ethnic group, the changes had different effects on perceptions of the two groups. Lightening the natural darker faces increased their perceived health (*M* = 5.51, 95% CI [5.19 - 5.84], z = 4.96, p < 0.01), whereas darkening the lighter faces had no effect (*M* = 5.05, 95% CI [4.70 - 5.43], z = 0.20, p = 0.99). Perceived health increased slightly for both groups when skin color and skin texture were homogenized (European Americans: *M* = 5.96 (95% CI [5.62 – 6.31], z = -3.12, p = 0.02; Me'phaa people: *M* = 5.21, 95% CI [4.85 - 5.60], z = 4.38, p < 0.01; Fig. 4). Finally, there was no effect of the rater´s sex, as this predictor was not present in the minimum adequate model selected by AIC (Table 1). The direction of the effects and statistical significance were not affected when the rater´s sexual preference was included (S1 Table.).

Fig. 4. Perceived health estimates for Me'Phaa and European American faces within each color version. Estimates for the Natural versions (NAT), Light skin color transformed versions (LSCt), and Dark skin color transformed versions (DSCt) of the Me'Phaa (left) and European American (Right) faces. Error bars show 95% Confidence Intervals. Grey points and dashed lines indicate the random effect estimates for each Face ID within each color version.

Perceived dominance

Unlike the previous qualities, perceived dominance was lower for natural lighter faces (*M* = 4.31, 95% CI [3.99 - 4.66], z = -3.63, p < 0.01) than for darker faces (*M* = 5.23, 95% CI [4.85 - 5.64], Table 2). Moreover, in contrast to perceived attractiveness, all faces received a lower dominance rating from male raters (*M* = 4.63, 95% CI [4.34 - 4.93], z = -2.24, p = 0.02) than from female raters (*M* = 4.88, 95% CI [4.61 - 5.16]). Finally, we were

unable to determine any effect from lightening, darkening, or homogenizing the faces, since these predictors were not present in the minimum adequate model selected by AIC (Table 1). Statistical significance of these results did not change in the model that included all raters (S1 Table.).

Perceived aggressiveness

Although perceived aggressiveness was lower for the lighter faces, the difference was not significant (z = -1.83, p = 0.06). Nevertheless, lightening the darker faces made them seem less aggressive (M = 5.05, 95% CI [4.85 - 5.26], z = -2.76, p < 0.01) but darkening the lighter faces also made them seem less aggressive (M = 4.56, 95% CI [4.37 - 4.77], z = 4.17, p < 0.01, Table 2). Finally, perceived aggressiveness did not change for either group when skin color and skin texture were homogenized (Me'Phaa faces M = 5.39, 95% CI [5.17 - 5.62]; z = -0.23, p = 0.82; European American faces M = 4.79, 95% CI [4.59 - 4.99]; z = 2.35, p = 0.17, Fig. 5.), and there was no effect of the rater´s sex, as this predictor was not present in the minimum adequate model selected by AIC (Table 1). Finally, the direction of the effects and statistical significance were not affected when the rater´s sexual preference was included (S1 Table.).

Fig. 5. Perceived aggressiveness estimates for Me'Phaa and European American faces within each color version. Estimates for the Natural versions (NAT), Light skin color transformed versions (LSCt), and Dark skin color transformed versions (DSCt) of the Me'Phaa (left) and European American (Right) faces. Error bars show 95% Confidence Intervals. Grey points and dashed lines indicate the random effect estimates for each Face ID within each color version.

Perceived masculinity

As with perceived dominance, perceived masculinity was lower for natural lighter faces (*M* = 5.14, 95% CI [4.80 - 5.50], *z* = -3.95, *p* < 0.01 than for natural darker faces (*M* = 6.18, 95% CI [5.79 - 6.60], Table 2). Moreover, lightening the darker faces made them seem less masculine (*M* = 5.97, 95% CI [5.59 - 6.37], *z* = -5.59, *p* < 0.01), but darkening the lighter faces had no effect (*M* = 4.93, 95% CI [4.61 - 5.27], *z* = 1.20, *p* = 0.83). In addition, perceived masculinity did not change for either group when skin color and skin texture were homogenized (DSCt Me'Phaa faces *M* = 5.97, 95% CI [5.59 - 6.37]; *z* = 1.05, *p* = 0.90; LSCt European American faces *M* = 4.83, 95% CI [4.53 - 5.15]; *z* = 1.82, *p* = 0.45, Fig. 6). Finally, there was no effect of the rater´s sex, as this predictor was not present in the minimum adequate model selected by AIC (Table 1). The direction of the effects and statistical significance was not affected when the rater´s sexual preference was included (S1 Table.).

Fig. 6. Perceived masculinity estimates for the Me'Phaa and European American faces within each color version. Estimates for the Natural versions (NAT), Light skin color transformed versions (LSCt), and Dark skin color transformed versions (DSCt) of the Me'Phaa (left) and European American (Right) faces. Error bars show 95% Confidence Intervals. Grey points and dashed lines indicate the random effect estimates for each Face ID within each color version.

Discussion

Our findings suggest that facial skin pigmentation influences social judgments in a Mexican population. Based on our results, we could distinguish two groupings of social perceptions that facial skin pigmentation affects in opposite directions. In comparison with

natural Me'Phaa faces, natural European American faces were perceived not only as more attractive, healthier, and more trustworthy but also as less dominant and less masculine. They were furthermore perceived as less aggressive, but not significantly. Moreover, the perceptions grouped in these two dimensions correlate more within than between dimensions (S2 Table.). The apparent grouping of social perceptions found here resembles the valence-dominance model of facial perception proposed by Oosterhof and Todorov [70] and recently discussed by Jones et al. [6]. The valence-dominance model suggests that humans make impressions of others based on their facial appearance in the dimensions of valence and dominance. The valence dimension is related to facial judgments such as trustworthiness and attractiveness; hence, higher rates of this dimension could promote positive social interactions, for example mate opportunities. The dominance dimension, in turn, is related to perceptions such as aggressiveness, dominance, and masculinity, which could inform about the physical or psychological capability to cause harm; hence, higher values of this dimension could negatively affect social interactions in a modern context [8]. Here, we found that natural light-skinned faces were rated more highly on all three aspects of social valence (attractiveness, healthiness, trustworthiness). In contrast, natural dark-skinned faces were rated more highly on two aspects of social dominance (dominance, masculinity).

In the context of our first hypothesis, these results support the idea that sociocultural learning favors the function of facial skin pigmentation as a mediator of social perceptions. Our Mexican participants showed a robust positive association of pro-social judgments (i.e., attractiveness, trustworthiness, and perceived health) with light skin pigmentation. During the Mexican colonial period, light skin pigmentation was one of the

most conspicuous phenotypic traits associated with a higher social status and access to different benefits, such as education and wealth [21]. Learning can reinforce a preference for a trait that may signal social benefits, even when the selected trait is not linked initially to any reproductive or survival benefit [19]. Therefore, a learned association over several generations between a physical trait—such as light skin—and deference might not only establish a social positive perception for the population that share this trait, but also give rise to racial stereotypes that negatively affect others coexisting populations, which reinforces the learning.. Although our methodological design did not explicitly provide information about the ethnicity of the stimuli subjects, it is possible that the stereotypes derived from this strong associative learning might have acted as the driving force influencing the social scores. In Mexico, darker skin color suggests that individuals are more indigenous, and consequently considered as more socially unfavorable. Furthermore, several studies have shown cultural preferences for light skin pigmentation and other European phenotypical traits and within-group discrimination based on skin color, a social phenomenon known as colorism [71]. In addition, in Mexico and other Latin American countries, lightness of skin correlates with years of schooling, hourly earnings, occupational status, poverty, and social mobility [23–25]. Therefore, the association of pro-social attributes with light skin pigmentation we observed may be the result of a preference that emerged as an adaptative strategy to deal with social pressures during the colonial period, which nowadays is reinforced by social learning and cultural mechanisms such as colorism, or the influence of Western societies on social media.

We also found that the dark-skinned faces were rated as more dominant and masculine. However, in contrast to our second prediction, they were not rated as more

attractive or healthy. These results could indicate that skin pigmentation, as a trait mediated by androgens, could inform the viewer about the physical or psychological capability to cause harm by the bearer of the signal [72,73]; therefore, this signaling function might be relevant in sexual selection through male-male competition instead of female choice[74]. Similar results have been found in previous studies where androgen-dependent sexually dimorphic traits, such as facial shape [75], voice pitch [76,77], or height [78], more strongly affect the perception of dominance, aggressiveness, or masculinity rather than attractiveness.

The human face embodies a variety of signals and cues [79], and even though humans process faces in an integrative manner, this process follows a hierarchy of relevance in which different facial features are more important than others, depending on the perceptions evaluated [80,81]. In this way we found that some social perceptions were more affected by skin color than others. For example, lightening the Me'Phaa faces made them seem more attractive, healthier, and more trustworthy. Conversely, darkening the European American faces made them seem less attractive. Other perceived qualities, however, did not change systematically when facial skin color was changed. Perceived dominance was not affected by manipulations of facial skin color within either group. Lightening the Me'Phaa faces decreased their perceived masculinity and aggressiveness, but darkening the European American faces did not increase their perceived masculinity and aggressiveness. These results are consistent with studies on the role of skin color in certain perceptions, such as attractiveness [59,82], which show that skin color might contribute more than facial shape. Moreover, recent studies have shown that trustworthiness judgments rely on emotional expressions [8] or facial resemblance [83], and

that perceptions of dominance, masculinity, and aggressiveness seem to rely more strongly on facial shape [84–88]. Yet another possibility is that skin color interacts with facial shape in some of these perceptions, and it becomes relevant, only when facial shape strongly elicits the perception. This may explain why lightening of skin color reduced the perceived masculinity and aggressiveness for the Me´Phaa faces, which were initially rated as more masculine and dominant, but darkening had no effect on the European American faces.

Another possibility is that there were systematic differences in the way that the experimental manipulation of skin color affected the European American and Me´Phaa faces. In the statistical analysis we fitted random slopes for skin color transformation on each facial photograph ID to reduce the standard error caused by these differences; however, it is important to note that any manipulation presents a risk of such artifacts. Moreover, we not only lightened and darkened facial color but also homogenized it by removing such texture-related aspects as spots, freckles, or wrinkles [59,89,90]. Comparing the two color transformed versions of each group, that is the ones with texture and color homogenized, we observed, in general, the main effects of skin color previously reported (see Fig. 2-6); however, our results suggest that skin texture does have an effect, particularly on perceived health. Therefore, skin texture might interact with skin color in perceptions of light and dark skin, and such interactions could be an exciting avenue for further studies. In addition, unlike the authors of previous studies, we manipulated overall skin color instead of gradually changing each of its aspects (e.g., luminosity, hue) [33,35,46,53,60,82,91–94]. In our study, we aimed to test the differences in skin colors typically related to two different ethnic groups by using a broader manipulation of skin

color. Nonetheless, future studies could examine how each aspect of skin color contributes to the way a population is perceived.

Finally, previous studies have shown that individuals are better at judging faces of their own ethnicity [35,95]. In this study our Mexican participants were asked to rate facial stimuli of a more closely related ethnic group (Me´Phaa faces), and stimuli from a more distant ethnic group (European American faces). Although 83% of our raters reported living in Mexico City and the surrounded area, and hence were likely exposed to European facial features due to immigration of people of European ethnicity and access to the internet and social media [96,97], these differences in the effects of the skin color manipulation between European American and Me´Phaa faces might be due to different degrees of familiarity with the facial features of the two ethnic groups.

In summary, we show that the Mexican population uses facial color to make certain social judgments. We found that a dark-skinned face is perceived as more dominant and more masculine. Those perceptions may be due to a process of coevolution between the human mind and the sex difference in skin color. One may therefore infer that male skin became darker through male-male competition rather than through female choice. We also found that a light-skinned face is perceived as more attractive, more trustworthy, and healthier. Those perceptions may be due to the association between skin color and social status that prevailed in Mexico during more than three centuries of colonial rule. Today, they are maintained by colorism. We are nonetheless speculating, and more data will be needed. Similar results could be obtained from other Latin American societies, which share a common colonial history and social structure.

This study may complement the social psychology and sociological literature on skin color-based discrimination. In Latin American countries, the distinction between indigenous and non-indigenous populations is a fluid continuum with complex relationships with skin color and socioeconomic status. In Mexico, cultural practices, sense of belonging, and regional language use are the characteristics that best describe this classification [98,99]; nonetheless, skin color still has effects on the way the Mexican population perceive each other and, thus, make this distinction, mainly by promoting racial attitudes, and stereotypes. For example, Flores and Telles [25] found that in Mexicans with the same skin color, those with high income are twice as likely to be perceived and described as white than those with low income. Here we found that our raters were more willing to rate light skinned faces higher in all three perceptions that are closely related to positive social interactions. Remarkably, even when our results come from a study designed to address different questions, they support the idea of a cultural preference for light skin color based on previous findings of studies on racial attitudes and social stratification. Therefore, the efforts from different disciplines to develop a better understanding of the complex effects that skin color has on several aspects of the Mexican psychology and society need to continue.

Acknowledgements

The authors are grateful to Juan Sebastían Lucero Carrasquilla for his help and assistance during the facial photograph acquisition of the Me’Phaa men sample.

References

1. Little AC, Jones BC, Debruine LM. Facial attractiveness: Evolutionary based research. Philosophical Transactions of the Royal Society B: Biological Sciences. 2011;366: 1638–1659. doi:10.1098/rstb.2010.0404

2. Puts DA, Jones BC, Debruine LM. Sexual selection on human faces and voices. J Sex Res. 2012;49: 227–243. doi:10.1080/00224499.2012.658924

3. Gonzalez-Santoyo I, Wheatley JR, Welling LLM, Cárdenas RA, Jimenez-Trejo F, Dawood K, et al. The face of female dominance: Women with dominant faces have lower cortisol. Horm Behav. 2015;71: 16–21. doi:10.1016/j.yhbeh.2015.03.006

4. Puts DA, Bailey DH, Cárdenas RA, Burriss RP, Welling LLM, Wheatley JR, et al. Women's attractiveness changes with estradiol and progesterone across the ovulatory cycle. Horm Behav. 2013;63: 13–19. doi:10.1016/j.yhbeh.2012.11.007

5. Ritchie KL, Palermo R, Rhodes G. Forming impressions of facial attractiveness is mandatory. Sci Rep. 2017;7: 1–8. doi:10.1038/s41598-017-00526-9

6. Jones BC, DeBruine LM, Flake JK, Liuzza MT, Antfolk J, Arinze NC, et al. To which world regions does the valence–dominance model of social perception apply? Nat Hum Behav. 2021;5: 159–169. doi:10.1038/s41562-020-01007-2

7. Olivola CY, Funk F, Todorov A. Social attributions from faces bias human choices. Trends Cogn Sci. 2014;18: 566–570. doi:10.1016/j.tics.2014.09.007

8. Todorov A, Olivola CY, Dotsch R, Mende-Siedlecki P. Social attributions from faces: Determinants, consequences, accuracy, and functional significance. Annu Rev Psychol. 2015;66: 519–545. doi:10.1146/annurev-psych-113011-143831

9. Barsh GS. What controls variation in human skin color? PLoS Biol. 2003;1: 19–23. doi:10.1371/journal.pbio.0000027

10. Samson N, Fink B, Matts PJ. Visible skin condition and perception of human facial appearance. Int J Cosmet Sci. 2010;32: 167–184. doi:10.1111/j.1468-2494.2009.00535.x

11. Edwards EA, Duntley SQ. THE PIGMENTS AND COLOR OF LIVING HUMAN SKIN. American Journal of Anathmy. 1939;65.

12. Darwin C. The Descent of Man, and Selection in Relation to Sex. J. Murray. London; 1871.

13. Jablonski NG, Chaplin G. The evolution of human skin coloration. J Hum Evol. 2000;39: 57–106. doi:10.1006/jhev.2000.0403

14. Adhikari K, Mendoza-Revilla J, Sohail A, Fuentes-Guajardo M, Lampert J, Chacón-Duque JC, et al. A GWAS in Latin Americans highlights the convergent evolution of lighter skin pigmentation in Eurasia. Nat Commun. 2019;10. doi:10.1038/s41467-018-08147-0

15. Deng L, Xu S. Adaptation of human skin color in various populations. Hereditas. 2018;155: 1. doi:10.1186/s41065-017-0036-2

16. Jablonski NG, Chaplin G. Human skin pigmentation as an adaptation to UV radiation. Proc Natl Acad Sci U S A. 2010;107: 8962–8968. doi:10.1073/pnas.0914628107

17. Feldman MW, Cavalli-Sforza LL. Cultural and biological evolutionary processes: Gene-culture disequilibrium. Isotopenpraxis. 1984;20: 1604–1607. doi:10.1073/pnas.81.5.1604

18. Verzijden MN, ten Cate C, Servedio MR, Kozak GM, Boughman JW, Svensson EI. The impact of learning on sexual selection and speciation. Trends Ecol Evol. 2012;27: 511–519. doi:10.1016/j.tree.2012.05.007

19. Little AC, Jones BC, Debruine LM, Caldwell CA. Social learning and human mate preferences: A potential mechanism for generating and maintaining between-population diversity in attraction. Philosophical Transactions of the Royal Society B: Biological Sciences. 2011;366: 366–375. doi:10.1098/rstb.2010.0192

20. Rendell L, Fogarty L, Hoppitt WJE, Morgan TJH, Webster MM, Laland KN. Cognitive culture: Theoretical and empirical insights into social learning strategies. Trends Cogn Sci. 2011;15: 68–76. doi:10.1016/j.tics.2010.12.002

21. Dixon AR, Telles EE. Skin color and colorism: Global research, concepts, and measurement. Annu Rev Sociol. 2017;43: 405–424. doi:10.1146/annurev-soc-060116-053315

22. Villarreal A. Stratification by skin color in contemporary Mexico. Am Sociol Rev. 2010;75: 652–678. doi:10.1177/0003122410378232

23. Telles E, Flores RD, Urrea-Giraldo F. Pigmentocracies: Educational inequality, skin color and census ethnoracial identification in eight Latin American countries. Res Soc Stratif Mobil. 2015;40: 39–58. doi:10.1016/j.rssm.2015.02.002

24. Campos-Vazquez RM, Medina-Cortina EM. Skin Color and Social Mobility: Evidence From Mexico. Demography. 2019;56: 321–343. doi:10.1007/s13524-018-0734-z

25. Flores R, Telles E. Social Stratification in Mexico: Disentangling Color, Ethnicity, and Class. Am Sociol Rev. 2012;77: 486–494. doi:10.1177/0003122412444720

26. van den Berghe PL, Frost P. Skin color preference, sexual dimorphism and sexual selection: A case of gene culture co-evolution? Ethn Racial Stud. 1986;9: 87–113. doi:10.1080/01419870.1986.9993516

27. Madrigal L, Kelly W. Human skin-color sexual dimorphism: A test of the sexual selection hypothesis. reply to frost (2007). Am J Phys Anthropol. 2007;133: 780–781. doi:10.1002/ajpa.20582

28. Aoki K. Sexual selection as a cause of human skin colour variation: Darwin's hypothesis revisited. Ann Hum Biol. 2002;29: 589–608. doi:10.1080/03014460210000019144

29. Giacomoni PU, Mammone T, Teri M. Gender-linked differences in human skin. J Dermatol Sci. 2009;55: 144–149. doi:10.1016/j.jdermsci.2009.06.001

30. Edwards EA, Hamilton JB, Duntley SQ, Hubert G. CUTANEOUS VASCULAR AND PIGMENTARY CHANGES IN CASTRATE AND EUNUCHOID MEN. Endocrinology. 1941;28: 119–128.

31. Manning JT, Bundred PE, Mather FM. Second to fourth digit ratio, sexual selection, and skin colour. Evolution and Human Behavior. 2004;25: 38–50. doi:10.1016/S1090-5138(03)00082-5

32. Harvey RG. Ecological factors in skin color variation among Papua New Guineans. Am J Phys Anthropol. 1985;66: 407–416. doi:10.1002/ajpa.1330660409

33. Carrito M de L, Santos IMB dos, Lefevre CE, Whitehead RD, Silva CF da, Perrett DI. The role of sexually dimorphic skin colour and shape in attractiveness of male faces. Evolution and Human Behavior. 2016;37: 125–133. doi:10.1016/j.evolhumbehav.2015.09.006

34. Stephen ID, Coetzee V, Perrett DI. Carotenoid and melanin pigment coloration affect perceived human health. Evolution and Human Behavior. 2011;32: 216–227. doi:10.1016/j.evolhumbehav.2010.09.003

35. Coetzee V, Greeff JM, Stephen ID, Perrett DI. Cross-cultural agreement in facial attractiveness preferences: The role of ethnicity and gender. PLoS One. 2014;9. doi:10.1371/journal.pone.0099629

36. Frost P. Different Phases of the Menstrual Cycle : Preliminary assesment of evidence for a hormonal relationship. Percept Mot Skills. 1994;79: 507–514.

37. Folstad I, Karter AJ. Parasites, bright males, and the immunocompetence handicap. American Naturalist. 1992;139: 603–622. doi:10.1086/285346

38. Rantala MJ, Moore FR, Skrinda I, Krama T, Kivleniece I, Kecko S, et al. Evidence for the stress-linked immunocompetence handicap hypothesis in humans. Nat Commun. 2012;3. doi:10.1038/ncomms1696

39. Marcinkowska UM, Rantala MJ, Lee AJ, Kozlov M V., Aavik T, Cai H, et al. Women's preferences for men's facial masculinity are strongest under favorable ecological conditions. Sci Rep. 2019;9: 1–10. doi:10.1038/s41598-019-39350-8

40. Hagiwara N, Kashy DA, Cesario J. The independent effects of skin tone and facial features on Whites' affective reactions to Blacks. J Exp Soc Psychol. 2012;48: 892–898. doi:10.1016/j.jesp.2012.02.001

41. Stepanova E v., Strube MJ. What's in a face? the role of skin tone, facial physiognomy, and color presentation mode of facial primes in affective priming effects. Journal of Social Psychology. 2012;152: 212–227. doi:10.1080/00224545.2011.597797

42. Dunham Y, Stepanova E v., Dotsch R, Todorov A. The development of race-based perceptual categorization skin color dominates early category judgments. Dev Sci. 2015;18: 469–483. doi:10.1111/desc.12228

43. Jack RE, Crivelli C, Wheatley T. Data-Driven Methods to Diversify Knowledge of Human Psychology. Trends Cogn Sci. 2018;22: 1–5. doi:10.1016/j.tics.2017.10.002

44. Torres F, Salgado M, Mackenna B, Núñez J. Who differentiates by skin color? Status attributions and skin pigmentation in Chile. Front Psychol. 2019;10: 1–12. doi:10.3389/fpsyg.2019.01516

45. Oosterhof NN, Todorov A. The functional basis of face evaluation. Proc Natl Acad Sci U S A. 2008;105: 11087–11092. doi:10.1073/pnas.0805664105

46. Stephen ID, Law Smith MJ, Stirrat MR, Perrett DI. Facial skin coloration affects perceived health of human faces. Int J Primatol. 2009;30: 845–857. doi:10.1007/s10764-009-9380-z

47. Stephen ID, Oldham FH, Perrett DI, Barton RA. Redness enhances perceived aggression, dominance and attractiveness in men's faces. Evolutionary Psychology. 2012;10: 562–572. doi:10.1177/147470491201000312

48. Borras-Guevara ML, Batres C, Perrett DI. Fear of Violence among Colombian Women Is Associated with Reduced Preferences for High-BMI Men. Human Nature. 2019;30: 341–369. doi:10.1007/s12110-019-09350-8

49. Carré JM, Archer J. Testosterone and human behavior: the role of individual and contextual variables. Curr Opin Psychol. 2018;19: 149–153. doi:10.1016/j.copsyc.2017.03.021

50. Geniole SN, Bird BM, McVittie JS, Purcell RB, Archer J, Carré JM. Is testosterone linked to human aggression? A meta-analytic examination of the relationship between baseline, dynamic, and manipulated testosterone on human aggression. Horm Behav. 2020;123: 104644. doi:10.1016/j.yhbeh.2019.104644

51. Puts DA, Welling LLM, Burriss RP, Dawood K. Men's masculinity and attractiveness predict their female partners' reported orgasm frequency and timing. Evolution and Human Behavior. 2012;33: 1–9. doi:10.1016/j.evolhumbehav.2011.03.003

52. Foo YZ, Rhodes G, Simmons LW. The carotenoid beta-carotene enhances facial color, attractiveness and perceived health, but not actual health, in humans. Behavioral Ecology. 2017;28: 570–578. doi:10.1093/beheco/arw188

53. Kleisner K, Kočnar T, Tureček P, Stella D, Akoko RM, Třebický V, et al. African and European perception of African female attractiveness. Evolution and Human Behavior. 2017;38: 744–755. doi:10.1016/j.evolhumbehav.2017.07.002

54. Alaluf S, Atkins D, Barrett K, Blount M, Carter N, Heath A. The impact of epidermal melanin on objective measurements of human skin colour. Pigment Cell Res. 2002;15: 119–126. doi:10.1034/j.1600-0749.2002.1o072.x

55. Visscher MO. Skin Color and Pigmentation in Ethnic Skin. Facial Plast Surg Clin North Am. 2017;25: 119–125. doi:10.1016/j.fsc.2016.08.011

56. del Bino S, Bernerd F. Variations in skin colour and the biological consequences of ultraviolet radiation exposure. British Journal of Dermatology. 2013;169: 33–40. doi:10.1111/bjd.12529

57. Tiddeman B, Perrett D. Transformation of dynamic facial image sequences using static 2D prototypes. Visual Computer. 2002;18: 218–225. doi:10.1007/s003710100142

58. Coetzee V, Faerber SJ, Greeff JM, Lefevre CE, Re DE, Perrett DI. African Perceptions of Female Attractiveness. PLoS One. 2012;7: 3–8. doi:10.1371/journal.pone.0048116

59. Fink B, Grammer K, Matts PJ. Visible skin color distribution plays a role in the perception of age, attractiveness, and health in female faces. Evolution and Human Behavior. 2006;27: 433–442. doi:10.1016/j.evolhumbehav.2006.08.007

60. Lefevre CE, Perrett DI. Fruit over sunbed: Carotenoid skin colouration is found more attractive than melanin colouration. Quarterly Journal of Experimental Psychology. 2015;68: 284–293. doi:10.1080/17470218.2014.944194

61. Paschetta C, de Azevedo S, Ramallo V, Cintas C, Pérez O, Navarro P, et al. The impact of socioeconomic and phenotypic traits on self-perception of ethnicity in Latin America. Sci Rep. 2021;11: 1–11. doi:10.1038/s41598-021-92061-x

62. Telles EE. Pigmentocracies: Ethnicity, Race and Color in Latin America. North Carolina: UNC Press Book; 2014.

63. Bolker BM, Brooks ME, Clark CJ, Geange SW, Poulsen JR, Stevens MHH, et al. Generalized linear mixed models: a practical guide for ecology and evolution. Trends Ecol Evol. 2009;24: 127–135. doi:10.1016/j.tree.2008.10.008

64. Burnham KP, Anderson DR. Multimodel inference: Understanding AIC and BIC in model selection. Sociol Methods Res. 2004;33: 261–304. doi:10.1177/0049124104268644

65. Zuur AF, Ieno EN, Walker NJ, Saveliev AA, Smith GM. Mixed Effects Models and Extensions in Ecology with R. NewYork: Springer; 2009.

66. Zuur AF, Ieno EN. A protocol for conducting and presenting results of regression-type analyses. Methods Ecol Evol. 2016;7: 636–645. doi:10.1111/2041-210X.12577

67. Bates D, Mächler M, Bolker BM, Walker SC. Fitting linear mixed-effects models using lme4. J Stat Softw. 2015;67. doi:10.18637/jss.v067.i01

68. Bolker M ben. Package ‘ bbmle .’ 2021.

69. R Core Team. R: A language and environment for statistical computing. Vienna, Austria: R Foundation for Statistical Computing.; 2020.

70. Oosterhof NN, Todorov A. The functional basis of face evaluation. Proc Natl Acad Sci U S A. 2008;105: 11087–11092. doi:10.1073/pnas.0805664105

71. Dixon AR. Colorism and classism confounded: Perceptions of discrimination in Latin America. Soc Sci Res. 2019;79: 32–55. doi:10.1016/j.ssresearch.2018.12.019

72. Kleisner K, Tureček P, Roberts SC, Havlíček J, Valentova JV, Akoko RM, et al. How and why patterns of sexual dimorphism in human faces vary across the world. Sci Rep. 2021;11: 1–14. doi:10.1038/s41598-021-85402-3

73. Puts DA. Human sexual selection. Curr Opin Psychol. 2016;7: 28–32. doi:10.1016/j.copsyc.2015.07.011

74. Puts DA. Beauty and the beast: Mechanisms of sexual selection in humans. Evolution and Human Behavior. 2010;31: 157–175. doi:10.1016/j.evolhumbehav.2010.02.005

75. Windhager S, Schaefer K, Fink B. Geometric morphometrics of male facial shape in relation to physical strength and perceived attractiveness, dominance, and masculinity. American Journal of Human Biology. 2011;23: 805–814. doi:10.1002/ajhb.21219

76. Puts DA, Apicella CL, Cárdenas RA. Masculine voices signal men’s threat potential in forager and industrial societies. Proceedings of the Royal Society B: Biological Sciences. 2012;279: 601–609. doi:10.1098/rspb.2011.0829

77. Rosenfield KA, Sorokowska A, Sorokowski P, Puts DA. Sexual selection for low male voice pitch among Amazonian forager-horticulturists. Evolution and Human Behavior. 2020;41: 3–11. doi:10.1016/j.evolhumbehav.2019.07.002

78. Batres C, Re DE, Perrett DI. Influence of Perceived Height, Masculinity, and Age on Each Other and on Perceptions of Dominance in Male Faces. Perception. 2015;44: 1293–1309. doi:10.1177/0301006615596898

79. Waller BM, Kavanagh E, Micheletta J, Clark PR, Whitehouse J. The face is central to primate multicomponent signals. Int J Primatol. 2022. doi:10.1007/s10764-021-00260-0

80. Karczmarek P, Pedrycz W, Kiersztyn A, Rutka P. A study in facial features saliency in face recognition: an analytic hierarchy process approach. Soft comput. 2017;21: 7503–7517. doi:10.1007/s00500-016-2305-9

81. Rotshtein P, Geng JJ, Driver J, Dolan RJ. Role of features and second-order spatial relations in face discrimination, face recognition, and individual face skills: Behavioral and functional magnetic resonance imaging data. J Cogn Neurosci. 2007;19: 1435–1452. doi:10.1162/jocn.2007.19.9.1435

82. Scott IML, Pound N, Stephen ID, Clark AP, Penton-Voak IS. Does masculinity matter? The contribution of masculine face shape to male attractiveness in humans. PLoS One. 2010;5. doi:10.1371/journal.pone.0013585

83. DeBruine LM. Trustworthy but not lust-worthy: Context-specific effects of facial resemblance. Proceedings of the Royal Society B: Biological Sciences. 2005;272: 919–922. doi:10.1098/rspb.2004.3003

84. Mogilski JK, Welling LLM. The relative contribution of jawbone and cheekbone prominence, eyebrow thickness, eye size, and face length to evaluations of facial masculinity and attractiveness: A conjoint data-driven approach. Front Psychol. 2018;9: 1–11. doi:10.3389/fpsyg.2018.02428

85. Torrance JS, Wincenciak J, Hahn AC, DeBruine LM, Jones BC. The relative contributions of facial shape and surface information to perceptions of attractiveness and dominance. PLoS One. 2014;9. doi:10.1371/journal.pone.0104415

86. Třebický V, Saribay SA, Kleisner K, Akoko RM, Kočnar T, Valentova JV, et al. Cross-Cultural Evidence for Apparent Racial Outgroup Advantage: Congruence between Perceived Facial Aggressiveness and Fighting Success. Sci Rep. 2018;8: 1–7. doi:10.1038/s41598-018-27751-0

87. Mitteroecker P, Windhager S, Müller GB, Schaefer K. The morphometrics of “masculinity” in human faces. PLoS One. 2015;10: 1–13. doi:10.1371/journal.pone.0118374

88. Windhager S, Schaefer K, Fink B. Geometric morphometrics of male facial shape in relation to physical strength and perceived attractiveness, dominance, and masculinity. American Journal of Human Biology. 2011;23: 805–814. doi:10.1002/ajhb.21219

89. Russell R. Sex, beauty, and the relative luminance of facial features. Perception. 2003;32: 1093–1107. doi:10.1068/p5101

90. Oh DW, Dotsch R, Todorov A. Contributions of shape and reflectance information to social judgments from faces. Vision Res. 2019;165: 131–142. doi:10.1016/j.visres.2019.10.010

91. Han C, Wang H, Hahn AC, Fisher CI, Kandrik M, Fasolt V, et al. Cultural differences in preferences for facial coloration. Evolution and Human Behavior. 2018;39: 154–159. doi:10.1016/j.evolhumbehav.2017.11.005

92. Stephen ID, Scott IML, Coetzee V, Pound N, Perrett DI, Penton-Voak IS. Cross-cultural effects of color, but not morphological masculinity, on perceived attractiveness of men's faces. Evolution and Human Behavior. 2012;33: 260–267. doi:10.1016/j.evolhumbehav.2011.10.003

93. Whitehead RD, Re D, Xiao D, Ozakinci G, Perrett DI. You are what you eat: Within-subject increases in fruit and vegetable consumption confer beneficial skin-color changes. PLoS One. 2012;7: 1–9. doi:10.1371/journal.pone.0032988

94. Whitehead RD, Ozakinci gözde, Perrett DI. Attractive skin coloration: Harnessing sexual selection to improve diet and health. Evolutionary Psychology. 2012;10: 842–854. doi:10.1177/147470491201000507

95. Rhodes G, Lee K, Palermo R, Weiss M, Yoshikawa S, Clissa P, et al. Attractiveness of own-race, other-race, and mixed-race faces. Perception. 2005;34: 319–340. doi:10.1068/p5191

96. INEGI. ENCUESTA NACIONAL SOBRE DISPONIBILIDAD Y USO DE TECNOLOGÍAS DE LA INFORMACIÓN EN LOS HOGARES (ENDUTIH) 2021. Encuesta Nacional Sobre Disponibilidad Y Uso De Tecnologías De La Información En Los Hogares (Endutih) 20211. 2022; 1–19.

97. INEGI. Los extranjeros en México. XII Censo General de Población y Vivienda, 2000. 2014; 67.

98. Flores RD, Vignau Loría M, Casas RM, Loria V, Martínez-Casas R. Transitory vs. durable boundary crossing: What explains the indigenous population boom in Mexico? 2021.

99. Villarreal A. Ethnic identification and its consequences for measuring inequality in Mexico. Am Sociol Rev. 2014;79: 775–806. doi:10.1177/0003122414541960

Supporting information

S1 Fig. Participant's self-perceived skin color ratings. PERLA scale distribution of the 700 participant's own skin color ratings. The color levels go from 1 to 10, being 1 the lightest and 10 the darkest. 11 (1.6 %) picked skin tone level 1, 36 (5.1%) skin tone level 2, 42 (6%) skin tone level 3, 240 (34.3%) skin tone level 4, 201 (28.8%) skin tone level 5, 102 (14.6%) skin tone level 6, 45 (6.4%) skin tone level 7, 14 (2%) skin tone level 8, 7 (1%) skin tone level 9, and 1 (0.1%) skin tone level 10.

S1 Table. Perception estimates with 95% confidence intervals with all participants included. Estimates (Est.) with 95% confidence intervals (CI) of the fixed predictors of the models for: (a) Perceived attractiveness, (b) Perceived trustworthiness, (c) Perceived health, (d) Perceived dominance, (e) Perceived aggressiveness, and (f) Perceived masculinity. *Note:* (□*)* The dominance best fitted model did not include the skin color transformation predictor, therefore the estimates shown represent the main effect of the natural skin color group only.

S2 Table. Pearson's correlation analysis between the scores of all perceptions rates of the natural versions only. Pearson's correlation between the natural versions scores of attractiveness, trustworthiness, perceived health, dominance, masculinity, and aggressiveness. *p<0.05, **p<0.01, ***p<0.001.

Reviewer 1:

Comment:

This revision is an improvement but there are still two problems. The first is correctable and I am not sure about the second:

1. The study focuses on the evolutionary role of skin color in Mexican society. However, they hardly engage with the metaphorical "elephant in the room:" current societal skin color inequality and discrimination. While the literature review acknowledges societal inequalities based on skin color, mostly in the social psychology and sociological literature, the authors do not discuss how it fits with their own findings. Except for mentioning this literature (not until pages 5-6), there is no serious engagement. How do their finding co-exist with those of racial attitudes, stereotyping and racial inequality? The abstract, for example, does not even mention that. I expect that readers would want to know how these evolutionary findings may complement (or not) the social science findings. For example, I see complementarity in how the findings are consistent with stereotypes of indigenous people.

Response:

We appreciate the previous comments from the reviewer; they have been instrumental in enhancing the manuscript's writing. Furthermore, we consider that these two recent observations provide valuable information to enhance the emphasis we have aimed about inequality and social discrimination based on skin stereotypes, and on how our evolutionary framework and results complement (or not) the social science findings.

We strongly agree that our results are consistent with previous studies in the social sciences regarding stereotypes on indigenous people. Additionally, we do not deny the current existence of societal skin color inequality and discrimination in the studied Mexican population. In fact, we propose that these behaviors are the result of these historical social pressures and cultural transmission.

Finally, the deliberate choice for alluding these concepts up until page 4 was because the initial segment of our introduction aimed to elucidate the physiological underpinnings of skin color evolution within the human species. It's important to note that this emphasis was not indicative of a lack of recognition or engagement with the prevailing issues of contemporary societal disparities related to skin color. To be more precise with these assertions, we have expanded the information in introduction (lines 101-120) and discussion (lines 569-585). In addition, we have modified the abstract to more clearly incorporate these aspects mentioned above (Lines 60-67).

Comment:

2. My other comment is on the methods, which have implications for the substantive interpretations. Reviewers 2 and 3 also seem to have alluded to this. The authors claim that there is no information about the ethnicity of the stimuli subjects but that in one treatment, only skin color and not facial features, was changed. But skin color is the information! Darker skin color in Mexico suggests that persons are more indigenous. Note that skin color, like indigeneity, is fluid and on a continuum, especially between mestizos and

indigenous people (see for example Villarreal 2013 in the American Sociological Review and Flores et al 2023 in the American Journal of Sociology).

Response:
Thank you for this insightful observation. Indeed, this aspect holds pivotal significance for our proposition. Precisely, the aim of this study was to ascertain whether skin color serves as a conduit of information influencing social perceptions. In our study we supported this premise experimentally by altering skin color according to the facial color patterns of these two racial groups. Consequently, we propose that skin color can be employed to associate ethnicity with its learned social stereotypes, and this interplay could potentially underpin social perceptions. These two elements, coloration and stereotype, would not be intertwined without a long and robust process of cultural learning and transmission that facilitates the stereotyping of a racial group based on a distinguishing phenotypic trait (i.e., skin color). In fact, this forms the bedrock of our proposition concerning cultural transmission and social learning in the evolution of human culture. We elucidate and expand upon this information in lines 484-492. Aligned with this notion, what holds significance here is that the color-ethnicity-perception nexus has evolved as a consequence of learned cultural pressures that have delineated what is socially favorable (or unfavorable) based on an evaluation of skin color—a physical attribute rooted in a distinct evolutionary origin (i.e., environmental pressures due to UV radiation). In contemporary times, this attribute is also harnessed for categorization, stereotyping, and modulation of social perceptions. This historical social pressure revolving around skin color could furthermore give rise to components of discrimination and racism within modern Mexican society. These intricate connections have been deeply interwoven throughout the history of Latin America and disentangling them proves challenging when attempting to elucidate their compounded effects. We clarify and expand upon this information in lines 569-585. Lastly, while acknowledging that disentangling racial stereotypes from skin color presents a formidable challenge, we believe that our study could serve as useful reference for future endeavors in social sciences that delve into this issue with the necessary depth. We do encourage this vision in the paragraph previously reported (lines 569-585). We also included a paragraph to explain that skin color and ethnicity form a continuous gradient, rather than a categorical distinction as implemented in the current study. Therefore, future research is needed to ascertain the extent to which these social perceptions are influenced by the fluid continuum of these two dimensions. Please see lines 536-548.

Reviewer #2:

Comment:
The manuscript could be better written. I have attached a corrected version of the PDF (see pages 16 to 51). Otherwise, I have no issues with the revised manuscript.

Response:
We greatly appreciate the reviewer's effort and time in providing us with precise suggestions, which will significantly improve our manuscript. Therefore, we have rewritten all sentences. Once again, thank you very much.

Reviewer #4:

Comment:

1. It's a bit surprising that Lightness (L*) is the only parameter considered in this study whereas the aim is to investigate the influence of skin pigmentation on social judgments.

Obviously, the reason given in line 222 (lightness has a negative correlation with melanin) is not rational. In fact, melanin pigmentation is not only negatively affects the skin's lightness, but possibly even more strongly and positively linked to skin yellowness. And it also influences skin redness. See for example the below reference. There are also other skin pigmentations. If it's only lightness (L*) is investigated, the title and the scope should be restricted to the effect of 'skin lightness' not 'skin pigmentation', on facial impressions. Otherwise, it's not fair to study the influence of skin colour on facial impressions only based on L* values.

"Fruit over sunbed: Carotenoid skin colouration is found more attractive than melanin colouration," Q. J. Exp. Psychol., vol. 68, no. 2, pp. 284–293, Feb. 2015, doi: 10.1080/17470218.2014.944194.

Kikuchi, Kumiko, et al. "Image analysis of skin colour heterogeneity focusing on skin chromophores and the age- related changes in facial skin." Skin Research and Technology 21.2 (2015): 175-183.

Response:

Thank you for this comment. Skin pigmentation arises from a complex interplay of three primary pigments: melanin, hemoglobin, and carotenoids, along with other components like collagen. In our study, we focused on the dimension of skin pigmentation linked to melanin, which spans from light to dark, given its salience and significant variation among human populations. Our method involved creating prototypes based on the average skin color values of each group and then using the differences between these prototypes as a "mask" to generate the two skin color versions. Consequently, because our manipulation was based on broad average values, the resulting stimuli exhibited variations not solely in the lightness parameter but across all dimensions; in essence, since the skin color dimension we focused on is inherently embedded within the broader context of "skin pigmentation", and given that we didn't exclusively modify lightness while controlling over other parameters, we chose to use the term "skin pigmentation" instead of "skin lightness." On the other hand, we need to make it clear that we calculated the CIELab values to determine if the skin color in each of the two groups (natural lighter from European American faces, and natural darker from Me´phaa faces) had observable and contrasting color properties. Here, we found that color properties effectively differed between groups. However, we chose lightness as it was the color parameter that contrasted the most in our groups and it could also influence other properties (like yellowness). We explained this in the manuscript, see lines 203-2010, and in 213-217.

Now, the statement we made in line 222(i.e., previous version line) is well-founded. Alaluf et al. (2002) compared measurements of human skin color (CIELab parameters) with biochemical measurements of melanin content, composition, and melanosome size in skin biopsies. They discovered a robust negative correlation between lightness and total melanin

content, as well as a weaker yet significant negative correlation between lightness and melanosome size. Although a positive correlation between yellowness and total melanin content was identified, it appeared to be influenced by lightness. Additionally, no other substantial correlations were found between yellowness and the other biological measurements. Given these considerations, we believe that this parameter best represents the dimension we focused on in our objectives. Nevertheless, for a better understanding of the use of this parameter in our study, we removed the association of this parameter with the biochemical relationships with melanin, since, in line with the reviewer, other color parameters could be affected by this pigment, and because determining the specific relations between the three parameters of color and the social perceptions is just out of the scope of this study (Please see lines 212-217). Having said this, we also believe that investigating how each facet of skin color contributes to the perception of this population could open up intriguing avenues for further research. This notion is elaborated upon in the discussion (Line 543-548).

Comment:
2. The creation of facial stimuli is unusual. Two averaged faces were created first and then the selected eight facial stimuli were either added or subtracted from the averaged face. Is there any reason for doing that? It is also not clear how the skin colour manipulation was done (lines 233-242). As the selected eight faces have different face shapes/ features/ colours/ colour distributions from the prototype, how the colour was added or subtracted without holding the facial shape unchanged? Whether the colour change was also applied to the facial features (eyes, mouth, brows). Any process for those facial features? Why was 70 per cent particularly selected? What software was used? It would be helpful to specify the detailed process.

Response:
We appreciate the comment. The software we used was Psychomorph and we did specify this information in the section named "Skin color facial stimuli" (now lines 218-220). Psychomorph has been used in numerous studies on face perception since it allows the independent manipulation of shape and color properties in facial photographs. To conduct the manipulations, the initial step involves outlining the facial contours of all photographs. Subsequently, two prototypes are fashioned, each representing the averaged values of the extreme points corresponding to the facial feature targeted for manipulation. Finally, using these two prototypes as reference, it is possible to create different versions of the same stimuli varying the facial feature based on the differences between the two prototypes (for a deeper understanding about how the software works and its details, we strongly recommend entering the following link: https://users.aber.ac.uk/bpt/jpsychomorph/).

Now, the sum or subtraction was made applying in each face the two color "mask" of the color prototypes. This was previously described and clarified in the section "Skin color facial stimuli" (Lines 218-239). Moreover, the setting of 70% of difference was used as a rule of thumb that the software developers recommend in order to avoid visual aberrations. This percentage not only prevents these visual aberrations to happened, but also ensures a significant change in color properties among the stimuli within the observable and feasible ranges for each ethnic group.

Finally, just like the unintended effect that occurred in texture homogenization because of the methodology we employed, a similar effect was observed in the color transformation, which extended to the eyes, mouth, and eyebrows. Our strategy to address this side effect involved comparing the ratings of the original versions with those of the corresponding versions with transformed skin color. This helped us measure the impact of the color homogenization effect. Nevertheless, we clarified this limitation in lines 531-548

Comment:
3. Apart from lightness (L*), the facial stimuli seem to show a large difference in yellowness (b*). Is that true? Since authors already 'measured its skin colour values in CIELab colour space (line 218)', it would be necessary to show the colour parameters in CLELAB colour space (including L*, a*, and b*) for all the face stimuli from both European American and Me'phaa groups, not just L*.

Response:
We appreciate this comment. As mentioned earlier, our calculation of the CIELab values primarily served as a validation method for selecting faces and for our manipulation process. In the response to comment 1 you can find all the information regarding why we used "L" as well as the lines manuscript we modified to clarify this important observation. Additionally, emphasizing one of the three color parameters based on its significance to the study is a customary practice in facial perception research (as seen in Kleisner et al., 2017).

Comment:
4. It's not clear about the colour information of facial stimuli. Whether the facial stimuli used in this study could represent the naturally occurring skin colour variations in the two populations, European American and Me'phaa. The average colour or the prototype is not enough as the variations within the same ethnic group are usually larger than the variations across the two ethnic groups. See the below reference. Thus, it would be very helpful to show the evolutionary meaningful skin colour range and variation of the real human faces from those two groups.
"Characterising the variations in ethnic skin colours: a new calibrated database for human skin," Ski. Res. Technol., vol. 23, no. 1, pp. 21–29, Feb. 2017, doi: 10.1111/srt.12295.

Response:

Thanks for this comment. Although our study standardizes skin color towards an "average" color, we also included the "Natural" group; which mantain individual variations for each ethnic group (8 natural Me'phaa faces and 8 European American faces). When comparing the natural group (with individual color variations) and its corresponding "average" group, perceptions don't change significantly, except for health. This might partially tell us that intra-groups color variation has less impact than the categorical (and experimental) change from one "average" skin color to the other one (i.e., Dark to Light and vice versa). We also included random effects for each evaluated facial photograph in our models. Here, we noticed that this random effect plays an important role in perceptions, but the fixed effect (transformed groups) consistently affects these individual variations across the groups. This demonstrates a systematized effect of contrasting "skin" color groups, despite the random

effect of natural variation. Additionally, the obtained average skin colors are within the typical range found in Mexican and European American populations. We've rewritten a section to clarify our rationale (lines 201-208 and 211-215).

Regarding the acknowledgement of the evolutionarily meaningful skin color range and variation of real human faces, we did provide a summary of human skin color evolution in the first section of the introduction (lines 75-90). Here, we emphasize that studying the evolution of human skin color has many aspects that have become more complex to describe the whole color diversity in human groups (e.g., microclimates, migration patterns, angles of UV radiation, etc.). Many of these factors are related to natural selection. In our case, we documented how the perception of human skin color, a trait shaped by natural selection, may have implications in other processes like sexual and social selection, as well as cultural transmission. This has already been clarified in the introduction and discussion.

Comment:

5. Line 240-242: 'The average lightness value was L* = 66.74, SD = 1.37 in the light skin colour transformed versions and L* = 52.75, SD = 2.58 in the dark skin colour transformed versions.' Are the light versions including both light European American and light Me'phaa faces and the dark versions include both dark European American and dark Me'phaa faces? If so, the standard deviation looks very small. Does that mean the European American and Me'phaa faces have the same lightness levels after manipulations? How does that come if both their original lightness and the prototype are different? It's better to give the actual colour distributions of the 48 face stimuli, not only the average lightness values.

Response:

Thank you for bringing up this point. Effectively, our study employs a crossover design. Hence, we obtained a total of 6 groups, which include a version darker and a lighter for each natural color group. Moreover, as we've clarified before, the process of manipulating skin color entailed applying a "color mask" derived from the two skin color prototypes. Consequently, within these versions, the transformed groups with paired colors (2 lighter and 2 darker) did not show significant variation in lightness. This outcome aligns with expectations, given that the color manipulation was conducted in a similar manner for both natural color groups. Furthermore, we observe that the lightness (L) of the transformed color groups (darker and lighter) remains consistent with their corresponding natural groups, as well as with what is commonly found and naturally distributed in both populations. We have provided clarification on this topic in lines 211-215 and 228-235 to enhance comprehension.

Comment:

6. It looks like the same facial stimuli changed not only the skin colour but also some other facial appearance traits after manipulations. E.g. in the below, the stimuli FC16, FC16co, and FC16cb are manipulated versions from the same original face, FC10, FC10cb,

FC10co are from the same face. If that’s the case, it looks like the beard completely changed after the colour changed, especially for the light version, the beard disappeared in the meantime. Those appearance traits could significantly influence sexual dimorphism and are even more sexually dimorphic traits than skin colour, and thus affect social judgements, especially judgements from the other gender.

Response:

Although the effect of facial sexually dimorphic traits, such as beard, is widely acknowledged, in our study it did not seem to be a significant factor. In the next forest plot we show the perceived attractiveness random estimates for all three skin color versions for each of the 16 faces (form right to left: natural version, light skin color version, dark skin color version).

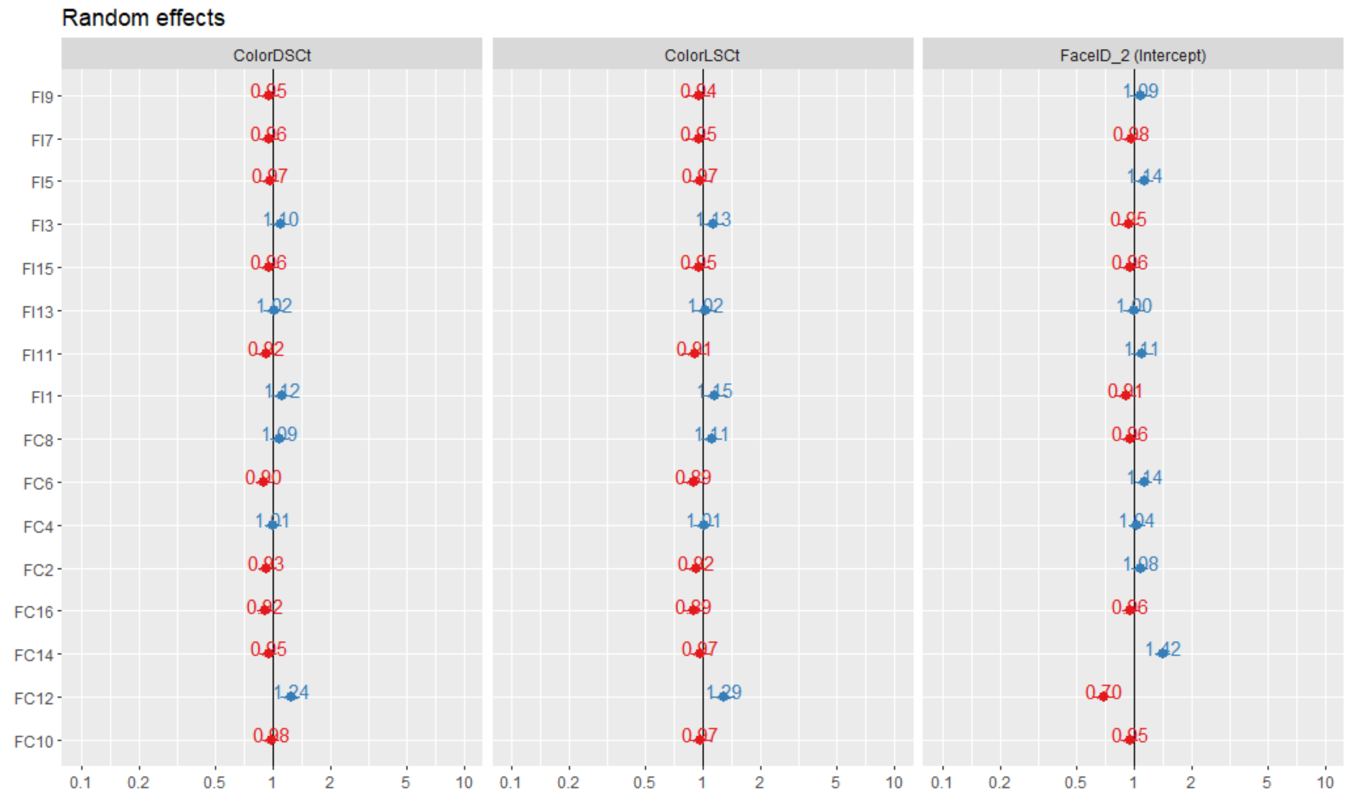

The forest plot shows that none of the two natural versions (FC10, FC16) got significant higher or lower ratings than the main estimate (right column). It also shows that removing the beard did not affect the rates significantly in comparison with the main estimate for each version (light skin color version, middle column; dark skin color version, left column). We have performed the same exploration for all response variables, and we found a similar result for all of them except for the perceived masculinity ratings, however, even those differences were not significant. The rest of the forest plot can be found in the R Markdown code lines 364, 594, 720, 851, and 984).

Comment:
7. The facial gloss could have a big influence on the lightness measurement results based on image methods. E.g. the stimulus FI15 below seems to have a considerable area of highlight. Within these areas, the colour could be completely different from the normal skin colour. Any considerations regarding the facial gloss issue? And how was the gloss morphed into different colour versions?

Response:
Thank you for the observation. The skin color prototypes were created by averaging the values of 30 faces of each group. One of the reasons why we had that sample size was to minimize the effect of any kind of individual variation such as the one mentioned; moreover, we did not use the actual lightness values to test the effect of skin color. Now regarding a possible difference in the way the raters perceived this stimuli in all of its versions, a random effect exploration showed that there are no significative differences between the scores this stimuli got and the main estimate for each version. We have included foster plots that show this in the R Markdown (code lines 364, 594, 720, 851, and 984).

Comment:
8. Images were captured under different environments and camera settings. It would be good to give information on settings, such as the white balance, CCT, ISO, aperture size, shutter speed, image formats (raw or post-processed), any colour characterization process, etc.

Response:
We appreciate this observation. We understand the relevance of giving as much information on settings as possible, however the information written in the lines 190 – 200 is all the setting information we took. Regarding the color characterization process, we followed the color calibration methodology reported in lines 208-217 which is a common method in the facial perception studies.

Comment:
9. During the experiments, participants were asked to rate the face without particular restriction or focus, e.g. based on the facial skin colour. In such a case, it's only meaningful to compare the rating of the lighter, natural, and darker versions of the same face. It's not reasonable to compare different faces or faces of different ethnic groups as the influence of structural facial traits is tangled together. However, those structural facial traits are not quantified or included in the analysis. in fact, previous studies show colour is less important than the structural facial traits in face perceptions. Meanwhile, various facial colour cues may be involved in face perceptions. See the reference below. E.g. the skin colour variation may matter a lot (homogeneous skin is preferred), which links to the colour and texture homogenization mentioned in the current study. Are there any considerations in the data analysis related to those confounding variables in visual stimuli? "Different colour predictions of facial preference by Caucasian and Chinese observers," Sci. Rep., vol. 12, no. 1, p. 12194, Jul. 2022, doi: 10.1038/s41598-022-15951-8.

Response:
We appreciate this comment. A complete explanation about why we compared the ratings between the natural versions of the European American and Me'Phaa faces can be found in the answer to the comment number 15. Now, regarding the considerations to the possible confounding effect of shape and color homogenization, we took different approaches. We designed a crossing over study where the skin color version is nested within the group in order to control the effect of facial shape; and we did so by creating different skin color version of the same facial stimuli. On the other hand, we are aware that one of the side effects of our protocol was the homogenization of color and texture, and we know this may have an effect on some of the perceptions we evaluated, so we compare the ratings of the natural versions with the ratings of the concordant skin color transformed version so we could have a measurement of this effect. Our results showed that, whereas skin color and texture homogenization seemed to affect some the perceptions, especially perceived health, in general the effect was either not significant or lower that the one due to skin color change. Nonetheless, we recognize this limitation and, hence, we highlight it in the discussion (lines 531-548).

Comment:
10. In the linear mixed effect model, the face colour was coded as categorical variable. But the same colour category has different lightness levels, e.g. the natural European American face and the natural Me'phaa face have different L*, and the lighter versions of different faces have different L*... Why not use the actual lightness (L*) value as a continuous variable in the lme analysis? Actual lightness value could be a more accurate measure for assessing the effect of skin lightness on impression judgements. The fixed effect of other colour parameters, such as a* and b*, could be included in the lme analysis as well.

Response:
We coded the skin color as a categorical variable because it is a more reasonable way to carry out the statistical analysis of a crossover research design. In addition, using lightness values as a continuous variable would present some major inconveniences for the data that we have. Firstly, continuous variables cannot be included as random factors, so we could not be able to deal with the lack of independency of our observations (the ratings come from three versions of the same faces). Secondly, since the skin color manipulation was based on average values, the lightness values´ distribution would behave in a "categorical" fashion, that is, the values would group in three points instead of being constantly distributed in the whole range of variation. The decision about what type of variable most be used is not based on the accuracy of the measurement but in the type of variable that fits with the specific needs of the research design.

Comment:
11. What's the observer variation? Any evaluation of the consistency across different raters.

Of the observer variations we assessed, factors such as sex, age, sexual preference, self-perception of ethnic group, and skin color were included. These variations and their effects are reported in the section titled "Facial Perception Rates." Given that we recognize the

relevance of the individual observer variation more than variables we measured them, we also decided to include “Rater” as a random factor in our mixed models. We know that, traditionally, a measure such as the Cronbach's alpha is calculated to know the participants response reliability. However, we think that our approach is better because mixed models do not only measure this sort of variance but also take it into account when they estimate the standard errors.

Comment:
12. Are there any differences in the results rated by mestizo participants and European participants? Is there any effect of the participants’ ethnicity on ratings?

Response:
We appreciate this question. We did not find that the raters’ ethnicities had an effect on their responses; and, as a matter of fact, this question was already asked by the first reviewer during the first submission and we wrote our answer with the whole process that led us to our conclusion in the statistical analysis section, lines 313-318.

Comment:
13. What’s the relationship between participants’ self-perceived skin colour ratings and their actual skin colour? Has their skin colour ever been measured by instruments like spectrophotometers? There might be a mismatch between self-reported and measured values.

Response:

This is an interesting question. Based on the literature available we do believe that there may be a mismatch between self-reported, reported by someone else and objective measures of skin color in Mexico and in other Latin American countries. In these regions, individuals could perceive themselves as lighter compared to others' perceptions and even lighter than what objective measures indicate. Despite the widespread theoretical consideration of this bias, to our knowledge, no studies have directly addressed this matter within Latin American populations. This highlights a significant knowledge gap that presents a substantial opportunity for investigation. With that being said, we deem this concept to extend beyond the scope of our current study, and we recognize that a completely new study is required to comprehensively examine and test this hypothesis.

Comment:
14. It would be interesting to know whether there are any significant differences in the structural facial traits (facial shapes and facial features) between the European American and Me’phaa faces, and what are the differences. It might be good to give some evidence based on the image database used in the present study.

Response:
This is an interesting question. A recent study found that some components of facial shape do vary across populations (Kleisner et al., 2021), and ethnicity is usually a significant predictor of facial shape variation in geometric morphometric studies. Therefore, we do

expect differences in the facial shape traits between the European American and Me'phaa faces, nonetheless, similar to the previous answer, we think that this idea is beyond the reach of our study. In this study we only focused on controlling the effects of shape, however, as suggested, we are currently working on a study on facial shape, using the same image database, where we sought to test the effect of shape variation on the same perceptions, and we model the facial shape configurations that best correlate with each of the perceptions in combination with skin color

Comment:

15. Both the abstract line 51-53 ('We found that….faces of …were.. more attractive….') and the discussion line 476-479 ('In comparison with….'.) gives an unreasonable conclusion. This conclusion is not justifying the effect of skin colour on facial impression but makes judgements which are out of the scope of the current study. It's hard to tell whether those comparisons were made based on the L* or other skin colour information or texture or the structural facial traits of the two ethnic groups. From what has been written, the scope of this study is to understand the role of skin lightness on face perceptions, not to compare the attractiveness of the two different ethnic faces. And it's not rational to make those comparisons on the bases of very different both structural facial traits and colour traits.

16. Similar issues for those statements in the results part: European American faces were rated 'x times' more attractive/trustworthy… than Me'phaa faces. Current data and analysis can only show the effect of skin colour, but cannot tell whether those 'x times' come from colour or other factors, also cannot prove the link between 'which ethnic face is more attractive' and 'colour plays a role in it'.

Response:

We agree with these two reviewer comments. Indeed, the focus of this study is to provide experimental evidence of how skin color affects social perceptions in a Mexican population, not to compare social perceptions of two ethnic groups. These groups not only differ in skin color but also in many other physical and cultural attributes, as previously mentioned. To make this objective clearer, we've made changes in the lines the reviewer mentioned and through the whole manuscript, directing the interpretation towards skin color and away from other facial or cultural attributes specific to ethnicity. Please see lines 54-57, and the results section for these modifications.

On the other hand, we used the term "x times" because it is the accurate way to describe differences when the estimated effect comes from a Poisson GLM. In this type of GLM, the effects are expected to be exponential. Therefore, when incorporating a categorical predictor, the estimates are expressed in terms of how many times it is greater or lesser compared to the baseline, instead of a fixed and continuous difference. Although we retained these terms ("x times"), we shifted the focus to skin color (evaluated in the L dimension) and not towards an "ethnic group." Thank you for this important observation.

Comment:
17. Whether the current results lead to the hypothesis? Skin colour preference/social preference also includes some mainstream aesthetic criteria, e.g. tanning skin is preferred in Western countries while whitening skin is preferred in Eastern countries, which may have nothing to do with racism or discrimination. Have these factors been considered in the current study? From the current data and analysis, it seems to be hard to elucidate the convincing mechanisms or drives behind the phenomenon. More survey and investigation is needed to know the clear answer.

Response:
This is an interesting question. We agree with the statement that some mainstream aesthetic criteria may play a role in the skin color preferences and that preference for tanning skin in Western countries is one example of it. However, skin color-based stratification and racism were in the very foundation of Latin American societies and still have strong effects on their social dynamic. Therefore, we think this is a more plausible factor that could explain the social preferences reported here. Nonetheless, we share the opinion that more investigation is needed to elucidate the relative contribution of mainstream aesthetic criteria on skin color preferences.

Comment:
18. Are there any considerations related to the adaptation to the living environment? Raters might be exposed to different environments composed of a variety of coloured faces. The long-term adaptation to the environment might influence their perceptions. It might be helpful to provide some evidence or data about the variety/proportions of faces in the participants' living environment.

Response:
Thank you for this observation. There are some studies that suggest that people are better at judging familiar faces, such as faces within the same ethnicity. We did propose this possibility as an explanation of the differences we observed in the effects of skin color between the two groups. We have rearranged the paragraph we had previously written to make it more visible (lines 549-557). It is important to point out that our discussion comes from an approximated measurement because there is not available data that allowed us to test this more precisely.

Comment:
19. Line 90: it would be more precise to say 'lower and more seasonal UVR'.

Response:
Thank you for this observation. We have changed the term.

Alaluf, S., Atkins, D., Barrett, K., Blount, M., Carter, N., & Heath, A. (2002). The impact of epidermal melanin on objective measurements of human skin colour. *Pigment Cell Research, 15*(2), 119–126. https://doi.org/10.1034/j.1600-0749.2002.1o072.x

Kleisner, K., Kočnar, T., Tureček, P., Stella, D., Akoko, R. M., Třebický, V., & Havlíček, J. (2017). African and European perception of African female attractiveness. *Evolution and Human Behavior*, *38*(6), 744–755. https://doi.org/10.1016/j.evolhumbehav.2017.07.002

Kleisner, K., Tureček, P., Roberts, S. C., Havlíček, J., Valentova, J. V., Akoko, R. M., Leongómez, J. D., Apostol, S., Varella, M. A. C., & Saribay, S. A. (2021). How and why patterns of sexual dimorphism in human faces vary across the world. *Scientific Reports*, *11*(1), 1–14. https://doi.org/10.1038/s41598-021-85402-3
