# Supplementary material for: Effects of facial skin pigmentation on social judgments in a Mexican population
Source: PLoS One. 2023 Nov 30;18(11):e0279858. doi: 10.1371/journal.pone.0279858 (PMC10688750; doi:10.1371/journal.pone.0279858)
Supplement: Attachment — Submitted filename: Response R1_Jaaziel_PLOS_2023.docx [file pone.0279858.s004.docx]

**Response to reviewers**

Effects of facial skin pigmentation on social judgments in a Mexican population

Jaaziel Martínez-Ramírez, David Puts , Javier Nieto, Isaac G-Santoyo^*^

*Corr. Author.

**Reviewer 1: Edward Telles**

-This is an important topic. The increasing interest and research on the topic is largely about the growing acknowledgment of the importance of race and color in Mexican society, by scientists and the general population. However, this analysis though seems to be largely of an evolutionary biological view with only a nod to ongoing racism and discrimination. Indeed, I could not find any use of “racism” but rather the use of “colorism.” When colorism is acknowledged, the authors usually refer to historical colorism under colonialization, which is learned across generations. As if racism and discrimination no longer exist. There is no consideration of the role of a Western ideology of race and racism and particularly, how Mexican elites, narratives, media, etc, shaped an ideology of mestizaje, which downplayed racism. See a considerable and growing literature by Patricio Solis, Regina Martinez Casas, Alan Knight and several others that shows the history of race in Mexico and how individuals use color to evaluate others in the labor market and other arenas. Indeed, the authors cite the PERLA studies but seem to overlook its findings about discrimination and inequality. Contrary to their claim that a social classification system based on phenotype no longer exists (p.5), these studies show that it is very profound in Mexican society, like it is throughout the Americas. I found it odd for, example, the claim that indigenous women chose to mate with Spanish men simply because they wanted to give their offspring better chances. What about the role of power or even rape?

**Response:**

We appreciate this comment. Indeed, our manuscript is in line with the perspective of the reviewer. The expression of various forms of racism in modern Mexican society is evident, leading to profound discrimination against individuals with physical attributes resembling pre-Columbian populations, with skin color being the most prominent attribute. Correspondingly, our study provides experimental evidence for the first time in a Mexican population that a relationship exists between "light" skin color and subjective preferences for favorable social traits such as attractiveness and reliability. Our manuscript aims to neither conceal nor distort this social effect; instead, it contributes to the extensive field of study in this area.

Based on the framework of behavioral ecology, social evolutionary psychology, and cultural evolution, our study aimed to test two non-mutually exclusive hypotheses explaining the effects of facial skin pigmentation on social judgments in the Mexican population. In the so-called social learning hypothesis, we proposed that this psychological mechanism underlies the preferences for lighter skin color and its association with social benefits, prestige, or deference. This association originated during the colonial period through the racial/color hierarchy system known as "castas," which placed the European colonizers at the top of the social hierarchy and the indigenous population at the bottom.

Therefore, we propose that skin color has led to the hierarchization of social benefits within the Mexican population through an extensive learning process that originated with the conquest. This process has influenced the ancestral evolutionary function of this physical attribute, from a mechanism of filtering UV radiation or synthesizing vitamin D to being an essential factor in social selection. In line with this, we do not deny that racism, colorism, and discrimination are also outcomes of this process and that nowadays, the cultural preferences for lighter skin color and other European phenotypical features may be reinforced by these sociological mechanisms. Our explanation from an ecological/evolutionary perspective does not contradict other authors from different fields of knowledge. Instead, it complements its study, not only due to the implementation of an experimental design that modifies skin color and controls for other confounding variables but also because of the theoretical argument that unifies the same observed phenomenon of racism through its observation in one of its facets related to subjective preferences determining different types of social interaction.

Additionally, although we did not explicitly use "racism," we considered "colorism" a more appropriate word to describe the effect observed regarding skin color, since we did not delve further into the implications of racism or other types of discrimination as it falls outside the scope of this study. Nonetheless, colorism, a form of prejudice or discrimination based on skin color, is evident, whereby individuals are treated differently based on this physical attribute, with lighter skin tones often being favored or perceived as more desirable than those with darker skin tones. Colorism can manifest both within racial and ethnic groups (as observed in our study) and between them, resulting in various negative impacts, such as reinforcing harmful stereotypes and creating disparities in education, employment, and social status. It is often interconnected with broader issues of racism and discrimination, making it essential to address in pursuing social justice and equality.

Consequently, we have extensively rewritten a significant portion of the introduction and discussion sections to clarify our stance ( for example see lines 113-131 and 502-513). . Moreover, we have expanded the information regarding previous studies demonstrating different racial features within the current Mexican population. Here, we have emphasized modifying the phrase "no longer exist" to more clearly convey that these preferences continue to permeate the psyche of the contemporary Mexican population, at least based on our evaluated sample (>700).

-The authors focus on the evaluations according to particular personality dimensions. They should consider the role of stereotypes as in how indigenous people are considered ugly -score low on attractiveness (note the recent reactions to the actress Yalitza Aparicio) and stereotypes about not being trusted and being sickly (two other dimensions studied). These findings are consistent with the use of ethnic/racial stereotypes. Aggressiveness may also be according to the idea of indigenous as savages. The dimensions of masculinity and dominance are less clear. Racism and stereotypes about indigenous people should certainly be considered at least as a hypothesis, which may compete with hypotheses about social learning, etc. The color ratings of the European faces is also consistent with ratings as they may appear more indigenous.

Response: We appreciate this comment. Indeed, our results could be closely linked to racism and stereotypes; again, our intention has not been to deny these concepts but to complement them. In this line, we do not posit racism and stereotypes as a hypothesis competing with the hypothesis of social learning but rather as a possible consequence of this historical-social learning, which determines what is deemed "good" or "bad" based on skin color.

What is particularly interesting here is that the rater's participants had no information about the ethnicity of the presented stimuli. Therefore, we filtered out any information that could promote stereotypes by the evaluators beforehand, to observe the exclusive effect of skin color. In our experiment (please see the referred web page in line 268- labneuroecologiacog.com), we did not use labels or any other indication to suggest that the face belonged to a European American or a Me'Phaa man. Hence, if a rater had any stereotype bias towards Indigenous people, it would be harder for them to recognize the face as Indigenous. We also applied masks to all facial stimuli to minimize potential confounding factors.

Consequently, our results demonstrate a shift in participants' perceptions when skin color is oppositely modified to the original ethnic group. This change shows that a light-skinned face was evaluated positively on socially favorable dimensions (such as attractiveness and trustworthiness) and negatively on "unfavorable" dimensions. This effect was regardless of the stimuli' facial shape, the rater's preexisting stereotypical prejudices, the original ethnic group of the facial stimuli, or the rater's self-perception of skin color or ethnicity. We clarified this information in the discussion, also emphasizing that the approximations of racism and stereotypes evaluated here could be, in part, a consequence of this historical-social learning.

-As far as the analysis, I think it is mostly well done but I am surprised that the color or the ethnicity of the raters was not controlled (See Hill, Mark E. "Race of the interviewer and perception of skin color: Evidence from the multi-city study of urban inequality." American Sociological Review (2002): 99-108. The authors claim that the rater color was concentrated in a few colors and so they didn’t control that. I don’t understand why that should be important. Also, if raters are indigenous, they may evaluate in a different way. By the way, there seems to be a hesitancy to call the Me’Pha “indigenous.” For these Mexican raters, I imagine stereotypes are based on indigeneity and they may have little or no familiarity with that particular ethnic group.

**Response:**

The data collected from self-reported ethnicities and PERLA scale responses indicated that 84% of the raters identified as Mestizos, and 78% chose levels 4-6 on the PERLA scale. The remaining 16% and 22% (respectively) were divided among four other ethnic groups and seven skin color levels. It's worth mentioning that some combinations had very low percentages, such as the self-reported ethnicity "Afro-American" at only 0.3%. This low percentage of other ethnic groups and scores on the PERLA scale suggests that our sample is quite homogeneous. Here, variables with low representativeness, such as these two mentioned, lead to heterogeneity of variance due to the unbalanced data in the sample size of each predictor included (i.e., ethnicity with five levels and PERLA scale with 11 levels). This unbalanced data and over-parametrization are significant structural violations when using linear models (Zuur, Leno, & Elphick, 2010; Faraway, 2016). However, following the reviewer's observation, we conducted the method described by Zuur, Leno, and Elphick (2010) to investigate the possible effects of these two variables. After the model validation process, we plotted Pearson residuals of each selected model against participants' self-reported ethnicities and the PERLA scale responses for self-perceived skin color (the box plots can be found in the R Markdown, code lines 263-264 for the attractiveness plots, 455-456 for the dominance plots, 557-558 for the masculinity plots, 681-682 for the aggressiveness plots, 806-807 for the trustworthiness plots, and 936-937 for the perceived health plots). Due to the plots did not show any effect, and to avoid structural errors in the design of linear models, we did not include either of these two predictors. We briefly explained our decision in the statistical analysis section (lines 326-330).

Finally, following the reviewer's observation, we already had included self-perception of ethnicity as "Indigenous" rather than particular ethnic groups, such as "Me'Phaa"( Please see line 191).

-I would refer to raters as “raters” or “participant raters rather than “participants.”

**Response:** We changed the heading “Participants” with “Participant raters” in all manuscript.

References:

Faraway, J. J. (2016). *Extending the linear model with R: generalized linear, mixed effects and nonparametric regression models*. CRC press.

Zuur, A. F., Ieno, E. N., & Elphick, C. S. (2010). A protocol for data exploration to avoid common statistical problems. *Methods in ecology and evolution*, *1*(1), 3-14.

**Reviewer #2: Peter Frost**

**-** “Its main difficulty is that of distinguishing between gendered and ethnic significations of skin color. The two have long coexisted in Mexican society. The authors have tried to control for ethnic significations by varying skin color within each ethnic group. The results of that strategy are mixed. It seems to me that ethnic significations contaminated the raters’ perceptions of the European American faces to a greater degree than they contaminated the raters’ perceptions of the Me’Phaa faces.”

**Response:**

We appreciate this precise observation. We propose two main reasons why we observed different results in the effects of skin color transformation between the European American and Me'Phaa faces, both closely related to the suggestions made by the reviewer.

The first possibility is that the participants may have rated the stimuli differently due to varying degrees of familiarity with the facial features of the two ethnic groups. Here, raters participants were asked to rate facial stimuli from a closely related ethnic group (Me'Phaa faces) and stimuli from a more distant ethnic group (European American faces). In this case, the effects on subjective perceptions were more evident when changing skin color within the more familiarized groups. However, although we found a similar effect on European American faces, it was not statistically significant in several social perceptions.

The second possibility is attributed to a potential artifact in our method to modify the skin color. In this case, the manipulation process outcomes could have differed slightly between the European American and Me'Phaa faces. To deal with this, we minimized the standard error by fitting random slopes for skin color transformation for each facial photograph ID. However, it is essential to highlight that there is always a risk of such artifacts in any manipulation process. We added a new paragraph in the discussion summarizing these possible explanations and limitations (lines 549-562).

**-**There are a few errors:

- The reference to Green and Martin (1990) does not support footnote #27

- The adjective “perceived” should be used in all of the headings, and not just “perceived health.” All of the qualities are perceived.

- Since perceived aggressiveness did not differ significantly between the two groups of faces, it should not be presented in the Discussion section as a perceived quality that differs between the two groups.

-In general, the wording is awkward. I would suggest the following corrections.

-Lines 83- 93 – replace the four sentences with: “The main pigments—melanin, hemoglobin, and carotenoids—absorb and reflect different wavelengths of light within the visible spectrum. Collagen also contributes by scattering light [10]. The skin’s pigments vary not only between individuals and populations but also between men and women, perhaps because of sexual selection [11]. Natural selection initially favored darker skin as a means to protect against the harmful effects of UV radiation in the tropics. The pressure of selection then shifted toward lighter skin among those humans who spread into higher latitudes with lower levels of UV [12-14]”

-Footnote #10 – add:

Edwards, E.A., and S.Q. Duntley. (1939). The pigments and color of living human skin. American Journal of Anatomy 65(1): 1-33. <https://doi.org/10.1002/aja.1000650102>

-Line 102 – replace “others” with “other”

-Line 110 – replace “preference honestly contributed to reproductive outcomes” with: “preference significantly contributes to reproductive success”

-Lines 128-140 – replace with: “The sex difference is due to exposure of skin tissues to differing ratios of androgens to estrogens, particularly at puberty. Testosterone has a stronger effect than estrogen on melanin synthesis and vascularization of the upper dermis [26].”

-Footnote #26 - add:

Edwards EA, Hamilton JB, Duntley SQ, Hubert G. Cutaneous vascular and pigmentary changes in castrate and eunuchoid men. Endocrinology 1941; 28(1): 119-128. doi:10.1210/endo-28-1-119

Manning JT, Bundred PE, Mather FM. Second to fourth digit ratio, sexual selection, and skin colour. Evolution and Human Behavior 2004; 25(1): 38-50. doi:10.1016/s1090-5138(03)00082-5

-Footnote #27 - the reference to Green and Martin (1990) does not support the statement that “males undergo a more intense facultative pigmentation after sun exposure and retain it for longer periods than females whereas female skin lightens faster after a reduction in sun exposure.”

Replace the reference to Green and Martin (1990) with:

Harvey RG. Ecological factors in skin color variation among Papua New Guineans, American Journal of Physical Anthropology 1985; 66(4): 407-416. doi:10.1002/ajpa.1330660409

-Footnotes #28-30 - add:

Frost P. Preference for darker faces in photographs at different phases of the menstrual cycle: Preliminary assessment of evidence for a hormonal relationship. Perceptual and Motor Skills 1994; 79(1): 507-14. doi:10.2466/pms.1994.79.1.507

Line 140 – replace “have” with “has”

-Line 148 – replace “no-WEIRD” with “non-WEIRD”

-Lines 151-152 – replace with: “In this study, we used an experimental design with a Mexican population to determine whether facial skin color can influence certain social perceptions: attractiveness, …”

-Line 155 – replace “To accomplish this” with “To that end”

-Line 164 – delete the extra comma after “perceptions”

-Line 193 – replace “create” with “created”

-Line 240 – replace “into” with “within”

-Line 253 – replace “prevent” with “made sure”

-Line 254 – insert “not” after “were”

-Line 256 – replace “on” with “of”

-Lines 259-261 – replace with: “… version. In other words, we compared natural European American faces with artificial light-skinned and dark-skinned versions and natural Me’Phaa faces with artificial light-skinned and dark-skinned versions. We could thus measure how the participants perceived differences in facial color independently of the face’s ethnicity.”

-Line 264 – replace “in” with “on”

- 272 – replace “men participants” with “the male participants”

-Line 281 – replace “be” with “have been”

-Line 285 – replace “in” with “on the”

-Line 329 – replace “Attractiveness” with “Perceived attractiveness”

-Line 330 – replace with: “Perceived attractiveness differed significantly between the two groups when they had their natural skin color”

-Line 331 – replace “as” with “more”

-Line 332 – replace “as” with “than”

-Lines 332-333 – replace with: “Furthermore, there were effects when natural skin color was changed within each of the two groups. Lightening the Me’Phaa faces made them more attractive (…), and darkening the European American faces made them less attractive (…). Both groups became slightly more attractive when skin color and skin texture were homogenized (…).

-Lines 343-344 – replace “men participants” with “the male participants”

-Lines 344-345 – replace “women participants” with “did the female participants”

-Table 2 - replace “Models estimates” with “Perception estimates”
- Replace “Attractiveness perception” with “Perceived attractiveness”
- Replace “Trustworthiness perception” with “Perceived trustworthiness”
- Replace “Dominance perception” with “Perceived dominance”
- Replace “Aggressiveness perception” with “Perceived aggressiveness”
- Replace “Masculinity perception” with “Perceived masculinity”

-Line 357 – replace “Trustworthiness” with “Perceived trustworthiness”

-Line 359 – replace “as” with “more”

-Line 360 – replace “as” with “than”

-Line 361-363 – replace with: “In addition, lightening the Me’Phaa faces increased their perceived trustworthiness (…), whereas darkening the European American faces had no effect”

-Line 383 – replace “Perceived health” with “Perceived healthiness”

-Line 384 – replace “As in” with “As with”

-Lines 386-394 – replace with: “On average, natural European American faces were perceived as being 1.19 times healthier (…) than natural Me’Phaa faces (…). When facial skin color was changed within each ethnic group, the changes had different effects on perceptions of the two groups. Lightening the Me’Phaa faces increased their perceived health (…), whereas darkening the European American faces had no effect (…). Perceived health increased slightly for both groups when skin color and skin texture were homogenized.

-Line 409 – replace “Dominance” with “Perceived dominance”

-Lines 410-411 – replace with: “Unlike the previous qualities, perceived dominance was lower for European American faces that had their natural skin color”

-Lines 413-416 – replace with: “in contrast to perceived attractiveness, all faces received a lower dominance rating from male participants (…) than from female participants (…). Finally, we were unable to determine any effect from lightening, darkening, or homogenizing the faces, since these predictors …”

-Line 420 – replace “Aggressiveness” with “Perceived aggressiveness”

-Lines 421- 427 – replace with: “Although perceived aggressiveness was lower for the European American faces, the difference was not significant (…). Lightening the Me’Phaa faces made them seem less aggressive (…), but darkening the European American faces also made them seem less aggressive (…). Finally, perceived aggressiveness did not change for either group when skin color and skin texture were homogenized (…)”

-Line 442 – replace “Masculinity” with “Perceived masculinity”

-Lines 443-450 – “As with perceived dominance, perceived masculinity was lower for natural European American faces (…) than for natural Me’Phaa faces (…). Lightening the Me’Phaa faces made them seem less masculine (…), but darkening the European American faces had no effect (…). In addition, perceived masculinity did not change for either group when skin color and skin texture were homogenized (…).”

-Lines 469-472 (sentence) – replace with: “In comparison with natural Me’Phaa faces, natural European American faces were perceived not only as more attractive, healthier, and more trustworthy but also as less dominant and less masculine. They were furthermore perceived as less aggressive, but not significantly so.”

-Lines 484-486 – replace with: “… Mexican participants rated light-skinned faces more highly on all three aspects of social valence (attractiveness, healthiness, trustworthiness). In contrast, they rated dark-skinned faces more highly on two aspects of social dominance (dominance, masculinity).

-Line 493 – replace “well-remunerated” with “well-paid”

-Lines 495-498 (sentence) – “Consequently, a learned association between a physical trait—in this case, light skin—and social deference might establish such a perception in a population over several generations”

-Line 498 – replace “learning association” with “learned association”

-Lines 505-507 (sentence) – replace with: “Also, in Mexico and other Latin American countries, lightness of skin correlates with years of schooling, hourly earnings, and other indicators of well-being.”

-Line 511 – replace “dark skin color faces” with “dark-skinned faces”

-Lines 512-513 - replace “dark skin color faces” with “dark-skinned faces”

-Line 516 – replace the comma before “therefore” with a semi-colon

-Line 518 – insert “sexually” before “dimorphic”

-Lines 528-535 – Replace with: “For example, lightening the Me’Phaa faces made them seem more attractive, healthier, and more trustworthy. Conversely, darkening the European American faces made them seem less attractive. Other perceived qualities, however, did not change systematically when facial skin color was changed. Perceived dominance did not change when changes were made to facial skin color within either group. Lightening the Me’Phaa faces decreased their perceived masculinity and aggressiveness, but darkening the European American faces did not increase their perceived masculinity and aggressiveness.”

-Lines 541-557 - Replace with: “Moreover, we not only lightened and darkened facial color but also homogenized it by removing such texture-related aspects as spots, freckles, or wrinkles […]. Our results suggest that skin texture does have an effect, particularly on perceived health. That effect, however, largely exists independently of the effects of skin color. Skin texture might nonetheless interact with skin color in perceptions of light and dark skin, and such interactions could be an exciting avenue for further studies. In addition, unlike the authors of previous studies, we manipulated overall skin color instead of gradually changing each of its aspects (e.g., luminosity, hue) […]. Future studies could examine how each aspect of skin color contributes to the way a population is perceived.”

-Lines 558-574 - replace with: “In summary, we show that the Mexican population uses facial color to make certain social judgments. We found that a dark-skinned face is perceived as more dominant and more masculine. Those perceptions may be due to a process of coevolution between the human mind and the sex difference in skin color. One may therefore infer that male skin became darker through male-male competition rather than through female choice. We also found that a light-skinned face is perceived as more attractive, more trustworthy, and healthier. Those perceptions may be due to the association between skin color and social status that prevailed in Mexico during more than three centuries of colonial rule. Today, they are maintained by colorism. We are nonetheless speculating, and more data will be needed. Similar results could be obtained from other Latin American societies, which share a common colonial history and social structure.”

**Response:**

We greatly appreciate the reviewer's effort and time in providing us with precise suggestions, which were timely and significantly improved our manuscript. Therefore, we have rewritten all sentences and added all recommended references as per the reviewer's assessment. Once again, thank you very much.

**Reviewer #3: Vojtech Fiala**

-The overall positive impression is also promoted by detailed report on the fitted models, availability of the script and data. It seems like a good craft (although I must admit that I have not checked if the ‘necessary’ rituals of frequentist statistics have been gone through by the authors). While I recommend to study for acceptance (this being their final destiny), I still find some aspects of it problematic – and I think these should be considered at least by extending/re-writing some paragraphs within this otherwise good manuscript.

-Major objection 1: Why Poisson?

-First, I wonder, why the authors decided to use generalised linear model with the family set to "Poisson". The expectation that the dataset (data of the dependent variable) comes from Poisson distribution is not commented on in the manuscript. Picking 1-9 points is, in my opinion, not a count of rare events within interval (of whatever kind; how the parameters of such a distribution may be set?). While this objection may seem serious, it is not the case (given my expectations about the "intestines" of glm fit that are above the scope of the current text). Nonetheless, the authors should either justify their decision (maybe I am just wrong, and I'd appreciate being proven so, since this may positively affect my own subsequent work) or consider using different distribution for the dependent variable’s population, which has been sampled.

**Response:**

We appreciate this observation. We decided to use GLMMs with Poisson errors due to the nature of our data, which are discrete quantitative data derived from a Likert scale. We did not average the perception data (i.e. among participant scores for each face rated) because we consider that this data treatment could affect the measures of the central tendency of some facial perceptions; this was particularly true for the attractiveness rating distribution, where we observed a positively skewed counts-type distribution (the histograms can be found in the R Markdown, code lines 234-238). Because of this and the discrete nature of our dependent variables, we decided to use Poisson GLMMs instead of Gaussians, which we explained briefly in the statistical analysis section (lines 302-307).

Major objection 2: Culture vs… and biology vs… (?)

Another serious notion that shall be considered during revisions, refer to the lines 163-173 of the manuscript. I put aside that the sentences are long and hard to follow. I have the following concerns: The authors begin by stating that lighter skin that is naturally associated with a given ethnicity may be a cue to favorable characteristic (as they are stereotypically ascribed to a face, not to its colouration itself – colour is cue to ethnicity, which affect the ascribed characteristics). However, the opposite hypothesis would read: Maybe, it does not matter if the face is European/Native American (I mean, the origin of the face) and what matters is lightness (as an exact measure). This contrasting view (while I acknowledge cannot be directly addressed in this study) is not applied here. The authors rather switch from cultural to biological explanation: once the face is darker, it is perceived as more attractive, also dominant, aggressive, etc., due to androgens. Together, this seems for me to not make much sense. Consider rewriting this paragraph (even if it is just to help slow-minded readers, which I eventually may be, to understand).

Response:

Thank you for this observation. In this study, we tested three hypotheses, each of them having its null and alternative prediction. These three hypotheses could be mutually non-exclusive and, therefore, complement each other. In the first one we propose that social learning may be the psychological mechanism underlying the effects of facial skin pigmentation on the social perceptions. Here we sought to test whether light facial color ( and the opposite for dark facial color) promotes greater rate values in favorable social attributes, such as reliability, attractiveness or perceived health. This effect would be independent of the of the origin of the face, but only by the skin coloration. In our second hypothesis, we propose that these effects may rather be the results of ancestral sexual selection of an androgen-dependent trait such as skin color. Here, darker faces and not lighter would influence greater rates values in social perceptions such as attractiveness, aggressiveness, masculinity and dominance. If this is supported, we would expect that the experimental modification of the skin pigmentation would change the previous perceptions in both the European American and Me´Phaa faces. This means that, as noticed in the review, the study seeks to determine the role of skin color controlling for other facial features like shape. Finally, our third hypothesis suggests that neither lighter nor darker skin color would affect any social perception, neither in the original faces nor in stimuli with exchanged colors

We have rewritten this paragraph to make our hypothesis clearer (lines 170-186).

Minor notes:

-"There was no effect of the skin color manipulation or color exchange [for the perceived dominance] in any group.”

This is a surprising result, given the previously identified positive association between darker skin and dominance-related characteristics like masculinity. I suggest the authors to add a paragraph in the discussion in which they comment on the result. Consider the opposite result for perceived aggressiveness, which was not significantly affected by the ethnicity in non-manipulated faces, while it was affected by skin colour manipulation in both the groups in the anticipated direction (the same applies for masculinity).

**Response:** As suggested by Torrance et al. (2014) it seems that the facial shape and surface features, such as skin color, contribute differently to judgements of attractiveness and dominance (See Torrance JS, Wincenciak J, Hahn AC, DeBruine LM, Jones BC (2014) The Relative Contributions of Facial Shape and Surface Information to Perceptions of Attractiveness and Dominance. PLoS ONE 9(10): e104415. doi: 10.1371/journal.pone.0104415). They found that the perception of dominance was more strongly affected by facial shape features whereas the opposite was observed for the perception of attractiveness. Yet another possibility is that the skin color and facial shape effects on some of these perceptions interact in such a way that skin color becomes relevant only when the facial shape strongly elicits one of these perceptions. This may be the case for what we observed in the perceptions of masculinity and aggressiveness, since the skin color manipulation affected mainly the Me´Phaa faces which, in turn, were the ones rated as more dominant and masculine. That would mean that skin color cues become relevant for the perception of masculine and aggressiveness only on faces with shapes that strongly elicit these perceptions. We have rewritten a paragraph in the discussion where we summarized this idea (lines 531-548).
