# Supplementary material for: Effects of facial skin pigmentation on social judgments in a Mexican population
Source: PLoS One. 2023 Nov 30;18(11):e0279858. doi: 10.1371/journal.pone.0279858 (PMC10688750; doi:10.1371/journal.pone.0279858)
Supplement: Attachment — Submitted filename: comments.docx [file pone.0279858.s006.docx]

The topic is quite important. The present study adds to the growing body of studies addressing the importance of skin colour on facial perceptions and provides important psychophysical data collected from Mexican populations, which hasn’t been revealed before. The revision of the present paper has been improved a lot from the perspective of sociology, anthropology, and human Evolution. Whereas there are still some concerns that need to be addressed related to the colour parameter setting, image colour manipulations, data analysis, conclusions and discussions, etc. The paper could be published once the below issues are solved.

1. It’s a bit surprising that Lightness (L*) is the only parameter considered in this study whereas the aim is to investigate the influence of skin pigmentation on social judgments.

Obviously, the reason given in line 222 (lightness has a negative correlation with melanin) is not rational. In fact, melanin pigmentation is not only negatively affects the skin’s lightness, but possibly even more strongly and positively linked to skin yellowness. And it also influences skin redness. See for example the below reference. There are also other skin pigmentations. If it’s only lightness (L*) is investigated, the title and the scope should be restricted to the effect of ‘skin lightness’ not ‘skin pigmentation‘, on facial impressions. Otherwise, it’s not fair to study the influence of skin colour on facial impressions only based on L* values.

“Fruit over sunbed: Carotenoid skin colouration is found more attractive than melanin colouration,” Q. J. Exp. Psychol., vol. 68, no. 2, pp. 284–293, Feb. 2015, doi: 10.1080/17470218.2014.944194.

Kikuchi, Kumiko, et al. "Image analysis of skin colour heterogeneity focusing on skin chromophores and the age‐related changes in facial skin." Skin Research and Technology 21.2 (2015): 175-183.

2. The creation of facial stimuli is unusual. Two averaged faces were created first and then the selected eight facial stimuli were either added or subtracted from the averaged face. Is there any reason for doing that? It is also not clear how the skin colour manipulation was done (lines 233-242). As the selected eight faces have different face shapes/ features/ colours/ colour distributions from the prototype, how the colour was added or subtracted without holding the facial shape unchanged? Whether the colour change was also applied to the facial features (eyes, mouth, brows). Any process for those facial features? Why was 70 per cent particularly selected? What software was used? It would be helpful to specify the detailed process.
3. Apart from lightness (L*), the facial stimuli seem to show a large difference in yellowness (b*). Is that true? Since authors already ‘measured its skin colour values in CIELab colour space (line 218)’, it would be necessary to show the colour parameters in CLELAB colour space (including L*, a*, and b*) for all the face stimuli from both European American and Me’phaa groups, not just L*.
4. It’s not clear about the colour information of facial stimuli. Whether the facial stimuli used in this study could represent the naturally occurring skin colour variations in the two populations, European American and Me’phaa. The average colour or the prototype is not enough as the variations within the same ethnic group are usually larger than the variations across the two ethnic groups. See the below reference. Thus it would be very helpful to show the evolutionary meaningful skin colour range and variation of the real human faces from those two groups.

“Characterising the variations in ethnic skin colours: a new calibrated database for human skin,” Ski. Res. Technol., vol. 23, no. 1, pp. 21–29, Feb. 2017, doi: 10.1111/srt.12295.

5. Line 240-242: ‘The average lightness value was L* = 66.74, SD = 1.37 in the light skin colour transformed versions and L* = 52.75, SD = 2.58 in the dark skin colour transformed versions.’ Are the light versions include both light European American and light Me’phaa faces and the dark versions include both dark European American and dark Me’phaa faces? If so, the standard deviation looks very small. Does that mean the European American and Me’phaa faces have the same lightness levels after manipulations? How does that come if both their original lightness and the prototype are different? It’s better to give the actual colour distributions of the 48 face stimuli, not only the average lightness values.
6. It looks like the same facial stimuli changed not only the skin colour but also some other facial appearance traits after manipulations. E.g. in the below, the stimuli FC16, FC16co, and FC16cb are manipulated versions from the same original face, FC10, FC10cb, FC10co are from the same face. If that’s the case, it looks like the beard completely changed after the colour changed, especially for the light version, the beard disappeared in the meantime. Those appearance traits could significantly influence sexual dimorphism and are even more sexually dimorphic traits than skin colour, and thus affect social judgements, especially judgements from the other gender.

7. The facial gloss could have a big influence on the lightness measurement results based on image methods. E.g. the stimulus FI15 below seems to have a considerable area of highlight. Within these areas, the colour could be completely different from the normal skin colour. Any considerations regarding the facial gloss issue? And how was the gloss morphed into different colour versions?

8. Images were captured under different environments and camera settings. It would be good to give information on settings, such as the white balance, CCT, ISO, aperture size, shutter speed, image formats (raw or post-processed), any colour characterisation process, etc.
9. During the experiments, participants were asked to rate the face without particular restriction or focus, e.g. based on the facial skin colour. In such a case, it’s only meaningful to compare the rating of the lighter, natural, and darker versions of the same face. It’s not reasonable to compare different faces or faces of different ethnic groups as the influence of structural facial traits is tangled together. However, those structural facial traits are not quantified or included in the analysis. in fact, previous studies show colour is less important than the structural facial traits in face perceptions. Meanwhile, various facial colour cues may be involved in face perceptions. See the reference below. E.g. the skin colour variation may matter a lot (homogeneous skin is preferred), which links to the colour and texture homogenization mentioned in the current study. Are there any considerations in the data analysis related to those confounding variables in visual stimuli? “Different colour predictions of facial preference by Caucasian and Chinese observers,” Sci. Rep., vol. 12, no. 1, p. 12194, Jul. 2022, doi: 10.1038/s41598-022-15951-8.
10. In the linear mixed effect model, the face colour was coded as categorical variable. But the same colour category has different lightness levels, e.g. the natural European American face and the natural Me’phaa face have different L*, and the lighter versions of different faces have different L*... Why not use the actual lightness (L*) value as a continuous variable in the lme analysis? Actual lightness value could be a more accurate measure for assessing the effect of skin lightness on impression judgements. The fixed effect of other colour parameters, such as a* and b*, could be included in the lme analysis as well.
11. What’s the observer variation? Any evaluation of the consistency across different raters.
12. Are there any differences in the results rated by mestizo participants and European participants? Is there any effect of the participants’ ethnicity on ratings?
13. What’s the relationship between participants’ self-perceived skin colour ratings and their actual skin colour? Has their skin colour ever been measured by instruments like spectrophotometers? There might be a mismatch between self-reported and measured values.
14. It would be interesting to know whether there are any significant differences in the structural facial traits (facial shapes and facial features) between the European American and Me’phaa faces, and what are the differences. It might be good to give some evidence based on the image database used in the present study.
15. Both the abstract line 51-53 (‘We found that….faces of …were.. more attractive….’ ) and the discussion line 476-479 (‘In comparison with….’. ) gives an unreasonable conclusion. This conclusion is not justifying the effect of skin colour on facial impression but makes judgements which are out of the scope of the current study. It’s hard to tell whether those comparisons were made based on the L* or other skin colour information or texture or the structural facial traits of the two ethnic groups. From what has been written, the scope of this study is to understand the role of skin lightness on face perceptions, not to compare the attractiveness of the two different ethnic faces. And it’s not rational to make those comparisons on the bases of very different both structural facial traits and colour traits.
16. Similar issues for those statements in the results part: European American faces were rated ‘x times’ more attractive/trustworthy… than Me’phaa faces. Current data and analysis can only show the effect of skin colour, but cannot tell whether those ‘x times’ come from colour or other factors, also cannot prove the link between ‘which ethnic face is more attractive’ and ‘colour plays a role in it’.
17. Whether the current results lead to the hypothesis? Skin colour preference/social preference also includes some mainstream aesthetic criteria, e.g. tanning skin is preferred in Western countries while whitening skin is preferred in Eastern countries, which may have nothing to do with racism or discrimination. Have these factors been considered in the current study? From the current data and analysis, it seems to be hard to elucidate the convincing mechanisms or drives behind the phenomenon. More survey and investigation is needed to know the clear answer.
18. Are there any considerations related to the adaptation to the living environment? Raters might be exposed to different environments composed of a variety of coloured faces. The long-term adaptation to the environment might influence their perceptions. It might be helpful to provide some evidence or data about the variety/proportions of faces in the participants’ living environment.
19. Line 90: it would be more precise to say ‘lower and more seasonal UVR’.
