# Supplementary material for: Effects of facial skin pigmentation on social judgments in a Mexican population
Source: PLoS One. 2023 Nov 30;18(11):e0279858. doi: 10.1371/journal.pone.0279858 (PMC10688750; doi:10.1371/journal.pone.0279858)
Supplement: Attachment — Submitted filename: Response to Reviewers.docx [file pone.0279858.s007.docx]

**Reviewer 1:**

**Comment:**

This revision is an improvement but there are still two problems. The first is correctable and I am not sure about the second:

1. The study focuses on the evolutionary role of skin color in Mexican society. However, they hardly engage with the metaphorical “elephant in the room:” current societal skin color inequality and discrimination. While the literature review acknowledges societal inequalities based on skin color, mostly in the social psychology and sociological literature, the authors do not discuss how it fits with their own findings. Except for mentioning this literature (not until pages 5-6), there is no serious engagement. How do their finding co-exist with those of racial attitudes, stereotyping and racial inequality? The abstract, for example, does not even mention that. I expect that readers would want to know how these evolutionary findings may complement (or not) the social science findings. For example, I see complementarity in how the findings are consistent with stereotypes of indigenous people.

Response:

We appreciate the previous comments from the reviewer; they have been instrumental in enhancing the manuscript's writing. Furthermore, we consider that these two recent observations provide valuable information to enhance the emphasis we have aimed about inequality and social discrimination based on skin stereotypes, and on how our evolutionary framework and results complement (or not) the social science findings.

We strongly agree that our results are consistent with previous studies in the social sciences regarding stereotypes on indigenous people. Additionally, we do not deny the current existence of societal skin color inequality and discrimination in the studied Mexican population. In fact, we propose that these behaviors are the result of these historical social pressures and cultural transmission.

Finally, the deliberate choice for alluding these concepts up until page 4 was because the initial segment of our introduction aimed to elucidate the physiological underpinnings of skin color evolution within the human species. It's important to note that this emphasis was not indicative of a lack of recognition or engagement with the prevailing issues of contemporary societal disparities related to skin color. To be more precise with these assertions, we have expanded the information in introduction (lines 101-120) and discussion (lines 569-585). In addition, we have modified the abstract to more clearly incorporate these aspects mentioned above (Lines 60-67).

**Comment:**

2. My other comment is on the methods, which have implications for the substantive interpretations. Reviewers 2 and 3 also seem to have alluded to this. The authors claim that there is no information about the ethnicity of the stimuli subjects but that in one treatment, only skin color and not facial features, was changed. But skin color is the information! Darker skin color in Mexico suggests that persons are more indigenous. Note that skin color, like indigeneity, is fluid and on a continuum, especially between mestizos and indigenous people (see for example Villarreal 2013 in the American Sociological Review and Flores et al 2023 in the American Journal of Sociology).

**Response:**

Thank you for this insightful observation. Indeed, this aspect holds pivotal significance for our proposition. Precisely, the aim of this study was to ascertain whether skin color serves as a conduit of information influencing social perceptions. In our study we supported this premise experimentally by altering skin color according to the facial color patterns of these two racial groups. Consequently, we propose that skin color can be employed to associate ethnicity with its learned social stereotypes, and this interplay could potentially underpin social perceptions. These two elements, coloration and stereotype, would not be intertwined without a long and robust process of cultural learning and transmission that facilitates the stereotyping of a racial group based on a distinguishing phenotypic trait (i.e., skin color). In fact, this forms the bedrock of our proposition concerning cultural transmission and social learning in the evolution of human culture. We elucidate and expand upon this information in lines 484-492. Aligned with this notion, what holds significance here is that the color-ethnicity-perception nexus has evolved as a consequence of learned cultural pressures that have delineated what is socially favorable (or unfavorable) based on an evaluation of skin color—a physical attribute rooted in a distinct evolutionary origin (i.e., environmental pressures due to UV radiation). In contemporary times, this attribute is also harnessed for categorization, stereotyping, and modulation of social perceptions. This historical social pressure revolving around skin color could furthermore give rise to components of discrimination and racism within modern Mexican society. These intricate connections have been deeply interwoven throughout the history of Latin America and disentangling them proves challenging when attempting to elucidate their compounded effects. We clarify and expand upon this information in lines 569-585. Lastly, while acknowledging that disentangling racial stereotypes from skin color presents a formidable challenge, we believe that our study could serve as useful reference for future endeavors in social sciences that delve into this issue with the necessary depth. We do encourage this vision in the paragraph previously reported (lines 569-585). We also included a paragraph to explain that skin color and ethnicity form a continuous gradient, rather than a categorical distinction as implemented in the current study. Therefore, future research is needed to ascertain the extent to which these social perceptions are influenced by the fluid continuum of these two dimensions. Please see lines 536-548.

**Reviewer #2:**

**Comment:**

The manuscript could be better written. I have attached a corrected version of the PDF (see pages 16 to 51). Otherwise, I have no issues with the revised manuscript.

**Response:**

We greatly appreciate the reviewer's effort and time in providing us with precise suggestions, which will significantly improve our manuscript. Therefore, we have rewritten all sentences. Once again, thank you very much.

**Reviewer #4:**

**Comment:**

1.	It’s a bit surprising that Lightness (L*) is the only parameter considered in this study whereas the aim is to investigate the influence of skin pigmentation on social judgments.

Obviously, the reason given in line 222 (lightness has a negative correlation with melanin) is not rational. In fact, melanin pigmentation is not only negatively affects the skin’s lightness, but possibly even more strongly and positively linked to skin yellowness. And it also influences skin redness. See for example the below reference. There are also other skin pigmentations. If it’s only lightness (L*) is investigated, the title and the scope should be restricted to the effect of ‘skin lightness’ not ‘skin pigmentation‘, on facial impressions. Otherwise, it’s not fair to study the influence of skin colour on facial impressions only based on L* values.

“Fruit over sunbed: Carotenoid skin colouration is found more attractive than melanin colouration,” Q. J. Exp. Psychol., vol. 68, no. 2, pp. 284–293, Feb. 2015, doi: 10.1080/17470218.2014.944194.

Kikuchi, Kumiko, et al. "Image analysis of skin colour heterogeneity focusing on skin chromophores and the age‐related changes in facial skin." Skin Research and Technology 21.2 (2015): 175-183.

**Response:**

Thank you for this comment. Skin pigmentation arises from a complex interplay of three primary pigments: melanin, hemoglobin, and carotenoids, along with other components like collagen. In our study, we focused on the dimension of skin pigmentation linked to melanin, which spans from light to dark, given its salience and significant variation among human populations. Our method involved creating prototypes based on the average skin color values of each group and then using the differences between these prototypes as a "mask" to generate the two skin color versions. Consequently, because our manipulation was based on broad average values, the resulting stimuli exhibited variations not solely in the lightness parameter but across all dimensions; in essence, since the skin color dimension we focused on is inherently embedded within the broader context of "skin pigmentation", and given that we didn't exclusively modify lightness while controlling over other parameters, we chose to use the term "skin pigmentation" instead of "skin lightness."

On the other hand, we need to make it clear that we calculated the CIELab values to determine if the skin color in each of the two groups (natural lighter from European American faces, and natural darker from Me´phaa faces) had observable and contrasting color properties. Here, we found that color properties effectively differed between groups. However, we chose lightness as it was the color parameter that contrasted the most in our groups and it could also influence other properties (like yellowness). We explained this in the manuscript, see lines 203-2010, and in 213-217.

Now, the statement we made in line 222(i.e., previous version line) is well-founded. Alaluf et al. (2002) compared measurements of human skin color (CIELab parameters) with biochemical measurements of melanin content, composition, and melanosome size in skin biopsies. They discovered a robust negative correlation between lightness and total melanin content, as well as a weaker yet significant negative correlation between lightness and melanosome size. Although a positive correlation between yellowness and total melanin content was identified, it appeared to be influenced by lightness. Additionally, no other substantial correlations were found between yellowness and the other biological measurements. Given these considerations, we believe that this parameter best represents the dimension we focused on in our objectives. Nevertheless, for a better understanding of the use of this parameter in our study, we removed the association of this parameter with the biochemical relationships with melanin, since, in line with the reviewer, other color parameters could be affected by this pigment, and because determining the specific relations between the three parameters of color and the social perceptions is just out of the scope of this study (Please see lines 212-217). Having said this, we also believe that investigating how each facet of skin color contributes to the perception of this population could open up intriguing avenues for further research. This notion is elaborated upon in the discussion (Line 543-548).

**Comment:**

2.	The creation of facial stimuli is unusual. Two averaged faces were created first and then the selected eight facial stimuli were either added or subtracted from the averaged face. Is there any reason for doing that? It is also not clear how the skin colour manipulation was done (lines 233-242). As the selected eight faces have different face shapes/ features/ colours/ colour distributions from the prototype, how the colour was added or subtracted without holding the facial shape unchanged? Whether the colour change was also applied to the facial features (eyes, mouth, brows). Any process for those facial features? Why was 70 per cent particularly selected? What software was used? It would be helpful to specify the detailed process.

**Response:**

We appreciate the comment. The software we used was Psychomorph and we did specify this information in the section named “Skin color facial stimuli” (now lines 218-220). Psychomorph has been used in numerous studies on face perception since it allows the independent manipulation of shape and color properties in facial photographs. To conduct the manipulations, the initial step involves outlining the facial contours of all photographs. Subsequently, two prototypes are fashioned, each representing the averaged values of the extreme points corresponding to the facial feature targeted for manipulation. Finally, using these two prototypes as reference, it is possible to create different versions of the same stimuli varying the facial feature based on the differences between the two prototypes (for a deeper understanding about how the software works and its details, we strongly recommend entering the following link: [https://users.aber.ac.uk/bpt/jpsychomorph/](about:blank)).

Now, the sum or subtraction was made applying in each face the two color “mask” of the color prototypes. This was previously described and clarified in the section “Skin color facial stimuli” (Lines 218-239). Moreover, the setting of 70% of difference was used as a rule of thumb that the software developers recommend in order to avoid visual aberrations. This percentage not only prevents these visual aberrations to happened, but also ensures a significant change in color properties among the stimuli within the observable and feasible ranges for each ethnic group.

Finally, just like the unintended effect that occurred in texture homogenization because of the methodology we employed, a similar effect was observed in the color transformation, which extended to the eyes, mouth, and eyebrows. Our strategy to address this side effect involved comparing the ratings of the original versions with those of the corresponding versions with transformed skin color. This helped us measure the impact of the color homogenization effect. Nevertheless, we clarified this limitation in lines 531-548

**Comment:**

3.	Apart from lightness (L*), the facial stimuli seem to show a large difference in yellowness (b*). Is that true? Since authors already ‘measured its skin colour values in CIELab colour space (line 218)’, it would be necessary to show the colour parameters in CLELAB colour space (including L*, a*, and b*) for all the face stimuli from both European American and Me’phaa groups, not just L*.

**Response:**

We appreciate this comment. As mentioned earlier, our calculation of the CIELab values primarily served as a validation method for selecting faces and for our manipulation process. In the response to comment 1 you can find all the information regarding why we used “L” as well as the lines manuscript we modified to clarify this important observation.

Additionally, emphasizing one of the three color parameters based on its significance to the study is a customary practice in facial perception research (as seen in Kleisner et al., 2017).

**Comment:**

4.	It’s not clear about the colour information of facial stimuli. Whether the facial stimuli used in this study could represent the naturally occurring skin colour variations in the two populations, European American and Me’phaa. The average colour or the prototype is not enough as the variations within the same ethnic group are usually larger than the variations across the two ethnic groups. See the below reference. Thus, it would be very helpful to show the evolutionary meaningful skin colour range and variation of the real human faces from those two groups.

“Characterising the variations in ethnic skin colours: a new calibrated database for human skin,” Ski. Res. Technol., vol. 23, no. 1, pp. 21–29, Feb. 2017, doi: 10.1111/srt.12295.

**Response:**

Thanks for this comment. Although our study standardizes skin color towards an "average" color, we also included the "Natural" group; which mantain individual variations for each ethnic group (8 natural Me'phaa faces and 8 European American faces). When comparing the natural group (with individual color variations) and its corresponding "average" group, perceptions don't change significantly, except for health. This might partially tell us that intra-groups color variation has less impact than the categorical (and experimental) change from one "average" skin color to the other one (i.e., Dark to Light and vice versa). We also included random effects for each evaluated facial photograph in our models. Here, we noticed that this random effect plays an important role in perceptions, but the fixed effect (transformed groups) consistently affects these individual variations across the groups. This demonstrates a systematized effect of contrasting "skin" color groups, despite the random effect of natural variation. Additionally, the obtained average skin colors are within the typical range found in Mexican and European American populations. We've rewritten a section to clarify our rationale (lines 201-208 and 211-215).

Regarding the acknowledgement of the evolutionarily meaningful skin color range and variation of real human faces, we did provide a summary of human skin color evolution in the first section of the introduction (lines 75-90). Here, we emphasize that studying the evolution of human skin color has many aspects that have become more complex to describe the whole color diversity in human groups (e.g., microclimates, migration patterns, angles of UV radiation, etc.). Many of these factors are related to natural selection. In our case, we documented how the perception of human skin color, a trait shaped by natural selection, may have implications in other processes like sexual and social selection, as well as cultural transmission. This has already been clarified in the introduction and discussion.

**Comment:**

5.	Line 240-242: ‘The average lightness value was L* = 66.74, SD = 1.37 in the light skin colour transformed versions and L* = 52.75, SD = 2.58 in the dark skin colour transformed versions.’ Are the light versions including both light European American and light Me’phaa faces and the dark versions include both dark European American and dark Me’phaa faces? If so, the standard deviation looks very small. Does that mean the European American and Me’phaa faces have the same lightness levels after manipulations? How does that come if both their original lightness and the prototype are different? It’s better to give the actual colour distributions of the 48 face stimuli, not only the average lightness values.

**Response:**

Thank you for bringing up this point. Effectively, our study employs a crossover design. Hence, we obtained a total of 6 groups, which include a version darker and a lighter for each natural color group. Moreover, as we've clarified before, the process of manipulating skin color entailed applying a "color mask" derived from the two skin color prototypes. Consequently, within these versions, the transformed groups with paired colors (2 lighter and 2 darker) did not show significant variation in lightness. This outcome aligns with expectations, given that the color manipulation was conducted in a similar manner for both natural color groups. Furthermore, we observe that the lightness (L) of the transformed color groups (darker and lighter) remains consistent with their corresponding natural groups, as well as with what is commonly found and naturally distributed in both populations. We have provided clarification on this topic in lines 211-215 and 228-235 to enhance comprehension.

**Comment:**

6.	It looks like the same facial stimuli changed not only the skin colour but also some other facial appearance traits after manipulations. E.g. in the below, the stimuli FC16, FC16co, and FC16cb are manipulated versions from the same original face, FC10, FC10cb, FC10co are from the same face. If that’s the case, it looks like the beard completely changed after the colour changed, especially for the light version, the beard disappeared in the meantime. Those appearance traits could significantly influence sexual dimorphism and are even more sexually dimorphic traits than skin colour, and thus affect social judgements, especially judgements from the other gender.

**Response:**

Although the effect of facial sexually dimorphic traits, such as beard, is widely acknowledged, in our study it did not seem to be a significant factor. In the next forest plot we show the perceived attractiveness random estimates for all three skin color versions for each of the 16 faces (form right to left: natural version, light skin color version, dark skin color version).

The forest plot shows that none of the two natural versions (FC10, FC16) got significant higher or lower ratings than the main estimate (right column). It also shows that removing the beard did not affect the rates significantly in comparison with the main estimate for each version (light skin color version, middle column; dark skin color version, left column). We have performed the same exploration for all response variables, and we found a similar result for all of them except for the perceived masculinity ratings, however, even those differences were not significant. The rest of the forest plot can be found in the R Markdown code lines 364, 594, 720, 851, and 984).

**Comment:**

7.	The facial gloss could have a big influence on the lightness measurement results based on image methods. E.g. the stimulus FI15 below seems to have a considerable area of highlight. Within these areas, the colour could be completely different from the normal skin colour. Any considerations regarding the facial gloss issue? And how was the gloss morphed into different colour versions?

**Response:**

Thank you for the observation. The skin color prototypes were created by averaging the values of 30 faces of each group. One of the reasons why we had that sample size was to minimize the effect of any kind of individual variation such as the one mentioned; moreover, we did not use the actual lightness values to test the effect of skin color. Now regarding a possible difference in the way the raters perceived this stimuli in all of its versions, a random effect exploration showed that there are no significative differences between the scores this stimuli got and the main estimate for each version. We have included foster plots that show this in the R Markdown (code lines 364, 594, 720, 851, and 984).

**Comment:**

8.	Images were captured under different environments and camera settings. It would be good to give information on settings, such as the white balance, CCT, ISO, aperture size, shutter speed, image formats (raw or post-processed), any colour characterization process, etc.

**Response:**

We appreciate this observation. We understand the relevance of giving as much information on settings as possible, however the information written in the lines 190 – 200 is all the setting information we took. Regarding the color characterization process, we followed the color calibration methodology reported in lines 208-217 which is a common method in the facial perception studies.

**Comment:**

9.	During the experiments, participants were asked to rate the face without particular restriction or focus, e.g. based on the facial skin colour. In such a case, it’s only meaningful to compare the rating of the lighter, natural, and darker versions of the same face. It’s not reasonable to compare different faces or faces of different ethnic groups as the influence of structural facial traits is tangled together. However, those structural facial traits are not quantified or included in the analysis. in fact, previous studies show colour is less important than the structural facial traits in face perceptions. Meanwhile, various facial colour cues may be involved in face perceptions. See the reference below. E.g. the skin colour variation may matter a lot (homogeneous skin is preferred), which links to the colour and texture homogenization mentioned in the current study. Are there any considerations in the data analysis related to those confounding variables in visual stimuli? “Different colour predictions of facial preference by Caucasian and Chinese observers,” Sci. Rep., vol. 12, no. 1, p. 12194, Jul. 2022, doi: 10.1038/s41598-022-15951-8.

**Response:**

We appreciate this comment. A complete explanation about why we compared the ratings between the natural versions of the European American and Me’Phaa faces can be found in the answer to the comment number 15. Now, regarding the considerations to the possible confounding effect of shape and color homogenization, we took different approaches. We designed a crossing over study where the skin color version is nested within the group in order to control the effect of facial shape; and we did so by creating different skin color version of the same facial stimuli. On the other hand, we are aware that one of the side effects of our protocol was the homogenization of color and texture, and we know this may have an effect on some of the perceptions we evaluated, so we compare the ratings of the natural versions with the ratings of the concordant skin color transformed version so we could have a measurement of this effect. Our results showed that, whereas skin color and texture homogenization seemed to affect some the perceptions, especially perceived health, in general the effect was either not significant or lower that the one due to skin color change. Nonetheless, we recognize this limitation and, hence, we highlight it in the discussion (lines 531-548).

**Comment:**

10.	In the linear mixed effect model, the face colour was coded as categorical variable. But the same colour category has different lightness levels, e.g. the natural European American face and the natural Me’phaa face have different L*, and the lighter versions of different faces have different L*... Why not use the actual lightness (L*) value as a continuous variable in the lme analysis? Actual lightness value could be a more accurate measure for assessing the effect of skin lightness on impression judgements. The fixed effect of other colour parameters, such as a* and b*, could be included in the lme analysis as well.

**Response:**

We coded the skin color as a categorical variable because it is a more reasonable way to carry out the statistical analysis of a crossover research design. In addition, using lightness values as a continuous variable would present some major inconveniences for the data that we have. Firstly, continuous variables cannot be included as random factors, so we could not be able to deal with the lack of independency of our observations (the ratings come from three versions of the same faces). Secondly, since the skin color manipulation was based on average values, the lightness values´ distribution would behave in a “categorical” fashion, that is, the values would group in three points instead of being constantly distributed in the whole range of variation. The decision about what type of variable most be used is not based on the accuracy of the measurement but in the type of variable that fits with the specific needs of the research design.

**Comment:**

11.	What’s the observer variation? Any evaluation of the consistency across different raters.

Of the observer variations we assessed, factors such as sex, age, sexual preference, self-perception of ethnic group, and skin color were included. These variations and their effects are reported in the section titled "Facial Perception Rates." Given that we recognize the relevance of the individual observer variation more than variables we measured them, we also decided to include “Rater” as a random factor in our mixed models. We know that, traditionally, a measure such as the Cronbach's alpha is calculated to know the participants response reliability. However, we think that our approach is better because mixed models do not only measure this sort of variance but also take it into account when they estimate the standard errors.

**Comment:**

12.	Are there any differences in the results rated by mestizo participants and European participants? Is there any effect of the participants’ ethnicity on ratings?

**Response:**

We appreciate this question. We did not find that the raters’ ethnicities had an effect on their responses; and, as a matter of fact, this question was already asked by the first reviewer during the first submission and we wrote our answer with the whole process that led us to our conclusion in the statistical analysis section, lines 313-318.

**Comment:**

13.	What’s the relationship between participants’ self-perceived skin colour ratings and their actual skin colour? Has their skin colour ever been measured by instruments like spectrophotometers? There might be a mismatch between self-reported and measured values.

**Response:**

This is an interesting question. Based on the literature available we do believe that there may be a mismatch between self-reported, reported by someone else and objective measures of skin color in Mexico and in other Latin American countries. In these regions, individuals could perceive themselves as lighter compared to others' perceptions and even lighter than what objective measures indicate. Despite the widespread theoretical consideration of this bias, to our knowledge, no studies have directly addressed this matter within Latin American populations. This highlights a significant knowledge gap that presents a substantial opportunity for investigation. With that being said, we deem this concept to extend beyond the scope of our current study, and we recognize that a completely new study is required to comprehensively examine and test this hypothesis.

**Comment:**

14.	It would be interesting to know whether there are any significant differences in the structural facial traits (facial shapes and facial features) between the European American and Me’phaa faces, and what are the differences. It might be good to give some evidence based on the image database used in the present study.

**Response:**

This is an interesting question. A recent study found that some components of facial shape do vary across populations (Kleisner et al., 2021), and ethnicity is usually a significant predictor of facial shape variation in geometric morphometric studies. Therefore, we do expect differences in the facial shape traits between the European American and Me’phaa faces, nonetheless, similar to the previous answer, we think that this idea is beyond the reach of our study. In this study we only focused on controlling the effects of shape, however, as suggested, we are currently working on a study on facial shape, using the same image database, where we sought to test the effect of shape variation on the same perceptions, and we model the facial shape configurations that best correlate with each of the perceptions in combination with skin color

**Comment:**

15.	Both the abstract line 51-53 (‘We found that….faces of …were.. more attractive….’ ) and the discussion line 476-479 (‘In comparison with….’. ) gives an unreasonable conclusion. This conclusion is not justifying the effect of skin colour on facial impression but makes judgements which are out of the scope of the current study. It’s hard to tell whether those comparisons were made based on the L* or other skin colour information or texture or the structural facial traits of the two ethnic groups. From what has been written, the scope of this study is to understand the role of skin lightness on face perceptions, not to compare the attractiveness of the two different ethnic faces. And it’s not rational to make those comparisons on the bases of very different both structural facial traits and colour traits.

16.	Similar issues for those statements in the results part: European American faces were rated ‘x times’ more attractive/trustworthy… than Me’phaa faces. Current data and analysis can only show the effect of skin colour, but cannot tell whether those ‘x times’ come from colour or other factors, also cannot prove the link between ‘which ethnic face is more attractive’ and ‘colour plays a role in it’.

**Response:**

We agree with these two reviewer comments. Indeed, the focus of this study is to provide experimental evidence of how skin color affects social perceptions in a Mexican population, not to compare social perceptions of two ethnic groups. These groups not only differ in skin color but also in many other physical and cultural attributes, as previously mentioned. To make this objective clearer, we've made changes in the lines the reviewer mentioned and through the whole manuscript, directing the interpretation towards skin color and away from other facial or cultural attributes specific to ethnicity. Please see lines 54-57, and the results section for these modifications.

On the other hand, we used the term "x times" because it is the accurate way to describe differences when the estimated effect comes from a Poisson GLM. In this type of GLM, the effects are expected to be exponential. Therefore, when incorporating a categorical predictor, the estimates are expressed in terms of how many times it is greater or lesser compared to the baseline, instead of a fixed and continuous difference. Although we retained these terms ("x times"), we shifted the focus to skin color (evaluated in the L dimension) and not towards an "ethnic group." Thank you for this important observation.

**Comment:**

17.	Whether the current results lead to the hypothesis? Skin colour preference/social preference also includes some mainstream aesthetic criteria, e.g. tanning skin is preferred in Western countries while whitening skin is preferred in Eastern countries, which may have nothing to do with racism or discrimination. Have these factors been considered in the current study? From the current data and analysis, it seems to be hard to elucidate the convincing mechanisms or drives behind the phenomenon. More survey and investigation is needed to know the clear answer.

**Response:**

This is an interesting question. We agree with the statement that some mainstream aesthetic criteria may play a role in the skin color preferences and that preference for tanning skin in Western countries is one example of it. However, skin color-based stratification and racism were in the very foundation of Latin American societies and still have strong effects on their social dynamic. Therefore, we think this is a more plausible factor that could explain the social preferences reported here. Nonetheless, we share the opinion that more investigation is needed to elucidate the relative contribution of mainstream aesthetic criteria on skin color preferences.

**Comment:**

18.	Are there any considerations related to the adaptation to the living environment? Raters might be exposed to different environments composed of a variety of coloured faces. The long-term adaptation to the environment might influence their perceptions. It might be helpful to provide some evidence or data about the variety/proportions of faces in the participants’ living environment.

**Response:**

Thank you for this observation. There are some studies that suggest that people are better at judging familiar faces, such as faces within the same ethnicity. We did propose this possibility as an explanation of the differences we observed in the effects of skin color between the two groups. We have rearranged the paragraph we had previously written to make it more visible (lines 549-557). It is important to point out that our discussion comes from an approximated measurement because there is not available data that allowed us to test this more precisely.

**Comment:**

19.	Line 90: it would be more precise to say ‘lower and more seasonal UVR’.

**Response:**

Thank you for this observation. We have changed the term.

Alaluf, S., Atkins, D., Barrett, K., Blount, M., Carter, N., & Heath, A. (2002). The impact of epidermal melanin on objective measurements of human skin colour. *Pigment Cell Research*, *15*(2), 119–126. https://doi.org/10.1034/j.1600-0749.2002.1o072.x

Kleisner, K., Kočnar, T., Tureček, P., Stella, D., Akoko, R. M., Třebický, V., & Havlíček, J. (2017). African and European perception of African female attractiveness. *Evolution and Human Behavior*, *38*(6), 744–755. https://doi.org/10.1016/j.evolhumbehav.2017.07.002

Kleisner, K., Tureček, P., Roberts, S. C., Havlíček, J., Valentova, J. V., Akoko, R. M., Leongómez, J. D., Apostol, S., Varella, M. A. C., & Saribay, S. A. (2021). How and why patterns of sexual dimorphism in human faces vary across the world. *Scientific Reports*, *11*(1), 1–14. https://doi.org/10.1038/s41598-021-85402-3
